# Evaluating AI in medicine: a comparative analysis of expert and ChatGPT responses to colorectal cancer questions

**DOI:** 10.1038/s41598-024-52853-3

**Published:** 2024-02-03

**Authors:** Wen Peng, Yifei feng, Cui Yao, Sheng Zhang, Han Zhuo, Tianzhu Qiu, Yi Zhang, Junwei Tang, Yanhong Gu, Yueming Sun

**Affiliations:** 1 Department of General Surgery, The First Affiliated Hospital with Nanjing Medical University, Nanjing, 210029 Jiangsu People’s Republic of China; 2 The First School of Clinical Medicine, Nanjing Medical University, Nanjing, China; 3 Department of Radiotherapy, The First Affiliated Hospital with Nanjing Medical University, Nanjing, People’s Republic of China; 4 Department of Intervention, The First Affiliated Hospital with Nanjing Medical University, Nanjing, People’s Republic of China; 5 Department of Oncology, The First Affiliated Hospital with Nanjing Medical University, Nanjing, People’s Republic of China

**Keywords:** Colorectal cancer, Health care

## Abstract

Colorectal cancer (CRC) is a global health challenge, and patient education plays a crucial role in its early detection and treatment. Despite progress in AI technology, as exemplified by transformer-like models such as ChatGPT, there remains a lack of in-depth understanding of their efficacy for medical purposes. We aimed to assess the proficiency of ChatGPT in the field of popular science, specifically in answering questions related to CRC diagnosis and treatment, using the book “Colorectal Cancer: Your Questions Answered” as a reference. In general, 131 valid questions from the book were manually input into ChatGPT. Responses were evaluated by clinical physicians in the relevant fields based on comprehensiveness and accuracy of information, and scores were standardized for comparison. Not surprisingly, ChatGPT showed high reproducibility in its responses, with high uniformity in comprehensiveness, accuracy, and final scores. However, the mean scores of ChatGPT’s responses were significantly lower than the benchmarks, indicating it has not reached an expert level of competence in CRC. While it could provide accurate information, it lacked in comprehensiveness. Notably, ChatGPT performed well in domains of radiation therapy, interventional therapy, stoma care, venous care, and pain control, almost rivaling the benchmarks, but fell short in basic information, surgery, and internal medicine domains. While ChatGPT demonstrated promise in specific domains, its general efficiency in providing CRC information falls short of expert standards, indicating the need for further advancements and improvements in AI technology for patient education in healthcare.

## Introduction

Colorectal cancer (CRC) remains a leading cause of cancer death worldwide, with an increased incidence of morbidity and mortality in recent years^1^. Especially in China, survival rates for CRC patients remain low despite medical advancements^2^. This necessitates a focus on early detection, effective treatment, comprehensive care, and patient support^3,4^.

However, a lack of accessible knowledge often results in patients overlooking crucial signs and delaying their first visit to the hospital^5^. Additionally, misleading information online may lead to misdiagnosis or improper treatment^3,6,7^. While there are valuable resources like UpToDate^8^ and MSD Manuals^9^ that provide peer-reviewed information, they are primarily tailored towards healthcare professionals rather than patients. To address this issue, it is essential to develop reliable online platforms specifically designed for patient education, which are accessible easily. Moreover, these platforms should present accurate and easily understandable medical information and incorporate clear guidelines on when to seek medical attention. By making this, patients can have a trustworthy source of information that empowers them to make informed decisions about their health.

ChatGPT, powered by large language models (LLMs) and deep learning-based AI systems, truly herald a new epoch in the realm of AI technologies^10^. In 2022, the noteworthy launch of GPT's version 3.5 (GPT-3.5) has underscored this fact even more^11^. With their vast capacities, these transformer-like models, including ChatGPT, can respond to a wide range of prompts, making them highly adaptable to various applications, particularly in health care^12–14^. These new technologies give the chance to bridge the gap between medical knowledge and patient understanding, empowering patients to make informed decisions about their health, ultimately improving early detection, diagnosis, and treatment outcomes^15–17^.

Despite being trained on an extensive data, and exhibiting promising potential in the realm of medicine, we still need to fully understand the efficacy and applicability of ChatGPT in medicine. It is therefore important to establish comprehensive data and research to assess their true capabilities in the medical field. To this end, we have referred to “Colorectal Cancer: Your Questions Answered” published in China^18^, to evaluate ChatGPT’s proficiency in popular science, specifically regarding patient questions of CRC diagnosis and treatment, by comparing its responses to the book’s answers.

## Results

### The high reproducibility of ChatGPT’s dual responses

**Figure 1:**
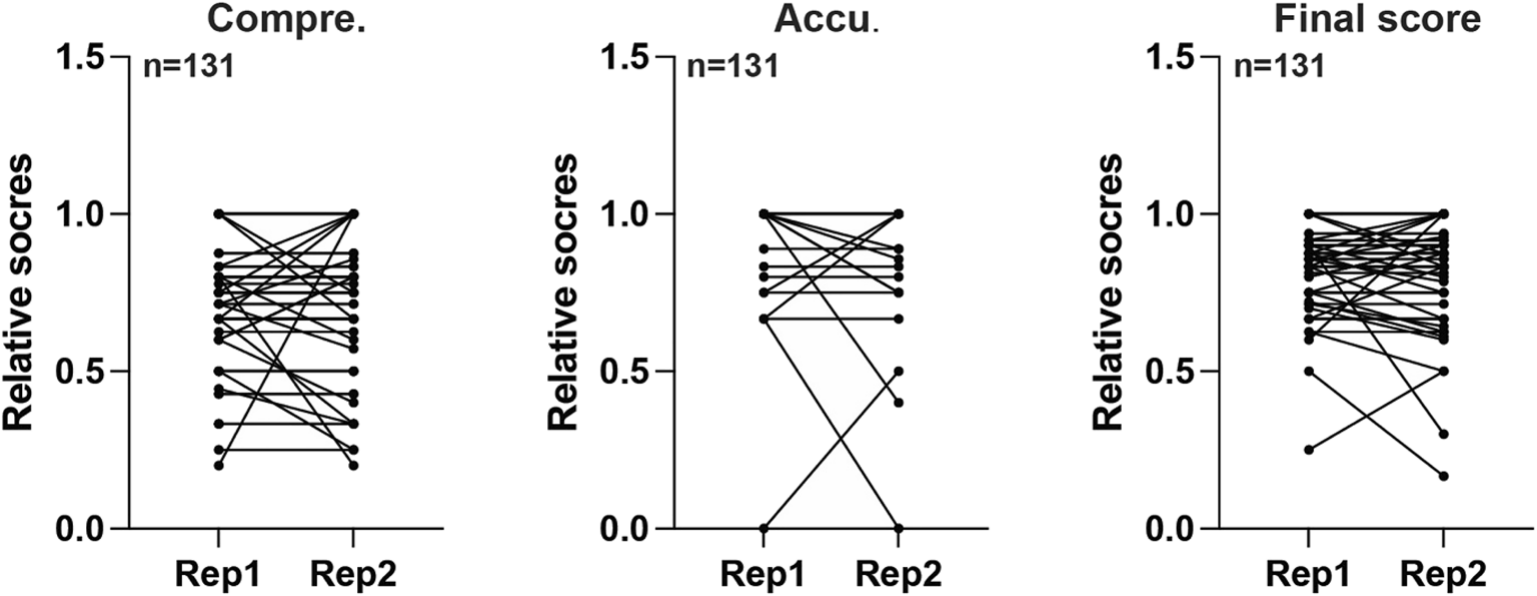
The high reproducibility of ChatGPT’s responses. The similarity between the first and second responses generated by ChatGPT for each query, and the comprehensiveness, accuracy and overall scores were assessed.

A total of 131 valid questions from the book were answered by ChatGPT (Appendix file). The primary and standardized scores of each question were assessed by physicians (Tables 1, 2, 3, 4, 5, 6, 7 and 8). In our initial examination, we scrutinized the reproducibility of ChatGPT, results indicated that in most of the sections, ChatGPT’s pair responses possessed a high level of uniformity in terms of comprehensiveness (n = 131, p = 0.6403), accuracy (n = 131, p = 0.5703), and final scores (n = 131, p = 0.6162) (Fig. 1A). On average, the comprehensive scores for the two responses were 0.86 and 0.85, the accuracy scores were 0.98 and 0.97, and the final scores were 0.92 and 0.91, respectively.

**Table 1:** Relative and standardized scores of answers provided by experts and ChatGPT.

The basic information					
	Benchmark				
	*Compre.	^#^Accu.	Compre.	Accu.	Final
1	8	8	1	1	1
2	8	8	1	1	1
3	4	4	1	1	1
4	7	7	1	1	1
5	6	6	1	1	1
6	10	10	1	1	1
7	6	6	1	1	1
8	9	9	1	1	1
9	3	3	1	1	1
10	6	6	1	1	1
11	6	6	1	1	1
12	8	8	1	1	1
13	4	4	1	1	1
14	6	6	1	1	1

	ChatGPT				
	Compre.	Accu.	Compre.	Accu.	Final
1	7	8	0.875	1	0.9375
2	7	8	0.875	1	0.9375
3	4	4	1	1	1
4	5	7	0.71428571	1	0.85714286
5	5	6	0.83333333	1	0.91666667
6	8	10	0.8	1	0.9
7	4	5	0.66666667	0.83333333	0.75
8	4	9	0.44444444	1	0.72222222
9	3	3	1	1	1
10	4	6	0.66666667	1	0.83333333
11	6	6	1	1	1
12	5	8	0.625	1	0.8125
13	4	4	1	1	1
14	5	6	0.83333333	1	0.91666667

	ChatGPT Rep2				
	Compre.	Accu.	Compre.	Accu.	Final
1	7	8	0.875	1	0.9375
2	7	8	0.875	1	0.9375
3	4	4	1	1	1
4	5	7	0.71428571	1	0.85714286
5	5	6	0.83333333	1	0.91666667
6	8	10	0.8	1	0.9
7	4	5	0.66666667	0.83333333	0.75
8	3	8	0.33333333	0.88888889	0.61111111
9	3	3	1	1	1
10	4	6	0.66666667	1	0.83333333
11	6	6	1	1	1
12	5	8	0.625	1	0.8125
13	4	4	1	1	1
14	5	6	0.83333333	1	0.91666667

*Compre. (Comprehensiveness of Information).

^#^Accu. (Accuracy of Information).

**Table 2:** Relative and standardized scores of answers provided by experts and ChatGPT.

Surgery management					
	Benchmark				
	Compre.	Accu.	Compre.	Accu.	Final
1	5	5	1	1	1
2	4	4	1	1	1
3	3	3	1	1	1
4	3	3	1	1	1
5	7	7	1	1	1
6	6	6	1	1	1
7	9	9	1	1	1
8	4	4	1	1	1
9	4	4	1	1	1
10	6	6	1	1	1
11	3	3	1	1	1
12	5	5	1	1	1
13	4	4	1	1	1
14	4	4	1	1	1
15	4	4	1	1	1
16	4	4	1	1	1

	ChatGPT				
	Compre.	Accu.	Compre.	Accu.	Final
1	4	5	0.8	1	0.9
2	4	4	1	1	1
3	3	3	1	1	1
4	3	3	1	1	1
5	5	7	0.71428571	1	0.85714286
6	6	6	1	1	1
7	7	8	0.77777778	0.88888889	0.83333333
8	4	4	1	1	1
9	3	4	0.75	1	0.875
10	6	6	1	1	1
11	2	3	0.66666667	1	0.83333333
12	1	5	0.2	1	0.6
13	2	4	0.5	1	0.75
14	4	4	1	1	1
15	4	4	1	1	1
16	3	4	0.75	1	0.875

	ChatGPT Rep2				
	Compre.	Accu.	Compre.	Accu.	Final
1	4	5	0.8	1	0.9
2	4	4	1	1	1
3	3	3	1	1	1
4	3	3	1	1	1
5	6	7	0.85714286	1	0.92857143
6	6	6	1	1	1
7	7	8	0.77777778	0.88888889	0.83333333
8	4	4	1	1	1
9	3	4	0.75	1	0.875
10	6	6	1	1	1
11	2	3	0.66666667	1	0.83333333
12	5	5	1	1	1
13	2	4	0.5	1	0.75
14	4	4	1	1	1
15	4	4	1	1	1
16	4	4	1	1	1

*Compre. (Comprehensiveness of Information).

^#^Accu. (Accuracy of Information).

**Table 3:** Relative and standardized scores of answers provided by experts and ChatGPT.

Internal medicine					
	Benchmark				
	Compre.	Accu.	Compre.	Accu.	Final
1	5	5	1	1	1
2	4	4	1	1	1
3	4	4	1	1	1
4	3	3	1	1	1
5	4	4	1	1	1
6	4	4	1	1	1
7	5	5	1	1	1
8	7	7	1	1	1
9	6	6	1	1	1
10	8	8	1	1	1
11	4	4	1	1	1
12	5	5	1	1	1
13	3	3	1	1	1
14	4	4	1	1	1
15	5	5	1	1	1
16	7	7	1	1	1
17	Repeated				
18	5	5	1	1	1
19	4	4	1	1	1
20	3	3	1	1	1
21	3	3	1	1	1
22	2	2	1	1	1
23	4	4	1	1	1
24	4	4	1	1	1
25	4	4	1	1	1
26	2	2	1	1	1
27	4	4	1	1	1
28	7	7	1	1	1
29	5	5	1	1	1
30	2	2	1	1	1
31	2	2	1	1	1
32	4	4	1	1	1
33	4	4	1	1	1
34	3	3	1	1	1
35	5	5	1	1	1

	ChatGPT				
	Compre.	Accu.	Compre.	Accu.	Final
1	4	5	0.8	1	0.9
2	1	4	0.25	1	0.625
3	4	4	1	1	1
4	3	3	1	1	1
5	3	4	0.75	1	0.875
6	4	4	1	1	1
7	3	4	0.6	0.8	0.7
8	5	7	0.71428571	1	0.85714286
9	4	6	0.66666667	1	0.83333333
10	4	8	0.5	1	0.75
11	3	4	0.75	1	0.875
12	4	5	0.8	1	0.9
13	3	3	1	1	1
14	4	4	1	1	1
15	3	5	0.6	1	0.8
16	5	7	0.71428571	1	0.85714286
17					
18	4	5	0.8	1	0.9
19	2	3	0.5	0.75	0.625
20	2	2	0.66666667	0.66666667	0.66666667
21	1	2	0.33333333	0.66666667	0.5
22	2	2	1	1	1
23	2	4	0.5	1	0.75
24	3	4	0.75	1	0.875
25	4	4	1	1	1
26	2	2	1	1	1
27	4	4	1	1	1
28	3	7	0.42857143	1	0.71428571
29	4	5	0.8	1	0.9
30	2	2	1	1	1
31	2	2	1	1	1
32	3	4	0.75	1	0.875
33	2	4	0.5	1	0.75
34	3	3	1	1	1
35	4	5	0.8	1	0.9

	ChatGPT Rep2				
	Compre.	Accu.	Compre.	Accu.	Final
1	4	5	0.8	1	0.9
2	1	4	0.25	1	0.625
3	4	4	1	1	1
4	3	3	1	1	1
5	3	4	0.75	1	0.875
6	4	4	1	1	1
7	2	4	0.4	0.8	0.6
8	4	7	0.57142857	1	0.78571429
9	4	6	0.66666667	1	0.83333333
10	4	6	0.5	0.75	0.625
11	3	4	0.75	1	0.875
12	4	5	0.8	1	0.9
13	3	3	1	1	1
14	3	4	0.75	1	0.875
15	4	5	0.8	1	0.9
16	5	7	0.71428571	1	0.85714286
17					
18	1	2	0.2	0.4	0.3
19	1	3	0.25	0.75	0.5
20	2	3	0.66666667	1	0.83333333
21	1	0	0.33333333	0	0.16666667
22	2	2	1	1	1
23	2	4	0.5	1	0.75
24	3	4	0.75	1	0.875
25	4	4	1	1	1
26	2	2	1	1	1
27	4	4	1	1	1
28	3	7	0.42857143	1	0.71428571
29	4	5	0.8	1	0.9
30	2	2	1	1	1
31	2	2	1	1	1
32	3	4	0.75	1	0.875
33	2	4	0.5	1	0.75
34	3	3	1	1	1
35	3	5	0.6	1	0.8

*Compre. (Comprehensiveness of Information).

^#^Accu. (Accuracy of Information).

**Table 4:** Relative and standardized scores of answers provided by experts and ChatGPT.

Radiation therapy					
	Benchmark				
	Compre.	Accu.	Compre.	Accu.	Final
1	7	7	1	1	1
2	3	3	1	1	1
3	6	6	1	1	1
4	2	2	1	1	1
5	3	3	1	1	1
6	4	4	1	1	1
7	3	3	1	1	1

	ChatGPT				
	Compre.	Accu.	Compre.	Accu.	Final
1	3	7	0.42857143	1	0.71428571
2	3	3	1	1	1
3	4	6	0.66666667	1	0.83333333
4	2	2	1	1	1
5	3	3	1	1	1
6	4	4	1	1	1
7	2	3	0.66666667	1	0.83333333

	ChatGPT Rep2				
	Compre.	Accu.	Compre.	Accu.	Final
1	3	6	0.42857143	0.85714286	0.64285714
2	3	3	1	1	1
3	2	6	0.33333333	1	0.66666667
4	2	2	1	1	1
5	3	3	1	1	1
6	4	4	1	1	1
7	2	3	0.66666667	1	0.83333333

*Compre. (Comprehensiveness of Information).

^#^Accu. (Accuracy of Information).

**Table 5:** Relative and standardized scores of answers provided by experts and ChatGPT.

Interventional therapy					
	Benchmark				
	Compre.	Accu.	Compre.	Accu.	Final
1	3	3	1	1	1
2	3	3	1	1	1
3	3	3	1	1	1
4	4	4	1	1	1
5	3	3	1	1	1
6	2	2	1	1	1
7	3	3	1	1	1
8	5	5	1	1	1

	ChatGPT				
	Compre.	Accu.	Compre.	Accu.	Final
1	3	3	1	1	1
2	3	3	1	1	1
3	3	3	1	1	1
4	3	3	0.75	0.75	0.75
5	3	3	1	1	1
6	1	0	0.5	0	0.25
7	3	3	1	1	1
8	3	5	0.6	1	0.8

	ChatGPT Rep2				
	Compre.	Accu.	Compre.	Accu.	Final
1	3	3	1	1	1
2	2	3	0.66666667	1	0.83333333
3	3	3	1	1	1
4	3	4	0.75	1	0.875
5	3	3	1	1	1
6	1	1	0.5	0.5	0.5
7	3	3	1	1	1
8	4	5	0.8	1	0.9

*Compre. (Comprehensiveness of Information).

^#^Accu. (Accuracy of Information).

**Table 6:** Relative and standardized scores of answers provided by experts and ChatGPT.

Stoma care					
	Benchmark				
	Compre.	Accu.	Compre.	Accu.	Final
1	6	6	1	1	1
2	2	2	1	1	1
3	6	6	1	1	1
4	2	2	1	1	1
5	8	8	1	1	1
6	3	3	1	1	1
7	3	3	1	1	1
8	3	3	1	1	1
9	4	4	1	1	1
10	2	2	1	1	1
11	5	5	1	1	1
12	3	3	1	1	1
13	4	4	1	1	1
14	2	2	1	1	1
15	4	4	1	1	1
16	3	3	1	1	1
17	5	5	1	1	1

	ChatGPT				
	Compre.	Accu.	Compre.	Accu.	Final
1	5	6	0.83333333	1	0.91666667
2	2	2	1	1	1
3	6	6	1	1	1
4	2	2	1	1	1
5	8	8	1	1	1
6	3	3	1	1	1
7	3	3	1	1	1
8	2	2	0.66666667	0.66666667	0.66666667
9	4	4	1	1	1
10	2	2	1	1	1
11	5	5	1	1	1
12	3	3	1	1	1
13	4	4	1	1	1
14	2	2	1	1	1
15	4	4	1	1	1
16	3	3	1	1	1
17	4	5	0.8	1	0.9

	ChatGPT Rep2				
	Compre.	Accu.	Compre.	Accu.	Final
1	6	6	1	1	1
2	2	2	1	1	1
3	6	6	1	1	1
4	2	2	1	1	1
5	8	8	1	1	1
6	3	3	1	1	1
7	3	3	1	1	1
8	2	2	0.66666667	0.66666667	0.66666667
9	4	4	1	1	1
10	2	2	1	1	1
11	5	5	1	1	1
12	3	3	1	1	1
13	4	4	1	1	1
14	2	2	1	1	1
15	4	4	1	1	1
16	3	3	1	1	1
17	4	5	0.8	1	0.9

*Compre. (Comprehensiveness of Information).

^#^Accu. (Accuracy of Information).

**Table 7:** Relative and standardized scores of answers provided by experts and ChatGPT.

Venous care					
	Benchmark				
	Compre.	Accu.	Compre.	Accu.	Final
1	3	3	1	1	1
2	3	3	1	1	1
3	3	3	1	1	1
4	3	3	1	1	1
5	4	4	1	1	1
6	3	3	1	1	1
7	5	5	1	1	1
8	4	4	1	1	1
9	2	2	1	1	1
10	2	2	1	1	1
11	3	3	1	1	1
12	3	3	1	1	1
13	3	3	1	1	1
14	5	5	1	1	1

	ChatGPT				
	Compre.	Accu.	Compre.	Accu.	Final
1	3	3	1	1	1
2	3	3	1	1	1
3	3	3	1	1	1
4	3	3	1	1	1
5	4	4	1	1	1
6	3	3	1	1	1
7	5	5	1	1	1
8	3	4	0.75	1	0.875
9	2	2	1	1	1
10	2	2	1	1	1
11	3	3	1	1	1
12	3	3	1	1	1
13	3	3	1	1	1
14	5	5	1	1	1

	ChatGPT Rep2				
	Compre.	Accu.	Compre.	Accu.	Final
1	3	3	1	1	1
2	3	3	1	1	1
3	3	3	1	1	1
4	3	3	1	1	1
5	4	4	1	1	1
6	3	3	1	1	1
7	5	5	1	1	1
8	3	3	0.75	0.75	0.75
9	2	2	1	1	1
10	2	2	1	1	1
11	3	3	1	1	1
12	3	3	1	1	1
13	3	3	1	1	1
14	5	5	1	1	1

*Compre. (Comprehensiveness of Information).

^#^Accu. (Accuracy of Information).

**Table 8:** Relative and standardized scores of answers provided by experts and ChatGPT.

Pain control					
	Benchmark				
	Compre.	Accu.	Compre.	Accu.	Final
1	5	5	1	1	1
2	3	3	1	1	1
3	3	3	1	1	1
4	2	2	1	1	1
5	2	2	1	1	1
6	3	3	1	1	1
7	3	3	1	1	1
8	3	3	1	1	1
9	2	2	1	1	1
10	3	3	1	1	1
11	3	3	1	1	1
12	2	2	1	1	1
13	3	3	1	1	1
14	2	2	1	1	1
15	3	3	1	1	1
16	3	3	1	1	1
17	3	3	1	1	1
18	2	2	1	1	1
19	2	2	1	1	1
20	3	3	1	1	1
21	3	3	1	1	1

	ChatGPT				
	Compre.	Accu.	Compre.	Accu.	Final
1	5	5	1	1	1
2	3	3	1	1	1
3	3	3	1	1	1
4	2	2	1	1	1
5	2	2	1	1	1
6	3	3	1	1	1
7	3	3	1	1	1
8	1	3	0.33333333	1	0.66666667
9	2	2	1	1	1
10	1	3	0.33333333	1	0.66666667
11	2	3	0.66666667	1	0.83333333
12	2	2	1	1	1
13	3	3	1	1	1
14	2	2	1	1	1
15	3	3	1	1	1
16	2	3	0.66666667	1	0.83333333
17	3	3	1	1	1
18	2	2	1	1	1
19	2	2	1	1	1
20	3	3	1	1	1
21	3	3	1	1	1

	ChatGPT Rep2				
	Compre.	Accu.	Compre.	Accu.	Final
1	5	5	1	1	1
2	3	3	1	1	1
3	3	3	1	1	1
4	2	2	1	1	1
5	2	2	1	1	1
6	3	3	1	1	1
7	3	3	1	1	1
8	1	3	0.33333333	1	0.66666667
9	2	2	1	1	1
10	1	3	0.33333333	1	0.66666667
11	2	3	0.66666667	1	0.83333333
12	2	2	1	1	1
13	3	3	1	1	1
14	2	2	1	1	1
15	3	3	1	1	1
16	3	3	1	1	1
17	3	3	1	1	1
18	2	2	1	1	1
19	2	2	1	1	1
20	3	3	1	1	1
21	3	3	1	1	1

*Compre. (Comprehensiveness of Information).

^#^Accu. (Accuracy of Information).

### An overview of ChatGPT’s mean performance

**Figure 2:**
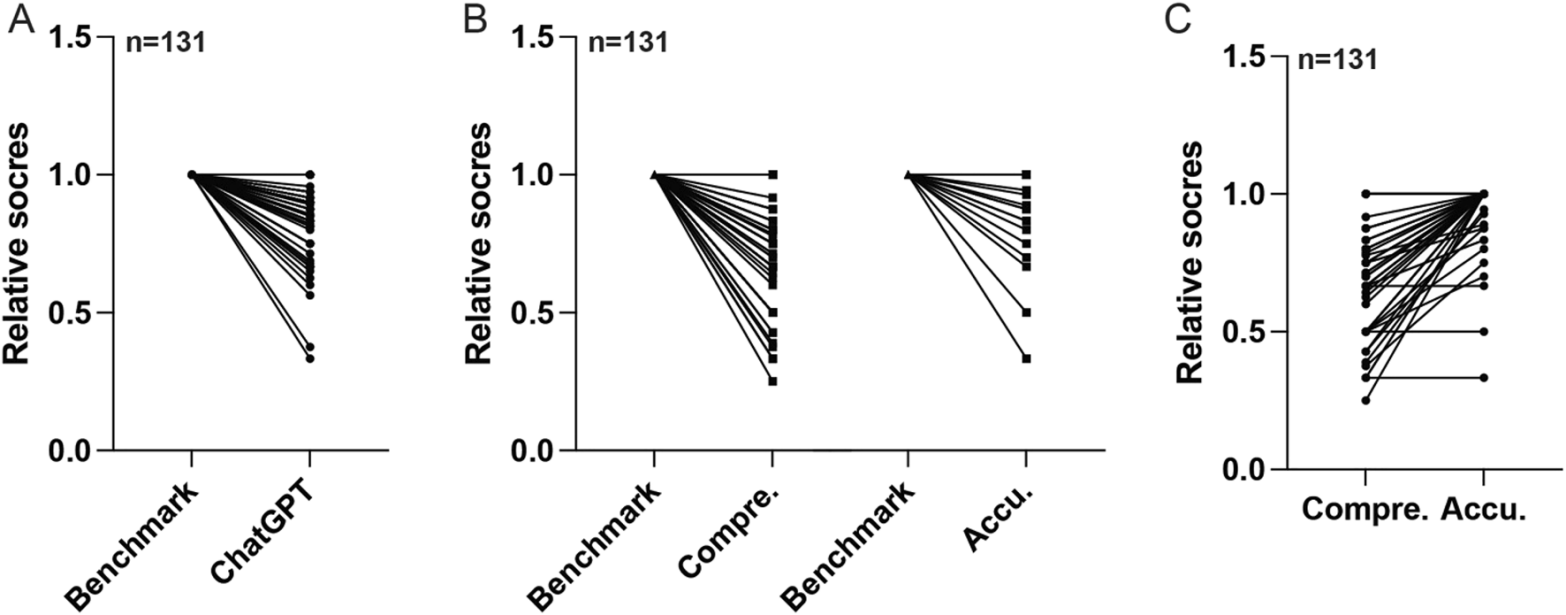
The General Performance of ChatGPT. (**A**) Overall scores combining accuracy and comprehensiveness for each query answered by ChatGPT, compared to the benchmarks in medical literature standards. (**B**) Comprehensiveness and accuracy scores for each query, compared to the benchmarks in medical literature. (**C**) Comparative analysis of accuracy and comprehensiveness scores for each query answered by ChatGPT.

To elucidate the general efficiency of ChatGPT's responses, we compared the final scores of ChatGPT to the benchmark scores from the mentioned book. Despite certain studies vindicating the proficiency of ChatGPT within the medical discipline^16,17^, our investigative endeavor herein revealed that the mean scores of ChatGPT’s responses were markedly inferior to that of the benchmark (n = 131, p < 0.0001), with the mean score of 0.91 within the AI group (Fig. 2A). This observation may suggest that ChatGPT has yet to attain an expert level of competence within the field of colorectal cancer (CRC).

Subsequently, we examined the average performance of ChatGPT in comprehensiveness and accuracy, separately. Upon further computation, the mean score of comprehensiveness and accuracy stood at 0.85 and 0.97, proving significantly inferior to the benchmark (n = 131, p < 0.0001; n = 131, p = 0.0001) (Fig. 2B). This also showed that ChatGPT had a higher ability to provide accurate information than to provide comprehensive information (n = 131, p < 0.0001) (Fig. 2C). Collectively, these observations infer that ChatGPT’s responses within the realm of CRC have not fully reached the proficiency of medical professionals.

### ChatGPT’s noteworthy performance in specific domains

**Figure 3:**
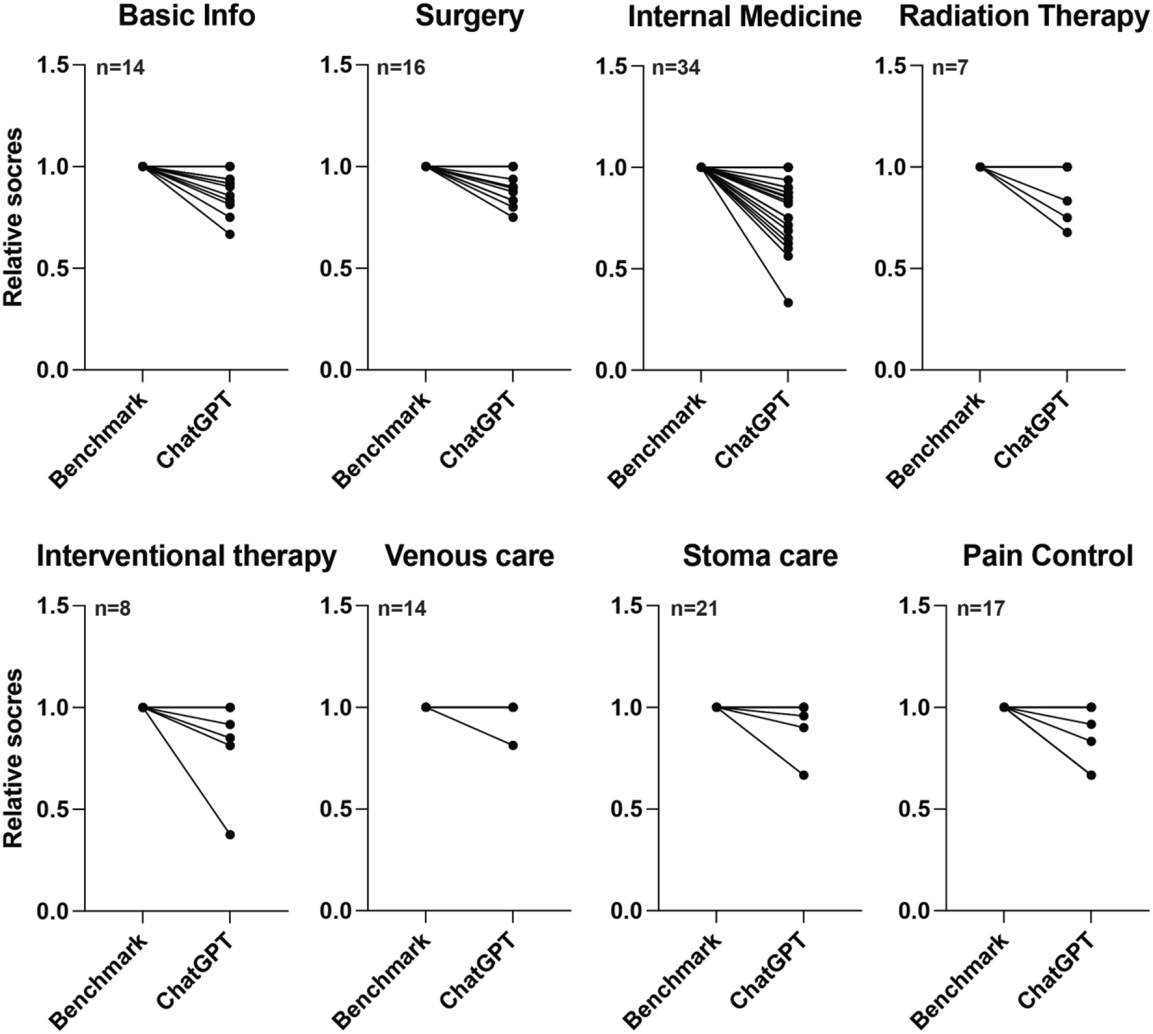
ChatGPT's Noteworthy Performance in Specific Domains. The overall scores are compared to the benchmarks in medical literature across various medical specialties or topics.

However, on a sectional consideration, we discerned that in many instances, ChatGPT's responses satisfactorily met the anticipated standards. For example, in the field of radiation therapy (n = 7, p = 0.25), interventional therapy (n = 8, p = 0.125), stoma care (n = 21, p = 0.25), venous care (n = 14, p > 0.99), and pain control (n = 17, p = 0.125), ChatGPT performed exceptionally well compared to the benchmark scores (Fig. 3A), with final scores almost rivaling the referential benchmark. This suggests that ChatGPT possessed high expertise and answering ability in these specific domains. Particularly in stoma and venous care, the mean scores were both > 0.97, evidencing ChatGPT’s reliability in answering questions in these two fields. In contrast, ChatGPT’s performance was significantly inferior in the fields of basic information (n = 14, p = 0.002), surgery (n = 16, p = 0.0078), and internal medicine (n = 34, p < 0.0001) when compared to the benchmark scores (Fig. 3A). Such deficits might be attributable to the complexity and specificity of these fields, or the necessity for more profound expertise and comprehensive explanations within these disciplines. Additionally, the rapidly evolving nature of treatment strategies in these fields poses a huge challenge for pre-trained models in acquiring the most recent and correct knowledge.

## Discussion

Our study explores the burgeoning domain of AI-assisted healthcare communication, using ChatGPT as a case study in the context of patient education for colorectal cancer (CRC). The observations from this investigation shed light on both the potential and the limitations of ChatGPT, offering a foundation upon which we can build future developments and improvements.

Among the findings, we noted the consistent reproducibility in the responses of ChatGPT, reflecting an aspect of reliability that is crucial for systems intended for the delivery of medical information. In further analysis, ChatGPT demonstrated a promising degree of accuracy in its responses, although it fell short in comprehensiveness compared to our benchmark. This shortfall could be attributed to the broad and nonspecific training data used to develop the AI model, which may lack certain in-depth specifics or nuanced aspects, particularly in specialized fields such as medicine. These results highlight an area for potential improvement, which could involve integrating more specialized and updated training data into AI models like ChatGPT to improve the breadth and depth of their responses.

Interestingly, our study revealed that ChatGPT’s overall performance, as measured by our scoring system, its performance in certain domains approaches the human benchmark. In particular, it performed exceptionally well in the fields of radiation therapy, interventional therapy, stoma care, venous care, and pain control. This finding is particularly encouraging as it suggests that deep learning-based AI systems like ChatGPT, when supplied with targeted, refined, and regularly updated data, could serve as potent tools in these specialized fields. However, the limited number of questions examined in these domains may have inadvertently skewed the results, and hence, these findings should be interpreted with caution. Future studies with larger sample sizes across these fields would be beneficial in ascertaining the real-world efficacy of ChatGPT.

Despite the promising results above, it was evident that ChatGPT was not yet on par with expert knowledge, in the field of basic information, surgical management, and internal medicine. Such an underperformance could potentially impede the deployment of AI models like ChatGPT in clinical practice, especially when considering the complexity, specificity, and the rapidly evolving nature of these fields.

Though we tried our best, there were still several drawbacks in our study. The first one was limited scope of questions. Our study here used public health book of CRC as its data source, which inherently limits the types of questions that are addressed. The questions included in the book were meticulously selected by the authors to cover a broad range of topics related to CRC, which might not encapsulate all the queries that patients and their families have in real life. The reference book only included highly clinical-related sections, while in real-life healthcare scenarios, patients often have sequential and personalized questions concerning their lifestyle, diet, side effects of medications, financial issues, or even emotional well-being. They might ask the follow-up questions based on the previous laboratory/imaging test, or questions requiring personal medical history. For instance, a patient could ask, “What’s my next treatment protocol?” or “Are my blood test results normal?” Answering such questions requires an understanding of the patient's previous treatments and their unique health situation, which ChatGPT currently lacks. This limits the range of questions AI can accurately answer. Future research should explore the ways to enable the sharing of personal health information with AI models to provide personalized and relevant information to patients. However, this raises privacy and ethical issues, and it is unclear if companies behind these AI systems will misuse patients’ private data. At least in this aspect, AI is far from being able to replace doctors.

Further, for the evaluation of AI applications in patient health, more studies need to be conducted using questions gathered from various sources, like hospitals, clinics, online forums, patient interviews, and social media platforms. Such an approach would provide a more realistic assessment of AI’s potential in healthcare. While our purpose was to investigate the implementation of AI in the realm of public health, the limitations posed by focusing solely on CRC disease may have compromised the rigor of our study. Each disease possesses distinct characteristics, complications, treatment methods, and patient considerations, which were not adequately addressed. For instance, a patient with a chronic disease like diabetes must have different concerns and information needs compared to a patient with an acute condition like pneumonia or a life-threatening disease like cancer. Therefore, to assess the true capabilities of AI in healthcare, future research should consider a more diverse range of medical conditions. This approach would not only provide a more comprehensive understanding of the potential of AI in healthcare but also help identify specific areas where AI could be most beneficial.

Another limitation of this study is using the book's answers as the benchmark for scoring ChatGPT’s responses. While it provides a clear standard for evaluation, it has limitations. Firstly, the answers in the book were provided by experts in the field of CRC and were presumably accurate and comprehensive. However, medicine evolves constantly, new research is leading to new treatments and best practices. While the book used in this study might be up to date at the time of its publication, it could become outdated over time and might not cover all possible answers to a given question. If ChatGPT generates an answer based on more recent research that contradicts the book, it would be penalized unfairly. Similarly, if the model fails to incorporate the latest practices into responses, its effectiveness could be overestimated. Therefore, by using the book's answers as the only benchmark, the study might not fully capture the range of correct and useful responses that ChatGPT provided. Also, evaluators were not blinded, and they were aware of the source they were scoring. If evaluators have any preconceived prejudices towards AI, they may have scored AI responses more harshly. Even if evaluators strive to be more objective, unconscious bias could still be introduced. On the other hand, knowing the book’s answers were provided by experts, they might have been more lenient in scoring ambiguities. This could skew the study's results to some extent and give an inaccurate picture of ChatGPT’s capabilities. To mitigate this risk, future studies should consider blinding the evaluators to the sources of responses. While scoring focused on factual accuracy and completeness, it missed the emotional aspect of patients. The tone and empathy in the responses significantly impact the patient's satisfaction and comfort level. The current study does not consider these factors, leading to an incomplete understanding of ChatGPT’s potential in patient education and support, as one proverb goes: *to cure sometimes, to relieve often, to comfort always*. More importantly, our study solely employed ChatGPT, and it did not include a comparative analysis with other AI tools. Numerous AI models have surfaced in recent years, professing advanced reasoning capabilities and the proficiency to provide answers with a high degree of certainty, like Google’s Bard and Anthropic’s Claude. A complete assessment would involve comparing the performance of several AI models across a range of tasks in healthcare. This approach could help pinpoint the optimal model for each task and guide the development of future AI models.

Inherent limitations also exist within the AI models themselves. For instance, ChatGPT and similar models, being static with a training cut-off, are unable to assimilate new data post-training at present. This absence of a continuous learning mechanism may restrict the usefulness of the AI model in rapidly progressing fields like healthcare, where new knowledge and practices are constantly emerging. Thus, these models might generate a seemingly correct but contextually wrong response, which might lead to confusion or misinterpretation. A promising enhancement could involve integrating continuous learning mechanisms into future AI models, allowing them to refresh their knowledge base and evolve over time. Additionally, our study highlights a critical aspect of these models' interaction dynamics: a tendency towards “confirmation bias”. ChatGPT, when corrected by users, often modifies its initial responses, even if they were factually accurate. This responsiveness could lead to manipulation of responses, where the model replicates user biases rather than maintaining objective accuracy. This is particularly problematic in healthcare domains, where the precision of information is crucial. Future iterations of AI models should be designed to discern between valid corrections and subjective feedback, maintaining the integrity of their knowledge in the face of user influence. Further, we must acknowledge the risks involved in relying on AI for medical information dissemination. Even as AI systems like ChatGPT achieve high levels of performance, they are prone to “hallucinations”—instances where they generate incorrect or misleading information. Moreover, these systems lack the empathy and contextual adaptability of a human doctor, which is crucial in understanding and addressing patient-specific concerns. Such limitations highlight the need for careful and supervised integration of AI in healthcare settings, ensuring that AI-assisted information is always verified and contextualized by medical professionals.

Indeed, we notice that the current version of ChatGPT now supports image input. This opens up prospects for AI systems to aid in the analysis of diagnostic images and patient records, as demonstrated by the interpretable approaches for colorectal whole-slide images^19^ and the clinically validated interpretable machine learning-based prototype for CRC^20^. However, ensuring accurate recognition and appropriate assessment remains critical, especially when dealing with intricate medical data. The integration of AI systems like ChatGPT with such advanced diagnostic tools could significantly enhance patient understanding and support, yet it necessitates rigorous validation to ensure reliability and accuracy.

## Conclusions

Our study provides significant insights into the utility of the ChatGPT AI model in the field of colorectal cancer (CRC) education. Despite some areas needing improvement, ChatGPT's overall performance affirms its potential as a reliable and easily accessible source of health information. And future research should explore further refining these AI models, ensuring they stay updated with the latest medical knowledge, and examine their application across a wider array of medical disciplines. But at the same time, AI should be used as a supplementary tool to, not a replacement for, the expertise of healthcare professionals at present.

## Methods

### Data source

All questions were obtained from the book titled “Colorectal Cancer: Your Questions Answered”, which is a collaborative effort by a team of experts in the field of CRC in Nanjing, China. It comprises a collection of carefully curated 152 questions gathered from healthcare professionals, patients, and their families. These questions cover various aspects of CRC, including basic information (14), surgical management (16), internal medicine treatments (34), radiation therapy (7), interventional treatments (8), ostomy care (17), deep vein care (14), pain control (21). The psychology and Chinese traditional medicine (TCM) sections were not included in this research. Therefore, this study ultimately incorporated a total of 131 valid questions. Each question in this book has been answered by experts actively engaged in clinical practice within their respective domains, ensuring the reliability of these responses. Furthermore, we have obtained authorization from the copyright holder of the book.

### Data preprocess

Due to a significant disparity in the volume of training data between Chinese and English text. In the statement of OpenAI, the ChatGPT model trained on GPT-3.5 has been exposed to billions of English texts, including all sorts of publicly available English content. However, the amount of Chinese text utilized for training the GPT-3.5 model is relatively limited. In order to maximize the effectiveness of ChatGPT, All questions in this book have been translated into English manually by senior physicians specializing in both internal medicine and surgery from our hospital. To facilitate subsequent scientific analysis, each answer has been meticulously reorganized, point by point, to form a grading criterion in a list format. Each item on the list serves as a scoring point, and the total points obtained were served as benchmark score.

### ChatGPT bot and prompt

The version GPT-3.5 was asked in our research in January 2023. Each individual question was entered separately and independently, employing the “New Chat” function. In order to examine the coherence and replicability of the responses produced by the ChatGPT model, every question was input into ChatGPT not only once but two times, using the “regenerate response” feature.

### Scoring

The process of review and scoring was meticulously carried out by clinical physician specialized in each field. And here we would like to extend our deepest appreciation for their invaluable assistance. The evaluators have access to all necessary information, including the origin questions, the answers provided in the selected book, and the responses generated by ChatGPT. Each of the two responses from ChatGPT for a single question was independently scored. The evaluators, unaware of which response was first or second, assessed them based on the predefined criteria of comprehensiveness and accuracy. The score for each question in our study was then determined by computing the mean scores of the two replicates. During the evaluation process, the evaluators assign scores in accordance with the grading template, allocating points according to pre-established criteria. In order to minimize subjective judgment and fully test the efficiency of ChatGPT in scoring process, we requested two scoring sections for each response of ChatGPT. (a) Comprehensiveness of Information (Conpre.): This assesses whether ChatGPT’s responses encompass all answers provided in the book. The benchmark score for each question is determined by the number of relevant aspects covered in the book’s response. If all the answers are given, the score of ChatGPT in this section will be the same as the benchmark score, and for each relevant aspect missing in ChatGPT’s response, one point is deducted from the benchmark score. (b) Accuracy of Information (Accu.): This focuses on the correctness of ChatGPT’s responses. If ChatGPT's response contains incorrect information, it should be penalized in this section. Each wrong element (including inaccurate, misleading, or irrelevant information) leads to a one-point deduction from the benchmark score, which represents the total number of relevant aspects in the book’s response, until reaching zero. The score of Conpre. or Accu. is calculated as the average of the two physicians’ assessments. And the final scores are determined by the mean of scores for comprehensiveness and accuracy. In the event of significant discrepancies between the scores of the two initial evaluators (a difference of 2 or more), a third highly experienced practitioner is consulted to provide an independent assessment. In such case, the score is not solely determined by the third evaluator. Instead, their assessment is integrated with the initial evaluations to reach a relative consensus. Dr. Sun and Dr. Yang have participated in this part of the work, and we also express thanks to them.

### Interpretation and standardization process

Due to the inherent variation in the benchmark scores of each individual question, we undertake a process of standardization for both accuracy and comprehensiveness scores. For instance, if the benchmark score for question A is set at 10, and ChatGPT achieves a comprehensiveness score of 8, we calculate the ratio of ChatGPT’s score to the benchmark score, which in this case would be 8/10, resulting in a comprehensiveness ratio of 0.8. Similarly, if ChatGPT’s accuracy score for the same question is 9, the accuracy ratio would be 9/10, equating to 0.9. To obtain a standardized overall score for question A, we then sum these two ratios—the comprehensiveness ratio (0.8) and the accuracy ratio (0.9)—which gives us 1.7. This sum is then divided by 2, resulting in a standardized score ratio of 0.85 for question A.

### Statistical analysis

We calculated the average ratios and standard deviations (SD) for the accuracy, comprehensiveness, and final scores to ascertain its performance and the variability exhibited by ChatGPT. We also investigated the reproducibility of ChatGPT's responses in the two replicates by comparing their similarity. To rigorously determine whether ChatGPT's performance either matched or was inferior to that of professional medical personnel, we utilized the Wilcoxon signed-rank test to analyze the overall score. The significance threshold was denoted as p < 0.05. All our analyses were performed on Prism 9.

### Ethics committee approval

All methods were performed in accordance with the relevant guidelines and regulations. The study followed the principles contained in the Declaration of Helsinki and approved by the Ethics Committee of Jiangsu Provincial People’s Hospital. Informed consent was obtained from all participants.

### Supplementary Information

Supplementary Information.

## Data Availability

All data generated or analyzed during the study are included in this published article.

## Abbreviations

CRC: Colorectal cancer
AI: Artificial intelligence
