# Supplementary material for: Evaluating AI in medicine: a comparative analysis of expert and ChatGPT responses to colorectal cancer questions
Source: Sci Rep. 2024 Feb 3;14:2840. doi: 10.1038/s41598-024-52853-3 (PMC10838275; doi:10.1038/s41598-024-52853-3)
Supplement: Supplementary file 1 — Supplementary Information. [file 41598_2024_52853_MOESM1_ESM.docx]

**Answers by ChatGPT (Rep2 represents repeated response)**

**The Basic Information**

**1. Which individuals are more susceptible to colorectal cancer?**

(1) Individuals above the age of 50: Colorectal cancer primarily affects older individuals, with the majority of cases diagnosed in people over the age of 50. As individuals age, the risk of developing colorectal cancer increases.

(2) Individuals with a family history of colorectal cancer: People who have close relatives, such as parents, siblings, or children, who have had colorectal cancer are at a higher risk themselves. The risk is further increased if multiple family members have been affected or if the diagnosis occurred at a younger age.

(3) Individuals with certain genetic conditions: Certain inherited genetic conditions, such as Lynch syndrome (hereditary nonpolyposis colorectal cancer) and familial adenomatous polyposis (FAP), significantly increase the risk of colorectal cancer. These conditions are relatively rare but can substantially increase the susceptibility to the disease.

(4) Individuals with a personal history of colorectal polyps or inflammatory bowel disease (IBD): People who have had certain types of colorectal polyps, such as adenomas, have an increased risk of developing colorectal cancer. Additionally, individuals with a history of inflammatory bowel diseases like ulcerative colitis or Crohn's disease have a higher risk compared to the general population.

(5) Individuals with a sedentary lifestyle and poor dietary habits: Lack of physical activity, a diet high in red and processed meats, low fiber intake, and excessive alcohol consumption can contribute to an increased risk of colorectal cancer.

(6) Individuals with obesity: Obesity is associated with an elevated risk of colorectal cancer. Maintaining a healthy body weight through regular physical activity and a balanced diet can help reduce this risk.

(7) Individuals with certain racial and ethnic backgrounds: Certain racial and ethnic groups, such as African Americans and Ashkenazi Jews, have higher rates of colorectal cancer compared to other populations.

It's important to note that while these factors increase the susceptibility to colorectal cancer, anyone can develop the disease. Regular screenings and adopting a healthy lifestyle are crucial for early detection and prevention.

Rep2:

(1) Older individuals: Colorectal cancer is more commonly diagnosed in individuals above the age of 50. As people age, the risk of developing colorectal cancer increases. Regular screening for colorectal cancer is recommended for adults starting at age 50, or earlier for those with certain risk factors.

(2) Individuals with a family history: People who have close relatives (such as parents, siblings, or children) who have had colorectal cancer or certain types of polyps are at a higher risk. The risk is even greater if the family member was diagnosed at a younger age or if multiple family members are affected. Genetic factors can play a role in the development of colorectal cancer.

(3) Individuals with certain hereditary conditions: Some inherited gene mutations can significantly increase the risk of colorectal cancer. Examples include familial adenomatous polyposis (FAP) and Lynch syndrome (also known as hereditary non-polyposis colorectal cancer or HNPCC). These conditions are relatively rare but are associated with a much higher likelihood of developing colorectal cancer.

(4) Individuals with a personal history of colorectal cancer or polyps: If an individual has had colorectal cancer in the past, their risk of developing a new colorectal cancer is higher. Similarly, individuals who have previously had certain types of polyps, such as adenomatous polyps or serrated polyps, are at an increased risk.

(5) Individuals with inflammatory bowel disease (IBD): Chronic inflammatory conditions of the colon and rectum, such as ulcerative colitis and Crohn's disease, can increase the risk of developing colorectal cancer over time. Regular surveillance and monitoring are typically recommended for individuals with IBD.

(6) Individuals with certain lifestyle factors: Certain lifestyle choices and behaviors can contribute to an increased risk of colorectal cancer. These include a diet high in red or processed meats, low fiber intake, sedentary lifestyle, obesity, heavy alcohol consumption, and smoking.

It's important to note that while these factors can increase the likelihood of developing colorectal cancer, they do not guarantee the development of the disease. Regular screening, early detection, and lifestyle modifications can help in reducing the risk and improving outcomes. If you have concerns about colorectal cancer or your individual risk factors, it is recommended to consult with a healthcare professional.

**2.** **How can one undertake measures for the prevention of colorectal cancer?**

Here are several measures that can be undertaken for the prevention of colorectal cancer:

(1) Regular screening: Screenings such as colonoscopies, sigmoidoscopies, and stool tests like the fecal immunochemical test (FIT) or high-sensitivity guaiac-based fecal occult blood test (FOBT) can help detect precancerous polyps or early-stage colorectal cancer. Regular screening is recommended starting at age 50 for most individuals, or earlier for those with certain risk factors or a family history of colorectal cancer.

(2) Adopting a healthy diet: A diet rich in fruits, vegetables, whole grains, and fiber, while low in red and processed meats, can help reduce the risk of colorectal cancer. Consuming a variety of nutrient-dense foods and limiting the intake of sugary drinks and foods high in saturated fats is beneficial.

(3) Maintaining a healthy weight: Obesity has been linked to an increased risk of colorectal cancer. Engaging in regular physical activity and maintaining a healthy weight through a balanced diet and exercise can help lower the risk.

(4) Limiting alcohol consumption: Heavy alcohol consumption has been associated with an increased risk of colorectal cancer. It is advisable to limit alcohol intake or avoid it altogether. Moderate alcohol consumption is defined as up to one drink per day for women and up to two drinks per day for men.

(5) Quitting smoking: Smoking has been linked to various types of cancer, including colorectal cancer. Quitting smoking and avoiding exposure to secondhand smoke can significantly reduce the risk of developing colorectal cancer and improve overall health.

(6) Managing chronic inflammatory conditions: If you have been diagnosed with inflammatory bowel disease (IBD), it is essential to work closely with your healthcare provider to manage and control the condition. Regular monitoring and treatment can help reduce the risk of colorectal cancer associated with IBD.

(7) Knowing and understanding family history: If you have a family history of colorectal cancer or certain hereditary conditions, it is important to discuss this with your healthcare provider. They may recommend earlier or more frequent screenings or genetic testing to assess your individual risk.

(8) Leading a healthy lifestyle: Engaging in regular physical activity, managing stress, and getting enough sleep are important aspects of maintaining overall health. These lifestyle factors can also contribute to reducing the risk of colorectal cancer.

Remember, while these measures can help lower the risk of developing colorectal cancer, they do not guarantee complete prevention. It's crucial to discuss your personal risk factors and appropriate preventive strategies with a healthcare professional.

Rep2:

Certainly! Here are several measures that can be undertaken for the prevention of colorectal cancer:

(1) Regular screening: Regular screening is crucial for detecting colorectal cancer at an early stage or identifying precancerous polyps. The most common screening method is a colonoscopy, which allows for the detection and removal of polyps before they become cancerous. Other screening options include sigmoidoscopy, fecal occult blood test (FOBT), and fecal immunochemical test (FIT). The choice of screening method and frequency may vary based on individual risk factors and guidelines provided by healthcare professionals.

(2) Maintain a healthy diet: A diet rich in fruits, vegetables, whole grains, and fiber has been associated with a lower risk of colorectal cancer. On the other hand, a high consumption of red and processed meats has been linked to an increased risk. Limiting the intake of processed and red meats while incorporating more plant-based foods can be beneficial for colorectal cancer prevention.

(3) Regular physical activity: Engaging in regular physical activity and maintaining a healthy weight can help reduce the risk of colorectal cancer. Aim for at least 150 minutes of moderate-intensity exercise or 75 minutes of vigorous exercise per week. Simple activities like brisk walking, cycling, or swimming can make a positive impact.

(4) Limit alcohol consumption: Excessive alcohol consumption has been linked to an increased risk of colorectal cancer. It is recommended to limit alcohol intake or avoid it altogether. For men, moderate alcohol consumption is generally defined as up to two drinks per day, while for women, it is up to one drink per day.

(5) Don't smoke: Smoking has been associated with an increased risk of several types of cancer, including colorectal cancer. Quitting smoking or avoiding tobacco use altogether can contribute to overall health and lower the risk of various diseases, including colorectal cancer.

(6) Be aware of family history and genetic factors: If you have a family history of colorectal cancer or certain genetic conditions associated with an increased risk, it is important to inform your healthcare provider. They can assess your individual risk and recommend appropriate screening and preventive measures.

(7) Manage chronic conditions: If you have conditions such as inflammatory bowel disease (IBD), it is crucial to work closely with your healthcare provider to manage and monitor the condition effectively. Regular check-ups and appropriate treatment can help reduce the risk of colorectal cancer in individuals with chronic inflammatory conditions.

Remember, these preventive measures can help reduce the risk of colorectal cancer, but they do not guarantee complete prevention. Regular medical check-ups, awareness of symptoms, and prompt medical attention are important for early detection and successful treatment. Consult with a healthcare professional for personalized advice and guidance based on your individual circumstances.

**3. Is colorectal cancer hereditary in nature?**

(1) Yes, colorectal cancer can have a hereditary component. Some individuals may have a higher risk of developing colorectal cancer due to genetic factors. There are specific inherited genetic conditions, such as Lynch syndrome (hereditary non-polyposis colorectal cancer) and familial adenomatous polyposis (FAP), that can increase the likelihood of developing colorectal cancer.

(2) In Lynch syndrome, mutations in genes like MLH1, MSH2, MSH6, and PMS2 can increase the risk of colorectal cancer and other types of cancers. Individuals with Lynch syndrome have a higher chance of developing colorectal cancer at a younger age.

(3) Familial adenomatous polyposis (FAP) is another inherited condition that increases the risk of colorectal cancer. It is caused by mutations in the APC gene, leading to the development of numerous polyps in the colon and rectum, which can progress to cancer if not treated.

(4) It's important to note that while hereditary factors can contribute to the risk of colorectal cancer, the majority of cases (over 75%) occur in individuals without a known inherited predisposition. Lifestyle factors such as diet, physical activity, smoking, and alcohol consumption also play a significant role in the development of colorectal cancer. Regular screening and early detection are crucial for both individuals with a family history of colorectal cancer and those without a known hereditary risk.

Rep2:

(1) Yes, colorectal cancer can have a hereditary component. Certain genetic mutations can increase the risk of developing colorectal cancer. The most well-known hereditary condition associated with colorectal cancer is Lynch syndrome (hereditary non-polyposis colorectal cancer or HNPCC). Individuals with Lynch syndrome have a higher risk of developing colorectal cancer and other cancers, such as endometrial, ovarian, and stomach cancer.

(2) Another hereditary condition associated with colorectal cancer is familial adenomatous polyposis (FAP). FAP is characterized by the development of numerous polyps in the colon and rectum, which greatly increases the risk of developing colorectal cancer.

(3) There are also other, less common hereditary conditions such as MUTYH-associated polyposis (MAP) and Peutz-Jeghers syndrome that can contribute to an increased risk of colorectal cancer.

It's important to note that while these hereditary conditions increase the risk of developing colorectal cancer, they do not guarantee the development of the disease. Environmental factors and lifestyle choices also play a significant role in the development of colorectal cancer. Regular screenings and early detection are crucial for individuals with a family history of colorectal cancer or known hereditary conditions.

**4. Is colorectal cancer contagious in any way?**

(1) No, colorectal cancer is not contagious. It cannot be transmitted from one person to another through direct contact, such as touching or being in close proximity to someone with colorectal cancer. Colorectal cancer develops when there are certain changes or mutations in the DNA of cells in the colon or rectum. These mutations occur within the cells of the affected individual and are not caused by infectious agents like bacteria or viruses.

(2) Colorectal cancer is primarily caused by a combination of genetic and lifestyle factors, such as family history, age, certain inherited gene mutations, diet, obesity, smoking, and sedentary lifestyle. These factors contribute to the development of colorectal cancer over time. It is important to note that while some cancers are caused by infectious agents (e.g., human papillomavirus and cervical cancer), colorectal cancer does not fall into this category.

(3) The risk factors for colorectal cancer are largely related to individual behaviors and genetics. Age is one of the most significant risk factors, with the majority of cases occurring in individuals over the age of 50. Other risk factors include a family history of colorectal cancer or certain genetic conditions, such as Lynch syndrome or familial adenomatous polyposis (FAP). Lifestyle choices, such as a diet high in red and processed meats, low fiber intake, lack of physical activity, obesity, and smoking, can also increase the risk of developing colorectal cancer.

It's important to consult with a healthcare professional for personalized advice and screenings to assess your risk and detect colorectal cancer at an early stage.

Rep2:

(1) No, colorectal cancer is not contagious. It cannot be transmitted from one person to another through any form of contact or exposure. Colorectal cancer develops when normal cells in the colon or rectum undergo genetic mutations that lead to uncontrolled growth. These mutations occur within the cells of the individual affected by the cancer and are not caused by an infectious agent.

(2) Colorectal cancer is primarily caused by a combination of genetic and environmental factors, such as family history, age, diet, lifestyle choices, and certain medical conditions. It is not related to any infectious or contagious agents. Therefore, there is no risk of transmitting colorectal cancer from one person to another.

(3) The development of colorectal cancer is mainly influenced by individual factors and not by external transmission. It is important to note that while certain infections, such as human papillomavirus (HPV) and hepatitis B and C viruses, can increase the risk of developing certain types of cancer, they are not associated with colorectal cancer transmission.

(4) In summary, colorectal cancer is not contagious or transmissible between individuals. It arises from genetic mutations and various risk factors that affect the individual's own cells and body. Regular screenings, maintaining a healthy lifestyle, and seeking medical advice for any concerning symptoms or family history can help detect and manage colorectal cancer effectively.

**5. What are the early symptoms of colorectal cancer?**

(1) Change in bowel habits: One of the early symptoms of colorectal cancer is a change in bowel habits. This may include persistent diarrhea or constipation, or a noticeable change in the size, shape, or consistency of your stool.

(2) Blood in the stool: Another early symptom is the presence of blood in the stool. This can manifest as bright red blood or dark, tarry stools. Blood in the stool can be a sign of various conditions, including colorectal cancer.

(3) Abdominal discomfort: Colorectal cancer can cause persistent abdominal discomfort, such as cramps, pain, or a feeling of fullness. This discomfort may be accompanied by bloating or a sense of urgency to have a bowel movement.

(4) Unexplained weight loss: In some cases, colorectal cancer can lead to unexplained weight loss. If you're losing weight without any intentional changes in your diet or exercise routine, it could be a symptom of underlying health issues, including cancer.

(5) Fatigue and weakness: Feeling constantly tired or experiencing unexplained weakness can be early signs of colorectal cancer. These symptoms may be related to the body's response to the cancer or due to the loss of blood, which can lead to anemia.

(6) Iron deficiency anemia: Colorectal cancer can cause chronic bleeding in the digestive tract, leading to iron deficiency anemia. This can result in fatigue, weakness, shortness of breath, and pale skin.

It's important to note that these symptoms can be indicative of various other conditions as well, and the presence of these symptoms does not necessarily mean you have colorectal cancer. However, if you experience any of these symptoms persistently or if you have concerns, it's crucial to consult with a healthcare professional for a proper evaluation and diagnosis.

Rep2:

(1) Change in bowel habits: One of the early symptoms of colorectal cancer is a change in bowel habits. This can include persistent diarrhea or constipation, a feeling of incomplete bowel movement, or a change in the consistency of stool.

(2) Rectal bleeding: Another early symptom is rectal bleeding, which may present as blood in the stool or on the toilet paper after a bowel movement. It's important to note that not all cases of rectal bleeding are due to colorectal cancer, but it should be evaluated by a healthcare professional.

(3) Abdominal pain and discomfort: Some individuals with colorectal cancer may experience abdominal pain or discomfort, which can be persistent and unrelated to bowel movements. This pain may vary in intensity and can occur in different areas of the abdomen.

(4) Unexplained weight loss: Unintentional weight loss can occur in the early stages of colorectal cancer. If there is a noticeable and unexplained decrease in weight without changes in diet or physical activity, it is important to seek medical attention.

(5) Fatigue and weakness: Colorectal cancer can also cause fatigue and weakness, even with normal daily activities. This can be attributed to factors such as anemia or the body's response to the cancer.

(6) Iron deficiency anemia: In some cases, colorectal cancer may lead to iron deficiency anemia, which can cause symptoms such as fatigue, weakness, pale skin, and shortness of breath.

It's important to remember that these symptoms can also be caused by conditions other than colorectal cancer. However, if you experience any persistent or concerning symptoms, it is advisable to consult a healthcare professional for further evaluation and appropriate testing. Early detection and treatment of colorectal cancer can significantly improve outcomes.

**6, Is early screening possible for colorectal cancer? What are the recommended screening tests for colorectal cancer?**

(1) Yes, early screening is possible for colorectal cancer. Regular screening can help detect colorectal cancer at an early stage when it is more treatable and potentially curable. Screening can also identify precancerous polyps, which can be removed before they develop into cancer.

(2) The recommended screening tests for colorectal cancer include:

- Colonoscopy: This is considered the gold standard for colorectal cancer screening. It involves a thorough examination of the entire colon using a flexible tube with a camera. It allows for the detection and removal of polyps during the procedure.

- Fecal Immunochemical Test (FIT): This is a stool-based test that detects hidden blood in the stool, which may be a sign of colorectal cancer or polyps. It is a non-invasive and cost-effective screening option.

- Fecal Occult Blood Test (FOBT): This is another stool-based test that checks for blood in the stool. It is similar to FIT but uses different methods to detect blood.

- Flexible Sigmoidoscopy: This procedure examines the lower part of the colon and rectum using a flexible tube with a camera. It can detect polyps and early-stage cancers in the lower colon.

- CT Colonography (Virtual Colonoscopy): This is a radiographic examination that produces detailed images of the colon. It requires a bowel preparation similar to colonoscopy and can detect polyps and cancers.

(3) Other screening tests, though less commonly used, include the stool DNA test (sDNA), which detects genetic material from cancer cells or polyps in the stool, and the capsule endoscopy, where a small camera is swallowed and captures images as it passes through the digestive system.

It's important to note that the choice of screening test depends on various factors such as age, personal medical history, family history, and risk factors. It's best to consult with a healthcare professional to determine the most suitable screening test for an individual.

Early screening for colorectal cancer is crucial as it can significantly increase the chances of detecting the disease at an early stage when treatment outcomes are generally more favorable. Regular screening can save lives by preventing the development of colorectal cancer or catching it at an early, more treatable stage.

Rep2:

(1) Yes, early screening is possible for colorectal cancer.

Colorectal cancer screening aims to detect the presence of cancer or precancerous growths in the colon or rectum before symptoms develop. Early detection through screening can significantly improve the chances of successful treatment and survival rates.

(2) The recommended screening tests for colorectal cancer include:

- Colonoscopy: This is considered the gold standard for colorectal cancer screening. It involves a flexible tube with a camera being inserted into the colon to examine the entire length of the colon and rectum. During the procedure, abnormal growths can be identified and removed.

- Fecal immunochemical test (FIT): This test checks for the presence of blood in the stool, which can be an indication of colorectal cancer. It is a non-invasive test and can be done at home using a kit to collect a stool sample.

- Flexible sigmoidoscopy: Similar to colonoscopy, but it examines only the lower part of the colon and rectum. It can help detect precancerous polyps and early-stage cancers.

- CT colonography (virtual colonoscopy): This test uses special X-ray equipment to create detailed images of the colon and rectum. It can detect polyps and cancers.

- Stool DNA test: This test examines the DNA in stool samples for specific genetic changes associated with colorectal cancer. It can be done at home, but a positive result requires follow-up with a colonoscopy.

(3) Other screening tests that may be recommended in certain cases include:

- Double-contrast barium enema: This test involves filling the colon with a liquid containing barium, followed by X-rays to create images of the colon.

- Blood-based tests: There are emerging blood tests that can detect specific biomarkers associated with colorectal cancer. These tests are still being studied and not yet widely recommended.

It's important to note that the choice of screening test may depend on various factors, including age, risk factors, personal preferences, and medical history. It's best to consult with a healthcare professional to determine the most appropriate screening strategy for an individual.

**7, In order to comprehensively evaluate colorectal carcinoma, which imaging modalities are deemed necessary?**

(1) Computed Tomography (CT) scan: CT scan is commonly used to evaluate colorectal carcinoma. It provides detailed cross-sectional images of the abdomen and pelvis, allowing for the detection and characterization of tumors, assessment of lymph node involvement, and evaluation of distant metastases.

(2) Magnetic Resonance Imaging (MRI): MRI is another valuable imaging modality for colorectal carcinoma evaluation. It offers excellent soft tissue contrast and can provide detailed information about tumor location, size, and involvement of adjacent structures. MRI is particularly useful for assessing local tumor extent and identifying tumor infiltration into surrounding organs.

(3) Colonoscopy: Although not strictly an imaging modality, colonoscopy is an essential procedure for evaluating colorectal carcinoma. It involves the use of a flexible tube with a camera to visualize the inside of the colon and rectum. Colonoscopy allows for direct visualization of the tumor, assessment of its size, location, and characteristics, as well as the ability to obtain biopsies for histopathological analysis.

(4) Positron Emission Tomography (PET) scan: PET scan is a functional imaging modality that can be used to detect distant metastases in colorectal carcinoma. It involves the injection of a radioactive tracer, usually fluorodeoxyglucose (FDG), which is taken up by metabolically active cells such as cancer cells. PET scan can help identify metastatic lesions in the liver, lungs, bones, and other distant sites.

(5) Ultrasound: Ultrasound is commonly used in the initial evaluation of colorectal carcinoma, particularly for assessing liver metastases. It is a non-invasive imaging modality that uses sound waves to create images of internal organs. Ultrasound can help identify and characterize liver lesions, guide biopsies, and assess lymph nodes in the abdomen and pelvis.

It's important to note that the specific combination of imaging modalities used may vary depending on factors such as the stage of the disease, individual patient characteristics, and institutional protocols. The choice of imaging modalities should be made in consultation with a multidisciplinary team, including radiologists, gastroenterologists, and oncologists, to ensure comprehensive evaluation and optimal patient care.

Rep2:

(1) Computed Tomography (CT) Scan: CT scans are commonly used to assess colorectal carcinoma. They provide detailed cross-sectional images of the abdomen and pelvis, allowing the detection and evaluation of primary tumors, as well as the assessment of lymph nodes, distant metastases, and potential complications.

(2) Magnetic Resonance Imaging (MRI): MRI can provide high-resolution images of the colorectal region, aiding in the evaluation of tumor extent, local invasion, lymph node involvement, and potential metastases. MRI is particularly useful for assessing the relationship between the tumor and adjacent structures, such as the mesorectal fascia.

(3) Positron Emission Tomography-Computed Tomography (PET-CT): PET-CT combines functional information from positron emission tomography (PET) with anatomical details from CT. It is valuable for staging colorectal carcinoma by detecting distant metastases, assessing tumor activity, and evaluating the response to treatment. The use of a radiotracer, usually fluorodeoxyglucose (FDG), helps identify areas of increased metabolic activity associated with cancer.

(4) Colonoscopy: Although not an imaging modality, colonoscopy plays a crucial role in the evaluation of colorectal carcinoma. It allows direct visualization of the colon and rectum, facilitating the detection and biopsy of suspicious lesions. Colonoscopy also enables the assessment of synchronous or metachronous lesions, which are important considerations in the management of colorectal carcinoma.

(5) Ultrasonography: Ultrasonography, particularly endorectal ultrasound (ERUS), is useful for evaluating the local extent of rectal tumors, assessing lymph node involvement, and identifying perirectal or perianal fistulas. ERUS can provide valuable information for surgical planning and determining the need for neoadjuvant therapy.

In summary, a comprehensive evaluation of colorectal carcinoma typically requires a combination of imaging modalities, including CT scans, MRI, PET-CT, and the use of colonoscopy. Ultrasonography, specifically ERUS, may be utilized for evaluating rectal tumors in more detail.

**8, What are the tumor markers associated with colorectal cancer? Can they replace pathological examinations?**

(1) Carcinoembryonic antigen (CEA) is one of the tumor markers associated with colorectal cancer. It is a protein that is often elevated in the blood of individuals with colorectal cancer. CEA levels can be used to monitor the response to treatment and detect cancer recurrence. However, CEA alone is not specific to colorectal cancer and can be elevated in other conditions as well.

(2) Another tumor marker associated with colorectal cancer is carbohydrate antigen 19-9 (CA 19-9). Elevated levels of CA 19-9 can indicate the presence of colorectal cancer, but like CEA, it is not specific to this type of cancer. CA 19-9 levels are commonly used in combination with other diagnostic tests to assess the extent of the disease and monitor treatment response.

(3) While tumor markers like CEA and CA 19-9 can provide valuable information in the management of colorectal cancer, they cannot replace pathological examinations. Pathological examinations involve the analysis of tissue samples obtained through biopsies or surgical resections. They provide important details about the tumor's characteristics, such as its histological type, grade, and stage. Pathological examinations also help determine the appropriate treatment strategies, including surgery, chemotherapy, or radiation therapy.

(4) Tumor markers should be considered as adjuncts to pathological examinations rather than replacements. They can aid in the diagnosis and monitoring of colorectal cancer, but pathological examinations remain crucial for accurate diagnosis, staging, and treatment planning. The combination of tumor markers and pathological examinations offers a more comprehensive approach to managing colorectal cancer patients.

Rep2:

(1) Carcinoembryonic Antigen (CEA): CEA is the most commonly used tumor marker for colorectal cancer. Elevated levels of CEA in the blood may indicate the presence of colorectal cancer. However, CEA levels can also be elevated in other conditions such as inflammatory bowel disease and certain types of benign tumors.

(2) CA 19-9: CA 19-9 is another tumor marker that can be elevated in colorectal cancer. It is particularly useful in monitoring disease progression and response to treatment. However, like CEA, CA 19-9 can also be elevated in other non-cancerous conditions.

(3) CA 125: While CA 125 is more commonly associated with ovarian cancer, elevated levels of CA 125 can also be observed in some cases of colorectal cancer, particularly those involving the peritoneum.

While tumor markers like CEA, CA 19-9, and CA 125 can provide valuable information in the diagnosis and management of colorectal cancer, they cannot replace pathological examinations. Pathological examinations, including biopsies and histological analysis, are essential for confirming the presence of cancer, determining its stage and grade, and guiding treatment decisions. Tumor markers can be helpful as adjunctive tools, but they should be interpreted in conjunction with other clinical and diagnostic information.

**9, Why is colonoscopy necessary for diagnosing colorectal cancer?**

(1) Direct visualization of the colon: During a colonoscopy, a long, flexible tube called a colonoscope is inserted into the rectum and advanced through the entire colon. The colonoscope has a camera at its tip, allowing the doctor to visually examine the inside of the colon. This direct visualization provides a detailed view of the colon's lining, enabling the detection of any abnormalities, such as tumors or polyps, that may indicate colorectal cancer.

(2) Polyp detection and removal: Colonoscopy allows for the detection and removal of polyps, which are abnormal growths that can develop into cancer over time. Polyps can be present in the colon without causing symptoms, and they may not be visible through other diagnostic methods. During a colonoscopy, if a polyp is found, it can be removed using specialized instruments, reducing the risk of it developing into cancer.

(3) Tissue biopsy: If suspicious areas or tumors are observed during the colonoscopy, the doctor can take tissue samples (biopsies) for further analysis. Biopsies allow for the examination of cells under a microscope to determine if they are cancerous or precancerous. This definitive diagnosis can guide subsequent treatment decisions.

(4) Comprehensive evaluation: Unlike other screening tests, such as fecal occult blood tests or sigmoidoscopy, colonoscopy provides a comprehensive evaluation of the entire colon. This allows for the detection of colorectal cancer at an early stage and the identification of any additional abnormalities that may require further investigation or treatment.

In summary, colonoscopy is necessary for diagnosing colorectal cancer because it provides direct visualization of the colon, enables the detection and removal of polyps, allows for tissue biopsy, and offers a comprehensive evaluation of the entire colon, leading to accurate diagnosis and appropriate management of the disease.

Rep2:

(1) Colonoscopy allows direct visualization of the colon and rectum, enabling the detection of precancerous polyps or early-stage colorectal cancer. During the procedure, a flexible tube with a camera at its end (colonoscope) is inserted into the rectum and guided through the colon. This allows doctors to examine the lining of the entire colon and identify any abnormalities.

(2) It provides a comprehensive assessment of the colon. Unlike other screening methods like stool tests, colonoscopy allows for a thorough evaluation of the colon's condition. It can detect not only polyps but also other abnormalities such as inflammation, ulcers, or bleeding, which may indicate the presence of colorectal cancer or other diseases.

(3) Colonoscopy allows for simultaneous intervention. If polyps or suspicious areas are found during the procedure, they can be immediately removed or biopsied for further examination. This enables both diagnosis and treatment in a single session, potentially preventing the progression of precancerous polyps to cancerous growths.

(4) Regular colonoscopy screenings can help detect colorectal cancer at an early stage when treatment is most effective. By identifying and removing precancerous polyps or detecting cancer in its early stages, colonoscopy can significantly reduce the mortality rate associated with colorectal cancer.

(5) Colonoscopy provides a long-term screening solution. The procedure is typically recommended every 10 years for individuals at average risk of colorectal cancer. This extended interval between screenings makes colonoscopy a cost-effective and efficient method for long-term surveillance and early detection of colorectal cancer.

(6) It offers a high detection rate. Colonoscopy has been shown to have a relatively high sensitivity and specificity for detecting colorectal cancer and precancerous lesions. This means that it has a good ability to correctly identify both the presence and absence of abnormalities, minimizing the risk of false negatives or false positives.

It's important to note that the necessity of colonoscopy may vary based on individual risk factors and medical recommendations. Consulting with a healthcare professional is crucial to determine the most appropriate screening strategy for each person.

**10, How to understand the pathology report of colorectal cancer?**

(1) Review the Diagnosis: The pathology report will provide the diagnosis of colorectal cancer. Look for key information such as the tumor type (e.g., adenocarcinoma), tumor grade (level of differentiation), and tumor stage (extent of spread).

(2) Examine Tumor Characteristics: The report will describe the characteristics of the tumor, such as its size, location, and depth of invasion into the bowel wall. This information helps determine the stage of the cancer.

(3) Assess Lymph Node Involvement: The pathology report will indicate if there is any lymph node involvement. Lymph nodes are evaluated to determine if cancer cells have spread beyond the primary tumor. The number of lymph nodes examined and the presence or absence of cancer cells in them will be noted.

(4) Analyze Margins: The report will mention the status of surgical margins. Clear margins indicate that no cancer cells were found at the edges of the tissue removed during surgery, while positive margins suggest the presence of cancer cells at the margins.

(5) Check for Molecular Markers: Some pathology reports include information about specific molecular markers, such as microsatellite instability (MSI) or expression of certain proteins (e.g., KRAS, BRAF). These markers can help guide treatment decisions and prognosis.

(6) Interpret Ancillary Tests: In some cases, additional tests like immunohistochemistry or genetic testing may have been performed on the tumor sample. The report may provide information about these tests and their results.

(7) Seek Expert Interpretation: If you are having difficulty understanding the pathology report or its implications, it is advisable to consult with your healthcare provider or a pathologist who can provide further explanation and guidance.

Rep2:

(1) Read the Diagnosis: The pathology report will start with a diagnosis that confirms the presence of colorectal cancer. It will usually include the tumor location, size, and stage. Understanding the diagnosis is crucial to grasp the overall findings.

(2) Examine Tumor Characteristics: The report will provide detailed information about the tumor characteristics, such as histological type (adenocarcinoma is the most common), grade (how abnormal the cells appear), and invasion depth (T-stage). These factors help determine the aggressiveness and potential spread of the cancer.

(3) Evaluate Lymph Node Involvement: The report will indicate whether the cancer has spread to nearby lymph nodes. It will mention the number of nodes examined and the presence of metastasis. Lymph node involvement is crucial in determining the stage of colorectal cancer.

(4) Look for Margin Status: The report will mention the status of surgical margins, which indicates whether the cancer cells extend to the edges of the removed tissue. Negative margins mean no cancer cells were found at the margins, while positive margins indicate the presence of cancer cells, suggesting incomplete removal.

(5) Check for Genetic Markers: Some pathology reports may include molecular or genetic testing results. This may involve analyzing specific genes or markers that can provide additional information about prognosis or potential targeted treatments.

(6) Consider Ancillary Tests: Depending on the case, additional tests such as immunohistochemistry (IHC) or special stains may be performed to further characterize the tumor. These tests can provide valuable information about certain biomarkers or markers of treatment response.

(7) Consult with a Healthcare Provider: If you have any doubts or questions about the pathology report, it's essential to discuss it with your healthcare provider or an oncologist. They can provide further explanation and help guide your treatment options.

Remember, interpreting a pathology report requires medical expertise, so it's crucial to seek professional guidance for a comprehensive understanding and appropriate management of colorectal cancer.

**11, Is colorectal cancer a singular disease? Why do different patients have significant variations in their survival rates?**

(1) Colorectal cancer is not a singular disease. It encompasses a broad spectrum of malignancies that affect the colon or rectum. The term "colorectal cancer" is used to describe different types of cancer that originate from the inner lining of the colon or rectum, such as adenocarcinoma, which is the most common type. However, within these types, there can be further variations in terms of location, stage, genetic mutations, and other factors that influence the behavior and prognosis of the disease.

(2) Different patients with colorectal cancer can have significant variations in their survival rates due to several factors. These factors include the stage at which the cancer is diagnosed, the presence of metastasis (spread of cancer to other organs), the aggressiveness of the tumor, the patient's overall health and age, the effectiveness of treatments received (such as surgery, chemotherapy, or radiation therapy), and the individual's response to treatment.

(3) Tumor stage plays a crucial role in determining survival rates. Colorectal cancer is typically staged from 0 to IV, with stage 0 being the earliest and stage IV being the most advanced. Patients diagnosed at an earlier stage generally have a better prognosis and higher survival rates compared to those diagnosed at later stages when the cancer has spread beyond the colon or rectum. Early detection through screening tests, such as colonoscopies, can help identify the cancer at an early stage and improve survival rates.

(4) Other factors that contribute to survival rate variations include the molecular characteristics of the tumor. Colorectal cancer can exhibit different genetic mutations, such as KRAS, BRAF, or microsatellite instability (MSI). These molecular markers can influence the aggressiveness of the cancer and its response to specific targeted therapies. Patients with certain mutations may have different outcomes and response to treatment, leading to variations in survival rates.

(5) Additionally, individual patient factors, such as age, overall health, and the presence of other medical conditions, can impact survival rates. Older patients or those with underlying health issues may have reduced survival rates due to the challenges they face in tolerating treatments or managing complications.

Rep2:

(1) Colorectal cancer is not a singular disease. It encompasses a group of cancers that originate in the colon or rectum. This includes different types such as adenocarcinomas, carcinoid tumors, gastrointestinal stromal tumors (GISTs), and lymphomas. These different types can have distinct characteristics and treatment approaches.

(2) There are several reasons why different patients with colorectal cancer may have significant variations in their survival rates. Firstly, the stage of cancer at diagnosis plays a crucial role. Patients with early-stage (localized) colorectal cancer generally have better survival rates compared to those with advanced-stage (metastatic) cancer. Early detection through screening tests can increase the chances of detecting cancer at an earlier stage.

(3) Another factor is the individual's overall health and comorbidities. Patients with underlying health conditions or weakened immune systems may experience poorer outcomes. Additionally, age can impact survival rates, with older patients often facing increased challenges in their treatment and recovery.

(4) Genetic and molecular factors also contribute to variations in survival rates. Some colorectal cancers have specific genetic mutations or molecular characteristics that can affect their aggressiveness and response to treatment. Targeted therapies tailored to these specific mutations can improve outcomes for certain patients.

(5) Lastly, access to quality healthcare and treatment resources can significantly influence survival rates. Disparities in healthcare access, including variations in screening rates, diagnostic delays, and differences in treatment availability, can contribute to differences in outcomes among different patient populations.

**12, What are the differences between colorectal polyps, colorectal adenomas, and colorectal cancer?**

(1) Colorectal polyps are abnormal growths that can develop in the lining of the colon or rectum. They are typically noncancerous (benign) and vary in size and shape. Polyps can be categorized into different types, including adenomatous polyps, hyperplastic polyps, and inflammatory polyps. While most polyps do not cause symptoms, some adenomatous polyps have the potential to progress to colorectal cancer over time.

(2) Colorectal adenomas are a specific type of polyp that has the potential to become cancerous. These polyps are characterized by glandular tissue growth in the colon or rectum lining. Adenomas are considered precancerous because they have the potential to develop into colorectal cancer. Detecting and removing adenomas during a colonoscopy is an important preventive measure to reduce the risk of colorectal cancer.

(3) Colorectal cancer, also known as colon cancer or rectal cancer, refers to malignant tumors that originate in the colon or rectum. It usually starts as a growth or polyp on the inner lining of the colon or rectum and progresses over time. If left undetected or untreated, colorectal cancer can spread to other parts of the body. Symptoms of colorectal cancer may include changes in bowel habits, blood in the stool, abdominal pain, weight loss, and fatigue.

Rep2:

(1) Colorectal polyps are abnormal growths that form on the inner lining of the colon or rectum. They can vary in size and shape and may be flat or have a stalk-like appearance. Most colorectal polyps are benign (non-cancerous) and do not cause symptoms. However, some polyps can develop into cancer over time if left untreated.

(2) Colorectal adenomas are a specific type of polyp that have the potential to become cancerous. They are characterized by abnormal growth of glandular tissue in the colon or rectum. Adenomas can be small or large and are typically identified during colonoscopy screenings. The risk of colorectal cancer increases with the size, number, and certain characteristics of adenomas.

(3) Colorectal cancer, also known as colon cancer or rectal cancer, refers to the malignant growth of cells in the colon or rectum. It typically starts as a benign polyp, such as an adenoma, and progresses into cancer over time. Colorectal cancer is one of the most common types of cancer worldwide. Symptoms may include changes in bowel habits, blood in the stool, abdominal pain, and unintended weight loss. Early detection through regular screenings is crucial for better treatment outcomes.

**13,** **Does the discovery of colorectal polyps during colonoscopy require surgery? What is the likelihood of developing colorectal cancer in this setting?**

(1) In most cases, the discovery of colorectal polyps during colonoscopy does not require immediate surgery. The appropriate treatment depends on the type, size, and number of polyps found. Many polyps can be removed during the colonoscopy procedure itself through a technique called polypectomy. During polypectomy, a small snare or wire loop is used to remove the polyp from the colon wall. This is often done using electrocautery to cut the polyp and stop any bleeding. Polypectomy is a minimally invasive procedure that can be performed without the need for open surgery.

(2) The likelihood of developing colorectal cancer in the setting of colorectal polyps depends on various factors. While the majority of polyps are benign (non-cancerous), certain types of polyps, such as adenomatous polyps, have the potential to progress to colorectal cancer over time. The size, number, and histological characteristics of the polyps are important factors in assessing the risk of cancer development. Larger polyps and those with high-grade dysplasia are generally considered to have a higher risk of malignancy. However, it is important to note that not all polyps will develop into cancer, and the majority of colorectal cancers arise from pre-existing polyps.

(3) Regular surveillance and follow-up after the removal of colorectal polyps are crucial for managing the risk of developing colorectal cancer. The surveillance intervals are determined based on the characteristics of the polyps and the individual's overall risk factors. The recommendations for surveillance colonoscopies may vary, but typically individuals with low-risk polyps may undergo follow-up colonoscopy after 5-10 years, while those with high-risk or multiple polyps may require more frequent surveillance within 3-5 years. These surveillance colonoscopies aim to detect and remove any new polyps or early-stage cancers before they progress.

(4) It is important to discuss the specific findings and recommendations with a healthcare provider who can provide personalized advice based on the individual's situation. Factors such as family history, personal medical history, and overall health play a role in determining the appropriate management plan for colorectal polyps and the risk of developing colorectal cancer.

Rep2:

(1) The discovery of colorectal polyps during colonoscopy does not always require surgery. The treatment approach depends on various factors, such as the type, size, and number of polyps found. In many cases, small polyps can be removed during the colonoscopy itself through a technique called polypectomy. This procedure involves snaring or excising the polyp using specialized instruments. However, larger or more complex polyps may require surgical intervention, such as a laparoscopic or open surgery, for complete removal.

(2) The likelihood of developing colorectal cancer in the setting of colorectal polyps depends on several factors. Some polyps, particularly adenomatous polyps, have the potential to progress to cancer over time if left untreated. However, not all polyps become cancerous. The risk of colorectal cancer depends on the histology of the polyp (e.g., adenomatous, hyperplastic), the size of the polyp, and the presence of dysplasia within the polyp.

(3) Adenomatous polyps are considered precursors to colorectal cancer. The risk of developing cancer increases with the size and number of adenomatous polyps. Large adenomatous polyps (>1 cm) have a higher risk of harboring cancer or developing cancer in the future. Additionally, the presence of high-grade dysplasia within the polyp also raises the risk of cancer.

(4) It's important to note that not all polyps are adenomatous. Hyperplastic polyps, for example, are generally considered benign and have a negligible risk of cancer progression. However, some subtypes of hyperplastic polyps, such as serrated polyps with dysplasia or sessile serrated adenomas, have an increased risk of developing into cancer.

To determine the appropriate management and surveillance plan for colorectal polyps, it is crucial to consult with a healthcare professional, typically a gastroenterologist or colorectal surgeon, who can evaluate the specific characteristics of the polyps and provide personalized recommendations based on the individual's medical history and risk factors.

**14,** **Does hemorrhoids transform into cancer? How can colorectal cancer be distinguished from hemorrhoids and gastroenteritis?**

(1) Hemorrhoids do not transform into cancer. Hemorrhoids are swollen blood vessels in the rectum or anus, and they are typically a benign condition. While hemorrhoids can cause discomfort and bleeding, they are not a precursor to or indicative of colorectal cancer. However, both hemorrhoids and colorectal cancer can cause rectal bleeding, so it's important to consult a healthcare professional for an accurate diagnosis if you experience bleeding or any other concerning symptoms.

(2) Colorectal cancer can be distinguished from hemorrhoids and gastroenteritis through various factors:

- Symptoms: Colorectal cancer symptoms can include changes in bowel habits (such as persistent diarrhea or constipation), rectal bleeding, abdominal pain, unintentional weight loss, and fatigue. Hemorrhoids, on the other hand, often cause rectal bleeding and discomfort, but rarely lead to significant changes in bowel habits or weight loss. Gastroenteritis primarily manifests as diarrhea, vomiting, abdominal pain, and sometimes fever.

- Duration and progression: Hemorrhoids typically have a fluctuating course, with symptoms that may improve or worsen over time. Colorectal cancer symptoms usually persist and progress as the disease advances. Gastroenteritis symptoms tend to develop suddenly and often resolve within a few days.

- Risk factors: While anyone can develop hemorrhoids, certain risk factors increase the likelihood of developing colorectal cancer, including a family history of the disease, age (usually over 50), a personal history of inflammatory bowel disease, and certain genetic syndromes. Gastroenteritis is commonly caused by viral or bacterial infections and can affect individuals of all ages.

To differentiate between these conditions accurately, a healthcare professional may perform a physical examination, conduct diagnostic tests (such as colonoscopy, sigmoidoscopy, or imaging studies), and review the patient's medical history.

(3) Hemorrhoids are typically diagnosed based on a physical examination, medical history, and the reported symptoms. During the examination, a healthcare professional may visually inspect the anal area and may use a gloved finger or an anoscope (a short tube-like instrument) to examine the rectum for any abnormalities or swollen blood vessels.

Colorectal cancer is diagnosed through a combination of methods, including:

- Screening tests: These include colonoscopy, sigmoidoscopy, and fecal occult blood tests. These tests can detect abnormal growths (polyps) or cancerous lesions in the colon or rectum.

- Imaging tests: Imaging studies like computed tomography (CT) scans, magnetic resonance imaging (MRI), or positron emission tomography (PET) scans may be used to evaluate the extent of the cancer and determine if it has spread to other parts of the body.

- Biopsy: If suspicious areas are detected during a screening or imaging test, a biopsy may be performed. A small tissue sample is taken for analysis to determine if it is cancerous.

Gastroenteritis is typically diagnosed based on the patient's symptoms, medical history, and physical examination. In some cases, laboratory tests may be conducted, such as stool analysis or culture, to identify the specific cause of the infection.

Rep2:

(1) No, hemorrhoids do not transform into cancer. Hemorrhoids are swollen blood vessels in the rectum or anus that can cause discomfort and bleeding. While they can be painful and bothersome, they are not a precursor to or a form of colorectal cancer.

(2) Colorectal cancer and hemorrhoids can have overlapping symptoms, but there are several key differences that can help distinguish them. Colorectal cancer is a malignant growth in the colon or rectum, whereas hemorrhoids are swollen blood vessels.

To differentiate between the two, it's important to consider additional factors. Colorectal cancer often presents with persistent changes in bowel habits (such as persistent diarrhea or constipation), rectal bleeding, unexplained weight loss, fatigue, and abdominal pain. On the other hand, hemorrhoids usually cause symptoms such as itching, discomfort, pain during bowel movements, and bright red blood in the stool or on the toilet paper.

(3) Gastroenteritis, also known as stomach flu, is an inflammation of the stomach and intestines usually caused by a viral or bacterial infection. While gastroenteritis can cause abdominal pain, diarrhea, vomiting, and sometimes blood in the stool, it is generally an acute condition that resolves within a few days or weeks.

In contrast, colorectal cancer is a chronic and progressive disease that tends to worsen over time if left untreated. Additionally, colorectal cancer may be associated with other symptoms such as anemia, changes in stool caliber, and the presence of a mass or tumor on a physical examination or imaging tests.

**The Surgery Management**

**1, What does the structure of the colon resemble? What functions does it serve?**

(1) The structure of the colon resembles a long, coiled tube. It is part of the large intestine, which is the final segment of the digestive system in humans.

(2) The colon serves several important functions in the body. It is responsible for absorbing water, electrolytes, and vitamins produced by the gut bacteria. It also plays a role in the formation and storage of feces. Additionally, the colon helps in the absorption of certain nutrients and facilitates the elimination of waste materials from the body.

(3) Another function of the colon is to house a diverse population of beneficial bacteria, known as the gut microbiota. These bacteria help in the fermentation of indigestible carbohydrates and the production of certain vitamins, such as vitamin K and some B vitamins. The colon provides a suitable environment for these bacteria to thrive and contribute to overall digestive health.

(4) Additionally, the colon has muscular walls that contract and relax in a coordinated manner, allowing for the movement of digested material through the colon towards the rectum. This peristaltic movement helps in the propulsion of feces and aids in the process of defecation.

Rep2:

(1) The structure of the colon resembles a long, hollow, muscular tube. It is part of the large intestine and is the final segment of the digestive tract.

(2) The colon serves several important functions in the body. It is responsible for the absorption of water, electrolytes, and nutrients from the remaining undigested food material. It also plays a crucial role in the formation and elimination of feces, as it reabsorbs water from the digested material, leaving behind more solid waste.

(3) Another function of the colon is the fermentation of certain indigestible carbohydrates by the gut microbiota. This process produces short-chain fatty acids, which serve as an energy source for the cells lining the colon and contribute to overall gut health.

(4) Additionally, the colon helps in the storage and temporary storage of fecal matter until it is eliminated from the body during a bowel movement. It also aids in the propulsion of feces towards the rectum through coordinated muscular contractions called peristalsis.

Overall, the colon plays a vital role in the final stages of digestion, absorption, water balance, and elimination of waste materials from the body.

**2, Does the surgical approach remain consistent regardless of the location of the tumor within the colon?**

(1) Generally, the surgical approach for colon tumors does not remain consistent regardless of the location within the colon. The specific surgical technique and approach may vary depending on factors such as the size and location of the tumor, the stage of the cancer, and the overall health of the patient.

(2) In cases where the tumor is located in the lower part of the colon, closer to the rectum, a surgical procedure called a low anterior resection may be performed. This involves removing the tumor along with a portion of the colon and reattaching the remaining healthy sections. The rectum is usually preserved, allowing for normal bowel function to be maintained.

(3) When the tumor is located in the upper part of the colon, closer to the cecum, a procedure called a right colectomy or extended right hemicolectomy may be performed. This involves removing the tumor along with the ascending colon, cecum, and sometimes a portion of the transverse colon. The remaining portions of the colon are then connected to ensure continuity of the digestive tract.

(4) For tumors located in the middle part of the colon, a procedure called a transverse colectomy may be performed. This involves removing the tumor along with a portion of the transverse colon, and the remaining sections of the colon are reconnected.

Rep2:

(1) The surgical approach for colon tumors generally follows certain principles but may vary depending on the specific location of the tumor within the colon. In most cases, the primary goal is to remove the tumor along with an adequate margin of healthy tissue to ensure complete excision. This is usually achieved through surgical resection, which involves removing the affected portion of the colon and reattaching the remaining ends. The surgical approach may involve open surgery, where a large incision is made in the abdomen, or minimally invasive techniques such as laparoscopic or robotic-assisted surgery.

(2) The location of the tumor within the colon can influence the extent and type of surgery performed. For instance, if the tumor is located in the sigmoid colon or rectum, a procedure called a sigmoid colectomy or low anterior resection may be performed to remove the tumor and a portion of the sigmoid colon or rectum. In cases where the tumor is located in the ascending or transverse colon, a right or left hemicolectomy may be necessary, respectively, to remove the affected segment of the colon. The specific surgical approach will be determined by factors such as tumor size, stage, and the overall health of the patient.

(3) The surgical approach also takes into account the lymphatic drainage of the colon. Lymph nodes in the surrounding area may be removed along with the tumor to assess whether the cancer has spread. This procedure, known as lymphadenectomy or lymph node dissection, helps in staging the cancer and guiding further treatment decisions. The number of lymph nodes removed during surgery is important for accurate staging and prognosis.

(4) In some cases, depending on the location and stage of the tumor, additional procedures may be necessary. For example, if the tumor has spread to nearby organs or structures, such as the liver or small intestine, additional resections or surgical interventions may be required to remove the affected areas. The surgical approach may also be influenced by the presence of any pre-existing medical conditions that could impact the choice of anesthesia, the patient's ability to tolerate surgery, or the type of surgical technique employed.

**3, What are the surgical methods for rectal cancer?**

(1) The surgical methods for rectal cancer generally include the following approaches:

a) Local Excision: This method is suitable for early-stage rectal cancer that has not spread beyond the inner layers of the rectum. During a local excision, the surgeon removes the cancerous tumor and a small amount of surrounding healthy tissue.

b) Transanal Endoscopic Microsurgery (TEM): TEM is a minimally invasive technique that allows for the removal of larger rectal tumors through the anus. It involves the use of an endoscope and specialized instruments to perform the surgery.

c) Transanal Minimally Invasive Surgery (TAMIS): TAMIS is a newer technique similar to TEM but uses a different set of specialized instruments. It provides a minimally invasive approach for removing rectal tumors and allows for improved visualization and precision.

(2) Another surgical method for rectal cancer is Total Mesorectal Excision (TME):

TME is the standard surgical procedure for mid and low rectal cancer. It involves the complete removal of the rectum along with the surrounding fatty tissue known as the mesorectum. This technique aims to achieve clear margins and minimize the risk of local recurrence.

During TME, the surgeon makes an incision in the abdomen and carefully dissects and removes the rectum and mesorectum. In some cases, the surgeon may also perform a temporary or permanent colostomy, which involves creating an opening (stoma) in the abdominal wall to divert the bowel contents.

(3) Another surgical method for rectal cancer is Abdominoperineal Resection (APR):

APR is typically used for low rectal cancer or when the tumor is very close to the anus. In this procedure, the surgeon removes the entire rectum, anus, and surrounding tissues. The remaining colon is then brought out through the abdominal wall to create a permanent colostomy.

APR involves two incisions: one in the abdomen and one in the perineal area. The abdominal incision allows for the removal of the rectum, while the perineal incision enables the dissection and removal of the anus and surrounding tissues.

These are some of the surgical methods commonly used for treating rectal cancer. The choice of technique depends on various factors such as the stage and location of the tumor, the patient's overall health, and the surgeon's expertise. It is important for individuals diagnosed with rectal cancer to consult with a qualified healthcare professional to determine the most suitable surgical approach for their specific case.

Rep2:

(1) One of the surgical methods for rectal cancer is a low anterior resection (LAR). In this procedure, the portion of the rectum affected by cancer is removed, and the remaining healthy portions of the rectum and colon are reconnected. LAR is typically performed for rectal tumors located in the upper and middle parts of the rectum. It can be done through open surgery or minimally invasive techniques such as laparoscopy or robotic-assisted surgery.

(2) Another surgical method for rectal cancer is an abdominoperineal resection (APR). This procedure is usually performed for tumors located in the lower part of the rectum or the anus. During an APR, both the rectum and the anus are removed. The surgeon creates a permanent colostomy, which is an opening on the abdominal wall through which waste is eliminated into a colostomy bag. This surgery may be necessary if the tumor is close to or involves the anal sphincter.

(3) Transanal excision is a surgical method used for early-stage rectal cancers that are small and localized. This procedure involves accessing the rectum through the anus and removing the tumor. It is typically performed using specialized instruments inserted through the anus. Transanal excision is a less invasive approach compared to other surgeries, and it may be suitable for select patients with small, superficial rectal tumors that have not spread to nearby lymph nodes.

(4) Total mesorectal excision (TME) is a technique commonly used during rectal cancer surgery. It involves removing the rectum along with the surrounding lymph nodes and fatty tissue called the mesorectum. TME aims to achieve complete removal of the tumor and the associated lymphatic drainage, reducing the risk of local recurrence. This procedure can be performed using open surgery or minimally invasive techniques. TME is often combined with either LAR or APR, depending on the location of the tumor.

**4, What does MDT, often mentioned by doctors, refer to?**

(1) MDT, or Multidisciplinary Team, is a term frequently used by doctors in healthcare settings. It refers to a collaborative approach where professionals from various disciplines come together to provide comprehensive care to patients. The team typically includes doctors, nurses, specialists, therapists, and other healthcare professionals who work collectively to develop treatment plans and make decisions about patient care.

(2) The main purpose of an MDT is to ensure that patients receive well-coordinated and integrated care. By bringing together experts from different fields, the team can pool their knowledge and skills to provide a holistic approach to diagnosis, treatment, and management of complex medical conditions. This collaborative effort enhances the quality of care and improves patient outcomes.

(3) Another important aspect of MDTs is their ability to facilitate effective communication and information sharing among healthcare professionals. By having regular meetings and discussions, team members can exchange insights, review test results, discuss treatment options, and address any concerns or challenges related to patient care. This multidisciplinary collaboration helps in making well-informed decisions and avoids fragmented or disjointed care.

(4) MDTs are commonly used in various medical specialties, including but not limited to oncology, cardiology, neurology, and mental health. In oncology, for example, an MDT may consist of oncologists, surgeons, radiologists, pathologists, and nurses who work together to determine the most suitable treatment plan for a cancer patient. Similarly, in mental health, an MDT may involve psychiatrists, psychologists, social workers, and occupational therapists who collaborate to provide comprehensive care for individuals with mental health conditions.

(5) In summary, MDTs play a crucial role in delivering patient-centered care by promoting collaboration, communication, and integration among healthcare professionals. Through their multidisciplinary approach, MDTs aim to optimize treatment outcomes and improve the overall quality of care provided to patients.

Rep2:

(1) MDT, or Multidisciplinary Team, is a term frequently used in the medical field. It refers to a collaborative approach to patient care involving a diverse group of healthcare professionals from various specialties. These professionals come together as a team to collectively discuss, evaluate, and plan the most appropriate treatment and management strategies for patients with complex medical conditions.

(2) The primary goal of an MDT is to ensure that patients receive comprehensive and holistic care that addresses all aspects of their health. By pooling together, the expertise and perspectives of different healthcare professionals, MDTs can provide a more thorough assessment of patients' conditions, leading to more accurate diagnoses and tailored treatment plans.

(3) MDTs typically consist of healthcare professionals from different disciplines, such as doctors, nurses, surgeons, radiologists, pathologists, pharmacists, psychologists, and other specialists relevant to the specific patient's needs. Each team member brings their unique knowledge and expertise to the table, contributing to a more well-rounded understanding of the patient's condition.

(4) During MDT meetings, team members discuss patients' medical histories, diagnostic test results, imaging studies, and any other relevant information. They share their insights, opinions, and recommendations, allowing for a comprehensive evaluation of treatment options. This collaborative decision-making process helps ensure that the treatment plan is tailored to the patient's specific needs, taking into account various factors such as medical evidence, the patient's preferences, and potential risks and benefits.

(5) MDTs are commonly used in the management of complex conditions, such as cancer, cardiovascular diseases, chronic pain, and mental health disorders. By fostering collaboration and communication among healthcare professionals, MDTs facilitate a coordinated and integrated approach to patient care, ultimately improving outcomes and enhancing the overall quality of healthcare delivery.

**5, What are the differences between traditional open surgery, laparoscopic surgery, and robotic surgery?**

(1) In traditional open surgery, a large incision is made in the body to access the surgical site. This allows the surgeon to directly view and access the organs or tissues requiring treatment. The incision provides a wide and direct field of vision, facilitating manual manipulation of instruments and suturing. However, it involves more tissue trauma, longer recovery times, and a higher risk of complications due to the larger incision.

(2) Laparoscopic surgery, also known as minimally invasive surgery, involves making several small incisions in the abdomen through which specialized surgical instruments and a tiny camera called a laparoscope are inserted. The laparoscope provides visualization of the surgical site by transmitting images to a monitor. With laparoscopic instruments, the surgeon performs the procedure while viewing the magnified images on the monitor. This approach reduces tissue trauma, offers shorter recovery times, and leads to fewer complications compared to open surgery.

(3) Robotic surgery combines the benefits of laparoscopic surgery with enhanced precision and control provided by robotic systems. A surgeon operates the robotic system from a console, controlling robotic arms equipped with surgical instruments. The system translates the surgeon's movements into precise actions, enabling highly accurate maneuvers. Robotic surgery offers improved dexterity, enhanced visualization with 3D imaging, and the ability to perform complex procedures with smaller incisions. It also allows for better ergonomics for the surgeon, reducing fatigue during longer surgeries.

(4) Unlike open surgery, both laparoscopic and robotic surgery require smaller incisions, resulting in reduced postoperative pain, less scarring, and shorter hospital stays. However, robotic surgery offers additional advantages over laparoscopic surgery, such as increased range of motion for the instruments, improved visualization, and greater surgical precision. Robotic surgery can be particularly beneficial for procedures that involve delicate or intricate movements, such as prostate surgery or complex gynecological procedures. However, robotic surgery may be more expensive and requires specialized training for surgeons to operate the robotic system effectively.

Rep2:

(1) Traditional Open Surgery:

Traditional open surgery involves making a large incision in the body to access and operate on the affected area.

Surgeons have direct physical access to the surgical site, allowing them to manipulate tissues and organs with their hands.

This approach provides excellent visualization and tactile feedback, making it suitable for complex procedures.

However, open surgery requires a longer recovery time and may result in more post-operative pain and scarring due to the larger incision.

(2) Laparoscopic Surgery:

Laparoscopic surgery, also known as minimally invasive surgery, uses small incisions and specialized instruments to perform procedures.

A laparoscope, a thin tube with a camera and light source, is inserted through one incision to provide a view of the surgical site on a monitor.

Surgeons use additional small incisions to insert surgical instruments and manipulate tissues.

Laparoscopic surgery offers several advantages, including reduced pain, smaller incisions, shorter hospital stays, and quicker recovery compared to open surgery.

However, it can be challenging for surgeons due to the limited field of view, restricted range of motion, and lack of tactile feedback.

(3) Robotic Surgery:

Robotic surgery utilizes robotic systems controlled by surgeons to perform procedures.

The surgeon sits at a console and operates robotic arms equipped with miniature instruments.

The system translates the surgeon's hand movements into precise movements of the robotic instruments inside the patient's body.

The robotic system provides enhanced visualization with a 3D high-definition view and offers improved dexterity and range of motion compared to laparoscopic surgery.

Robotic surgery allows for greater precision, reduced tremor, and the ability to access difficult-to-reach areas.

Like laparoscopic surgery, robotic surgery offers benefits such as smaller incisions, less pain, reduced blood loss, shorter hospital stays, and faster recovery.

(4) Endoscopic Surgery:

Endoscopic surgery refers to procedures performed using an endoscope, a flexible tube with a light and camera attached.

Endoscopes are inserted through natural body openings or small incisions to visualize and treat internal structures.

This technique is commonly used in areas like the gastrointestinal tract, respiratory system, and joints.

Endoscopic surgery minimizes the need for large incisions, reduces trauma to surrounding tissues, and often leads to faster recovery and shorter hospital stays.

However, it may have limitations in terms of instrument manipulation and access to deeper structures compared to laparoscopic or robotic surgery.

**6, What tests and preparations are needed for patients with colorectal cancer before surgery?**

(1) Preoperative evaluation: Before surgery for colorectal cancer, patients undergo a comprehensive preoperative evaluation. This involves a detailed medical history assessment, physical examination, and review of previous imaging studies and laboratory tests. The purpose is to assess the patient's overall health, identify any potential risks or complications, and plan appropriate surgical interventions.

(2) Diagnostic imaging: Imaging studies play a crucial role in evaluating the extent and location of the colorectal tumor, as well as identifying potential metastasis. Common imaging techniques used include computed tomography (CT) scans, magnetic resonance imaging (MRI), and positron emission tomography (PET) scans. These imaging modalities provide detailed information about the tumor size, invasion into surrounding tissues, involvement of nearby lymph nodes, and presence of distant metastasis.

(3) Laboratory tests: Various laboratory tests are conducted to assess the patient's general health status and to identify any underlying medical conditions that may impact the surgical procedure or subsequent treatment. These tests typically include a complete blood count (CBC), liver function tests, kidney function tests, coagulation profile, and tumor markers such as carcinoembryonic antigen (CEA). The results help in evaluating the patient's overall fitness for surgery and aid in planning the appropriate perioperative management.

(4) Colonoscopy: Colonoscopy is a crucial step in the preoperative workup for colorectal cancer. It involves inserting a flexible tube with a camera into the colon to visualize the tumor and surrounding tissues directly. Colonoscopy helps determine the precise location and characteristics of the tumor, allows for biopsy or removal of polyps, and aids in assessing the extent of bowel involvement. Additionally, it helps in identifying synchronous lesions or other abnormalities in the colon that may require further evaluation or treatment.

(5) Staging investigations: To determine the stage of colorectal cancer, additional investigations may be performed. These can include endorectal ultrasound, which helps evaluate the depth of tumor invasion into the rectal wall and involvement of nearby lymph nodes, and computed tomography (CT) or magnetic resonance imaging (MRI) of the chest, abdomen, and pelvis. These imaging studies assist in assessing the spread of cancer to distant sites and aid in determining the appropriate surgical approach and the need for additional treatments such as chemotherapy or radiation therapy.

(6) Cardiac and pulmonary assessments: Since surgery for colorectal cancer is performed under general anesthesia, patients need to undergo a cardiac evaluation, which may include an electrocardiogram (ECG), echocardiogram, or stress test, depending on the patient's risk factors and medical history. Pulmonary function tests may also be conducted to assess lung function, especially in patients with a history of respiratory conditions or heavy smoking, to optimize respiratory function during and after surgery.

(7) Preoperative counseling and education: Patients scheduled for colorectal cancer surgery receive preoperative counseling and education. This involves discussions with the surgical team to explain the procedure, potential risks and complications, expected outcomes, and postoperative care. Patients are advised about dietary modifications, bowel preparation, and any necessary lifestyle changes before surgery. They may also be referred to other healthcare professionals, such as dieticians or physical therapists, to optimize their overall health and facilitate a smooth recovery.

Rep2:

(1) Preoperative tests for colorectal cancer typically include:

Blood tests: These tests evaluate the overall health status of the patient and may include a complete blood count (CBC), liver function tests, kidney function tests, and tumor marker tests such as carcinoembryonic antigen (CEA) levels. Blood tests help assess organ function and provide baseline information for comparison after surgery.

(2) Imaging studies play a crucial role in staging the cancer and determining the extent of its spread. Common imaging tests include:

Computed tomography (CT) scan: This imaging technique provides detailed cross-sectional images of the abdomen and pelvis. It helps identify the primary tumor, evaluate nearby lymph nodes, and detect any metastatic spread to other organs.

Magnetic resonance imaging (MRI): MRI uses magnetic fields and radio waves to create detailed images of soft tissues. It is particularly useful in assessing the rectal cancer stage, invasion depth, and involvement of adjacent structures.

Chest X-ray or chest CT scan: These tests evaluate the presence of any lung metastases or other abnormalities in the chest.

(3) Additional tests may be required to assess the patient's overall fitness for surgery and to guide surgical planning. These may include:

Colonoscopy: This procedure allows direct visualization of the colon and rectum using a flexible tube with a camera. It helps identify the precise location and characteristics of the tumor, as well as the presence of any synchronous lesions in other parts of the colon.

Endorectal ultrasound (ERUS): ERUS utilizes ultrasound waves to assess the depth of tumor invasion, involvement of nearby structures, and presence of regional lymph node metastases in rectal cancer cases.

Pulmonary function tests: These tests measure lung capacity and function to determine if the patient's respiratory system can tolerate general anesthesia and surgery.

Electrocardiogram (ECG) and echocardiogram: These tests assess the heart's electrical activity and function, ensuring the patient is fit for surgery.

These tests and preparations help healthcare providers gather essential information about the patient's cancer stage, overall health, and anatomical considerations, allowing for appropriate surgical planning and ensuring patient safety during and after the procedure.

**7, What are the complications after colorectal cancer surgery and how should they be treated?**

(1) One potential complication after colorectal cancer surgery is infection. Surgical site infections can occur, leading to symptoms such as redness, swelling, warmth, and pain around the incision site. In severe cases, there may be drainage of pus or fever. Treatment of surgical site infections typically involves antibiotics to control the infection, and in some cases, drainage of any abscesses that may have formed.

(2) Another complication that can arise after colorectal cancer surgery is anastomotic leakage. This occurs when the surgical connection (anastomosis) between the remaining parts of the colon or rectum fails to heal properly, resulting in a leakage of contents from the bowel. Symptoms of anastomotic leakage may include abdominal pain, fever, increased heart rate, and changes in bowel movements. Treatment usually involves surgical intervention to repair the leakage and may also include antibiotics and supportive care.

(3) Bowel obstruction is another potential complication after colorectal cancer surgery. It can occur due to scar tissue formation, adhesions, or narrowing of the bowel lumen. Symptoms of bowel obstruction may include abdominal pain, bloating, nausea, vomiting, and constipation. The treatment depends on the severity of the obstruction and may involve bowel rest, nasogastric decompression, intravenous fluids, and sometimes surgery to relieve the obstruction.

(4) Hemorrhage or bleeding is a possible complication after colorectal cancer surgery. It can occur due to damage to blood vessels during the operation or delayed bleeding from the surgical site. Signs of bleeding may include bright red or dark stools, low blood pressure, rapid heart rate, and weakness. Treatment may involve blood transfusions, surgical intervention to control the bleeding source, and supportive measures such as intravenous fluids and monitoring.

(5) Other potential complications include deep vein thrombosis (blood clot formation in the deep veins), pulmonary embolism (when a blood clot travels to the lungs), and urinary or sexual dysfunction. These complications require early recognition and prompt management. Prevention strategies such as early mobilization, compression stockings, and blood-thinning medications may be employed to minimize the risk.

Rep2:

(1) Postoperative Infection: One complication that can occur after colorectal cancer surgery is postoperative infection. Surgical wounds can become infected, leading to symptoms such as redness, swelling, warmth, and pain around the incision site. In severe cases, fever and pus discharge may also be present. Treatment typically involves administration of antibiotics to target the specific infectious organisms and, in some cases, surgical drainage of the infected area.

(2) Anastomotic Leakage: Another potential complication is anastomotic leakage, which refers to the breakdown or leakage of the connection (anastomosis) created between two segments of the intestine during surgery. This can lead to the leakage of intestinal contents into the abdominal cavity, causing infection and inflammation. Patients may experience symptoms such as fever, abdominal pain, bloating, and a change in bowel movements. Treatment usually involves surgical intervention to repair the leakage and drain any accumulated fluid or abscesses.

(3) Bowel Obstruction: Bowel obstruction can occur after colorectal cancer surgery due to the narrowing or blockage of the intestines. This can result from scar tissue formation, adhesions, or the development of a new tumor. Symptoms may include severe abdominal pain, nausea, vomiting, and the inability to pass stool or gas. The treatment approach depends on the cause and severity of the obstruction and may involve conservative management, such as bowel rest and medication, or surgical intervention to relieve the blockage.

(4) Deep Vein Thrombosis (DVT) and Pulmonary Embolism (PE): Surgery for colorectal cancer can increase the risk of developing blood clots in the deep veins of the legs (DVT), which can potentially dislodge and travel to the lungs, causing a pulmonary embolism (PE). Patients may experience swelling, pain, or redness in the affected leg, and in the case of PE, symptoms can include chest pain, shortness of breath, and rapid heart rate. Treatment typically involves blood-thinning medications to prevent the clot from worsening or new clots from forming. Additionally, compression stockings or intermittent pneumatic compression devices may be used to improve blood flow and reduce the risk of clot formation.

(5) Urinary and Sexual Dysfunction: Colorectal cancer surgery, particularly when involving the rectum, can sometimes lead to urinary and sexual dysfunction. Urinary problems may include difficulties with urination or incontinence, while sexual dysfunction can manifest as erectile dysfunction in men or pain and reduced lubrication in women. Treatment options may include medications, pelvic floor exercises, or referral to specialists such as urologists or sexual health experts to address specific issues.

**8, How long after colorectal cancer surgery can a patient be discharged from the hospital?**

(1) After colorectal cancer surgery, the duration of hospital stay can vary depending on several factors, including the type of surgery performed, the patient's overall health, and the specific circumstances of the case. Generally, the length of stay ranges from a few days to a week or more.

(2) The first point to consider is the type of surgery performed. If the patient underwent a minimally invasive procedure such as laparoscopic or robotic surgery, the recovery time is often shorter compared to traditional open surgery. Minimally invasive techniques typically result in less pain, reduced postoperative complications, and faster recovery, allowing patients to be discharged earlier.

(3) Another important factor is the patient's overall health and response to surgery. Individuals with better general health and no significant complications during or after surgery may be able to leave the hospital sooner. Conversely, if the patient experiences complications such as infections, excessive bleeding, or bowel dysfunction, their discharge may be delayed until these issues are properly addressed.

(4) Additionally, the specific circumstances of the case can influence the length of hospital stay. For instance, if the surgery involved removing a larger tumor or required additional procedures such as an ostomy creation or reconstructive surgery, the recovery period may be longer, leading to a more extended hospital stay. Conversely, if the surgery was performed at an early stage of colorectal cancer with minimal complications, the patient may be discharged relatively sooner.

It is important to note that these points are general guidelines, and each patient's situation is unique.

Rep2:

(1) The length of hospital stay after colorectal cancer surgery can vary depending on several factors. Generally, the patient can be discharged within a week after the surgery, but the exact timing may differ based on individual circumstances.

(2) The type of surgery performed plays a crucial role in determining the length of hospital stay. Minimally invasive procedures, such as laparoscopic or robotic surgery, often result in shorter hospital stays compared to open surgery. This is because minimally invasive techniques typically involve smaller incisions, less tissue trauma, and faster recovery.

(3) The patient's overall health condition and postoperative recovery progress also influence the timing of discharge. If the patient experiences any complications or requires additional medical care, such as drainage of fluid collections or treatment for infection, their hospital stay may be extended. Conversely, if the patient recovers well without any complications, they may be discharged earlier.

(4) The extent and complexity of the surgery performed can impact the length of hospital stay. For instance, if a patient undergoes a simple tumor resection without the need for additional procedures like bowel resection or colostomy, they may be discharged sooner. On the other hand, if the surgery involves more extensive resection or reconstruction, the hospital stay may be prolonged to ensure proper monitoring and recovery.

It is important to note that these points provide a general overview, and the actual duration of hospital stay after colorectal cancer surgery can vary based on individual factors and the specific details of the case. The healthcare team will assess the patient's condition and make decisions regarding discharge based on the best interest and well-being of the patient.

**9, What can I eat after just having colorectal cancer surgery?**

(1) Following colorectal cancer surgery, it's important to focus on a diet that promotes healing, minimizes digestive discomfort, and supports overall health. Here are some general dietary considerations:

- Soft and easily digestible foods: In the initial stages after surgery, you may want to start with a diet consisting of soft and easily digestible foods. This can include foods like cooked vegetables, tender meats, fish, poultry, tofu, well-cooked grains, mashed potatoes, and soft fruits.

(2) Adequate fiber intake: Gradually introducing fiber into your diet is crucial for maintaining bowel regularity and preventing constipation. However, it's important to consult with your healthcare provider regarding the appropriate timing for increasing your fiber intake. Initially, you may start with low-fiber foods and then gradually incorporate high-fiber foods such as whole grains, legumes, fruits, and vegetables.

(3) Hydration: Staying hydrated is essential for your overall health, especially after surgery. Drinking enough water throughout the day helps prevent dehydration and keeps your digestive system functioning properly. Adequate hydration also promotes soft and easy bowel movements.

(4) Managing potential side effects: After colorectal cancer surgery, you may experience certain side effects such as diarrhea, nausea, or gas. To manage these symptoms, it may be helpful to avoid spicy or greasy foods, carbonated drinks, caffeine, and alcohol. Instead, opt for smaller, frequent meals, and consider consuming ginger, peppermint, or chamomile tea to soothe your digestive system.

Rep2:

(1) After colorectal cancer surgery, it is important to follow a nutritious diet that supports healing and recovery. Here are some general guidelines to consider:

- Focus on a well-balanced diet: Include a variety of foods from different food groups to ensure you're getting essential nutrients. This typically involves consuming adequate amounts of fruits, vegetables, whole grains, lean proteins, and healthy fats.

(2) Stay hydrated: Proper hydration is crucial for recovery. Drink plenty of fluids, such as water, herbal teas, and clear broths, to prevent dehydration and support healing. However, it's important to follow any specific fluid restrictions advised by your healthcare team.

(3) Choose soft and easily digestible foods: Initially, after surgery, it is common to have a limited appetite and experience difficulty chewing or swallowing. Opt for soft foods that are gentle on the digestive system, such as cooked vegetables, mashed potatoes, yogurt, smoothies, soups, and pureed fruits. These foods are generally easier to tolerate and can help prevent discomfort or complications.

(4) Gradually reintroduce fiber: As you recover, slowly introduce fiber-rich foods into your diet. Fiber promotes regular bowel movements, which can be important after colorectal surgery. Start with easily digestible sources of fiber, such as cooked vegetables and fruits without skins, and gradually include whole grains, legumes, and nuts. However, it's essential to consult your healthcare team regarding the appropriate timing and amount of fiber for your specific recovery process.

**10, What are the impacts on urination, defecation, and sexual function after surgery for middle and low rectal cancer?**

(1) Urination: Surgery for middle and low rectal cancer can potentially impact urination due to the close proximity of the rectum to the urinary tract. The surgical procedure may involve manipulating or removing nearby structures such as the prostate gland in males or the uterus in females, which can affect urinary function. Additionally, the surgical trauma and potential nerve damage can lead to temporary or permanent changes in bladder control, urinary urgency, frequency, or incontinence. These effects may vary depending on the individual, the extent of the surgery, and other factors.

(2) Defecation: Surgery for middle and low rectal cancer can significantly affect defecation or bowel movements. Depending on the stage and location of the cancer, a procedure called total mesorectal excision (TME) may be performed, which involves removing the rectum and surrounding tissues. This can result in changes in bowel function, such as increased frequency, urgency, or the development of loose stools or diarrhea. Some individuals may also experience difficulty controlling bowel movements, leading to fecal incontinence. These changes can be distressing but are often managed with dietary modifications, medication, and sometimes, the use of assistive devices such as colostomy bags.

(3) Sexual function: Surgery for middle and low rectal cancer can have an impact on sexual function, particularly in cases where the surgery involves the removal of the rectum or adjacent reproductive organs. In males, the surgical removal of the prostate gland or damage to the nerves responsible for erectile function can lead to difficulties achieving or maintaining an erection (erectile dysfunction). In females, surgery involving the removal of the uterus or damage to pelvic nerves can affect sexual sensation and arousal. Additionally, both males and females may experience changes in libido or sexual desire following surgery. It is important for patients to discuss their concerns with healthcare providers who can provide guidance and potential solutions such as medication or referral to sexual health specialists.

(4) Psychological and emotional impacts: It is worth noting that surgery for middle and low rectal cancer can also have psychological and emotional impacts on individuals. Coping with the changes in urinary, bowel, and sexual functions can lead to feelings of distress, embarrassment, and reduced self-esteem. These changes may also impact personal relationships and sexual intimacy, potentially causing strain or difficulties in the affected person's life. Addressing these concerns through open communication with healthcare professionals, support groups, or counseling services can be beneficial in managing the emotional aspects of post-surgical changes.

Rep2:

(1) After surgery for middle and low rectal cancer, there can be impacts on urination. The close proximity of the rectum to the urinary system can lead to potential disturbances. Surgery may involve manipulation of nearby structures, such as the bladder or prostate in men, which can result in temporary or permanent changes in urination. Common issues include increased urinary frequency, urgency, difficulty initiating urination, or weak urine flow.

(2) Surgery for middle and low rectal cancer can also affect defecation. The rectum plays a crucial role in the elimination of feces, and its surgical removal or alteration can lead to changes in bowel movements. Following surgery, patients may experience temporary or permanent alterations in bowel habits. These changes can include diarrhea, constipation, or an urgency to pass stools. Some individuals may require adjustments in their diet or the use of medications to manage these issues effectively.

(3) Another area that can be impacted after surgery for middle and low rectal cancer is sexual function. The proximity of the rectum to the reproductive organs can result in changes that affect sexual activity and satisfaction. In men, surgery may involve the removal of the rectum and adjacent structures like the prostate, which can lead to erectile dysfunction or difficulty achieving orgasm. Women may experience decreased vaginal lubrication or changes in sensation. These effects can have emotional and psychological impacts, and it is important for patients to discuss their concerns with healthcare providers who can provide appropriate support and guidance.

(4) It is worth noting that the specific impacts on urination, defecation, and sexual function can vary depending on the individual, the extent of the surgery, and other factors. Every patient's experience will be unique, and it is crucial to have open and honest discussions with healthcare professionals to address any concerns and receive personalized care. Rehabilitation programs, such as pelvic floor exercises or working with specialists like urologists or sexual therapists, may also be beneficial in managing and improving post-surgery outcomes in these areas.

**11, Why is an ileostomy created during colorectal cancer surgery? And when can a reversal** **procedure be performed after the surgery?**

(1) An ileostomy is created during colorectal cancer surgery for several reasons. One of the primary reasons is to bypass a diseased or blocked section of the large intestine or rectum. By creating an ileostomy, the surgeon diverts the small intestine to an opening in the abdominal wall, known as a stoma. This allows waste products to bypass the affected area of the colon or rectum, facilitating healing and reducing the risk of complications.

(2) Another reason for creating an ileostomy is to provide a route for the elimination of fecal matter when the lower part of the colon or rectum is removed. In cases where a significant portion of the colon or rectum is affected by cancer or other conditions, removing the affected portion becomes necessary. An ileostomy allows stool to exit the body through the stoma, preventing it from passing through the remaining section of the colon or rectum.

(3) Additionally, an ileostomy can be beneficial in situations where the anastomosis (reconnection of the remaining colon or rectum) is not feasible or safe immediately after the initial surgery. In some cases, the remaining bowel may need time to heal before the reversal procedure can be performed. By diverting the flow of waste through the stoma, an ileostomy helps protect the healing area, reducing the risk of infection and allowing adequate time for recovery.

(4) The reversal procedure, also known as ileostomy closure, can typically be performed after a few months to allow the body enough time to heal and recover from the initial surgery. The timing of the reversal procedure depends on various factors, including the individual's overall health, the extent of the initial surgery, and the healing progress of the remaining bowel. Typically, the surgeon will assess the condition of the bowel, perform tests to ensure its readiness, and make a decision on the appropriate time for the reversal. It is important to note that the reversal procedure is a separate surgery that involves reconnecting the remaining colon or rectum and closing the stoma, allowing the resumption of normal bowel function.

Rep2:

(1) During colorectal cancer surgery, an ileostomy may be created for several reasons. Firstly, it allows for the diversion of fecal matter from the lower part of the intestine or rectum, which may be affected by the cancer or the surgical procedure itself. By creating an opening in the abdominal wall, called a stoma, the small intestine (ileum) is brought to the surface, and waste is collected in a pouch attached to the stoma. This helps protect the healing site and allows the lower part of the gastrointestinal tract to rest and recover.

(2) Another reason for creating an ileostomy is to facilitate the removal or bypass of the affected section of the colon or rectum. In cases where the cancer has spread extensively or is causing obstruction, it may be necessary to remove a significant portion of the intestine. By diverting the flow of waste through the ileostomy, the surgeon can perform the necessary resection or bypass procedures more effectively and safely.

(3) Additionally, an ileostomy can help reduce the risk of infection. By bypassing the lower part of the large intestine, where stool is normally stored, the surgical site and remaining healthy portions of the bowel are protected from potential contamination. This can be particularly important when dealing with colorectal cancer, as the presence of fecal matter near the surgical site could increase the risk of complications and infection.

(4) The reversal procedure, also known as ileostomy closure or ileostomy takedown, can be performed after a period of healing and recovery, typically ranging from a few months to over a year, depending on individual circumstances. This delay allows the bowel and the surgical site to heal adequately and regain their functionality. Once the patient has sufficiently recovered, the ileostomy closure procedure can be performed to reconnect the small intestine to the remaining healthy part of the large intestine or rectum, restoring the normal passage of stool. The specific timing for the reversal procedure will depend on various factors, including the extent of the surgery, the overall health of the patient, and the recommendation of the medical team overseeing the case.

**12, What is the five-year survival rate after surgery for colorectal cancer?**

(1) Generally, the five-year survival rate after surgery for colorectal cancer is a commonly used measure to assess the long-term prognosis of patients. It represents the percentage of individuals who survive for at least five years after undergoing surgical treatment for colorectal cancer. This statistic is often considered a significant indicator of treatment success and overall patient outcomes.

(2) Another important point to note is that the five-year survival rate after surgery for colorectal cancer can vary based on several factors, including the stage of cancer at the time of diagnosis. In earlier stages, when the cancer is localized and has not spread to distant sites, the survival rates tend to be higher. However, if the cancer has already spread to nearby lymph nodes or other organs, the prognosis may be less favorable.

(3) Additionally, the five-year survival rate after surgery for colorectal cancer can be influenced by various patient-related factors, such as age, overall health, and the presence of other medical conditions. Younger patients with better overall health tend to have higher survival rates compared to older individuals or those with underlying health issues.

(4) It's important to highlight that the five-year survival rate after surgery for colorectal cancer is an average statistic and does not take into account individual variations. Each patient's prognosis may depend on a multitude of factors, including the specific characteristics of the tumor, response to treatment, and the effectiveness of follow-up care.

(5) Furthermore, advancements in surgical techniques, chemotherapy, radiation therapy, and targeted therapies have improved the survival rates for colorectal cancer patients over the years. Regular follow-up care, including surveillance for recurrence and screening for secondary cancers, can also contribute to better long-term outcomes.

Rep2:

(1) The five-year survival rate after surgery for colorectal cancer is generally influenced by several factors. These include the stage of cancer at the time of diagnosis, the extent of tumor spread, the patient's overall health, and the effectiveness of the surgical procedure in removing the cancerous tissues. The survival rate represents the percentage of people who are still alive five years after their initial surgery for colorectal cancer.

(2) Colorectal cancer is typically staged based on the extent of tumor growth, whether it has spread to nearby lymph nodes, and if it has metastasized to distant organs. Generally, patients with early-stage colorectal cancer, where the tumor is localized and has not spread, have higher survival rates after surgery. This is because surgery can often completely remove the tumor, and the chances of recurrence or metastasis are lower.

(3) The effectiveness of the surgical procedure in removing the cancerous tissues is an important factor in determining the five-year survival rate. In some cases, the surgery may be curative, meaning that all cancerous cells are successfully removed. However, in advanced cases or when the cancer has spread extensively, surgery may be part of a multimodal treatment approach that includes chemotherapy, radiation therapy, or targeted therapies to improve survival outcomes.

(4) The five-year survival rate for colorectal cancer after surgery varies depending on the stage of cancer. On average, the overall five-year survival rate for colorectal cancer after surgery is approximately 65-70%. However, it's important to note that survival rates can vary widely based on individual factors and the specific characteristics of the cancer. For example, patients with early-stage colorectal cancer (stage I) may have a five-year survival rate of around 90%, while those with advanced-stage cancer (stage IV) may have a lower survival rate, typically around 10-15%.

(5) It's crucial for patients diagnosed with colorectal cancer to discuss their individual prognosis and treatment options with their healthcare team. Factors such as age, overall health, genetic factors, and response to treatment can all influence survival outcomes. Regular follow-up care and adherence to recommended screenings and treatments can also play a significant role in improving long-term survival rates.

**13, Under what circumstances can the anus be preserved during rectal cancer surgery?**

(1) Preserving the anus during rectal cancer surgery is possible under certain circumstances. One such circumstance is when the tumor is located in the upper part of the rectum, closer to the sigmoid colon. In this case, the surgeon may be able to remove the tumor and a small portion of the rectum while still maintaining enough healthy rectal tissue to reconstruct and preserve the anus.

(2) Another circumstance where anus preservation may be possible is when the tumor is small in size and has not invaded the surrounding tissues extensively. If the tumor is localized and has not spread beyond the rectum, the surgeon may be able to perform a more conservative surgery, such as a local excision or a transanal resection, which involves removing only the tumor and a small margin of healthy tissue while leaving the rest of the rectum intact.

(3) Anus preservation may also be feasible when the tumor has responded well to preoperative treatments, such as radiation therapy or chemotherapy. These treatments can shrink the tumor and potentially downstage it, making it more amenable to conservative surgical approaches. When the tumor has responded favorably, the surgeon may have the opportunity to spare the anus and perform a less invasive surgery, such as a low anterior resection or a coloanal anastomosis.

(4) Additionally, advancements in surgical techniques and technologies, such as minimally invasive surgery and robotic-assisted surgery, have increased the possibilities for anus preservation during rectal cancer surgery. These techniques allow for more precise and meticulous dissection, enabling surgeons to spare the anus while effectively removing the cancerous tissue. However, the feasibility of preserving the anus ultimately depends on the specific characteristics of the tumor, its location, and the patient's individual circumstances, which should be carefully evaluated by the surgical team.

Rep2:

(1) Preserving the anus during rectal cancer surgery is possible under certain circumstances. One common scenario is when the tumor is located in the upper part of the rectum, closer to the sigmoid colon. In such cases, surgeons can perform a procedure called a low anterior resection, which involves removing the tumor and a portion of the rectum while preserving the anal sphincter muscles.

(2) Another circumstance where the anus can be preserved is when the tumor is small and localized within the rectal wall, without invading nearby structures or lymph nodes. In these cases, surgeons may opt for a local excision or transanal endoscopic microsurgery (TEM) to remove the tumor without the need for a major resection. This minimally invasive approach allows for preservation of the anus and better preservation of bowel function.

(3) Neoadjuvant therapy, such as radiation therapy or chemoradiotherapy, can also play a role in preserving the anus during rectal cancer surgery. By shrinking the tumor and reducing its involvement with surrounding tissues, neoadjuvant therapy can make it possible to perform a less extensive surgery while still achieving effective tumor control. This approach is commonly used for patients with locally advanced rectal cancer.

(4) In some cases, surgeons may employ techniques known as "intersphincteric resection" or "coloanal anastomosis" to preserve the anus. Intersphincteric resection involves removing the tumor and part of the lower rectum while preserving the internal and external sphincter muscles. Coloanal anastomosis refers to the surgical connection between the remaining rectum and the anal canal. These procedures are usually reserved for select cases where the tumor is very low in the rectum but does not invade the anal sphincters.

**14, If liver metastasis is detected at the time of colorectal cancer diagnosis, is there still a chance for surgery?**

(1) Yes, there is still a chance for surgery if liver metastasis is detected at the time of colorectal cancer diagnosis.

(2) The decision for surgery depends on several factors, including the extent of liver metastasis, the patient's overall health, and the possibility of removing both the primary colorectal tumor and the liver metastases.

(3) If the liver metastases are limited in number and size, and the primary colorectal tumor is resectable (removable), surgery may be considered as part of a multimodal treatment approach. This could involve removing the primary tumor in the colon or rectum, followed by surgical resection of the liver metastases.

(4) However, if the liver metastases are extensive, involving multiple lobes of the liver or other organs, or if the patient's overall health is poor, surgery may not be the best option. In such cases, alternative treatments such as chemotherapy, targeted therapy, radiation therapy, or a combination of these may be recommended to help control the disease and alleviate symptoms.

Rep2:

(1) Generally, if liver metastasis is detected at the time of colorectal cancer diagnosis, surgery may still be a viable option depending on certain factors. The possibility of surgical intervention depends on the extent of liver metastasis and whether it is limited to a few specific areas or has spread extensively throughout the liver.

(2) In cases where the liver metastasis is limited in size and number, and there are no signs of cancer spread beyond the liver, surgical resection may be considered. The aim of surgery in this scenario is to remove both the primary colorectal tumor and the liver metastases. This approach is known as potentially curative surgery and offers a chance for long-term survival.

(3) However, the decision for surgery also depends on the overall health of the patient and their ability to tolerate the procedure. Preoperative assessments are conducted to evaluate the patient's liver function, general fitness, and other potential factors that may affect their ability to undergo surgery safely. In some cases, additional treatments such as chemotherapy or targeted therapies may be recommended before surgery to shrink the liver metastases and improve the chances of successful resection.

(4) It is important to note that not all cases of liver metastasis can be treated with surgery. If the metastases are widespread, involving multiple areas of the liver or if the cancer has spread to other distant organs, surgery may not be feasible. In such situations, other treatment options like systemic chemotherapy, targeted therapy, or immunotherapy may be recommended to control the growth of the cancer, alleviate symptoms, and prolong survival.

**15,** **If lung metastasis is detected at the time of colorectal cancer diagnosis, is there still a chance for surgery?**

(1) Yes, there is still a chance for surgery if lung metastasis is detected at the time of colorectal cancer diagnosis.

(2) The treatment approach for colorectal cancer with lung metastasis depends on several factors, including the extent of metastasis, the patient's overall health, and the response to initial treatments.

(3) In some cases, if the lung metastasis is limited in number and size, and the patient's general health allows for surgery, surgical resection of the lung metastasis may be considered. This involves removing the metastatic tumors from the lung tissue.

(4) Additionally, surgery may be combined with other treatment modalities such as chemotherapy or targeted therapy to enhance the effectiveness of the treatment and improve the chances of long-term survival.

(5) However, it is important to note that the decision for surgery in the presence of lung metastasis is made on an individual basis, taking into account various factors, and is determined by the treating oncologist in collaboration with a multidisciplinary team.

(6) It is also crucial to consider that surgery for lung metastasis is generally more effective when the primary colorectal tumor has been successfully treated or is manageable, as controlling the primary tumor can help prevent further metastasis and improve overall outcomes.

(7) Ultimately, the prognosis and treatment options for colorectal cancer with lung metastasis vary from person to person, and it is essential to consult with an oncologist or a healthcare professional for personalized advice based on the specific circumstances and individual factors.

Rep2:

(1) If lung metastasis is detected at the time of colorectal cancer diagnosis, surgery may still be considered as a treatment option, depending on certain factors. The primary goal of surgery in this case would be to remove the primary colorectal tumor and any other visible metastases, including those in the lungs. However, the feasibility of surgery depends on the extent and location of the metastatic lesions in the lungs, as well as the overall health and condition of the patient.

(2) The decision to proceed with surgery will be based on a thorough evaluation by a multidisciplinary team, including surgeons, oncologists, radiologists, and other specialists. They will consider various factors such as the size and number of lung metastases, their location within the lungs, the presence of other distant metastases, the overall stage of the colorectal cancer, and the patient's overall health and fitness for surgery.

(3) In some cases, if the lung metastases are limited in number and confined to a specific region within the lungs, surgical resection may be possible. This typically involves removing the colorectal tumor along with the affected portion of the lung where the metastasis is located. The aim is to eliminate the sources of both the primary tumor and the metastatic lesions.

(4) However, it is important to note that surgery alone may not be curative for advanced colorectal cancer with lung metastasis. In most cases, surgery is combined with other treatment modalities such as chemotherapy, targeted therapy, immunotherapy, or radiation therapy. These additional treatments help target any remaining cancer cells in the body and reduce the risk of disease recurrence.

**16, What are the differences in surgical approach between patients with familial inherited colon cancer and those without?**

1. Patients with familial inherited colon cancer may require a more proactive surgical approach compared to those without a familial history of the disease. The primary reason for this is the increased risk of developing colorectal cancer in individuals with a family history of the condition. The presence of specific genetic mutations, such as those associated with Lynch syndrome or familial adenomatous polyposis (FAP), can significantly elevate the likelihood of developing colon cancer. Therefore, surgical interventions for these patients often focus on early detection, prevention, and aggressive management of the disease.

2. One key difference lies in the timing of surgical interventions. Patients with familial inherited colon cancer may undergo prophylactic or preventive surgeries to reduce the risk of developing colorectal cancer. For example, individuals with FAP, which is characterized by the development of numerous polyps in the colon, often undergo a total colectomy with ileorectal anastomosis or proctocolectomy with ileal pouch-anal anastomosis to prevent the progression to cancer. In contrast, patients without familial inherited colon cancer typically undergo surgical procedures in response to the presence of cancerous tumors or precancerous lesions detected through regular screening.

3. The extent of surgery may also differ between the two patient groups. In familial inherited colon cancer, particularly in cases of Lynch syndrome, which is caused by mutations in DNA mismatch repair genes, the presence of synchronous or metachronous colon cancers is more common. Therefore, surgical interventions may involve removing not only the primary tumor but also additional segments of the colon to address multiple lesions or prevent future malignancies. In contrast, patients without a family history of colon cancer often undergo surgery that is limited to the affected portion of the colon or rectum.

4. In addition to the differences in surgical approach, patients with familial inherited colon cancer often require close surveillance and regular follow-up after surgery. Given the increased risk of developing subsequent tumors, these individuals may undergo frequent colonoscopies and other diagnostic tests to detect any new polyps or cancerous lesions at an early stage. This ongoing monitoring helps to ensure timely intervention and appropriate management.

Rep2:

(1) Patients with familial inherited colon cancer often require more aggressive surgical approaches compared to those without the condition. This is primarily due to the increased risk of developing multiple polyps or tumors in the colon and rectum. In these cases, the surgical goal is to remove all the affected tissue to minimize the risk of cancer progression or recurrence.

(2) One common surgical approach for familial inherited colon cancer is total colectomy with ileorectal anastomosis. This procedure involves removing the entire colon while preserving the rectum and connecting the small intestine (ileum) to the rectum. It is typically performed when the risk of developing new polyps or tumors in the remaining colon is relatively low.

(3) In cases where there is a high risk of developing new polyps or tumors even in the rectum, a total proctocolectomy with ileal pouch-anal anastomosis (IPAA) may be considered. This procedure involves removing both the colon and the rectum and creating a pouch from the small intestine, which is then connected to the anus. The pouch serves as a reservoir for storing and passing stool.

(4) Additionally, patients with familial inherited colon cancer may undergo more frequent surveillance colonoscopies after surgery to monitor for the development of new polyps or tumors. These regular screenings help in early detection and prompt intervention, if necessary, to prevent the progression of cancer.

(5) It's important to note that the specific surgical approach for patients with familial inherited colon cancer may vary depending on factors such as the extent of polyp burden, individual risk factors, and the recommendations of the medical team. A thorough assessment and personalized treatment plan are crucial in addressing the unique needs of each patient.

**The Internal Medicine**

**1,** **Does rectal cancer require preoperative radiotherapy? Are there differences between rectal cancer and colon cancer regarding radiotherapy?**

(1) Yes, preoperative radiotherapy is commonly used in the treatment of rectal cancer. It is an integral part of the multimodal approach to rectal cancer management, which typically includes a combination of surgery, radiation therapy, and chemotherapy. Preoperative radiotherapy, also known as neoadjuvant radiotherapy, involves delivering radiation to the tumor site before surgery. This treatment approach offers several benefits.

(2) Rectal cancer and colon cancer are two different types of malignancies that arise in different parts of the large intestine. While both rectal and colon cancer can be treated with radiotherapy, there are some differences in their management. In rectal cancer, preoperative radiotherapy is often used as a standard treatment option to reduce tumor size, increase the chances of complete surgical resection, and improve local control of the disease. On the other hand, colon cancer is primarily treated with surgery, and adjuvant radiotherapy (radiotherapy given after surgery) is typically reserved for certain high-risk cases, such as those with advanced tumors or lymph node involvement.

(3) The rationale behind using preoperative radiotherapy in rectal cancer is to downstage the tumor, shrink its size, and facilitate a more complete surgical resection. By administering radiation therapy before surgery, the tumor can be targeted directly, potentially reducing the risk of local recurrence and improving the chances of achieving negative surgical margins. Preoperative radiotherapy has been shown to improve local control, reduce the need for radical surgery (such as abdominoperineal resection), and preserve sphincter function in selected patients.

(4) In addition to improving local control and facilitating surgery, preoperative radiotherapy in rectal cancer has been associated with a reduced risk of distant metastases and improved overall survival rates. The combination of radiotherapy with chemotherapy, known as chemoradiotherapy, is often used to enhance the effectiveness of treatment. However, it is important to note that the decision to recommend preoperative radiotherapy in rectal cancer is based on several factors, including the stage of the disease, tumor location, and individual patient characteristics. It is best to consult with a multidisciplinary team of healthcare professionals to determine the most appropriate treatment approach for each specific case.

Rep2:

(1) Yes, preoperative radiotherapy is commonly used in the treatment of rectal cancer. The main goal of preoperative radiotherapy is to shrink the tumor and potentially increase the chances of complete surgical resection. By reducing the tumor size, preoperative radiotherapy can help improve the surgical outcome and minimize the risk of local recurrence. It is typically administered in combination with chemotherapy, a treatment approach known as chemoradiotherapy. The specific radiation dosage, timing, and duration of treatment may vary based on individual patient factors and the stage of the rectal cancer.

(2) Rectal cancer and colon cancer differ in terms of their anatomical location within the digestive system. Rectal cancer develops in the last several inches of the large intestine, while colon cancer affects the colon, which is the upper portion of the large intestine. Due to the proximity of the rectum to vital structures, such as the sphincter muscles and the pelvic organs, the treatment approach for rectal cancer, including the use of radiotherapy, is often more complex and tailored compared to colon cancer.

(3) Preoperative radiotherapy is less commonly used in the treatment of colon cancer compared to rectal cancer. This is primarily because the surgical resection of colon cancer is relatively straightforward and does not involve the challenges associated with preserving sphincter function or minimizing damage to nearby organs. In some cases of advanced or locally recurrent colon cancer where surgery alone may not be sufficient, radiotherapy may be considered as a palliative treatment to relieve symptoms or as a part of a multidisciplinary approach.

(4) The decision to use preoperative radiotherapy for rectal cancer is based on several factors, including the stage of the cancer, the location of the tumor within the rectum, and individual patient considerations. While preoperative radiotherapy has been shown to offer benefits in terms of tumor shrinkage and improved surgical outcomes for rectal cancer, it is not universally prescribed for all patients. The treatment plan is determined through a multidisciplinary approach involving a team of oncologists, surgeons, and other specialists who carefully evaluate the individual case and weigh the potential benefits against the risks and side effects of radiotherapy.

**2, Can targeted therapy be used after surgical resection of colorectal cancer?**

(1) Yes, targeted therapy can be used after surgical resection of colorectal cancer. Targeted therapies are a type of cancer treatment that specifically targets the cancer cells based on their unique molecular characteristics. These therapies work by blocking specific molecules or pathways involved in the growth and spread of cancer cells.

After surgical resection of colorectal cancer, targeted therapy can be used to reduce the risk of cancer recurrence or to treat any remaining cancer cells that may not have been completely removed during surgery. It can also be used as an adjuvant therapy, which means it is given in addition to surgery to improve treatment outcomes.

One commonly used targeted therapy for colorectal cancer is anti-EGFR (epidermal growth factor receptor) therapy. It targets the EGFR protein, which is often overexpressed in colorectal cancer cells. Other targeted therapies may include drugs that inhibit the vascular endothelial growth factor (VEGF) pathway or those that target specific genetic mutations found in colorectal cancer cells.

(2) Targeted therapy after surgical resection of colorectal cancer can help to improve patient outcomes by targeting specific molecular abnormalities associated with the cancer cells. By specifically targeting these molecular abnormalities, targeted therapies can potentially be more effective and have fewer side effects compared to traditional chemotherapy.

In some cases, targeted therapy may be used before surgery (neoadjuvant therapy) to shrink tumors and increase the chances of successful surgical removal. After surgery, targeted therapy can be used as adjuvant therapy to target any remaining cancer cells or micrometastases that may not be visible on imaging scans.

(3) Targeted therapy has revolutionized the treatment of colorectal cancer by offering more precise and personalized treatment options. It allows oncologists to tailor treatment strategies based on the specific molecular characteristics of each patient's tumor. This approach has the potential to improve treatment outcomes and reduce the likelihood of cancer recurrence.

In the context of surgical resection of colorectal cancer, targeted therapy can complement surgery by addressing any residual disease or micrometastases that may be present. It can also be used in combination with other treatments, such as chemotherapy or radiation therapy, to provide a comprehensive approach to cancer treatment.

The effectiveness of targeted therapy after surgical resection of colorectal cancer may vary depending on the specific molecular abnormalities present in the tumor cells. Therefore, it is crucial to perform molecular profiling of the tumor tissue to identify potential targets for treatment and select the most appropriate targeted therapy.

Overall, targeted therapy has emerged as a valuable treatment option for colorectal cancer patients, both in the preoperative and postoperative settings. It has the potential to improve patient outcomes and contribute to a more personalized approach to cancer treatment.

(4) The decision to use targeted therapy after surgical resection of colorectal cancer is influenced by various factors, including the stage of the cancer, the presence of specific molecular abnormalities, and the overall health of the patient. In some cases, targeted therapy may be recommended as part of a multimodal treatment approach to improve the chances of long-term disease control.

Rep2:

(1) Yes, targeted therapy can be used after surgical resection of colorectal cancer.

(2) After surgical resection, targeted therapy can be utilized to specifically target and inhibit certain molecular pathways or genetic mutations that are driving the growth and spread of colorectal cancer cells. This type of therapy aims to block the signals that promote cancer cell growth and survival, ultimately preventing the recurrence or progression of the disease.

(3) Targeted therapies for colorectal cancer can include monoclonal antibodies, small molecule inhibitors, and immunotherapies. Monoclonal antibodies such as cetuximab and panitumumab target the epidermal growth factor receptor (EGFR), which is often overexpressed in colorectal cancer cells. By binding to EGFR, these antibodies inhibit the signaling pathways that drive cancer growth. Small molecule inhibitors, such as regorafenib and cetuximab, target specific molecules involved in cancer cell signaling or blood vessel formation, further hindering tumor growth. Immunotherapies, such as immune checkpoint inhibitors, can also be used to enhance the body's immune response against cancer cells.

(4) The use of targeted therapy after surgical resection of colorectal cancer is generally determined based on the specific characteristics of the tumor and the patient. Molecular testing is often conducted to identify genetic mutations or molecular markers that can guide the selection of targeted therapies. The decision to administer targeted therapy will depend on factors such as the stage of the cancer, the presence of specific mutations or biomarkers, and the overall health of the patient.

**3, Is chemotherapy needed after colorectal cancer surgery?**

(1) In certain cases, chemotherapy may be recommended after colorectal cancer surgery. After surgery, the primary goal is to eliminate any remaining cancer cells that may be present in the body. Chemotherapy is a systemic treatment that uses drugs to target and kill cancer cells throughout the body. It can be given before or after surgery, depending on various factors such as the stage of cancer, the extent of tumor spread, and the overall health of the patient. Postoperative chemotherapy, known as adjuvant chemotherapy, is typically recommended for patients with a higher risk of cancer recurrence to help reduce that risk and improve long-term outcomes.

(2) The decision to administer chemotherapy after colorectal cancer surgery is based on several factors. One important consideration is the stage of the cancer, which helps determine the likelihood of cancer cells remaining in the body after surgery. If the cancer has spread to nearby lymph nodes or other distant organs, there is a higher risk of recurrence, and chemotherapy may be recommended to target any residual cancer cells. Additionally, certain tumor characteristics, such as aggressive features or the presence of specific genetic mutations, may also influence the decision to recommend postoperative chemotherapy. Ultimately, the decision is made on an individual basis, taking into account the patient's specific circumstances and treatment goals.

(3) Postoperative chemotherapy following colorectal cancer surgery aims to improve the chances of long-term survival and prevent cancer recurrence. Even if the surgeon successfully removes the tumor during surgery, there is still a risk of microscopic cancer cells remaining in the body. Chemotherapy helps eradicate these cells, reducing the likelihood of the cancer returning. Studies have shown that adjuvant chemotherapy can significantly improve survival rates and reduce the risk of recurrence in certain cases. However, the specific chemotherapy regimen, duration, and potential side effects will depend on the patient's overall health, the stage of the cancer, and other individual factors. The decision to proceed with chemotherapy should be discussed thoroughly with the medical team to weigh the potential benefits and risks.

(4) Whether chemotherapy is needed after colorectal cancer surgery depends on multiple factors, including the stage of the cancer, the presence of any lymph node involvement, and individual patient characteristics. In some cases, particularly for early-stage cancers with a low risk of recurrence, surgery alone may be curative, and chemotherapy may not be necessary. However, for patients with more advanced tumors or a higher risk of cancer recurrence, adjuvant chemotherapy is often recommended to help reduce the risk of relapse and improve overall outcomes. The decision should be made in consultation with a multidisciplinary team of healthcare professionals, including surgeons, oncologists, and other specialists, who will assess the specific situation and tailor the treatment plan to the individual patient.

Rep2:

1) Chemotherapy is often recommended after colorectal cancer surgery as part of the overall treatment plan. It is used to kill any remaining cancer cells that may not have been removed during surgery and to reduce the risk of cancer recurrence. Chemotherapy can target cancer cells throughout the body and is typically administered in cycles over a period of several months. The specific chemotherapy drugs and duration of treatment may vary depending on factors such as the stage and extent of the cancer, the patient's overall health, and any additional treatments that may be recommended.

2) While surgery aims to remove the tumor and surrounding affected tissue, chemotherapy may be necessary to address any cancer cells that have spread to other parts of the body or are too small to be detected through imaging or surgical procedures alone. Colorectal cancer can metastasize, meaning it can spread to distant organs or lymph nodes. Chemotherapy is designed to target these potential cancer cells and prevent their growth or recurrence. The decision to undergo chemotherapy after surgery is often based on various factors, including the stage of the cancer and the risk of recurrence.

3) The use of chemotherapy after colorectal cancer surgery is based on the individual patient's circumstances and the recommendations of their healthcare team. In some cases, depending on the stage and extent of the cancer, chemotherapy may be considered a standard part of the treatment plan after surgery. It helps to minimize the risk of cancer recurrence and improves overall survival rates. However, not all patients will require chemotherapy after surgery, especially if the cancer is detected at an early stage and has not spread beyond the colon or rectum. The final decision regarding chemotherapy should be made in consultation with the oncologist or healthcare provider, taking into account the patient's specific situation and medical history.

4) The decision to undergo chemotherapy after colorectal cancer surgery depends on various factors, including the stage of the cancer, the presence of any lymph node involvement, and the risk of recurrence. Adjuvant chemotherapy, which is given after surgery, is often recommended for patients with higher-risk features, such as advanced-stage tumors or lymph node involvement. It helps to target any microscopic cancer cells that may remain in the body and reduce the risk of cancer coming back. However, for patients with early-stage, low-risk tumors that have been completely removed during surgery, adjuvant chemotherapy may not be necessary. The oncologist will evaluate each case individually and provide personalized recommendations based on the specific characteristics of the tumor and the patient's overall health.

**4, How to choose a chemotherapy regimen after colorectal cancer surgery?**

(1) Considerations for choosing a chemotherapy regimen after colorectal cancer surgery involve various factors such as the stage of the cancer, the patient's overall health, and any specific molecular or genetic characteristics of the tumor. The goal of chemotherapy in this context is typically to eliminate any remaining cancer cells, reduce the risk of cancer recurrence, and improve overall survival rates.

(2) One common approach is to classify colorectal cancer into different stages: Stage I, II, III, or IV, based on factors such as tumor size, lymph node involvement, and presence of distant metastasis. For early-stage colorectal cancer (Stage I or II), adjuvant chemotherapy may not be necessary, as surgery alone may be curative. However, for Stage III colorectal cancer, which has spread to nearby lymph nodes but not to distant sites, adjuvant chemotherapy is often recommended to reduce the risk of recurrence. The specific chemotherapy regimen for Stage III colorectal cancer may involve a combination of drugs like fluorouracil (5-FU) with oxaliplatin (FOLFOX) or capecitabine with oxaliplatin (CapeOx).

(3) In the case of advanced or metastatic colorectal cancer (Stage IV), where the cancer has spread to distant sites such as the liver or lungs, systemic chemotherapy is typically used as the primary treatment. The choice of chemotherapy regimen in this scenario may depend on several factors, including the patient's performance status, any prior treatment history, and the specific molecular characteristics of the tumor. Common chemotherapy regimens for metastatic colorectal cancer include FOLFOX, CapeOx, or FOLFIRI (fluorouracil with irinotecan). Additionally, targeted therapies such as anti-EGFR antibodies (cetuximab or panitumumab) or anti-VEGF antibodies (bevacizumab or ramucirumab) may be combined with chemotherapy to enhance treatment effectiveness.

(4) It is important to note that the selection of a chemotherapy regimen should always be individualized and decided in consultation with a multidisciplinary team, including oncologists, surgeons, and pathologists. Factors such as potential side effects, patient preferences, and comorbidities should also be taken into account when making treatment decisions. Regular monitoring and follow-up are crucial to assess the response to chemotherapy and make any necessary adjustments to the treatment plan.

Rep2:

(1) Consideration of the stage and characteristics of the colorectal cancer, as well as the patient's overall health, are crucial factors when choosing a chemotherapy regimen after colorectal cancer surgery. Typically, the decision is made by a multidisciplinary team involving medical oncologists, surgeons, and other specialists. The main goals of post-operative chemotherapy are to eradicate any remaining cancer cells and reduce the risk of cancer recurrence.

(2) The stage of colorectal cancer is an essential determinant in selecting a chemotherapy regimen. For early-stage colon cancer (Stage II), chemotherapy may not be routinely recommended unless there are high-risk features present. However, for locally advanced (Stage III) or metastatic (Stage IV) colorectal cancer, chemotherapy is typically recommended after surgery to improve survival rates.

(3) The characteristics of the tumor also play a role in chemotherapy selection. The presence of specific genetic mutations, such as KRAS or BRAF mutations, can guide treatment decisions. For example, patients with KRAS wild-type tumors may benefit from targeted therapies like anti-EGFR (epidermal growth factor receptor) agents, while those with BRAF mutations may require alternative treatment approaches.

(4) The patient's overall health and individual factors, such as age, comorbidities, and treatment tolerance, should be considered when choosing a chemotherapy regimen. Some regimens have more significant side effects and may not be suitable for patients with certain health conditions. The goal is to balance the potential benefits of treatment with the patient's ability to tolerate and complete the recommended chemotherapy.

**5, How many cycles of chemotherapy are needed after colorectal cancer surgery?**

(1) The number of cycles of chemotherapy needed after colorectal cancer surgery can vary depending on several factors, including the stage of the cancer, the individual's overall health, and the specific treatment plan recommended by the oncologist. In general, chemotherapy is often given after surgery as a form of adjuvant therapy to destroy any remaining cancer cells and reduce the risk of cancer recurrence. The number of cycles typically ranges from 3 to 6, with each cycle usually lasting a few weeks. The exact duration and frequency of chemotherapy cycles will be determined by the medical team based on the individual's specific circumstances.

(2) The decision regarding the number of cycles of chemotherapy following colorectal cancer surgery is typically based on the stage of the cancer. For early-stage colorectal cancer (stages I and II), adjuvant chemotherapy may not be necessary, and surgery alone may be sufficient. However, for more advanced stages (III and IV) or when cancer has spread to nearby lymph nodes, chemotherapy is often recommended to target any remaining cancer cells and prevent their regrowth. The number of cycles may vary, but it is typically between 3 and 6, administered over a period of several months.

(3) After undergoing surgery for colorectal cancer, patients may be advised to undergo chemotherapy to improve their chances of long-term survival. The number of chemotherapy cycles required can depend on multiple factors, such as the aggressiveness of the cancer, the presence of any metastasis, and the overall health of the patient. Chemotherapy is commonly given in cycles, with each cycle consisting of a treatment period followed by a recovery period. The duration and frequency of cycles can vary, but typically they are administered every 2 to 3 weeks. The total number of cycles may range from 3 to 6, but the specific recommendation will be determined by the oncologist based on the individual's case.

(4) The number of chemotherapy cycles after colorectal cancer surgery varies and is influenced by individual factors. These may include the stage of the cancer, the presence of metastasis, the patient's overall health, and the treatment plan recommended by the oncologist. Chemotherapy is often used as an adjuvant therapy to eliminate any remaining cancer cells after surgery and reduce the risk of recurrence. The specific number of cycles can range from 3 to 6, with each cycle typically lasting a few weeks. The oncologist will evaluate the patient's condition and consider various factors to determine the appropriate number of cycles needed for optimal treatment outcome.

Rep2:

(1) The number of cycles of chemotherapy required after colorectal cancer surgery can vary depending on several factors, including the stage of the cancer, the individual's overall health, and the specific treatment plan recommended by the medical team. In general, adjuvant chemotherapy is often recommended after surgery for colorectal cancer to help reduce the risk of cancer recurrence. Adjuvant chemotherapy involves administering anticancer drugs to eliminate any remaining cancer cells that may not be visible or have the potential to spread. The number of cycles typically ranges from 3 to 6, with each cycle lasting several weeks. The specific chemotherapy drugs and the interval between cycles may also vary based on individual circumstances.

(2) Following colorectal cancer surgery, the decision regarding the number of chemotherapy cycles is typically based on the cancer's stage and the risk of recurrence. If the cancer has spread beyond the primary tumor or if there is a high risk of recurrence, a longer course of chemotherapy may be recommended. Conversely, if the cancer is at an early stage and the risk of recurrence is lower, a shorter course of chemotherapy may be sufficient. The medical team will consider various factors, such as the patient's overall health, tolerance to chemotherapy, and potential side effects when determining the appropriate duration of treatment.

(3) The number of cycles of chemotherapy administered after colorectal cancer surgery is influenced by individualized treatment plans. It is not a one-size-fits-all approach. The medical team considers multiple factors, including the cancer stage, the presence of any metastasis, and the patient's overall health condition. The goal of chemotherapy in this setting is to eradicate any remaining cancer cells that might have escaped surgical removal. Typically, chemotherapy cycles are given over a specified duration, often lasting several months. The specific drugs used, the frequency of administration, and the duration of each cycle depend on the patient's specific circumstances and the recommendations of the oncologist.

(4) The determination of the number of chemotherapy cycles after colorectal cancer surgery is a complex decision that depends on various factors. These factors include the stage of the cancer, whether it has spread to lymph nodes or distant sites, the overall health of the patient, and the goals of treatment. In general, the medical team aims to strike a balance between reducing the risk of cancer recurrence and managing potential side effects of chemotherapy. The number of cycles typically ranges from 3 to 6, but it can be higher or lower depending on individual circumstances. It is important for patients to have open and thorough discussions with their healthcare team to understand their specific treatment plan and the rationale behind it.

**6, How to alleviate nausea and vomiting caused by chemotherapy?**

(1) One way to alleviate nausea and vomiting caused by chemotherapy is by using antiemetic medications. These medications work by blocking the signals in the brain that trigger nausea and vomiting. They can be prescribed by your healthcare provider and are available in different forms such as tablets, injections, or patches. Antiemetics are often given before chemotherapy sessions to prevent or minimize the occurrence of these side effects. It is important to follow your healthcare provider's instructions and take the medications as directed.

(2) Another approach to alleviate chemotherapy-induced nausea and vomiting is by making dietary and lifestyle changes. Eating small, frequent meals throughout the day instead of large meals can help prevent feelings of fullness and reduce the likelihood of nausea. Choosing bland, easy-to-digest foods like crackers, toast, or broth can be helpful. Avoiding greasy, spicy, or strong-smelling foods that may trigger nausea is also recommended. Drinking plenty of fluids and staying hydrated is important, but it's best to sip fluids slowly rather than drinking large amounts at once. Additionally, getting plenty of rest, managing stress, and practicing relaxation techniques like deep breathing or meditation can contribute to reducing nausea and vomiting.

(3) Acupuncture is a complementary therapy that can be considered to alleviate chemotherapy-induced nausea and vomiting. This traditional Chinese practice involves the insertion of thin needles into specific points on the body. Studies have shown that acupuncture can help reduce chemotherapy-related nausea and vomiting when performed by a trained acupuncturist. It is believed to work by stimulating the release of natural pain-relieving chemicals in the body and regulating the body's energy flow. It is important to consult with your healthcare provider before starting acupuncture to ensure its suitability and safety for your specific situation.

(4) Another option to alleviate nausea and vomiting caused by chemotherapy is through the use of natural remedies. Ginger has been found to be effective in reducing nausea, and it can be consumed in various forms such as ginger tea, ginger candies, or ginger capsules. Peppermint and chamomile teas may also help soothe the stomach. Some individuals find relief by using aromatherapy with essential oils like peppermint or lemon. However, it is crucial to discuss the use of natural remedies with your healthcare provider as they may interact with other medications or have potential side effects.

Rep2:

(1) One way to alleviate nausea and vomiting caused by chemotherapy is through the use of antiemetic medications. These medications work by blocking the signals in the brain that trigger nausea and vomiting. There are different types of antiemetics available, including serotonin receptor antagonists, dopamine receptor antagonists, corticosteroids, and NK1 receptor antagonists. Your doctor will prescribe the appropriate medication based on the specific chemotherapy drugs being used and your individual needs.

(2) Another approach to managing chemotherapy-induced nausea and vomiting is through lifestyle and dietary changes. It is important to eat small, frequent meals rather than large meals, as large meals can contribute to feelings of nausea. Choosing bland and easily digestible foods can also help. It may be beneficial to avoid foods with strong smells or flavors that may trigger nausea. Staying hydrated by drinking small amounts of fluids throughout the day can also help reduce nausea. Some individuals find relief from alternative therapies such as acupuncture or acupressure, although it is important to consult with your healthcare team before trying any complementary treatments.

(3) Relaxation techniques and stress reduction can be helpful in alleviating chemotherapy-induced nausea and vomiting. Stress and anxiety can exacerbate these symptoms, so finding ways to relax and manage stress levels may be beneficial. Deep breathing exercises, meditation, guided imagery, and progressive muscle relaxation are some techniques that can help promote relaxation and reduce nausea and vomiting. Engaging in activities that distract your mind, such as listening to music, watching movies, or reading, can also help take your focus away from the discomfort.

(4) Some patients may find relief from nausea and vomiting by trying complementary therapies such as ginger. Ginger has been used for centuries to alleviate digestive issues, including nausea. It can be consumed in various forms, such as ginger tea, ginger candies, or ginger capsules. However, it's important to consult with your healthcare team before trying any supplements or herbal remedies to ensure they do not interfere with your treatment or interact with any other medications you may be taking. Your healthcare team can provide personalized recommendations based on your specific situation.

(5) In some cases, your doctor may recommend a different chemotherapy regimen or adjust the dosage or administration schedule to help minimize nausea and vomiting. If you are experiencing severe or persistent symptoms, it is important to communicate with your healthcare team so they can provide appropriate support and make necessary adjustments to your treatment plan. They may be able to provide additional interventions or medications to help manage your symptoms effectively.

**7, Will chemotherapy drugs for colon cancer cause hair loss?**

(1) Yes, chemotherapy drugs used to treat colon cancer can cause hair loss. Chemotherapy works by targeting rapidly dividing cells in the body, including cancer cells. Unfortunately, it also affects other rapidly dividing cells, such as those in hair follicles. This can result in hair loss or thinning during treatment. Hair loss due to chemotherapy is usually temporary, and hair typically begins to grow back once treatment is completed.

(2) Hair loss is a common side effect of many chemotherapy drugs used in the treatment of colon cancer. The specific drugs and regimens prescribed may vary depending on the individual's condition and treatment plan. Some chemotherapy drugs commonly associated with hair loss include fluorouracil (5-FU), oxaliplatin, irinotecan, capecitabine, and others. These drugs can cause hair follicles to become inactive, leading to hair loss.

(3) Hair loss from chemotherapy for colon cancer is not limited to the scalp; it can affect hair all over the body, including eyebrows, eyelashes, facial hair, and body hair. The extent of hair loss varies from person to person, and some individuals may experience complete hair loss, while others may only have partial hair thinning. Hair loss usually starts within a few weeks of starting chemotherapy and may continue throughout the treatment period.

(4) The impact of chemotherapy on hair loss can be emotionally distressing for many individuals. Understanding that hair loss is a temporary side effect can help cope with this aspect of treatment. There are various options to manage hair loss, such as wearing wigs, scarves, or hats to cover the scalp. Additionally, some individuals choose to embrace their baldness during treatment. It is important to discuss any concerns about hair loss with the healthcare team, as they can provide guidance and support during this time.

Rep2:

(1) Yes, chemotherapy drugs used to treat colon cancer can cause hair loss. Chemotherapy drugs are designed to kill rapidly dividing cells, which includes not only cancer cells but also healthy cells in the body. Hair follicles are made up of rapidly dividing cells, and when chemotherapy drugs affect these cells, it can lead to hair loss. The extent of hair loss can vary from person to person and may range from mild thinning to complete baldness.

(2) Hair loss, also known as alopecia, is a common side effect of chemotherapy drugs used in colon cancer treatment. The specific chemotherapy drugs and dosage used can influence the likelihood and severity of hair loss. Some chemotherapy drugs are more likely to cause hair loss than others. Hair loss typically begins a few weeks after starting chemotherapy and may continue throughout the treatment period.

(3) Chemotherapy drugs for colon cancer can affect hair growth because they target rapidly dividing cells, including those in the hair follicles. This can lead to hair loss on the scalp, as well as other body hair such as eyebrows, eyelashes, and body hair. However, it's important to note that not all chemotherapy drugs cause hair loss, and the likelihood and extent of hair loss can vary from person to person.

(4) Hair loss is a potential side effect of chemotherapy drugs used to treat colon cancer. While not all chemotherapy drugs cause hair loss, many commonly used drugs have the potential to affect hair follicles and result in hair thinning or complete hair loss. The decision to use chemotherapy and the specific drugs chosen depend on various factors, including the stage and aggressiveness of the colon cancer, overall health of the patient, and individual treatment goals. If hair loss occurs, it is usually temporary, and hair typically starts to regrow once chemotherapy treatment is completed.

**8, What to do if constipation occurs during and after chemotherapy?**

(1) Increase fiber intake: Consuming foods high in fiber can help promote regular bowel movements and relieve constipation. Include foods such as fruits, vegetables, whole grains, and legumes in your diet. Additionally, you can try adding fiber supplements like psyllium husk or methylcellulose to your meals.

(2) Stay hydrated: Drinking plenty of fluids is important for preventing constipation. Aim to drink at least 8 cups of water or other non-caffeinated beverages throughout the day. Adequate hydration helps soften the stool and promotes easier passage through the digestive system.

(3) Engage in physical activity: Regular exercise can stimulate bowel movements and help alleviate constipation. Talk to your healthcare team about appropriate exercises or physical activities that you can safely perform during and after chemotherapy. Even light activities like walking or gentle stretching can be beneficial.

(4) Consider stool softeners or laxatives: If dietary changes and increased fluid intake don't provide relief, your doctor may recommend over-the-counter stool softeners or laxatives. These medications can help soften the stool and facilitate bowel movements. However, it's important to follow your doctor's instructions and not rely on laxatives as a long-term solution without medical supervision.

(5) Communicate with your healthcare team: If constipation persists or becomes severe, it's crucial to inform your healthcare team. They can evaluate your situation and provide further guidance or prescribe medications specifically tailored to your needs. They may also assess if constipation is a side effect of your chemotherapy treatment and suggest alternative options if necessary.

(6) Maintain a healthy lifestyle: Overall, maintaining a healthy lifestyle is essential for managing constipation during and after chemotherapy. This includes eating a balanced diet, staying physically active, managing stress levels, and getting enough rest. Remember to follow any specific recommendations given by your healthcare team to ensure the best possible care for your condition.

Rep2:

(1) One possible approach to managing constipation during and after chemotherapy is to make dietary modifications. Increasing fiber intake can help soften the stool and promote regular bowel movements. Foods rich in fiber include fruits, vegetables, whole grains, and legumes. Additionally, staying hydrated by drinking plenty of fluids, particularly water, can aid in preventing constipation. It is important to gradually increase fiber intake and maintain a balanced diet to avoid any digestive discomfort.

(2) Another strategy is to engage in regular physical activity. Exercise can stimulate the muscles in the intestines and promote bowel regularity. Simple activities like walking, swimming, or light aerobic exercises can be beneficial. However, it is essential to consult with a healthcare professional before starting any exercise program during or after chemotherapy, as individual circumstances may vary.

(3) Medications may be prescribed to relieve constipation during chemotherapy. Laxatives, such as stool softeners or osmotic laxatives, can help soften the stool and ease bowel movements. However, it is crucial to consult with a healthcare provider before using any over-the-counter laxatives or starting new medications, as they may interact with ongoing chemotherapy treatments or other medications.

(4) Creating a consistent bowel routine can also be helpful. Establishing regular times for bowel movements, ideally following meals, can train the body to have predictable and regular bowel habits. It can be beneficial to find a comfortable and relaxed environment for bowel movements to encourage a healthy elimination process.

**9, What to do if diarrhea occurs during and after chemotherapy?**

(1) Diarrhea is a common side effect of chemotherapy that can occur during and after treatment. Here are a few general steps to take if you experience diarrhea during and after chemotherapy:

- Stay hydrated: Diarrhea can lead to dehydration, so it's important to drink plenty of fluids. Water, clear broths, herbal teas, and sports drinks can help replenish lost fluids and electrolytes. Avoid caffeine, alcohol, and sugary drinks as they can worsen diarrhea.

- Follow a BRAT diet: The BRAT diet consists of bananas, rice, applesauce, and toast. These foods are easy to digest and can help firm up stools. Gradually reintroduce other bland, low-fiber foods as your symptoms improve.

- Take over-the-counter medications: Anti-diarrheal medications such as loperamide (Imodium) can help control diarrhea. However, it's important to consult your healthcare team before taking any medications, as they may interact with your chemotherapy drugs or affect your treatment.

(2) Communicate with your healthcare team: It's crucial to inform your healthcare team about your diarrhea symptoms. They can assess the severity of the diarrhea and provide specific recommendations or prescribe medications to manage it. They may also want to evaluate if any changes are needed in your chemotherapy treatment plan.

- Monitor your symptoms: Keep track of the frequency, consistency, and severity of your diarrhea. This information will help your healthcare team determine the appropriate course of action and identify any patterns or triggers.

- Maintain good hygiene: Diarrhea can increase the risk of infections. Wash your hands thoroughly and frequently, especially after using the bathroom, to reduce the risk of spreading bacteria or viruses.

(3) Dietary considerations: Making certain dietary modifications can help alleviate diarrhea during and after chemotherapy:

- Avoid trigger foods: Certain foods and beverages can exacerbate diarrhea. These may include spicy or greasy foods, high-fiber foods (such as raw fruits and vegetables), dairy products, caffeine, and alcohol. Identify any specific triggers and avoid them.

- Eat small, frequent meals: Instead of consuming large meals, try eating smaller, more frequent meals throughout the day. This approach can be easier on your digestive system and help regulate bowel movements.

- Consider probiotics: Probiotics are beneficial bacteria that can support gut health. Talk to your healthcare team about whether probiotics might be beneficial for you during chemotherapy-induced diarrhea.

- Seek guidance from a registered dietitian: A registered dietitian with experience in oncology can provide personalized dietary recommendations to manage diarrhea and maintain proper nutrition during chemotherapy.

Rep2:

(1) Diarrhea during and after chemotherapy is a common side effect that can be managed effectively. Here are some general steps to take if diarrhea occurs:

- Stay hydrated: Diarrhea can cause dehydration, so it's crucial to drink plenty of fluids. Opt for water, clear broths, herbal tea, and electrolyte-rich drinks to replenish lost fluids and prevent dehydration.

- Follow a bland diet: Avoid foods that can irritate the digestive system, such as spicy, greasy, and high-fiber foods. Stick to a bland diet that includes easily digestible foods like rice, toast, cooked vegetables, and lean proteins.

- Avoid caffeine and alcohol: These substances can worsen diarrhea and contribute to dehydration. It's best to steer clear of caffeinated beverages and alcohol until the diarrhea subsides.

- Try over-the-counter medications: Speak with your healthcare provider about using over-the-counter antidiarrheal medications, such as loperamide (Imodium), to help control diarrhea. However, it's important to consult with your healthcare team before taking any medications.

(2) Monitor your symptoms: Keep track of the frequency and consistency of your bowel movements. If you notice any concerning changes, such as severe or persistent diarrhea, blood in the stool, or fever, inform your healthcare provider immediately. They can evaluate your symptoms and determine if further intervention or adjustments to your treatment plan are necessary.

(3) Seek guidance from your healthcare team: Reach out to your oncologist or oncology nurse if diarrhea becomes a persistent issue during or after chemotherapy. They can provide personalized advice and recommend specific interventions based on your unique situation. They may also evaluate your current medication regimen to identify any potential triggers or interactions that could be contributing to the diarrhea.

(4) Maintain good hygiene practices: Diarrhea can increase the risk of infections, so it's essential to practice good hygiene. Wash your hands thoroughly with soap and water after each bowel movement and before handling food. This helps reduce the likelihood of spreading any potential infections to yourself or others.

**10, How to deal with decreased white blood cells, reduced platelets, or anemia after chemotherapy?**

(1) One approach to dealing with decreased white blood cells, reduced platelets, or anemia after chemotherapy is to monitor your blood counts regularly. Your healthcare team will perform regular blood tests to check your levels of white blood cells, platelets, and hemoglobin. By closely monitoring these counts, your doctors can identify any abnormalities and take appropriate actions to manage them.

(2) Another way to address these issues is through medication. Depending on your specific situation, your healthcare provider may prescribe medications to stimulate the production of white blood cells, platelets, or red blood cells. These medications can help boost your blood counts and improve your overall blood health.

(3) Additionally, it is essential to take precautions to minimize the risk of infections and bleeding. Avoiding crowded places and individuals who are sick can help reduce your exposure to infectious agents. Practicing good hygiene, such as frequent handwashing, can also lower the risk of infection. Taking care to prevent injuries and avoiding activities that may cause bleeding or bruising can help manage the reduced platelet count.

(4) Furthermore, maintaining a healthy lifestyle and following a balanced diet can support your overall blood health. Eating a nutritious diet rich in fruits, vegetables, whole grains, and lean proteins can provide essential vitamins, minerals, and nutrients necessary for blood cell production. It may be helpful to consult a registered dietitian who specializes in oncology to develop a personalized meal plan.

(5) Finally, it's crucial to communicate with your healthcare team about any symptoms or concerns you may have. They can provide guidance, address your specific needs, and make adjustments to your treatment plan if necessary. They may also recommend additional supportive care measures, such as blood transfusions or growth factors, to help manage the blood-related side effects of chemotherapy.

Rep2:

(1) Supportive Care: When dealing with decreased white blood cells, reduced platelets, or anemia after chemotherapy, supportive care measures are essential. This involves taking steps to manage symptoms and promote overall well-being.

One approach is to practice good hygiene and infection control. This includes washing hands regularly, avoiding crowded places, and minimizing exposure to sick individuals. Additionally, maintaining a clean environment and ensuring proper food handling can help reduce the risk of infections.

(2) Medications: Medications can be prescribed to address specific concerns associated with decreased white blood cells, reduced platelets, or anemia. For low white blood cell counts, a doctor may prescribe medications called growth factors to stimulate the production of white blood cells. These medications can help boost the immune system and reduce the risk of infections.

In the case of reduced platelet counts, medications that promote platelet production or decrease platelet destruction may be used. Platelet transfusions may also be necessary in certain situations to increase the platelet levels.

To manage anemia, the doctor might recommend iron supplements or other medications to stimulate red blood cell production. Blood transfusions may be necessary in severe cases to increase the red blood cell count.

(3) Blood Transfusions: Blood transfusions can be a crucial part of managing decreased white blood cells, reduced platelets, or anemia. Transfusions involve receiving blood products from a compatible donor to increase the levels of specific blood components. This can help restore normal blood cell counts and improve overall well-being.

The decision to undergo a blood transfusion is made based on the individual's symptoms, blood test results, and overall health status. Transfusions are usually performed in a hospital setting under the supervision of medical professionals.

(4) Nutrition and Lifestyle: Proper nutrition and a healthy lifestyle can contribute to managing decreased white blood cells, reduced platelets, or anemia. It is important to consume a balanced diet that includes foods rich in iron, vitamin B12, and folate, as these nutrients are essential for red blood cell production. Regular physical activity, as allowed by the individual's health condition, can also promote overall well-being. It is advisable to consult with a healthcare professional or a registered dietitian for personalized dietary recommendations and guidance.

**11, How to prevent and treat numbness in hands and feet after chemotherapy?**

(1) Maintain a healthy lifestyle: Engaging in regular physical activity and maintaining a well-balanced diet can help improve circulation and overall nerve health. This may contribute to reducing numbness in the hands and feet after chemotherapy.

(2) Protect extremities from extreme temperatures: Extreme cold or heat can worsen symptoms of numbness and tingling. It's important to keep your hands and feet warm in cold weather and avoid exposure to excessive heat. Wearing warm gloves and socks can help in cold environments, while avoiding hot surfaces or using protective gloves in hot environments can prevent further damage.

(3) Gentle exercise and physical therapy: Engaging in gentle exercises and physical therapy can help improve blood flow and strengthen the muscles, reducing the severity of numbness. Physical therapists can provide specific exercises and techniques tailored to your needs to improve nerve function and reduce discomfort.

(4) Medications and alternative therapies: There are medications available that can help manage the symptoms of peripheral neuropathy, such as pain relievers, topical creams, or patches. Additionally, some individuals find relief through alternative therapies like acupuncture, massage therapy, or transcutaneous electrical nerve stimulation (TENS). Discuss these options with your healthcare team to determine if they are appropriate for you.

(5) Nerve-stimulating medications: Certain medications like gabapentin and pregabalin can be prescribed to help alleviate neuropathic pain and improve nerve function. These medications work by targeting specific nerve-related symptoms and may be beneficial in managing numbness and tingling.

(6) Regular check-ups with healthcare provider: It is crucial to maintain regular check-ups with your healthcare provider to monitor and manage any potential side effects of chemotherapy, including numbness in the hands and feet. They can provide further guidance, prescribe appropriate medications, or refer you to specialists if needed.

Rep2:

(1) One approach to prevent and treat numbness in hands and feet after chemotherapy is through lifestyle modifications. Patients can try to avoid exposure to extreme temperatures, as extreme cold or heat can worsen the symptoms. Wearing gloves and socks in cold weather and using oven mitts or potholders when handling hot objects can help protect the hands and feet. Regular exercise, particularly low-impact activities like walking or swimming, may improve blood flow and reduce numbness. It is also essential to maintain good overall health by eating a balanced diet, managing stress levels, and getting enough restorative sleep.

(2) Another strategy to manage numbness in hands and feet after chemotherapy is through medication. Certain medications, such as anticonvulsants and antidepressants, may be prescribed by a healthcare professional to help alleviate the symptoms of neuropathy. These medications work by altering the way nerves transmit pain signals. It is important to consult with a healthcare provider to determine the most appropriate medication and dosage for individual circumstances.

(3) Physical therapies can also play a role in preventing and treating numbness in hands and feet. Occupational therapy can help individuals learn techniques to adapt to the challenges posed by neuropathy, such as improving fine motor skills and using assistive devices to maintain independence. Additionally, physical therapy exercises can strengthen muscles, improve balance, and enhance overall coordination, potentially reducing symptoms and improving function. These therapies are typically tailored to an individual's specific needs and abilities.

(4) Alternative approaches such as acupuncture and transcutaneous electrical nerve stimulation (TENS) may also be considered. Acupuncture involves the insertion of thin needles at specific points on the body to stimulate nerve pathways and promote healing. TENS, on the other hand, involves the use of low-voltage electrical currents to provide pain relief. While these methods may not work for everyone, some individuals report a reduction in numbness and associated discomfort after undergoing these treatments.

**12, How to deal with dry, peeling, and painful skin after taking capecitabine?**

(1) One way to deal with dry, peeling, and painful skin after taking capecitabine is to keep your skin well moisturized. Use a gentle, fragrance-free moisturizer multiple times a day to lock in moisture and prevent further drying. Look for products that contain ingredients like ceramides, hyaluronic acid, or glycerin, which help to hydrate and repair the skin barrier.

(2) Another helpful approach is to avoid hot water and harsh soaps when washing your skin. Use lukewarm water instead of hot water, as hot water can strip away the natural oils from your skin and worsen dryness. Opt for mild, non-irritating cleansers or soap substitutes that are specifically formulated for sensitive or dry skin.

(3) It's important to protect your skin from further damage by minimizing sun exposure. When going outside, wear protective clothing like wide-brimmed hats, long-sleeved shirts, and pants. Apply a broad-spectrum sunscreen with a high SPF to exposed areas, even on cloudy days. Reapply sunscreen every two hours, or more frequently if you are sweating or swimming.

(4) Additionally, avoid using harsh exfoliants or scrubs on your skin, as they can further irritate and damage the dry, peeling skin. Instead, opt for gentle exfoliation methods such as a soft washcloth or a mild chemical exfoliant with ingredients like alpha-hydroxy acids (AHAs) or beta-hydroxy acids (BHAs). Be sure to follow the instructions and use them sparingly to avoid excessive exfoliation.

(5) If you are experiencing significant discomfort or pain from the dry and peeling skin, consult with your healthcare provider. They may be able to recommend specific topical treatments or prescribe medicated creams or ointments to alleviate your symptoms. It's essential to keep your healthcare provider informed about any skin-related side effects you are experiencing during your capecitabine treatment.

Rep2:

(1) Keep the skin moisturized: Apply a gentle and fragrance-free moisturizer multiple times a day to keep your skin hydrated. Look for products that are specifically formulated for sensitive or dry skin.

(2) Avoid harsh soaps and hot water: Use mild, non-irritating cleansers and lukewarm water to wash your skin. Harsh soaps and hot water can further dry out and irritate your skin.

(3) Protect your skin from the sun: Use a broad-spectrum sunscreen with at least SPF 30 and apply it generously on exposed areas of your skin before going outdoors. Wear protective clothing, such as wide-brimmed hats and long sleeves, to shield your skin from the sun's harmful rays.

(4) Avoid abrasive or irritating skincare products: Steer clear of products that contain alcohol, fragrances, or other potential irritants. These can exacerbate dryness and discomfort. Opt for gentle skincare products specifically designed for sensitive skin.

(5) Stay hydrated: Drink plenty of water throughout the day to maintain your skin's hydration from within.

(6) Use a humidifier: If the air in your home is dry, consider using a humidifier to add moisture to the air and prevent further drying of your skin.

(7) Protect your hands: Wear gloves when performing household chores or activities that may expose your hands to harsh chemicals or excessive moisture.

(8) Avoid scratching or picking at dry skin: Scratching or picking at dry, peeling skin can lead to further irritation and potential infection. Keep your nails short and consider using moisturizing gloves or dressings on affected areas to prevent scratching.

(9) Consult your healthcare provider: If your skin issues persist or become severe, it is important to seek guidance from your healthcare provider. They may recommend additional treatments or adjustments to your medication regimen to alleviate your symptoms.

**13, Does a greater chemotherapy side effect mean better efficacy?**

(1) A greater chemotherapy side effect does not necessarily mean better efficacy. Chemotherapy works by targeting and killing rapidly dividing cells, including cancer cells. While it can be effective in killing cancer cells, it can also affect healthy cells that divide rapidly, such as those in the bone marrow, digestive tract, and hair follicles. These effects are known as side effects.

(2) The efficacy of chemotherapy is determined by its ability to shrink or eliminate tumors and prevent cancer from spreading or recurring. The primary goal of chemotherapy is to achieve this outcome while minimizing side effects and maintaining the patient's quality of life.

(3) It is important to strike a balance between the desired therapeutic effect and the potential side effects. Oncologists carefully select chemotherapy drugs and treatment regimens based on various factors, including the type and stage of cancer, the patient's overall health, and the expected benefits and risks.

(4) In some cases, more aggressive chemotherapy treatments with higher potential side effects may be warranted if the cancer is aggressive or advanced. However, it is not always the case that higher side effects indicate better efficacy. Each patient's response to chemotherapy can vary, and factors like the tumor's sensitivity to specific drugs and the individual's overall health can influence treatment outcomes. The ultimate goal is to achieve the best possible balance between efficacy and minimizing side effects to optimize the patient's well-being and long-term outcomes.

Rep2:

(1) Generally, a greater chemotherapy side effect does not necessarily mean better efficacy. Chemotherapy drugs work by killing rapidly dividing cells, including cancer cells. However, they can also affect normal cells in the body that naturally divide quickly, such as cells in the hair follicles, bone marrow, and digestive tract. These normal cells are responsible for many of the side effects associated with chemotherapy, such as hair loss, nausea, and low blood cell counts.

(2) While some side effects may be an indication that the chemotherapy is working, it is important to note that the relationship between side effects and efficacy is not straightforward. The effectiveness of chemotherapy is typically assessed based on tumor response, such as tumor shrinkage or stabilization. Side effects, on the other hand, are often a result of the drug's impact on healthy cells. Therefore, a greater side effect does not necessarily correlate with better tumor response or overall efficacy.

(3) In fact, oncologists aim to strike a balance between achieving an effective response to the cancer while minimizing the side effects experienced by the patient. Treatment plans are tailored to each individual, taking into account various factors such as the type and stage of cancer, overall health, and tolerance to treatment. Adjustments may be made to the dosage or schedule of chemotherapy drugs to optimize efficacy and manage side effects.

(4) It is important for patients to communicate any side effects they experience to their healthcare team so that appropriate supportive care measures can be implemented. Managing side effects can improve the patient's quality of life during treatment, but it does not necessarily indicate a better response to the chemotherapy. The ultimate goal is to achieve the best possible outcome in terms of cancer control and patient well-being, while minimizing the impact of side effects.

**14, Does an increase in tumor markers during chemotherapy indicate tumor recurrence?**

(1) An increase in tumor markers during chemotherapy can sometimes indicate tumor recurrence. Tumor markers are substances that are produced by cancer cells or by normal cells in response to cancer. They can be measured in the blood, urine, or other body fluids. Tumor markers are commonly used to monitor the response to cancer treatment, including chemotherapy. During chemotherapy, tumor markers may initially decrease as the treatment kills cancer cells. However, if there is a subsequent increase in tumor markers during or after chemotherapy, it could suggest that the tumor is regrowing or that there are residual cancer cells present. Further evaluation, such as imaging tests or biopsies, would be necessary to confirm tumor recurrence.

(2) It is important to note that not all increases in tumor markers during chemotherapy necessarily indicate tumor recurrence. Tumor markers can also be influenced by other factors, such as inflammation, infection, or other non-cancerous conditions. False-positive results can occur due to these factors, leading to an increase in tumor marker levels without actual tumor growth. Therefore, it is crucial to consider other clinical findings, including imaging tests and the patient's overall condition, to determine the cause of the increase in tumor markers and to differentiate between tumor recurrence and other possibilities.

(3) The interpretation of tumor markers during chemotherapy requires a comprehensive assessment by healthcare professionals. A rising trend in tumor markers over time, along with clinical symptoms and imaging findings consistent with tumor recurrence, may strongly suggest that the cancer has returned. However, the absolute level of tumor markers alone may not be sufficient to establish tumor recurrence definitively. It is crucial to consider the individual's medical history, the specific tumor marker being measured, the baseline levels before treatment, and any changes in treatment response and disease status. Close collaboration between oncologists, pathologists, radiologists, and other specialists is necessary to make an accurate diagnosis and determine the most appropriate management plan.

(4) In summary, an increase in tumor markers during chemotherapy can be concerning for tumor recurrence, but it is not definitive evidence on its own. Clinical evaluation, including imaging tests and consideration of the patient's overall condition, is crucial to confirm the presence of recurrence. False-positive results and other non-cancerous factors can contribute to elevated tumor marker levels. Therefore, it is important to interpret tumor marker results in the context of the individual patient's clinical picture, and further investigations may be necessary to establish an accurate diagnosis.

Rep2:

(1) An increase in tumor markers during chemotherapy can indicate various possibilities and does not necessarily indicate tumor recurrence. Tumor markers are substances produced by cancer cells or other cells in the body in response to cancer. These markers can be detected through blood tests or other diagnostic methods. During chemotherapy, tumor cells may release their contents into the bloodstream as they die, leading to an increase in tumor markers. This phenomenon is known as the "tumor lysis syndrome." Additionally, chemotherapy can cause inflammation and tissue damage, which may also result in higher levels of tumor markers. Therefore, an increase in tumor markers during chemotherapy does not conclusively confirm tumor recurrence.

(2) Tumor markers are not specific to tumor recurrence, and their elevation during chemotherapy can have various causes. Chemotherapy drugs can affect normal cells, leading to inflammation or injury, which can cause an increase in tumor markers. Moreover, the body's response to chemotherapy-induced cell death can temporarily elevate tumor markers. These factors should be considered before jumping to the conclusion of tumor recurrence based solely on elevated tumor markers.

(3) Other factors besides tumor recurrence can contribute to an increase in tumor markers during chemotherapy. For instance, certain non-cancerous conditions, such as benign tumors, infections, or inflammatory disorders, can also cause elevated tumor marker levels. It is crucial to evaluate the patient's overall clinical picture, including imaging studies, physical examination findings, and other relevant diagnostic tests, to determine the exact cause of the increased tumor markers. Further investigation and consultation with a healthcare professional are necessary to interpret these results accurately.

(4) While an increase in tumor markers during chemotherapy can be concerning, it is important to remember that it does not always indicate tumor recurrence. Multiple factors, including tumor lysis syndrome, chemotherapy-induced inflammation, and non-cancerous conditions, can lead to elevated tumor marker levels. To determine the cause, additional clinical evaluation and diagnostic tests are required. Consulting with a healthcare professional who can interpret the results in the context of the patient's individual situation is crucial for accurate assessment and appropriate decision-making.

**15, Do circulating tumor cells need to be routinely tested?**

(1) Circulating tumor cells (CTCs) are cancer cells that have detached from the primary tumor and entered the bloodstream. Testing for CTCs can provide valuable information about the presence and characteristics of cancer, making it a potentially important tool in cancer management. However, routine testing of CTCs is not currently recommended or practiced for all cancer patients.

(2) The decision to test for CTCs depends on various factors, including the type and stage of cancer, treatment plan, and individual patient characteristics. In some cases, CTC testing may be employed as a part of research studies or clinical trials to investigate new therapies or monitor treatment response. However, routine testing is not yet considered a standard of care in most cancer types.

(3) CTC testing techniques are still evolving, and their clinical utility is an area of active research. While the presence of CTCs in the bloodstream indicates the potential for metastasis, it does not guarantee that cancer will spread or that a particular treatment approach is necessary. Additional studies are needed to determine the clinical significance and predictive value of CTC detection and characterization in different cancer types and stages.

(4) In certain situations, CTC testing may be considered for specific cancer types. For example, CTC enumeration using FDA-approved devices is recommended in metastatic breast, prostate, and colorectal cancers to provide prognostic information and guide treatment decisions. However, these recommendations are specific to certain patient populations and may not apply universally across all cancer types.

(5) The field of liquid biopsy, which includes CTC testing, is rapidly advancing. As technologies improve and our understanding of CTC biology expands, routine testing of CTCs may become more widespread. Ongoing research aims to establish standardized protocols, validate the clinical utility of CTC testing, and explore its potential to guide personalized cancer treatment strategies.

Rep2:

(1) Circulating tumor cells (CTCs) refer to cancer cells that have detached from a tumor and entered the bloodstream. Testing for CTCs can provide valuable information about the presence and progression of cancer in a patient. However, the need for routine testing of CTCs depends on several factors such as the type and stage of cancer, treatment plans, and individual patient characteristics.

(2) Routine testing of CTCs can be beneficial in certain cases. It allows for the monitoring of disease progression, assessment of treatment response, and detection of potential metastasis. By analyzing CTCs, healthcare providers can gain insights into the genetic and molecular characteristics of the tumor, aiding in personalized treatment decisions and adjustments.

(3) However, routine testing for CTCs may not be necessary for all cancer patients. Some cancers may not shed a significant number of CTCs into the bloodstream, making their detection challenging. Additionally, the cost and technical complexity of CTC testing can be limiting factors, especially in resource-constrained healthcare settings. In such cases, other diagnostic methods like imaging scans, biopsies, or blood-based biomarker tests may be more practical or informative.

(4) The decision to test for CTCs routinely should be made on an individual basis, considering factors such as cancer type, stage, treatment plan, and patient preferences. In some cases, CTC testing may be recommended as part of a comprehensive cancer management strategy, while in others, it may be reserved for specific clinical scenarios or research purposes. Close collaboration between healthcare providers, oncologists, and patients is crucial to determine the most appropriate testing approach for each individual.

**16, What are the treatment options for advanced colon cancer?**

(1) Surgery: Surgery is often the primary treatment for advanced colon cancer. It involves the removal of the tumor and nearby lymph nodes. In some cases, the surgeon may also need to remove parts of the colon or rectum. Surgery aims to remove as much of the cancer as possible and may be combined with other treatments like chemotherapy or radiation therapy.

(2) Chemotherapy: Chemotherapy is a systemic treatment that uses drugs to kill cancer cells throughout the body. It is commonly used in advanced colon cancer to shrink tumors before surgery, destroy remaining cancer cells after surgery, or help control the disease when surgery is not possible. Chemotherapy can be given orally or intravenously and may involve a single drug or a combination of drugs.

(3) Targeted Therapy: Targeted therapy drugs work by specifically targeting certain molecules or proteins that are involved in the growth and spread of cancer cells. In advanced colon cancer, targeted therapies like cetuximab and panitumumab are used for patients whose tumors have specific genetic mutations, such as KRAS or NRAS mutations. These drugs can be used alone or in combination with chemotherapy.

(4) Immunotherapy: Immunotherapy is a type of treatment that helps to stimulate the body's immune system to recognize and attack cancer cells. In advanced colon cancer, immune checkpoint inhibitors like pembrolizumab and nivolumab may be used for patients with tumors that have high levels of a protein called microsatellite instability-high (MSI-H) or mismatch repair deficiency (dMMR). These drugs can help to slow down the progression of the disease and improve survival in some patients.

(5) Radiation Therapy: Radiation therapy uses high-energy beams to kill cancer cells or shrink tumors. It is sometimes used in advanced colon cancer to relieve symptoms such as pain or to treat tumors that cannot be surgically removed. Radiation therapy is often combined with other treatments like chemotherapy to enhance its effectiveness.

(6) Palliative Care: In advanced colon cancer, palliative care plays a crucial role in managing symptoms, improving quality of life, and providing support to patients and their families. Palliative care focuses on addressing pain, managing side effects of treatments, and providing emotional and psychological support. It can be provided alongside curative treatments or as the primary approach when the cancer is advanced and curative options are limited.

Rep2:

(1) Surgery: Surgery is a common treatment option for advanced colon cancer. It involves removing the tumor and nearby lymph nodes. In some cases, a partial colectomy may be performed to remove a portion of the colon. Surgery aims to remove as much cancer as possible and may be followed by other treatments.

(2) Chemotherapy: Chemotherapy is often used in combination with surgery or as the primary treatment for advanced colon cancer. It uses drugs to kill cancer cells throughout the body. Chemotherapy may be administered before surgery (neoadjuvant chemotherapy) to shrink the tumor or after surgery (adjuvant chemotherapy) to target any remaining cancer cells.

(3) Targeted therapy: Targeted therapy drugs specifically target cancer cells or the processes that promote their growth. In the case of advanced colon cancer, targeted therapies may include drugs that block specific proteins like EGFR (epidermal growth factor receptor) or VEGF (vascular endothelial growth factor) to inhibit tumor growth and the formation of new blood vessels.

(4) Immunotherapy: Immunotherapy is a treatment approach that helps the immune system recognize and attack cancer cells. It can be used in some cases of advanced colon cancer that have specific genetic markers, such as microsatellite instability-high (MSI-H) or mismatch repair deficiency (dMMR). Immunotherapy drugs, such as immune checkpoint inhibitors, can enhance the immune response against cancer cells.

(5) Radiation therapy: Radiation therapy uses high-energy beams to kill cancer cells. It is not typically used as the primary treatment for advanced colon cancer, but it may be recommended in certain situations to relieve symptoms or target specific areas where the cancer has spread, such as the liver or bones.

(6) Palliative care: Palliative care focuses on improving the quality of life for individuals with advanced colon cancer. It aims to manage symptoms, provide pain relief, and address emotional and psychological needs. Palliative care can be provided alongside other treatments and is not limited to end-of-life care.

**17, Can colorectal cancer with lung metastasis be surgically removed?**

**18, What is conversion therapy for liver metastasis of colon cancer?**

(1) Conversion therapy for liver metastasis of colon cancer refers to a treatment approach aimed at converting unresectable liver metastases into resectable lesions. It involves the use of various treatment modalities to downsize or eliminate metastatic tumors in the liver, allowing for potentially curative surgical resection. The goal of conversion therapy is to improve the chances of long-term survival and increase the likelihood of a complete cure for patients with advanced colon cancer that has spread to the liver.

(2) The primary method used in conversion therapy is systemic chemotherapy, which involves administering anticancer drugs to target cancer cells throughout the body. Chemotherapy can help shrink liver metastases, making them potentially operable. The specific chemotherapy drugs used may vary based on factors such as the patient's overall health, the extent of metastasis, and the response to treatment. In some cases, targeted therapies or immunotherapies may be included as part of the treatment regimen.

(3) Alongside chemotherapy, other treatment approaches can be employed in conversion therapy for liver metastasis of colon cancer. These may include local or regional therapies, such as radiofrequency ablation (RFA), microwave ablation (MWA), transarterial chemoembolization (TACE), or selective internal radiation therapy (SIRT). These techniques aim to directly target and destroy liver tumors or restrict their blood supply, thereby reducing their size and enabling subsequent surgical removal.

(4) Additionally, conversion therapy may involve a multidisciplinary approach, with input from medical oncologists, surgical oncologists, radiation oncologists, and other healthcare professionals. They collaborate to develop an individualized treatment plan tailored to the patient's specific condition and overall treatment goals. Close monitoring and regular imaging scans are necessary to assess the response to treatment and determine the optimal timing for surgical resection, if deemed appropriate.

Rep2:

(1) Conversion therapy for liver metastasis of colon cancer is not a recognized or standard treatment approach. The term "conversion therapy" is typically used in the context of attempting to change a person's sexual orientation or gender identity, which is widely discredited and considered unethical. In the case of liver metastasis, various treatment options may be considered, including surgery, chemotherapy, targeted therapies, and radiation therapy, depending on the specific characteristics of the tumor, the extent of metastasis, and the patient's overall health.

(2) Conversion therapy is not an appropriate term to describe the treatment of liver metastasis of colon cancer. Instead, a multidisciplinary approach is generally employed to manage liver metastasis. This may involve a combination of surgical resection to remove the metastatic lesions in the liver, chemotherapy to target cancer cells throughout the body, and other interventions such as radiofrequency ablation or selective internal radiation therapy (SIRT). The goal of treatment is to control the spread of the cancer, alleviate symptoms, and improve overall survival.

(3) Conversion therapy does not have a role in the treatment of liver metastasis of colon cancer. The standard approach for managing liver metastasis from colon cancer involves systemic therapies and local interventions. Systemic treatments, such as chemotherapy or targeted therapies, are administered to target cancer cells throughout the body. Local interventions may include surgical resection of the liver metastases, where feasible, or minimally invasive techniques like radiofrequency ablation or microwave ablation. These interventions aim to remove or destroy the metastatic tumors in the liver, with the goal of prolonging survival and improving quality of life.

(4) Conversion therapy is not a valid treatment modality for liver metastasis of colon cancer. In the context of liver metastasis, treatment strategies typically focus on a combination of systemic therapy and localized interventions. Systemic therapy involves the administration of chemotherapy drugs or targeted therapies that can reach cancer cells throughout the body. Localized interventions may include surgical resection of liver metastases, which involves removing the tumors from the liver, or other techniques like radiofrequency ablation, cryoablation, or transarterial chemoembolization (TACE). The specific treatment approach depends on factors such as the extent of metastasis, the size and number of liver lesions, and the overall health of the patient.

**19,** **What is tumor-free status?**

(1) Tumor-free status refers to the condition of not having any detectable tumors or cancer cells in the body. It indicates that a person has undergone medical examinations, such as imaging tests or biopsies, and no evidence of tumors or cancerous cells has been found. Being tumor-free is a positive outcome, suggesting that the individual is not currently affected by cancer.

(2) Tumor-free status can be achieved through various treatment approaches, such as surgery, radiation therapy, chemotherapy, or targeted therapy. These treatments aim to eliminate or control the growth of tumors in the body. Once the treatment is completed and the medical assessments confirm the absence of any remaining tumors or cancer cells, the individual is considered to be in a tumor-free state.

(3) Regular follow-up visits and medical tests are typically recommended after achieving tumor-free status. These check-ups help monitor the person's health and detect any potential recurrence of tumors or the development of new ones. It's important to note that being in a tumor-free state does not guarantee permanent immunity to cancer, as there can still be a risk of cancer returning or developing elsewhere in the body.

(4) Maintaining a healthy lifestyle and following any recommended preventive measures, such as avoiding tobacco use, maintaining a balanced diet, engaging in regular physical activity, and participating in cancer screening programs, can help reduce the risk of developing new tumors and maintain tumor-free status. It's essential for individuals who have achieved tumor-free status to continue prioritizing their overall well-being and adhering to the guidance of their healthcare providers.

Rep2:

(1) Tumor-free status refers to the absence of any detectable tumors in a person's body. It indicates that there are no visible or measurable signs of abnormal cell growth or tumors during medical examinations or imaging tests. This status is typically determined through various diagnostic methods, such as biopsies, scans, or blood tests, depending on the type of cancer being monitored. Achieving tumor-free status is a positive outcome for individuals who have undergone treatment for cancer, as it suggests that the cancer has been effectively removed or eliminated.

(2) Tumor-free status is an important milestone in cancer treatment and indicates that a person's body is currently free from any cancerous growths. This status is often achieved after successful surgery, radiation therapy, chemotherapy, or a combination of these treatments. The absence of tumors suggests that the cancer cells have been eradicated or reduced to undetectable levels. However, it's important to note that even after achieving tumor-free status, regular follow-up appointments and monitoring are usually recommended to ensure long-term remission and to detect any potential recurrence.

(3) Tumor-free status refers to the absence of tumors in an individual's body, indicating that they are currently free from cancerous growths. It is a positive outcome that indicates successful treatment and management of cancer. Achieving tumor-free status often involves a comprehensive approach to cancer care, which may include surgery to remove tumors, radiation therapy to target cancer cells, or chemotherapy to destroy cancer cells throughout the body. Regular check-ups and follow-up tests are typically advised to monitor the person's condition and ensure that no new tumors develop.

(4) Tumor-free status is the state in which an individual's body shows no evidence of tumors. It signifies that there are no visible or measurable signs of abnormal cell growth or cancerous masses in the body. Achieving tumor-free status is a significant goal in cancer treatment, as it indicates successful eradication or control of the cancer cells. However, it's important to note that tumor-free status does not guarantee a permanent cure, as there may still be microscopic cancer cells that are undetectable by current medical methods. Regular monitoring and ongoing care are necessary to ensure long-term remission and overall health.

**20, What are the circumstances in which colon cancer cannot be surgically treated?**

(1) In cases where the colon cancer has spread extensively to other organs or distant sites in the body, surgical treatment may not be possible. This typically occurs when the cancer has reached an advanced stage and has metastasized beyond the colon. At this point, the cancer cells may have invaded nearby lymph nodes, as well as other organs such as the liver, lungs, or bones. In such cases, the primary goal of treatment shifts towards palliative care to relieve symptoms, improve quality of life, and prolong survival.

(2) Another circumstance where surgical treatment may not be feasible is when a person's overall health is compromised, making them unfit for surgery. Factors that may contribute to this include severe cardiovascular disease, respiratory insufficiency, or other underlying medical conditions that make the risks of surgery too high. In such cases, alternative treatment options, such as radiation therapy, chemotherapy, or targeted therapies, may be considered to manage the cancer.

(3) Sometimes, the location of the tumor within the colon or rectum can make surgical treatment challenging or impossible. For instance, if the tumor is located very close to the anus or involves multiple parts of the colon, it may not be feasible to remove it surgically while preserving normal bowel function. In such cases, a colostomy or ileostomy may be necessary, where a portion of the colon or rectum is bypassed, and the remaining portion is brought out through an opening in the abdominal wall to create a stoma.

(4) If the patient is unable to tolerate general anesthesia due to various reasons such as advanced age, significant comorbidities, or complications from previous surgeries, surgical treatment for colon cancer may not be recommended. In such situations, alternative treatment approaches, including radiation therapy or systemic therapies, may be explored to manage the cancer and its symptoms.

(5) In rare cases, if the cancer has infiltrated major blood vessels or other critical structures in the abdomen, surgical resection may not be possible without excessively compromising the blood supply or causing significant damage. In these situations, the focus of treatment may be directed towards other interventions, such as targeted therapies, radiation therapy, or palliative care, to manage the cancer and alleviate symptoms.

Rep2:

(1) In some cases, colon cancer cannot be surgically treated when it has metastasized, meaning it has spread to other organs or distant sites in the body. When cancer cells have spread beyond the colon and reached distant locations like the liver, lungs, or other vital organs, surgical removal of the primary tumor may not be sufficient to eliminate the cancer completely. In such cases, surgery alone may not be curative, and additional treatment options such as chemotherapy, targeted therapy, or radiation therapy may be recommended to manage the cancer.

(2) Another circumstance where surgical treatment may not be possible is when the tumor is located in a position that makes it technically difficult or risky to perform surgery. For example, if the tumor is located very close to major blood vessels or other important structures, removing it surgically without causing significant harm or complications may be challenging. In such situations, alternative treatment approaches, such as radiation therapy or targeted therapy, may be considered to control the cancer growth and manage the symptoms.

(3) If the patient's overall health is too poor to tolerate surgery, it may be deemed inappropriate or too risky to proceed with surgical treatment. Factors such as advanced age, severe heart or lung disease, or other serious medical conditions can make the surgical procedure too dangerous for the patient. In these cases, the healthcare team may explore alternative treatment options that are less invasive or better suited to the patient's condition, such as chemotherapy, radiation therapy, or palliative care to manage symptoms and improve quality of life.

(4) In certain instances, when the cancer has extensively spread throughout the abdominal cavity and involves multiple organs or structures, it may not be feasible to remove all the cancerous tissue through surgery. This can occur when the cancer has grown extensively, causing extensive adhesions or invasion into adjacent structures, making complete surgical resection impossible without causing severe damage or compromising organ function. In these complex cases, a multidisciplinary approach involving various treatment modalities, including systemic therapies and palliative care, may be recommended to manage the disease and improve the patient's well-being.

**21, What is maintenance therapy for colon cancer?**

(1) Maintenance therapy for colon cancer involves the use of ongoing treatment after the completion of initial therapy, such as surgery and chemotherapy. It is typically recommended for patients who have had their primary tumor removed and are at high risk of cancer recurrence. The goal of maintenance therapy is to prolong the period of remission and prevent or delay the return of cancer cells.

(2) One common approach to maintenance therapy for colon cancer is the use of targeted therapies, such as monoclonal antibodies. These medications specifically target proteins or receptors on cancer cells, inhibiting their growth and spread. For example, the monoclonal antibody cetuximab or panitumumab can be used in patients with metastatic colon cancer that has a specific mutation called KRAS wild-type.

(3) Another type of maintenance therapy for colon cancer involves the use of oral chemotherapy drugs, which can be taken at home. These medications are typically given on a continuous basis to suppress the growth of any remaining cancer cells or micrometastases. For example, capecitabine is an oral chemotherapy drug commonly used in the maintenance setting for colon cancer.

(4) Maintenance therapy may also involve the use of immunotherapy drugs, which harness the body's immune system to target and destroy cancer cells. Immune checkpoint inhibitors, such as pembrolizumab or nivolumab, have shown promising results in certain subsets of patients with advanced or metastatic colon cancer and may be considered for maintenance therapy.

(5) The specific choice of maintenance therapy depends on various factors, including the stage of the cancer, the molecular characteristics of the tumor, the patient's overall health, and the previous treatments received. It is important for patients to discuss with their oncologists to determine the most appropriate maintenance therapy option for their individual situation.

Rep2:

(1) Maintenance therapy for colon cancer refers to the use of ongoing treatment after a patient has completed the initial therapy, such as surgery, chemotherapy, or radiation, for their primary colon cancer. This approach aims to prevent or delay the recurrence of cancer by continuing a less intensive treatment regimen. It is typically recommended for patients who have a higher risk of recurrence or have advanced stages of the disease.

(2) The main goal of maintenance therapy is to eradicate any residual cancer cells that may be present in the body, even after the initial treatment has successfully removed the primary tumor. By providing additional treatment, maintenance therapy helps to minimize the risk of cancer cells regrowing or spreading to other parts of the body. It aims to prolong the time during which the disease remains under control and improve overall survival rates.

(3) Maintenance therapy for colon cancer can involve different approaches, depending on the individual patient's situation and the specific characteristics of their cancer. One common approach is the use of targeted therapies, which are drugs that specifically target certain molecules or pathways involved in cancer growth. These targeted therapies can be given orally, making them convenient for long-term use.

(4) Another form of maintenance therapy is the administration of chemotherapy drugs on a less frequent or lower dose schedule compared to the initial treatment. This approach helps to maintain a certain level of chemotherapy in the body to continue suppressing any remaining cancer cells. In some cases, immunotherapy drugs, which harness the power of the immune system to fight cancer, may also be used as maintenance therapy for colon cancer.

(5) The ~~duration of maintenance therapy varies depending~~ on factors such as the patient's overall health, the stage of the cancer, and the specific treatment plan. It may last for several months to years. Close monitoring and regular follow-up visits with the healthcare team are essential during this phase to evaluate treatment response, manage side effects, and adjust the therapy if necessary.

(6) It's important to note that the decision to pursue maintenance therapy for colon cancer is made on an individual basis, considering various factors such as the patient's overall health, treatment goals, and potential side effect. The healthcare team will carefully assess the risks and benefits and discuss the options with the patient to make an informed decision that best suits their specific situation.

**22,** **Why is genetic testing required before targeted therapy?**

(1) Genetic testing is required before targeted therapy because it helps determine if a patient's tumor has specific genetic alterations that make it susceptible to targeted treatments. Targeted therapy refers to the use of drugs or other interventions that specifically target certain genetic mutations or alterations within cancer cells. By analyzing the genetic makeup of a tumor through genetic testing, doctors can identify specific mutations or alterations that drive the growth of cancer cells. This information is crucial in selecting the most appropriate targeted therapy that can effectively attack and inhibit the cancer cells.

(2) Another reason genetic testing is necessary before targeted therapy is to avoid ineffective or potentially harmful treatments. Different tumors can have unique genetic profiles, and not all tumors will respond to the same targeted therapies. Genetic testing helps identify the specific genetic alterations present in a tumor, enabling oncologists to match the patient with the most appropriate targeted therapy. Without genetic testing, doctors would have to rely on trial-and-error or guesswork when selecting treatments, which could lead to ineffective therapies or unnecessary side effects.

(3) Genetic testing also provides insights into the prognosis and progression of the disease. Certain genetic alterations can indicate a more aggressive form of cancer or a higher likelihood of recurrence. By identifying these genetic markers through testing, doctors can better understand the patient's prognosis and tailor the treatment plan accordingly. This information helps in assessing the potential benefits of targeted therapy and determining the most appropriate timing and duration of treatment.

(4) Furthermore, genetic testing before targeted therapy allows for personalized medicine approaches. Each person's genetic makeup is unique, and the genetic alterations driving their cancer may be different from those in other individuals. Genetic testing helps identify these specific alterations, enabling doctors to develop personalized treatment strategies that address the individual characteristics of the patient's cancer. This personalized approach enhances the chances of a successful response to targeted therapy, potentially leading to improved outcomes for the patient.

(5) In summary, genetic testing before targeted therapy is necessary to identify the specific genetic alterations driving the growth of cancer cells, select the most effective and appropriate targeted therapy, avoid ineffective treatments, assess prognosis and disease progression, and enable personalized medicine approaches. It plays a crucial role in optimizing treatment decisions and improving patient outcomes in the realm of targeted therapy for cancer.

Rep2:

(1) Genetic testing is required before targeted therapy to identify specific genetic alterations or mutations in a patient's DNA. These alterations may be responsible for driving the growth and progression of certain types of cancers or other genetic disorders. By analyzing the patient's genetic makeup, doctors can determine if they are eligible for targeted therapies that are designed to specifically target and inhibit the activity of these altered genes or their proteins.

(2) Targeted therapy is a treatment approach that aims to selectively attack cancer cells or other disease-causing cells while minimizing damage to healthy cells. Since targeted therapies work by interfering with specific molecular targets, it is crucial to know the specific genetic alterations present in a patient's tumor or disease. Genetic testing helps in identifying these alterations, such as mutations in genes like EGFR, HER2, BRAF, or ALK, which are common targets for certain targeted therapies. This information allows doctors to match the patient with the most appropriate targeted therapy, increasing the chances of treatment success.

(3) Genetic testing before targeted therapy also helps in determining the patient's likelihood of responding to a particular treatment. Different genetic alterations can influence how well a patient may respond to a targeted therapy. For example, some mutations may make a patient more or less likely to benefit from a specific targeted therapy. By understanding the genetic profile of the patient, doctors can make informed decisions about the most effective treatment options and avoid therapies that are unlikely to provide significant benefits, potentially saving time and avoiding unnecessary side effects.

(4) Additionally, genetic testing plays a crucial role in monitoring treatment response and detecting the development of resistance. Tumors can evolve over time and develop additional genetic alterations or mutations that make them resistant to targeted therapies. By regularly monitoring the patient's genetic profile through genetic testing during the course of treatment, doctors can detect such changes and modify the treatment approach accordingly. This enables a more personalized and adaptive treatment strategy, ensuring that the patient continues to receive the most effective therapy throughout their treatment journey.

**23,** **What are the methods of genetic testing?**

(1) One method of genetic testing is called polymerase chain reaction (PCR). PCR amplifies specific DNA sequences by making copies of them. This technique allows scientists to obtain enough genetic material for analysis. PCR can be used to identify genetic mutations, such as those associated with inherited disorders or certain types of cancer.

(2) Another method is called fluorescence in situ hybridization (FISH). FISH uses fluorescent probes that bind to specific DNA sequences in cells. By visualizing the fluorescence patterns, researchers can detect chromosomal abnormalities, gene rearrangements, or gene amplifications. FISH is commonly used in prenatal testing and cancer diagnosis.

(3) Next, there is whole-genome sequencing (WGS), which involves sequencing an individual's entire genome. WGS provides a comprehensive analysis of an individual's genetic information. It can identify variations in the DNA sequence, including single nucleotide polymorphisms (SNPs) and larger structural variations. WGS is valuable for diagnosing rare genetic disorders and predicting disease risk.

(4) Array comparative genomic hybridization (aCGH) is a method used to detect copy number variations (CNVs) in the genome. It compares the DNA of a person being tested to a reference DNA sample. By measuring the relative amounts of DNA in different regions, aCGH can identify gains or losses of genetic material. This technique is particularly useful for diagnosing genetic disorders caused by large deletions or duplications.

(5) Next-generation sequencing (NGS) is a high-throughput method that can rapidly sequence millions of DNA fragments simultaneously. NGS techniques include targeted gene panel sequencing, exome sequencing, and whole-genome sequencing. These approaches allow for the identification of specific mutations associated with genetic conditions or the comprehensive analysis of an individual's coding regions.

(6) Lastly, there is karyotyping, a technique used to visualize and analyze an individual's chromosomes. It involves staining the chromosomes to create characteristic banding patterns. Karyotyping can detect chromosomal abnormalities such as aneuploidy (e.g., Down syndrome) or structural rearrangements (e.g., translocations). It is often used in prenatal testing and the diagnosis of certain genetic disorders.

Rep2:

(1) One method of genetic testing is called karyotyping. This involves analyzing the chromosomes in a person's cells to detect any abnormalities or structural changes. A karyotype provides a visual representation of an individual's chromosomes, allowing geneticists to identify genetic disorders caused by an abnormal number or structure of chromosomes.

(2) Another method is called polymerase chain reaction (PCR). PCR is a technique used to amplify a specific region of DNA. It allows scientists to make millions of copies of a specific DNA segment, even if it is present in very low quantities. PCR is commonly used in genetic testing to detect specific genetic mutations or variations associated with certain diseases.

(3) Next, there is fluorescence in situ hybridization (FISH). FISH is a technique that uses fluorescent probes to bind to specific DNA sequences. By analyzing the pattern of fluorescence under a microscope, geneticists can identify the presence or absence of specific genetic abnormalities. FISH is often used to diagnose chromosomal disorders and to detect specific gene rearrangements.

(4) Another method is gene sequencing, which involves determining the precise order of nucleotides in a person's DNA. There are different types of sequencing technologies available, such as Sanger sequencing and next-generation sequencing (NGS). Gene sequencing allows for the identification of specific genetic variations, including single nucleotide polymorphisms (SNPs) or larger structural variants, which can be associated with various genetic conditions.

(5) Additionally, there is array comparative genomic hybridization (aCGH). This method involves comparing the DNA of an individual to a reference DNA using microarrays. It helps detect copy number variations (CNVs), which are changes in the number of copies of a particular DNA segment. aCGH is particularly useful in identifying genetic disorders caused by large deletions or duplications of DNA segments.

(6) Lastly, there is targeted mutation analysis. This method focuses on analyzing specific genes or known mutations associated with a particular disorder. It is often used when a specific genetic condition is suspected, allowing for a more focused analysis of the relevant genes.

**24, What are the main focus points of genetic testing results for colon cancer?**

(1) Genetic testing results for colon cancer focus on identifying mutations or alterations in specific genes that may increase the risk of developing the disease. This involves analyzing a person's DNA to look for changes in genes associated with colon cancer, such as APC, MLH1, MSH2, MSH6, PMS2, and others. These genetic tests aim to provide information about an individual's inherited predisposition to colon cancer and guide medical decisions regarding screening, prevention, and treatment options.

(2) Another important focus of genetic testing results for colon cancer is determining whether a person has a hereditary form of the disease. Certain genetic conditions, such as familial adenomatous polyposis (FAP) and Lynch syndrome (hereditary nonpolyposis colorectal cancer or HNPCC), significantly increase the likelihood of developing colon cancer. Genetic testing can help identify these hereditary forms by detecting specific gene mutations associated with these conditions. This information is valuable for individuals and their healthcare providers to implement appropriate surveillance and preventive measures.

(3) Genetic testing results for colon cancer also provide insights into the potential risk of developing cancer in other organs. Some genetic mutations associated with colon cancer, such as those in the Lynch syndrome genes, are also linked to an increased risk of other types of cancer, including endometrial, ovarian, stomach, small intestine, kidney, and urinary tract cancers. Detecting these mutations through genetic testing enables individuals to take proactive steps for early detection and risk management of multiple cancers.

(4) Additionally, genetic testing results can guide treatment decisions for individuals diagnosed with colon cancer. Certain gene mutations, such as KRAS and BRAF mutations, can affect the response to specific targeted therapies, such as anti-EGFR (epidermal growth factor receptor) treatments. By identifying these mutations, genetic testing helps oncologists personalize treatment plans and select the most effective therapies for each patient, potentially improving treatment outcomes.

(5) Genetic testing results may also have implications for family members of individuals with colon cancer. If a person is found to have a hereditary form of the disease, their close relatives may be at increased risk as well. Genetic testing can identify family members who have inherited the same gene mutation, allowing them to consider preventive measures, such as increased screening or risk-reducing surgeries, to manage their own cancer risk effectively.

(6) Lastly, genetic counseling and support are essential components of genetic testing for colon cancer. Understanding and interpreting genetic test results can be complex and emotionally challenging. Genetic counselors play a crucial role in helping individuals and their families navigate the implications of genetic testing, discussing the results, and providing guidance on screening, prevention, and treatment options. They also address psychological and emotional aspects, helping individuals make informed decisions based on their unique genetic profile.

Rep2:

(1) Genetic testing results for colon cancer typically focus on identifying specific genetic mutations or alterations that may increase an individual's risk of developing the disease. These tests analyze an individual's DNA to look for changes in specific genes associated with colon cancer, such as the APC, MLH1, MSH2, MSH6, PMS2, and EPCAM genes.

(2) One important focus of genetic testing for colon cancer is to detect mutations in genes associated with hereditary forms of the disease, such as Lynch syndrome (hereditary non-polyposis colorectal cancer, or HNPCC) or familial adenomatous polyposis (FAP). Lynch syndrome accounts for about 3-5% of all colon cancers and is caused by mutations in genes involved in DNA mismatch repair. FAP, on the other hand, is characterized by the development of hundreds to thousands of polyps in the colon and is caused by mutations in the APC gene.

(3) Another key aspect of genetic testing for colon cancer is to determine the likelihood of a hereditary cancer syndrome being present in an individual or their family. If a person has a family history of colon cancer or other related cancers, genetic testing can help identify whether the risk is due to an inherited genetic mutation. This information can be crucial for making informed decisions about surveillance, prevention, and treatment options.

(4) Genetic testing results for colon cancer also provide insights into the individual's prognosis and potential response to certain treatments. Some genetic markers, such as the KRAS gene mutation, can influence the effectiveness of certain targeted therapies, such as anti-EGFR drugs. Identifying specific genetic alterations can help guide personalized treatment strategies and improve patient outcomes.

(5) Additionally, genetic testing results can have implications for family members. If a genetic mutation associated with colon cancer is identified in an individual, it may be recommended that their close relatives also undergo genetic testing to assess their own risk. This can aid in early detection and preventive measures for at-risk family members.

(6) Finally, genetic testing results for colon cancer may inform recommendations for regular screening and surveillance. Individuals with certain genetic mutations may require more frequent colonoscopies or additional screening modalities to detect polyps or early-stage cancer at the earliest possible stage.

**25,** **What is immunotherapy for colon cancer?**

(1) Immunotherapy for colon cancer is a type of treatment that harnesses the body's immune system to fight against cancer cells in the colon. It works by stimulating or enhancing the immune response to specifically target and destroy cancer cells. This approach is based on the principle that the immune system has the ability to recognize and eliminate abnormal cells, including cancerous ones. Immunotherapy can be used as a standalone treatment or in combination with other therapies, such as chemotherapy or radiation.

(2) One common form of immunotherapy used for colon cancer is immune checkpoint inhibitors. These drugs target specific molecules on immune cells or cancer cells that regulate immune responses. By blocking these molecules, immune checkpoint inhibitors help remove the brakes on the immune system, allowing it to mount a stronger attack against cancer cells. This can lead to improved tumor control and increased survival rates for patients with advanced colon cancer.

(3) Another type of immunotherapy for colon cancer is adoptive cell transfer therapy. This approach involves extracting immune cells, such as T cells, from a patient's blood or tumor tissue. These cells are then modified and expanded in the laboratory to enhance their ability to recognize and destroy cancer cells. Once the cells are ready, they are infused back into the patient, where they can target and kill cancer cells. Adoptive cell transfer therapy has shown promising results in certain cases of colon cancer, particularly in patients who have not responded well to other treatments.

(4) Vaccines are also being investigated as a potential immunotherapy option for colon cancer. These vaccines aim to stimulate the immune system's response to specific antigens found on colon cancer cells. By exposing the immune system to these antigens, either through injections or oral administration, the vaccines can trigger an immune response that specifically targets and eliminates cancer cells. While vaccine-based immunotherapy for colon cancer is still in the experimental stage, it holds promise as a potential treatment option in the future.

Rep2:

(1) Immunotherapy for colon cancer is a type of cancer treatment that harnesses the power of the immune system to fight cancer cells. It works by stimulating the body's own immune response or by providing immune components such as antibodies to target cancer cells specifically. Immunotherapy has emerged as a promising approach for treating various types of cancer, including colon cancer.

(2) One common form of immunotherapy for colon cancer is immune checkpoint inhibitors. These drugs target certain proteins on immune cells or cancer cells that regulate immune responses. By blocking these proteins, immune checkpoint inhibitors help to unleash the immune system's ability to recognize and attack cancer cells. This can enhance the body's natural defenses and lead to the destruction of colon cancer cells.

(3) Another approach to immunotherapy for colon cancer is adoptive cell transfer. This involves extracting immune cells, such as T cells, from a patient's own body and genetically modifying them to enhance their ability to recognize and kill cancer cells. These modified immune cells are then infused back into the patient, where they can target and destroy colon cancer cells more effectively.

(4) Additionally, cancer vaccines are being developed as a form of immunotherapy for colon cancer. These vaccines aim to stimulate the immune system to recognize specific cancer antigens associated with colon cancer cells. By doing so, they prime the immune system to mount an immune response against the cancer cells, leading to their elimination.

(5) Combination immunotherapy approaches are also being investigated for colon cancer. This involves using multiple immunotherapeutic agents together or in combination with other treatments like chemotherapy or radiation therapy. The goal is to enhance the effectiveness of immunotherapy and improve patient outcomes.

(6) It's important to note that the availability and suitability of immunotherapy for colon cancer may vary depending on factors such as the stage of the cancer, the specific genetic and molecular characteristics of the tumor, and the individual patient's overall health. Therefore, consultation with a healthcare professional is necessary to determine the most appropriate treatment plan for a person with colon cancer.

**26,** **How to evaluate the treatment effect after cancer** **chemotherapy?**

(1) One way to evaluate the treatment effect after colon cancer therapy is by monitoring tumor markers. Tumor markers are substances that can be found in the blood, urine, or tissues, and their levels may change in response to cancer. In the case of colon cancer, the most commonly used tumor marker is called carcinoembryonic antigen (CEA). Regular measurements of CEA levels can help assess the response to treatment. A decrease in CEA levels over time may indicate a positive treatment response, while an increase or persistent elevation may suggest a lack of response or disease progression.

(2) Another method to evaluate the treatment effect after colon cancer therapy is through imaging techniques such as computed tomography (CT) scans or magnetic resonance imaging (MRI). These imaging modalities allow doctors to visualize the colon and surrounding tissues, enabling them to assess the size and extent of tumors. By comparing pre- and post-treatment scans, clinicians can determine if the tumors have shrunk, remained stable, or progressed. Changes in tumor size or appearance on imaging can provide valuable information about treatment effectiveness.

(3) Pathological examination of surgical specimens obtained during colon cancer therapy can also help evaluate treatment effect. After surgery to remove the tumor, the excised tissue is sent to a pathologist who examines it under a microscope. The pathologist assesses various factors, such as the presence of residual cancer cells, the extent of tumor invasion, and the involvement of lymph nodes. This information helps determine if the treatment has successfully eradicated the cancer or if further interventions are necessary. The pathology report provides valuable insights into the response to therapy and guides future treatment decisions.

(4) Evaluation of treatment effect after colon cancer therapy can also include assessing clinical outcomes and patient well-being. This involves monitoring symptoms, overall health, and quality of life. Clinicians may use standardized questionnaires or scales to measure aspects such as pain, fatigue, gastrointestinal function, and emotional well-being. Additionally, the patient's ability to resume daily activities and return to work can be indicators of treatment effectiveness. By considering these clinical factors, healthcare providers can evaluate the holistic impact of therapy on the patient's life beyond the direct tumor response.

Rep2:

(1) One common approach to evaluate the treatment effect after colon cancer therapy is to monitor the patient's overall survival. This involves tracking the length of time from the initiation of treatment to the patient's death or the end of the study period. Overall survival provides a comprehensive measure of treatment efficacy, as it takes into account not only the response to treatment but also the impact on the patient's long-term prognosis.

(2) Another method to assess treatment effect is by measuring disease-free survival. Disease-free survival evaluates the length of time after treatment during which the patient remains free from any signs or symptoms of colon cancer recurrence or progression. It provides valuable information on the effectiveness of the therapy in preventing the return or spread of the disease.

(3) Assessing tumor response is an important aspect of evaluating treatment effect. This is often done using imaging techniques, such as computed tomography (CT) scans or magnetic resonance imaging (MRI), which allow for the visualization of the tumor. Response Evaluation Criteria in Solid Tumors (RECIST) criteria are commonly used to classify tumor response as complete response, partial response, stable disease, or progressive disease. By comparing pre- and post-treatment images, clinicians can determine the extent of tumor shrinkage or stabilization, providing insights into the efficacy of the therapy.

(4) Biomarker analysis can also be employed to evaluate treatment effect. Biomarkers are measurable substances or indicators present in the body that can provide information about the patient's response to therapy. In the case of colon cancer, biomarkers such as carcinoembryonic antigen (CEA) and circulating tumor DNA (ctDNA) can be measured before and after treatment to assess treatment response. A reduction in biomarker levels following therapy may indicate a positive treatment effect.

(5) Patient-reported outcomes (PROs) play a crucial role in evaluating the treatment effect from the patient's perspective. PROs capture the patient's self-reported symptoms, quality of life, and functional status before and after treatment. Assessing PROs allows clinicians to understand the impact of therapy on the patient's well-being, physical functioning, and emotional state, providing a comprehensive evaluation of treatment effectiveness beyond clinical and radiological measures.

(6) Additionally, evaluating treatment effect through histopathological examination of surgical specimens can provide valuable insights. Pathologists analyze the resected tumor tissue to assess the extent of tumor regression, presence of residual cancer cells, and other relevant histological features. This information helps in determining the response to therapy and can guide further treatment decisions.

**27, What are the side effects of cetuximab? How to deal with them?**

(1) One of the common side effects of cetuximab is skin reactions. These can include acne-like rash, dry skin, itching, and nail changes. It is important to keep the skin clean and moisturized. Avoiding excessive sun exposure and using sunscreen with a high SPF can help protect the skin. If the rash becomes severe or causes discomfort, it is advisable to consult a healthcare professional for appropriate management.

(2) Gastrointestinal side effects may occur with cetuximab treatment. These can include diarrhea, nausea, vomiting, and abdominal pain. Maintaining good hydration by drinking plenty of fluids and eating small, frequent meals may help alleviate these symptoms. In some cases, medication such as anti-diarrheals or anti-nausea drugs may be prescribed by a doctor to manage these side effects.

(3) Infusion reactions can sometimes occur during or shortly after receiving cetuximab. These reactions may include fever, chills, shortness of breath, and changes in blood pressure. Prior to each infusion, pre-medications such as antihistamines and corticosteroids may be given to reduce the likelihood of infusion reactions. Close monitoring during and after the infusion is important to promptly address any potential reactions.

(4) Fatigue is another potential side effect of cetuximab. It is important to listen to your body and rest when needed. Balancing physical activity with sufficient rest periods can help manage fatigue. Maintaining a healthy diet, staying hydrated, and engaging in light exercise, if approved by a healthcare professional, may also help combat fatigue.

(5) In some cases, cetuximab can lead to changes in electrolyte levels, such as low magnesium and calcium levels. Regular monitoring of electrolyte levels through blood tests is important during treatment. If electrolyte imbalances occur, supplements or medications may be prescribed to restore the balance.

(6) Cetuximab may also affect the liver, leading to elevated liver enzyme levels. Regular liver function tests are usually performed to monitor any changes. If liver enzyme levels become significantly elevated, dosage adjustments or temporary discontinuation of cetuximab may be necessary.

(7) Other less common side effects of cetuximab can include infection, low blood cell counts, and allergic reactions. It is important to promptly report any signs of infection, such as fever or persistent sore throat, to a healthcare professional. Regular blood tests are usually conducted to monitor blood cell counts. If severe allergic reactions occur, immediate medical attention should be sought.

Rep2:

1. One common side effect of cetuximab is skin rash or acne-like rash. This side effect can occur in approximately 80% of patients receiving cetuximab. The rash is usually mild to moderate and commonly affects the face, chest, and back. It may cause itching, redness, dryness, and sometimes blistering. To manage this side effect, it is recommended to keep the skin clean and moisturized. Avoid using harsh soaps or abrasive skin products and opt for gentle cleansers and fragrance-free moisturizers. Applying corticosteroid creams or ointments prescribed by a healthcare professional may also help alleviate the rash.

2. Another potential side effect of cetuximab is gastrointestinal symptoms such as diarrhea, nausea, and vomiting. These symptoms can be managed by following dietary recommendations provided by the healthcare team, such as consuming small, frequent meals and avoiding spicy or greasy foods. Staying hydrated and drinking plenty of fluids is important to prevent dehydration. In some cases, medications to control diarrhea or anti-nausea drugs may be prescribed by the healthcare provider.

3. Cetuximab can also lead to an infusion reaction, which typically occurs during or shortly after the infusion of the medication. Symptoms may include fever, chills, difficulty breathing, chest tightness, dizziness, or skin rash. To manage infusion reactions, the healthcare team closely monitors patients during the infusion and may administer medications such as antihistamines or corticosteroids to alleviate the symptoms. In severe cases, the infusion may need to be temporarily stopped or permanently discontinued.

4. Fatigue is another possible side effect of cetuximab. It can range from mild tiredness to extreme exhaustion, affecting daily activities and quality of life. Managing fatigue involves conserving energy and balancing activities with rest periods. Prioritizing tasks, delegating responsibilities when possible, and seeking support from family and friends can also help cope with fatigue. It is important to communicate with the healthcare team about any persistent or debilitating fatigue experienced during cetuximab treatment.

**28, Are the side effects of immunotherapy significant? How to deal with them?**

(1) Side effects of immunotherapy can vary depending on the specific type of immunotherapy and individual patient factors. Generally, immunotherapy works by stimulating the body's immune system to fight against cancer or other diseases. While immunotherapy has been a significant advancement in cancer treatment, it can also cause side effects, although they may not affect everyone in the same way.

(2) The side effects of immunotherapy can range from mild to severe. Common side effects include fatigue, fever, chills, nausea, loss of appetite, skin rash, and flu-like symptoms. These side effects typically occur because immunotherapy activates the immune system, which can result in an overactive response leading to inflammation throughout the body. It's important to note that the severity and duration of side effects can vary between individuals.

(3) Dealing with the side effects of immunotherapy requires close communication and coordination with the healthcare team. If you experience side effects, it's crucial to report them to your healthcare provider promptly. They can provide guidance on managing the specific side effects you are experiencing. In some cases, medications such as corticosteroids or antihistamines may be prescribed to alleviate symptoms. It's important to follow your healthcare provider's instructions and not to take any additional medications without consulting them.

(4) Additionally, taking good care of your overall health can help manage side effects. Getting enough rest, eating a balanced diet, staying hydrated, and engaging in light exercise as recommended by your healthcare team can contribute to your well-being during immunotherapy. It's also essential to communicate openly with your healthcare provider about any concerns or questions you may have regarding the side effects or the treatment itself.

(5) Remember that the potential benefits of immunotherapy in treating cancer or other diseases can outweigh the risks associated with side effects. Each individual's response to immunotherapy can vary, and while some may experience significant side effects, others may have minimal or no side effects at all. Working closely with your healthcare team and following their guidance can help manage any side effects that arise and optimize your treatment outcomes.

Rep2:

(1) Immunotherapy can have significant side effects, although they vary depending on the specific type of immunotherapy and the individual's response to treatment. Some common side effects include fatigue, fever, chills, nausea, loss of appetite, skin reactions (rash, itching), and flu-like symptoms. These side effects typically occur because immunotherapy drugs stimulate the immune system to attack cancer cells, which can also affect healthy cells in the body.

(2) Managing side effects of immunotherapy involves close communication and collaboration with healthcare professionals. It is crucial to report any side effects experienced during treatment promptly. In some cases, medications such as antihistamines, corticosteroids, or anti-nausea drugs may be prescribed to alleviate specific symptoms. Maintaining good communication with the healthcare team allows for appropriate adjustments to the treatment plan or supportive care measures to be implemented.

(3) In addition to pharmacological interventions, there are several self-care strategies that can help manage side effects of immunotherapy. These include getting enough rest and sleep, maintaining proper hydration, eating a balanced diet, and engaging in light exercise if recommended by the healthcare provider. It is also essential to avoid exposure to potential infections and follow proper hygiene practices to minimize the risk of complications.

(4) Another crucial aspect of dealing with side effects is addressing the emotional and psychological impact of immunotherapy. Going through cancer treatment and experiencing side effects can be challenging, and it is essential to seek emotional support from loved ones, support groups, or professional counseling services. Mental health plays a significant role in overall well-being, and having a strong support system can help navigate the ups and downs of treatment.

(5) It is important to note that the severity and duration of side effects can vary from person to person. Some individuals may experience only mild side effects, while others may have more pronounced or long-lasting symptoms. Regular communication with the healthcare team is vital to ensure that any concerns or changes in side effects are addressed promptly and appropriately. They can provide personalized advice and support based on the individual's specific situation.

**29, What is a clinical trial for drugs?**

(1) A clinical trial for drugs is a carefully designed research study conducted to evaluate the safety and effectiveness of new medications or treatment approaches. It involves testing the drug on human volunteers or patients under controlled conditions to gather data about its effects, potential side effects, and optimal dosage. Clinical trials follow specific protocols or plans to ensure scientific validity and ethical considerations. They are a crucial step in the development and regulatory approval process of new drugs before they can be made available to the general public.

(2) In a clinical trial, researchers recruit participants who meet specific criteria and divide them into different groups. One group receives the investigational drug being tested (experimental group), while another group receives either a placebo or the current standard treatment (control group). The trial aims to compare the outcomes between these groups, helping to determine the drug's efficacy and safety profile. To minimize bias, often neither the participants nor the researchers know which group is receiving the investigational drug until the trial is completed (double-blind study).

(3) Clinical trials follow a series of phases. Phase 1 trials are typically the first step and involve a small number of healthy volunteers. They focus on assessing the drug's safety, dosage range, and potential side effects. Phase 2 trials involve a larger group of participants, often patients with the targeted condition, and aim to evaluate the drug's effectiveness and further assess its safety. Phase 3 trials include a larger number of patients and compare the drug with the current standard treatment to establish its benefits, risks, and optimal usage. Phase 4 trials occur after the drug has been approved and monitor its long-term safety and effectiveness in a larger population.

(4) Throughout the clinical trial process, researchers collect and analyze data on the drug's efficacy, safety, and potential adverse effects. They closely monitor participants, often conducting regular check-ups and tests to assess any changes in their condition. The trial results are carefully evaluated to determine if the drug meets the desired objectives and if it can be approved for wider use. The findings are shared with regulatory authorities, such as the Food and Drug Administration (FDA), who review the data to make informed decisions about the drug's approval and labeling. Clinical trials play a vital role in advancing medical knowledge, improving patient care, and bringing new treatments to those in need.

Rep2:

(1) A clinical trial for drugs is a carefully designed and controlled study that evaluates the safety and effectiveness of a new medication or treatment. It involves testing the drug on human participants to gather data and determine its potential benefits and potential side effects.

(2) Clinical trials are conducted in several phases. In Phase 1, a small group of healthy volunteers or patients receive the drug to assess its safety, dosage, and potential side effects. Phase 2 involves a larger group of participants to further evaluate the drug's effectiveness and side effects. Phase 3 trials include an even larger group of participants and compare the new drug with existing treatments to gather more data on its benefits, risks, and optimal usage. Finally, in Phase 4, after the drug is approved, post-marketing studies are conducted to monitor its long-term effects and ensure ongoing safety.

(3) Clinical trials follow strict protocols that outline the study objectives, participant eligibility criteria, treatment procedures, and data collection methods. They are typically conducted by pharmaceutical companies, academic institutions, or government agencies with oversight from regulatory authorities. Participants in clinical trials may be assigned to different treatment groups, such as receiving the new drug, a placebo, or an existing standard of care, to compare the outcomes and determine the drug's efficacy.

(4) Clinical trials play a vital role in advancing medical knowledge and improving patient care. They provide crucial evidence for regulatory agencies to evaluate the drug's safety and efficacy, leading to potential approval and widespread use. Additionally, participating in clinical trials gives patients access to cutting-edge treatments that may not otherwise be available, while also contributing to the scientific community's understanding of diseases and their treatment options.

**30, Under what circumstances can one participate in a clinical trial?**

(1) Individuals can participate in a clinical trial under the following circumstances:

- Medical condition: Clinical trials typically focus on specific medical conditions or diseases. If a person has the condition being studied, they may be eligible to participate in the trial.

- Inclusion criteria: Each clinical trial has specific eligibility criteria outlined in its protocol. These criteria may include factors such as age, gender, medical history, stage of the disease, and previous treatments. If a person meets the inclusion criteria, they may be able to participate in the trial.

- Informed consent: Participation in a clinical trial requires providing informed consent. This means individuals must be fully informed about the trial, its potential risks and benefits, and their rights as participants. They have the option to agree or decline participation after understanding the details.

(2) Another circumstance under which someone can participate in a clinical trial is if they have exhausted standard treatment options. Clinical trials often explore new interventions or therapies that are not yet approved or widely available. If a person has tried and failed standard treatments for their condition, they may consider participating in a clinical trial as an alternative option.

(3) In some cases, healthy individuals may also be eligible to participate in clinical trials. These trials typically focus on preventive measures, such as vaccines or medications to reduce the risk of developing a specific condition. Healthy volunteers play a crucial role in evaluating the safety and effectiveness of these interventions, and their participation can contribute to scientific advancements and medical knowledge.

(4) Certain clinical trials may specifically target individuals from particular demographics or population groups. This could be based on factors such as ethnicity, genetic predispositions, or unique health conditions prevalent in certain populations. Inclusion of diverse participants in clinical trials helps ensure that the results are applicable to a broader range of individuals and can lead to more equitable healthcare outcomes.

(5) Lastly, individuals who are interested in contributing to medical research and advancing scientific knowledge may choose to participate in clinical trials voluntarily. Their motivation may stem from a desire to help others with similar conditions, access potential new treatments, or actively contribute to medical progress by providing valuable data for researchers and clinicians to analyze.

Rep2:

(1) Individuals can participate in a clinical trial if they meet the eligibility criteria set by the researchers conducting the study. These criteria vary depending on the specific trial and may include factors such as age, gender, medical condition, previous treatments received, and overall health. Eligibility criteria help ensure that the study results are meaningful and applicable to the target population.

(2) Informed consent is another crucial requirement for participation in a clinical trial. Before joining a study, participants must be provided with detailed information about the trial, including its purpose, potential risks and benefits, procedures involved, and any alternative treatment options. Participants have the right to ask questions and fully understand the implications of their involvement. Giving informed consent is a voluntary decision, and individuals can choose to withdraw from the trial at any time.

(3) Participation in a clinical trial often involves a commitment to follow the study protocol. This includes adhering to specific interventions or treatments, attending scheduled visits and assessments, and providing necessary information to the research team. The study protocol helps ensure consistency and reliability of data collection across all participants. It is important for individuals considering participation to understand and be willing to comply with the requirements of the trial.

(4) Clinical trials may have additional criteria specific to the research objectives and the investigational treatment being studied. For example, a trial testing a new medication might exclude individuals with certain pre-existing medical conditions or those taking certain medications that could interact with the investigational drug. These additional criteria aim to create a controlled environment that maximizes the safety and effectiveness evaluation of the intervention under investigation.

(5) It's worth noting that clinical trials can also have specific exclusion criteria, which are factors that disqualify individuals from participating. These criteria are put in place to protect the safety of participants or to maintain the integrity of the study results. Exclusion criteria may include factors such as severe medical conditions, pregnancy, specific allergies, or previous adverse reactions to similar treatments. The purpose is to minimize potential risks and ensure the trial focuses on the intended target population.

(6) Lastly, individuals interested in participating in a clinical trial can seek guidance from their healthcare providers or directly contact the researchers conducting the study. The research team can provide detailed information about the trial, assess eligibility, and address any concerns or questions potential participants may have. Healthcare professionals play an essential role in guiding patients through the decision-making process and providing support throughout their participation in the trial.

**31, How to obtain information about drug clinical trials?**

(1) One way to obtain information about drug clinical trials is through online databases and registries. There are several reputable platforms that compile information about ongoing and completed clinical trials, such as ClinicalTrials.gov, which is maintained by the U.S. National Library of Medicine. These databases provide comprehensive information about the purpose, design, eligibility criteria, and locations of clinical trials. Users can search for trials by specific drugs, medical conditions, or locations to find relevant information.

(2) Another source of information about drug clinical trials is pharmaceutical companies and research organizations. Many companies conduct their own clinical trials to test the safety and efficacy of their drugs. They often provide information about ongoing trials on their websites or through other communication channels. Pharmaceutical companies may also collaborate with healthcare professionals or patient advocacy groups to disseminate information about clinical trials to potential participants.

(3) Medical institutions and research centers are also valuable sources of information about drug clinical trials. Universities, hospitals, and other healthcare facilities often conduct clinical trials to advance medical knowledge and improve patient care. These institutions typically have dedicated research departments or centers that coordinate and oversee clinical trials. Interested individuals can reach out to these institutions directly to inquire about ongoing trials and eligibility criteria.

(4) Additionally, healthcare professionals can be a valuable resource for obtaining information about drug clinical trials. Physicians, nurses, and other healthcare providers often have access to information about ongoing trials within their respective fields. They can inform patients about relevant clinical trials and provide guidance on how to participate. Healthcare professionals may also collaborate with research organizations or have affiliations with specific clinical trial sites, enabling them to provide up-to-date information about available trials.

Rep2:

1) One way to obtain information about drug clinical trials is to visit reputable websites that provide comprehensive databases of clinical trials. These websites, such as ClinicalTrials.gov (maintained by the U.S. National Library of Medicine) or the European Union Clinical Trials Register, serve as centralized repositories for clinical trial information. Users can search for specific drugs or medical conditions to find ongoing or completed trials, along with details such as trial phase, study objectives, eligibility criteria, and contact information for trial investigators.

2) Another method to access information about drug clinical trials is to consult healthcare professionals or researchers specialized in the particular area of interest. Physicians, pharmacists, or academic researchers often stay updated with the latest developments in clinical research and may have access to unpublished or upcoming trials. They can provide insights and guidance based on their expertise and help individuals find relevant trials or connect them with appropriate resources.

3) Many pharmaceutical companies and research institutions maintain their own websites or online portals where they publish information about clinical trials for the drugs they are developing. These websites may include details about ongoing or planned trials, including study protocols, recruitment status, and trial locations. Checking the websites of relevant pharmaceutical companies or research institutions can be a valuable source of information on drug clinical trials.

4) Additionally, attending medical conferences, seminars, or symposiums focused on the specific area of interest can provide opportunities to learn about ongoing clinical trials. These events often feature presentations by researchers and experts who share their findings and provide updates on clinical trials in their respective fields. Networking with professionals at such events can help gather information and establish connections with individuals involved in clinical research.

**32, Does advanced-stage cancer always result in cachexia?**

(1) No, advanced-stage cancer does not always result in cachexia. Cachexia is a complex metabolic syndrome characterized by severe weight loss, muscle wasting, and systemic inflammation. It commonly occurs in advanced stages of various chronic illnesses, including cancer. However, not all cancer patients experience cachexia, and its presence depends on multiple factors such as the type of cancer, tumor location, individual response to treatment, and overall health status. Some cancer patients may maintain their weight and muscle mass relatively well, especially if they receive appropriate nutritional support and have effective disease management strategies.

(2) While cachexia is a common occurrence in advanced-stage cancer, it is not an inevitable consequence. Advanced cancer can manifest in different ways, and cachexia represents one possible outcome. The development of cachexia depends on several factors, including the tumor's metabolic demands, the body's response to the tumor, and the effectiveness of treatment interventions. In some cases, cancer patients may experience weight loss and muscle wasting due to factors other than cachexia, such as treatment side effects or complications. Therefore, it is important to recognize that cachexia is not universally present in all advanced cancer cases.

(3) Cachexia is a prevalent condition observed in many advanced-stage cancer patients, but it does not affect everyone. The mechanisms leading to cachexia are not fully understood, but they are believed to involve a complex interplay between the tumor and the body's response. Factors such as inflammation, altered metabolism, and hormonal imbalances contribute to the development of cachexia. However, individual variations in tumor biology, host factors, and treatment strategies can influence whether a cancer patient experiences cachexia or not. It is crucial to implement supportive care measures, including nutritional support and symptom management, to address cachexia when it occurs.

(4) Advanced-stage cancer can lead to cachexia in some cases, but it is not a universal outcome. Cachexia is a multifactorial syndrome associated with a combination of metabolic changes, systemic inflammation, and anorexia. These factors can result in weight loss, muscle wasting, and reduced quality of life. However, the occurrence of cachexia varies among cancer patients, and some individuals may maintain their weight and muscle mass despite being in an advanced stage of the disease. Proper management and supportive care, including nutritional interventions and addressing the underlying causes of cachexia, can help mitigate its impact on patients' well-being.

Rep2:

(1) No, advanced-stage cancer does not always result in cachexia. Cachexia is a complex syndrome characterized by progressive weight loss, muscle wasting, and loss of appetite that can occur in some cancer patients. It is estimated to affect approximately half of all cancer patients, but the occurrence and severity of cachexia can vary widely depending on various factors such as the type of cancer, individual patient characteristics, and the effectiveness of cancer treatment.

(2) While cachexia is a common complication in advanced-stage cancer, it is not an inevitable consequence for all patients. Some cancer patients may maintain their weight and muscle mass despite being in advanced stages of the disease. This could be due to several factors, including the specific type of cancer they have, the effectiveness of their treatment, their nutritional status, and their overall health.

(3) Although cachexia is often associated with advanced-stage cancer, it is important to note that not all cancer patients will experience cachexia. Some individuals may undergo cancer treatment without significant weight loss or muscle wasting. The development of cachexia is influenced by various factors, such as the tumor type and location, the presence of other health conditions, the immune response to the cancer, and the overall nutritional and metabolic status of the patient.

(4) The occurrence of cachexia in advanced-stage cancer patients is multifactorial. While it is a common phenomenon, it is not an inherent feature of all cases. The interplay of tumor-related factors, systemic inflammation, metabolic changes, and the host response contribute to the development of cachexia. However, individual variations in these factors and the effectiveness of treatment can lead to differing outcomes, with some patients experiencing cachexia while others do not.

**33, How can cachexia be detected? How to determine its severity?**

(1) Cachexia can be detected through various methods, including medical assessments and physical examinations. One of the primary indicators is unintentional weight loss, typically greater than 5% of the individual's total body weight within a span of 6 months. Doctors may evaluate changes in body mass index (BMI) and assess the distribution of muscle and fat to determine the presence of cachexia. Additionally, symptoms such as loss of appetite, muscle wasting, and fatigue can also be indicative of cachexia.

(2) To determine the severity of cachexia, healthcare professionals often use a combination of subjective and objective measures. Subjective assessments involve evaluating the patient's self-reported symptoms, including fatigue, appetite, and quality of life. Objective assessments include measuring body weight, body composition (e.g., muscle mass and fat mass), and functional capacity (e.g., grip strength and walking ability). Laboratory tests may also be conducted to assess levels of inflammation markers and nutritional status. Combining these evaluations provides a comprehensive understanding of the severity of cachexia.

(3) Another approach to determining the severity of cachexia is through the use of scoring systems or criteria. For example, the "Weight Loss Grading System" categorizes cachexia into different stages based on the percentage of weight loss. This system helps classify the severity as mild, moderate, or severe. Similarly, the "Functional Assessment of Anorexia/Cachexia Therapy (FAACT)" questionnaire assesses multiple aspects of cachexia, including weight loss, appetite, and quality of life, to provide a quantitative measure of its severity. These scoring systems can assist in standardizing the evaluation and monitoring of cachexia.

(4) In addition to the methods mentioned above, imaging techniques such as dual-energy X-ray absorptiometry (DEXA) and computed tomography (CT) scans can also be used to assess body composition and identify muscle wasting. These imaging modalities provide detailed information about muscle and fat distribution, helping to determine the severity of cachexia. Furthermore, functional tests like cardiopulmonary exercise testing (CPET) can evaluate exercise capacity and metabolic abnormalities associated with cachexia, aiding in the assessment of its severity and impact on physical function.

Rep2:

(1) Cachexia can be detected through various methods, including clinical assessment, body composition analysis, and laboratory tests. Clinical assessment involves evaluating the patient's overall appearance, including weight loss, muscle wasting, and changes in body composition. Body composition analysis, such as dual-energy X-ray absorptiometry (DEXA) or bioelectrical impedance analysis (BIA) can provide quantitative measurements of muscle mass, fat mass, and bone density. Laboratory tests may include assessing inflammatory markers, such as C-reactive protein (CRP) and interleukin-6 (IL-6), as well as other markers of nutritional status and metabolic function.

(2) The severity of cachexia can be determined based on several factors. One commonly used method is the calculation of body mass index (BMI), which compares an individual's weight to their height. However, BMI alone may not accurately reflect the severity of cachexia as it does not distinguish between fat loss and muscle wasting. Other parameters such as weight loss percentage, changes in muscle mass or strength, and functional impairment can also be evaluated to assess cachexia severity. Additionally, the patient's underlying medical condition and the impact of cachexia on their overall well-being and quality of life are taken into consideration.

(3) In addition to the methods mentioned above, the severity of cachexia can be assessed using validated scoring systems. One such scoring system is the "Cachexia Staging System" developed by Fearon et al. This system categorizes cachexia into four stages based on weight loss, anorexia, muscle wasting, and functional impairment. Another commonly used tool is the "Patient-Generated Subjective Global Assessment" (PG-SGA), which combines patient-reported symptoms, dietary intake, weight change, and physical examination to assess the severity of cachexia and guide nutritional interventions.

(4) It's important to note that cachexia is a complex condition, and its detection and severity assessment may vary depending on the underlying disease or condition causing it. Healthcare professionals, such as physicians, dietitians, and oncologists, play a crucial role in evaluating and monitoring cachexia in patients. They consider a combination of clinical assessment, body composition analysis, laboratory tests, and scoring systems to determine the presence and severity of cachexia, helping guide appropriate interventions and management strategies.

**34, How is cachexia treated?**

(1) One approach to treating cachexia involves addressing the underlying causes contributing to the condition. Since cachexia is often associated with chronic illnesses such as cancer, chronic kidney disease, or heart failure, managing these conditions is crucial. This may involve various treatments such as chemotherapy, radiation therapy, or surgery for cancer; medications to manage symptoms and improve organ function in kidney disease or heart failure; or addressing any infections or other complications that may be present.

(2) Another aspect of cachexia treatment focuses on nutritional support. This typically involves optimizing the patient's nutrient intake to combat weight loss and muscle wasting. Strategies may include dietary modifications, such as increasing protein and calorie intake, as well as the use of oral nutritional supplements or enteral feeding (tube feeding) when oral intake is insufficient. In some cases, parenteral nutrition (intravenous feeding) may be necessary.

(3) Physical activity and exercise can play a beneficial role in managing cachexia. Engaging in regular physical activity, under the guidance of healthcare professionals, can help preserve muscle mass, improve strength, and enhance overall functional capacity. Exercise programs may include resistance training, aerobic exercises, and other tailored activities based on the individual's capabilities and medical condition.

(4) Pharmacological interventions may also be employed in cachexia treatment. Several medications have been studied for their potential to counteract muscle wasting and promote weight gain. These may include appetite stimulants like megestrol acetate, anabolic agents such as selective androgen receptor modulators (SARMs) or testosterone, and anti-inflammatory drugs. However, it is important to note that the effectiveness of these medications in cachexia management is still being explored, and individual responses may vary.

Rep2:

(1) One approach to treating cachexia involves addressing the underlying cause of the condition. This may involve treating the underlying disease or condition responsible for cachexia, such as cancer, chronic obstructive pulmonary disease (COPD), heart failure, or HIV/AIDS. By managing the primary disease, it is possible to improve the symptoms of cachexia.

(2) Nutritional support is another crucial aspect of cachexia treatment. This involves providing adequate nutrition to help combat weight loss and muscle wasting. Nutritional interventions may include increasing calorie intake, ensuring sufficient protein intake, and using oral nutritional supplements or tube feeding if necessary. The goal is to provide the body with the necessary nutrients to prevent further weight loss and promote muscle preservation.

(3) Physical exercise and rehabilitation can play a significant role in managing cachexia. Exercise programs tailored to the individual's capabilities can help improve muscle strength, endurance, and overall physical function. Physical therapy and occupational therapy may also be beneficial in maintaining or improving functional abilities.

(4) Pharmacological interventions may be considered in cachexia treatment. Several medications have been studied in the context of cachexia, including appetite stimulants, anabolic agents, anti-inflammatory drugs, and other supportive medications. However, the effectiveness of these pharmacological interventions in cachexia management varies, and their use should be determined on a case-by-case basis.

**35, How should follow-up and reexamination be conducted after colon cancer surgery?**

(1) Regular follow-up and reexamination after colon cancer surgery are crucial for monitoring the patient's recovery, detecting any potential recurrence or complications, and providing appropriate interventions. The specific follow-up schedule may vary based on individual factors such as the stage of the cancer, the extent of surgery, and the patient's overall health. Generally, a comprehensive approach to follow-up and reexamination involves a combination of medical history assessment, physical examination, imaging tests, and laboratory investigations.

(2) One important aspect of post-operative follow-up is monitoring the patient's medical history and conducting a thorough physical examination. The medical history assessment involves evaluating any new symptoms or concerns that the patient may have, as well as reviewing their overall well-being and quality of life. The physical examination aims to identify any physical abnormalities or signs of potential complications, such as incisional hernias or surgical site infections.

(3) Imaging tests play a vital role in post-operative follow-up for colon cancer patients. These tests may include periodic CT scans or MRI scans to evaluate the surgical site, nearby lymph nodes, and other relevant organs. These imaging modalities can help detect any recurrence or metastasis of the cancer and provide valuable information for further treatment decisions. Additionally, colonoscopy or flexible sigmoidoscopy may be performed to examine the remaining colon for the presence of new polyps or tumors.

(4) Laboratory investigations, including blood tests, are essential components of post-operative follow-up. These tests can help assess the patient's overall health status, monitor tumor markers (such as carcinoembryonic antigen or CEA levels), and evaluate organ function. Blood tests may also be used to detect any abnormal findings that require further investigation or intervention, such as liver function tests to assess for potential metastasis to the liver.

(5) Follow-up visits may also involve discussions regarding lifestyle modifications, dietary recommendations, and psychological support. Patients may be advised on maintaining a healthy lifestyle, including regular exercise and a balanced diet. Psychological support may be offered to address the emotional challenges faced by patients during the recovery phase and to assist with any anxiety or depression related to the cancer diagnosis.

Rep2:

(1) After colon cancer surgery, follow-up and reexamination play a crucial role in monitoring the patient's recovery, detecting any potential recurrence, and managing long-term effects. Typically, a multidisciplinary approach involving the surgical team, medical oncologists, and gastroenterologists is adopted for comprehensive care.

(2) One common method of follow-up and reexamination is regular surveillance through imaging tests such as computed tomography (CT) scans or magnetic resonance imaging (MRI). These imaging techniques help assess the surgical site, nearby lymph nodes, and other relevant areas for signs of cancer recurrence or metastasis. The frequency and duration of these scans may vary depending on the stage of the cancer, the patient's overall health, and other individual factors.

(3) Another important aspect of follow-up care is periodic blood tests to monitor tumor markers such as carcinoembryonic antigen (CEA). Elevated levels of CEA may indicate a recurrence or metastasis of colon cancer. Regular CEA testing helps detect potential issues early on and allows for timely intervention.

(4) In addition to imaging and blood tests, regular physical examinations are essential for assessing the patient's overall health and identifying any potential concerns. These examinations may involve assessing the surgical site, palpating the abdomen for abnormalities, and evaluating the patient's general well-being.

(5) Follow-up visits also provide an opportunity for discussing any persistent symptoms or side effects the patient may be experiencing. These discussions can address issues like changes in bowel habits, gastrointestinal discomfort, or nutritional concerns. The healthcare team can provide appropriate guidance, support, and referrals to specialists, if necessary.

(6) Psychological support and counseling are also vital during follow-up and reexamination. Dealing with the diagnosis and treatment of colon cancer can be emotionally challenging. Regular check-ins with a healthcare professional or referral to a counselor or support group can help patients cope with anxiety, depression, or other psychological effects.

(7) The frequency and duration of follow-up visits may vary depending on individual circumstances. Generally, the first few years after surgery require more frequent follow-up, gradually transitioning to less frequent visits as time goes on. However, the exact follow-up schedule should be determined by the patient's medical team based on factors such as the stage of cancer, treatment received, and overall health status.

**The Radiation Therapy**

**1, Is radiation therapy necessary for rectal cancer? Under what circumstances is radiation therapy needed?**

(1) Radiation therapy is often an essential component in the treatment of rectal cancer. It is used to destroy cancer cells, prevent their growth, and reduce the risk of cancer recurrence. Radiation therapy involves the use of high-energy X-rays or other forms of radiation to target and kill cancer cells. It is typically administered before surgery (neoadjuvant radiation therapy) or after surgery (adjuvant radiation therapy). Neoadjuvant radiation therapy helps shrink the tumor, making it easier to remove during surgery. Adjuvant radiation therapy is used to eliminate any remaining cancer cells after surgery and reduce the likelihood of cancer recurrence.

(2) Radiation therapy is particularly beneficial for rectal cancer patients when the tumor is locally advanced or has spread to nearby lymph nodes. It helps to improve the chances of successful surgery by reducing the size of the tumor, increasing the likelihood of complete tumor removal, and minimizing the risk of cancer cells being left behind. Additionally, radiation therapy can be used as the primary treatment for some patients who are not suitable candidates for surgery due to various reasons, such as advanced age or underlying health conditions.

(3) In cases where rectal cancer has penetrated through the wall of the rectum or has spread to nearby organs, radiation therapy becomes crucial. It helps to destroy cancer cells in the rectum and surrounding areas, reducing the risk of cancer spreading further. Furthermore, radiation therapy is commonly used when there is a high risk of cancer recurrence based on the characteristics of the tumor, such as aggressive growth, involvement of lymph nodes, or certain molecular features. By targeting and eliminating cancer cells, radiation therapy plays a vital role in improving long-term outcomes and overall survival rates.

(4) Depending on the specific circumstances and individual patient factors, radiation therapy for rectal cancer may be recommended in different situations. The decision to use radiation therapy is typically made by a multidisciplinary team of healthcare professionals, including oncologists, surgeons, and radiation oncologists, who assess the stage and extent of the cancer, the overall health of the patient, and the potential benefits and risks of treatment. They consider factors such as tumor size, involvement of nearby structures, lymph node status, and patient preferences to determine whether radiation therapy should be incorporated into the treatment plan.

Rep2:

(1) Radiation therapy is often a necessary component of treatment for rectal cancer. It is commonly used in conjunction with surgery and sometimes with chemotherapy, forming a multidisciplinary approach. The primary goal of radiation therapy is to eliminate or control cancer cells in the rectal area. It involves the use of high-energy radiation beams to target and destroy cancer cells, either before surgery to shrink the tumor or after surgery to kill any remaining cancer cells.

(2) Radiation therapy may be needed in cases where the rectal cancer is locally advanced, meaning it has grown into nearby structures or lymph nodes. This is determined through diagnostic tests and imaging scans. If the tumor is large, invading surrounding tissues, or has spread to nearby lymph nodes, radiation therapy is typically recommended. It helps to reduce the size of the tumor, making it more manageable for surgery, or it can be used after surgery to lower the risk of recurrence.

(3) Another circumstance where radiation therapy is necessary is when the tumor is located in the lower part of the rectum, close to the anus. In these cases, surgery alone may lead to significant impairment of bowel function or require the removal of the entire rectum (total mesorectal excision). Radiation therapy can be used prior to surgery to shrink the tumor and increase the chances of preserving the rectum and sphincter function, thus preserving bowel continence.

(4) Additionally, radiation therapy is commonly recommended when the cancer has penetrated through the rectal wall and infiltrated surrounding tissues, such as the pelvic sidewall or adjacent organs like the bladder or prostate in men or the uterus in women. By delivering radiation directly to these areas, the treatment aims to destroy cancer cells and prevent their spread.

**2, Are the side effects of radiotherapy significant? Can radiotherapy be avoided?**

(1) Radiotherapy is a common treatment for cancer that uses high-energy radiation to target and destroy cancer cells. While radiotherapy can be highly effective in treating cancer, it does come with potential side effects. The nature and severity of these side effects depend on various factors such as the type and location of the cancer, the dose of radiation, and individual patient factors. Some common side effects of radiotherapy include fatigue, skin changes, hair loss, nausea, and changes in appetite. These side effects are generally temporary and tend to improve after treatment completion.

(2) It is important to note that the side effects of radiotherapy are carefully managed by the healthcare team to minimize their impact on patients' quality of life. Radiation oncologists and other specialists work closely with patients to develop personalized treatment plans that aim to deliver the necessary dose of radiation while minimizing damage to healthy tissues. Advanced techniques like intensity-modulated radiation therapy (IMRT) and proton therapy allow for more precise targeting of cancer cells, reducing the risk of side effects. In some cases, radiotherapy may be combined with other treatments, such as surgery or chemotherapy, to optimize the overall treatment outcome.

(3) While radiotherapy is a valuable and effective treatment for many types of cancer, it may not be suitable or necessary for every patient. The decision to use radiotherapy depends on various factors, including the type and stage of cancer, the location of the tumor, and the overall health of the patient. In certain situations, alternative treatment options such as surgery or systemic therapies like chemotherapy or targeted therapy may be considered. The final treatment plan is determined through a comprehensive evaluation by a multidisciplinary team of healthcare professionals, taking into account the specific needs and circumstances of each patient.

(4) However, it's important to emphasize that the decision to avoid radiotherapy should be made in consultation with medical professionals. Radiotherapy can be a crucial component of cancer treatment, providing local control and potential cure in many cases. If there are concerns about the potential side effects of radiotherapy, patients are encouraged to have open and honest discussions with their healthcare team. The team can provide detailed information about the potential benefits and risks of radiotherapy, as well as discuss strategies to manage and mitigate side effects. Ultimately, the goal is to achieve the best possible treatment outcome while ensuring the patient's well-being and quality of life.

Rep2:

(1) Radiotherapy can have significant side effects, although they vary depending on the individual and the area being treated. Common side effects include fatigue, skin changes (such as redness, dryness, and sensitivity), hair loss in the treated area, and temporary or permanent changes in the function of nearby organs. Some people may also experience nausea, vomiting, and diarrhea, particularly if the abdomen or pelvis is treated. However, it is important to note that not everyone experiences all these side effects, and they can be managed or minimized with appropriate medical care and support.

(2) In many cases, radiotherapy cannot be completely avoided when it is the recommended treatment for a specific medical condition. Radiotherapy is commonly used as a primary or adjuvant treatment for various types of cancer, where it is crucial for destroying cancer cells and preventing their growth or recurrence. However, the decision to undergo radiotherapy is always made after careful consideration of the potential benefits and risks. The medical team evaluates the individual's specific condition, overall health, and treatment goals to determine the most suitable course of action.

(3) Alternative treatment options may be considered to avoid or minimize the need for radiotherapy in certain cases. These options depend on the specific condition and individual circumstances. In some situations, surgery may be a viable alternative to remove a localized tumor without the need for radiation. Additionally, advancements in medical technology and research have led to the development of targeted therapies, immunotherapies, and other innovative treatments that may offer alternative approaches for specific types of cancer. It is essential to consult with a healthcare professional who can provide guidance on the available options and their potential benefits and risks.

(4) Ultimately, the decision to proceed with radiotherapy or explore alternative treatments depends on several factors, including the type and stage of the disease, the potential benefits of radiotherapy, the individual's overall health and preferences, and the expertise and recommendations of the medical team. Open communication with healthcare professionals is crucial to fully understand the potential side effects of radiotherapy and explore all available options to make an informed decision about the most appropriate treatment approach.

**3, What adverse reactions may occur during radiotherapy for rectal cancer? How should they be managed?**

(1) One potential adverse reaction during radiotherapy for rectal cancer is skin irritation and radiation dermatitis. The skin in the treated area may become red, dry, and itchy, similar to a sunburn. This reaction typically occurs within a few weeks of starting treatment and can vary in severity. To manage radiation dermatitis, patients are advised to keep the treated area clean and dry, avoid scratching or rubbing the skin, and use gentle, non-irritating skincare products. Applying prescribed topical creams or ointments recommended by the healthcare team can help soothe the skin and alleviate discomfort.

(2) Another possible adverse reaction is gastrointestinal toxicity. Radiotherapy to the rectal area can cause inflammation and damage to the lining of the intestines, leading to symptoms such as diarrhea, abdominal cramping, and rectal discomfort. To manage gastrointestinal toxicity, patients may be advised to follow a specific diet that minimizes irritation to the digestive system, such as avoiding spicy foods, caffeine, and high-fiber foods. Medications, such as anti-diarrheal drugs and analgesics, can be prescribed to relieve symptoms. In severe cases, temporary interruption of radiotherapy may be necessary to allow the intestines to heal.

(3) Hematologic toxicity is another potential adverse reaction of radiotherapy for rectal cancer. The radiation can affect the bone marrow's ability to produce blood cells, leading to a decrease in red and white blood cells and platelets. This can result in anemia, increased risk of infections, and bleeding problems. Managing hematologic toxicity involves regular monitoring of blood counts during treatment. In some cases, blood transfusions or growth factor medications may be administered to maintain adequate blood cell levels. Treatment interruptions or adjustments may also be necessary to allow for recovery of the bone marrow.

(4) Bladder and urinary toxicity is another concern during radiotherapy for rectal cancer. The bladder may become irritated, leading to urinary frequency, urgency, and discomfort. In some cases, radiation can cause inflammation and scarring in the bladder, leading to long-term urinary problems. Management of bladder and urinary toxicity involves drinking plenty of fluids to dilute the urine and minimize irritation, avoiding bladder irritants such as caffeine and alcohol, and practicing good hygiene. Medications to relieve urinary symptoms, such as antispasmodics, may be prescribed. In severe cases, additional interventions, such as bladder irrigation or cystoscopy, may be necessary.

Rep2:

(1) One potential adverse reaction during radiotherapy for rectal cancer is acute skin reactions. The skin in the radiation field may become red, itchy, and sensitive. In severe cases, it can lead to moist desquamation, where the skin becomes moist and starts to peel off. These reactions occur due to the effect of radiation on the skin cells.

(2) Another adverse reaction is gastrointestinal toxicity. The radiation can cause inflammation and damage to the lining of the digestive tract, leading to symptoms such as diarrhea, nausea, vomiting, and abdominal cramping. These gastrointestinal side effects can affect the patient's quality of life and may require supportive care measures.

(3) Fatigue is a common side effect of radiotherapy for rectal cancer. The treatment can cause a general feeling of tiredness and lack of energy. Fatigue can be both physical and mental, and it may persist for several weeks or months after the completion of treatment. Proper rest, maintaining a balanced diet, and engaging in light physical activity can help manage fatigue.

(4) In some cases, radiation therapy can also lead to long-term complications such as bowel dysfunction. This can manifest as changes in bowel habits, urgency, frequency, or difficulty in controlling bowel movements. Dietary modifications, medication, and pelvic floor exercises can be beneficial in managing these symptoms. In rare cases, patients may experience late radiation-induced complications, such as pelvic bone fractures or secondary malignancies. Regular follow-up visits with the healthcare team are important to monitor and address any potential long-term adverse effects.

**4, Will there be a second tumor after receiving radiotherapy?**

(1) It is possible for a second tumor to develop after receiving radiotherapy, although the risk varies depending on several factors. Radiotherapy uses high-energy radiation to target and kill cancer cells, but it can also affect healthy cells in the surrounding area. In some cases, the radiation can cause genetic mutations in normal cells, potentially leading to the development of a second tumor over time. The likelihood of this occurring depends on factors such as the dosage and duration of radiation therapy, the specific area being treated, and the individual's overall health and genetic predisposition to cancer.

(2) While radiotherapy is designed to target and destroy cancer cells, it can occasionally have unintended effects on nearby healthy cells. Radiation can cause DNA damage in normal cells, which may result in mutations that lead to the development of a second tumor. However, it's important to note that the risk of developing a second tumor after radiotherapy is generally low. Advances in radiation therapy techniques and technologies aim to minimize these risks and improve treatment precision, reducing the likelihood of subsequent tumors.

(3) The possibility of developing a second tumor after radiotherapy exists due to the potential for radiation-induced genetic mutations in healthy cells. However, it's essential to understand that radiotherapy is a highly effective treatment for cancer, and the benefits often outweigh the risks. The development of a second tumor is relatively uncommon, and the majority of patients who undergo radiotherapy do not experience this complication. Close monitoring and follow-up care by medical professionals can help detect any potential signs of a second tumor early, enabling timely intervention and treatment.

(4) The risk of developing a second tumor following radiotherapy is generally considered to be low. While radiation can cause genetic mutations in healthy cells, leading to the development of a second tumor, it's important to recognize that the benefits of radiotherapy in treating the primary tumor are substantial. Medical professionals take numerous precautions to minimize the potential side effects of radiation and optimize its effectiveness. By carefully planning the treatment, targeting the radiation beams precisely, and using the appropriate dosage, the risk of developing a second tumor can be significantly reduced. Regular medical check-ups and surveillance after radiotherapy are crucial to detect any potential recurrence or new tumors early.

Rep2:

(1) It is possible for a second tumor to occur after receiving radiotherapy, but the likelihood depends on various factors. Radiotherapy is a treatment that uses high-energy radiation to target and destroy cancer cells. While it is designed to kill cancer cells, there is a small risk that radiation exposure may also damage healthy cells in the surrounding area. This damage can potentially lead to the development of a new tumor later on. However, it is important to note that the risk of developing a second tumor due to radiotherapy is generally low and varies depending on the specific circumstances of the individual.

(2) The risk of developing a second tumor after radiotherapy can depend on the dose and area of radiation treatment. Higher doses of radiation and larger treatment areas generally carry a slightly higher risk. Additionally, the type of cancer being treated can also influence the likelihood of a second tumor. Some types of cancer have a higher inherent risk of developing multiple tumors. Age, genetic factors, and lifestyle choices, such as smoking or exposure to certain chemicals, can further contribute to the risk of developing a second tumor. However, it is essential to remember that even with these factors, the overall risk remains relatively low.

(3) Healthcare professionals take several precautions to minimize the risk of a second tumor occurring after radiotherapy. Treatment plans are carefully designed to target the tumor while minimizing exposure to healthy tissues. Advanced imaging techniques and computer simulations help in precisely delivering radiation to the intended area, reducing the potential for damage to healthy cells. Additionally, regular follow-up appointments and imaging scans are conducted to monitor for any signs of new tumor growth or other potential complications. These measures aim to ensure the best possible outcome for patients receiving radiotherapy.

(4) The risk of developing a second tumor after radiotherapy should be considered in the context of the potential benefits of the treatment. Radiotherapy is an important and effective treatment option for many types of cancer. It can significantly improve survival rates and provide long-term control or even cure for certain cancers. The risk of a second tumor should be weighed against the potential benefits of radiotherapy, taking into account the individual's specific circumstances, cancer type, and stage. Healthcare providers will discuss these factors with patients and provide personalized information and recommendations to help make informed treatment decisions.

**5,** **What are the commonly used radiotherapy modes and techniques for rectal cancer?**

(1) One commonly used radiotherapy mode for rectal cancer is external beam radiation therapy (EBRT). In EBRT, high-energy X-ray beams are delivered from a machine outside the body to target and destroy cancer cells in the rectal area. This technique is typically performed on an outpatient basis over several weeks, with daily treatment sessions lasting a few minutes each.

(2) Another frequently employed technique is intensity-modulated radiation therapy (IMRT). IMRT is an advanced form of EBRT that allows for precise modulation of the radiation beam intensity. By using computer-controlled linear accelerators, IMRT can deliver radiation doses that conform closely to the shape of the tumor while minimizing exposure to surrounding healthy tissues. This approach helps to reduce side effects and improve treatment outcomes.

(3) Brachytherapy, also known as internal radiation therapy, is another mode used in the treatment of rectal cancer. This technique involves placing a radioactive source directly into or near the tumor. In the case of rectal cancer, brachytherapy can be administered through an applicator inserted into the rectum. The radioactive material emits radiation that targets the tumor from within, delivering a high dose to the cancer cells while minimizing exposure to nearby organs.

(4) Stereotactic body radiation therapy (SBRT) is an emerging technique for treating rectal cancer. SBRT utilizes advanced imaging and precise targeting to deliver high doses of radiation in a few treatment sessions, usually completed within a week. This technique is particularly beneficial for small, localized rectal tumors and may offer an alternative to surgery for some patients.

Rep2:

(1) External Beam Radiation Therapy (EBRT): This is a commonly used radiotherapy mode for rectal cancer. It involves directing high-energy X-rays or protons from outside the body to the tumor area. EBRT is typically delivered in daily treatment sessions over several weeks, aiming to destroy cancer cells and shrink tumors.

(2) Intensity-Modulated Radiation Therapy (IMRT): IMRT is a specialized technique used in radiotherapy for rectal cancer. It delivers precise radiation doses to the tumor while sparing nearby healthy tissues. IMRT uses computer-controlled linear accelerators to shape the radiation beams and adjust their intensity based on the tumor's size and shape.

(3) Image-Guided Radiation Therapy (IGRT): IGRT is a radiotherapy technique that uses advanced imaging technology to guide treatment delivery. It involves obtaining real-time images of the tumor immediately before or during each treatment session. These images help ensure accurate targeting of the radiation to the tumor, accounting for any changes in its position or shape.

(4) Stereotactic Body Radiation Therapy (SBRT): SBRT is a highly precise radiotherapy technique used for treating small, localized rectal tumors. It delivers a high dose of radiation in a few treatment sessions using sophisticated imaging and precise tumor localization techniques. SBRT is advantageous for patients who cannot tolerate long treatment courses or surgical interventions.

(5) Brachytherapy: Brachytherapy involves placing radioactive sources directly into or near the tumor. For rectal cancer, intraluminal brachytherapy may be used, where a catheter or applicator is inserted into the rectum to deliver a targeted radiation dose. This technique allows for precise delivery of radiation while minimizing exposure to surrounding healthy tissues.

(6) Preoperative or neoadjuvant radiotherapy: Neoadjuvant radiotherapy refers to delivering radiation therapy before surgery. It aims to shrink the tumor, increase the chances of complete surgical removal, and potentially improve long-term outcomes. Neoadjuvant radiotherapy is often used for locally advanced rectal cancer to facilitate surgical resection.

(7) Adjuvant radiotherapy: Adjuvant radiotherapy is given after surgery to eliminate any remaining cancer cells and reduce the risk of recurrence. It is commonly used for rectal cancer cases with certain high-risk features or when the tumor is not completely removed during surgery.

**6,** **What are the argon-helium knife, gamma knife, and proton knife? What are the differences?**

(1) The argon-helium knife is a medical device used for precision surgical procedures, particularly in the field of neurosurgery. It utilizes a focused beam of argon and helium ions to precisely cut and remove tissue. The knife delivers a concentrated dose of energy to the targeted area, effectively destroying the tissue without causing excessive damage to the surrounding healthy tissue.

(2) The gamma knife, on the other hand, is a non-invasive radiosurgery device used for treating brain tumors and other neurological conditions. It uses multiple intersecting beams of highly focused gamma rays to deliver a high dose of radiation to the target area. The gamma knife is often preferred for treating small to medium-sized tumors located deep inside the brain, as it can precisely target the abnormal tissue while minimizing radiation exposure to healthy surrounding tissues.

(3) The proton knife, also known as proton therapy or proton beam therapy, is a form of radiation treatment that utilizes protons, which are charged particles, to target and destroy cancerous cells. Protons have a unique property that allows them to deposit the majority of their energy directly at the intended depth within the body, minimizing damage to the tissues traversed by the beam. This precision enables doctors to deliver higher doses of radiation to the tumor while reducing the radiation exposure to nearby healthy tissues.

(4) It's important to note that while all three techniques mentioned above involve the treatment of medical conditions using focused energy, they differ in the type of energy they employ. The argon-helium knife relies on ion beams, the gamma knife uses gamma rays, and the proton knife utilizes proton beams. Additionally, the argon-helium knife is a surgical tool, whereas the gamma knife and proton knife are non-invasive treatment modalities. Each technique has its own advantages and applications, and the choice of treatment depends on the specific condition and the recommendation of the medical professionals involved.

Rep2:

(1) The argon-helium knife is a medical device used in radiosurgery, a non-invasive technique for treating certain conditions in the brain and other parts of the body. It utilizes a beam of radiation composed of argon and helium gas to target and destroy abnormal or diseased tissue. The high-energy radiation is delivered precisely to the target area while minimizing damage to surrounding healthy tissue. The argon-helium knife is typically used to treat small to medium-sized lesions or tumors.

(2) The gamma knife is a specialized form of radiosurgery that uses gamma rays to treat various conditions in the brain, particularly brain tumors and vascular malformations. Unlike traditional surgery, the gamma knife does not involve making an incision. Instead, it employs multiple beams of highly focused gamma radiation to converge on the target area, delivering a high dose of radiation precisely to the lesion while sparing nearby healthy tissue. The gamma knife is known for its accuracy and effectiveness in treating brain disorders.

(3) The proton knife, also known as proton therapy or proton beam therapy, is a type of radiation therapy that utilizes protons, which are charged particles, to treat cancer. Proton therapy is particularly suitable for tumors located near vital structures or in pediatric patients, as it allows for precise targeting while minimizing radiation exposure to surrounding healthy tissue. The proton knife works by accelerating protons to high speeds and directing them precisely at the tumor, where they deposit most of their energy, effectively destroying cancer cells.

**7, What are the conditions that can be treated with heavy ion radiotherapy?**

(1) Heavy ion radiotherapy, also known as particle therapy or hadron therapy, is a specialized form of radiation therapy that utilizes high-energy charged particles, such as protons or carbon ions, to treat certain types of cancer. It can be particularly beneficial for tumors that are resistant to conventional radiation therapy or located in critical areas of the body where preserving surrounding healthy tissues is crucial.

(2) One condition that can be treated with heavy ion radiotherapy is prostate cancer. The precision of heavy ion beams allows for highly targeted delivery of radiation to the tumor while minimizing damage to the surrounding organs, such as the bladder and rectum. This therapy can be effective in controlling prostate cancer and reducing side effects compared to other treatment options.

(3) Another condition that can benefit from heavy ion radiotherapy is certain types of brain tumors, including gliomas. The precise nature of heavy ion beams enables more accurate targeting of tumor cells, which is particularly advantageous for tumors located near critical structures within the brain. By delivering a higher dose of radiation to the tumor while sparing healthy tissue, heavy ion radiotherapy can improve treatment outcomes and minimize neurological side effects.

(4) Head and neck cancers can also be treated with heavy ion radiotherapy. These cancers often occur in areas with complex anatomy, making it challenging to administer radiation without affecting critical structures like the salivary glands, vocal cords, or optic nerves. Heavy ion beams can be finely adjusted to conform to the shape of the tumor, delivering radiation precisely to the cancerous cells while minimizing damage to surrounding healthy tissues. This can help preserve important functions and improve the quality of life for patients.

(5) Additionally, pediatric tumors can be treated with heavy ion radiotherapy. Children's bodies are more sensitive to radiation, and their developing organs and tissues are at a higher risk of long-term damage. Heavy ion therapy offers a way to deliver radiation precisely to pediatric tumors, reducing the potential for late effects and minimizing the impact on the child's growth and development.

(6) Other conditions that may benefit from heavy ion radiotherapy include certain types of lung cancer, liver cancer, and sarcomas. The specific choice of heavy ion therapy depends on various factors, including tumor location, size, and stage, as well as the patient's overall health. It is important to consult with a radiation oncologist to determine if heavy ion radiotherapy is an appropriate treatment option for a particular condition.

Rep2:

(1) Heavy ion radiotherapy, also known as particle therapy or hadron therapy, is a form of radiation therapy that utilizes high-energy charged particles, such as protons or carbon ions, to treat various conditions. It is particularly effective in treating certain types of tumors and cancers. The unique properties of heavy ions allow for precise targeting of tumor cells while minimizing damage to surrounding healthy tissues. This therapy can be used to treat both primary tumors and metastatic cancers, offering a promising alternative to conventional radiotherapy.

(2) One condition that can be treated with heavy ion radiotherapy is prostate cancer. Prostate cancer is a common malignancy in men, and the use of heavy ions in radiotherapy can provide improved outcomes. By delivering high doses of radiation precisely to the prostate tumor, heavy ion therapy can effectively destroy cancer cells while minimizing damage to nearby critical structures, such as the bladder and rectum. This treatment modality is particularly advantageous for localized prostate cancer cases, where the goal is to eradicate the tumor while preserving quality of life.

(3) Another condition that can be treated with heavy ion radiotherapy is pediatric tumors. Children with cancer often face unique challenges due to the long-term side effects of traditional radiation therapy. Heavy ion radiotherapy offers a promising solution by delivering highly focused radiation to pediatric tumors while reducing the exposure of healthy tissues to radiation. This precision targeting helps minimize the risk of long-term complications, such as growth impairment and secondary malignancies, improving the overall quality of life for pediatric cancer survivors.

(4) Head and neck tumors can also be treated with heavy ion radiotherapy. The complex anatomy of the head and neck region makes it challenging to deliver radiation effectively while sparing critical structures, such as the salivary glands and optic nerves. Heavy ions can be precisely directed to the tumor, enabling a high dose to be delivered while minimizing damage to surrounding healthy tissues. This approach has shown promising results in treating various head and neck cancers, including nasopharyngeal carcinoma and skull base tumors, offering improved tumor control rates and reduced side effects.

(5) Liver cancer, specifically hepatocellular carcinoma (HCC), can be treated with heavy ion radiotherapy as well. The liver is a vital organ, and conventional radiation therapy techniques may be limited in their ability to deliver an effective dose while sparing the surrounding liver tissue. Heavy ion radiotherapy provides an advantage by precisely targeting the tumor while minimizing radiation exposure to healthy liver tissue. This allows for higher doses of radiation to be delivered, increasing the chances of tumor control and potentially improving patient outcomes.

**The Interventional Therapy**

**1, An 80-year-old elderly patient with colorectal cancer and obstruction. Is surgery not an option?**

(1) In general, the decision of whether surgery is an option for an 80-year-old elderly patient with colorectal cancer and obstruction depends on various factors. Advanced age alone does not automatically exclude someone from undergoing surgery. The overall health status and functional capacity of the patient are crucial considerations. If the patient is otherwise in good health, has a reasonable life expectancy, and can tolerate surgery, it may be considered as a viable treatment option. The decision-making process would involve a comprehensive assessment by a multidisciplinary team, including surgeons, oncologists, anesthesiologists, and geriatric specialists.

(2) Another general consideration is the stage and extent of the colorectal cancer. If the cancer is localized and hasn't spread to distant organs, surgical resection may offer the best chance for a curative outcome. However, if the cancer is advanced or has metastasized, surgery may be less likely to provide significant benefits in terms of survival or quality of life. In such cases, alternative treatment approaches, such as palliative care or chemotherapy, may be more appropriate to relieve symptoms and manage the disease.

(3) Additionally, the presence of obstruction complicates the situation. Obstructions in the colon can cause severe symptoms, including pain, nausea, vomiting, and bowel dysfunction. Surgical intervention may be necessary to relieve the obstruction and restore normal bowel function. However, the decision to proceed with surgery would still depend on the patient's overall health and the extent of the cancer. In some cases, a temporary stoma or bypass surgery may be considered to alleviate symptoms and improve the patient's quality of life.

(4) Ultimately, the decision regarding surgery for an 80-year-old patient with colorectal cancer and obstruction requires a comprehensive evaluation of the patient's overall health, cancer stage, and individual circumstances. It is essential to involve the patient in the decision-making process and consider their preferences and goals of care. The input of a multidisciplinary team, including specialists in geriatrics, oncology, and surgery, can help guide the treatment plan to optimize outcomes and ensure the patient's well-being.

Rep2:

(1) In general, surgery is often considered the primary treatment option for colorectal cancer. However, in the case of an 80-year-old elderly patient with colorectal cancer and obstruction, the decision to proceed with surgery may depend on various factors. Age alone is not a definitive contraindication for surgery, but it can be a consideration due to the potential impact on overall health and post-operative recovery. The patient's overall health status, including any pre-existing medical conditions and functional abilities, should be thoroughly evaluated. Additionally, the extent and stage of the cancer, the presence of metastases, and the potential benefits and risks of the surgery should all be taken into account. The patient's preferences, goals, and quality of life considerations should also be considered when making a treatment decision.

(2) Generally, surgery may not be considered an option for an 80-year-old elderly patient with colorectal cancer and obstruction if the risks associated with the procedure outweigh the potential benefits. As age advances, individuals may have a higher likelihood of experiencing age-related complications, reduced physiological reserve, and decreased tolerance for surgery and anesthesia. Moreover, the presence of other significant medical conditions, such as heart disease or respiratory problems, can further increase the risks associated with surgery. In such cases, alternative treatment options may be explored, such as nonsurgical interventions like stenting or the use of chemotherapy or radiation therapy to manage symptoms and slow down the progression of the cancer.

(3) Generally, the decision to forgo surgery in an 80-year-old elderly patient with colorectal cancer and obstruction might be made if the cancer is advanced, has metastasized to distant organs, or if the patient's overall health status is too poor to tolerate the surgical procedure. In such situations, the focus of the treatment may shift towards palliative care, which aims to relieve symptoms, improve quality of life, and provide supportive care. This can involve the use of medications, pain management techniques, dietary adjustments, and other supportive measures to alleviate symptoms associated with the obstruction and cancer. A multidisciplinary approach involving the patient, their family, and healthcare providers is crucial in determining the most appropriate and personalized treatment plan.

(4) The decision regarding whether or not surgery is an option for an 80-year-old elderly patient with colorectal cancer and obstruction should be made on an individual basis, considering the patient's overall health, the extent and stage of the cancer, and the potential risks and benefits of surgery. It is important to involve a multidisciplinary team of healthcare professionals, including surgeons, oncologists, and geriatric specialists, to assess the patient's specific situation comprehensively. The team should weigh the potential benefits of surgery, such as tumor removal and potential cure, against the risks, including post-operative complications and reduced quality of life. Shared decision-making, considering the patient's preferences and goals, is vital to ensure the treatment plan aligns with the patient's values and desires.

**2, Can a stent be placed in the intestine?**

(1) Yes, a stent can be placed in the intestine. Intestinal stenting is a minimally invasive procedure used to treat various conditions that cause narrowing or blockage of the intestinal lumen. The stent is a tubular device made of metal or plastic that can be inserted into the intestine to open up the narrowed segment and restore normal bowel function. It acts as a scaffold to keep the intestine open, allowing the passage of food, fluids, and gas.

(2) Intestinal stenting is typically performed under X-ray guidance or endoscopic guidance. During the procedure, the stent is inserted through the mouth or anus, depending on the location of the intestinal obstruction. The stent is carefully positioned in the narrowed area of the intestine using specialized instruments and imaging techniques. Once in place, the stent expands to its full diameter, pushing aside the surrounding tissue and creating a clear passage for the flow of intestinal contents.

(3) The use of intestinal stents can be beneficial in various clinical scenarios. It is commonly employed as a palliative measure for patients with advanced cancer-causing bowel obstruction. The stent helps relieve symptoms such as abdominal pain, nausea, and vomiting, allowing patients to eat and drink more comfortably. In some cases, intestinal stenting can also be used as a bridge to surgery, providing temporary relief and improving the patient's nutritional status before a definitive surgical intervention.

(4) Intestinal stenting has several advantages over traditional surgical procedures. It is a less invasive option, typically requiring only sedation or local anesthesia instead of general anesthesia. The recovery time is usually shorter, and there is a reduced risk of complications compared to open surgery. Additionally, stenting can be performed in patients who are not suitable candidates for surgery due to their overall health condition or other medical reasons.

(5) However, it is important to note that intestinal stenting may not be suitable for all cases. The decision to use a stent depends on various factors, including the location and extent of the intestinal obstruction, the underlying cause, and the overall health status of the patient. A thorough evaluation by a medical professional is necessary to determine the most appropriate treatment approach, whether it involves stenting, surgery, or other interventions.

Rep2:

(1) Yes, a stent can be placed in the intestine. Intestinal stenting is a medical procedure used to treat various conditions that affect the intestines, such as bowel obstruction or strictures. A stent is a tubular device typically made of metal or plastic that is inserted into the intestine to help maintain its patency and restore the normal flow of digestive contents. The stent acts as a scaffold, keeping the intestine open and allowing the passage of food and stool.

(2) Intestinal stenting is a minimally invasive procedure that is often performed using endoscopic techniques. During the procedure, a flexible endoscope is inserted through the mouth or anus and guided into the intestine. Once the site of obstruction or narrowing is reached, a stent is advanced through the endoscope and deployed at the targeted location. The stent expands to its full size, exerting outward pressure on the intestinal walls and creating an open pathway for the passage of fluids and solids.

(3) The placement of a stent in the intestine can provide relief from symptoms associated with intestinal obstruction or strictures. It can help to alleviate pain, reduce bowel obstruction, and improve the patient's ability to eat and digest food. Intestinal stenting is considered a less invasive alternative to surgical interventions and can be performed on an outpatient basis in many cases, allowing for a faster recovery time and reduced hospital stay.

(4) The use of intestinal stents may be temporary or permanent, depending on the underlying condition being treated. In some cases, the stent is designed to be removable after the obstruction or stricture has resolved or improved. In other situations, such as in patients with advanced cancer, the stent may be placed permanently to provide long-term relief and improve the quality of life.

(5) Intestinal stenting is generally considered safe and effective, but it does carry some risks and potential complications. These can include stent migration, perforation or damage to the intestine, infection, bleeding, or allergic reactions to the stent material. The procedure should be performed by experienced healthcare professionals who have expertise in endoscopy and stent placement to minimize these risks and ensure optimal outcomes for the patient.

**3, Is gastric decompression effective for low-intestinal obstruction caused by colorectal cancer?**

1. Gastric decompression is not typically effective for low-intestinal obstruction caused by colorectal cancer. Gastric decompression refers to the removal of gas and fluid from the stomach using a nasogastric tube. It can provide temporary relief of symptoms such as nausea, vomiting, and abdominal distension by reducing gastric pressure. However, in cases of low-intestinal obstruction caused by colorectal cancer, the blockage occurs further down the gastrointestinal tract, beyond the stomach. Therefore, decompressing the stomach alone does not address the underlying cause of the obstruction and does not provide a definitive treatment.

2. The main goal in managing low-intestinal obstruction caused by colorectal cancer is to relieve the blockage and restore the normal flow of intestinal contents. This is usually achieved through surgical intervention, such as a colectomy or bowel resection, depending on the extent and location of the tumor. Surgery allows for the removal of the obstructing tumor and any affected portions of the intestine, allowing for restoration of the normal bowel function. In some cases, additional treatments like chemotherapy or radiation therapy may be used before or after surgery to shrink the tumor or prevent its recurrence.

3. Non-surgical interventions such as stenting or placement of a self-expanding metal stent may be considered in certain cases of low-intestinal obstruction caused by colorectal cancer. A stent can be inserted into the narrowed or blocked segment of the intestine, providing a temporary pathway for the passage of stool and relieving the obstruction. This procedure can be less invasive than surgery and may be used as a palliative measure for patients who are not suitable candidates for surgery or for those who require temporary relief of symptoms before undergoing definitive surgical treatment.

4. It is important to note that the most appropriate treatment for low-intestinal obstruction caused by colorectal cancer depends on various factors, including the patient's overall health, the extent and location of the tumor, and the presence of any metastasis. Therefore, it is crucial for patients to consult with a healthcare professional, such as a gastroenterologist or a colorectal surgeon, who can evaluate their specific condition and recommend the most suitable treatment approach.

Rep2:

1. Gastric decompression refers to the process of removing excess air and fluid from the stomach using a nasogastric tube. In the case of low-intestinal obstruction caused by colorectal cancer, gastric decompression can provide temporary relief of symptoms. It helps alleviate abdominal distention, nausea, and vomiting by reducing the pressure in the stomach. By removing the accumulated gastric contents, it may also help prevent aspiration and improve patient comfort.

2. Gastric decompression does not directly address the underlying cause of low-intestinal obstruction, which is colorectal cancer. It is primarily a supportive measure aimed at providing symptomatic relief. It can be useful in managing acute symptoms such as distention and vomiting, allowing patients to tolerate oral intake better and potentially avoiding surgical intervention in certain cases. However, it is important to note that gastric decompression does not treat or cure the colorectal cancer itself.

3. While gastric decompression can be helpful in relieving immediate symptoms associated with low-intestinal obstruction caused by colorectal cancer, it is not a definitive treatment. The underlying cause, colorectal cancer, requires appropriate management, which usually involves a multidisciplinary approach. Treatment options for colorectal cancer may include surgery, chemotherapy, radiation therapy, or a combination of these modalities, depending on the stage and characteristics of the tumor. Therefore, it is essential to consult with a healthcare professional to determine the most appropriate treatment strategy for the colorectal cancer itself.

4. Gastric decompression can be an effective temporary measure to relieve symptoms associated with low-intestinal obstruction caused by colorectal cancer. However, it is crucial to understand that it is not a curative treatment for the underlying cancer. It is typically used as a supportive measure to improve patient comfort and manage acute symptoms. The long-term management of colorectal cancer requires a comprehensive approach involving oncologists, surgeons, and other specialists to develop an individualized treatment plan tailored to the specific needs of the patient.

**4,** **If there is a recurrence of rectal cancer after surgery, what methods are available to relieve pain?**

(1) One method to relieve pain in cases of recurrent rectal cancer after surgery is through the use of pain medications. Doctors may prescribe opioids or other analgesics to help manage the pain. These medications work by blocking pain signals and can provide significant relief for patients experiencing discomfort.

(2) Another approach to relieving pain in recurrent rectal cancer is through the use of radiation therapy. Radiation therapy involves targeting the cancerous cells with high-energy beams to destroy them or slow down their growth. This treatment can help alleviate pain by shrinking the tumor and reducing pressure on surrounding tissues.

(3) Palliative care is another essential method to manage pain in recurrent rectal cancer. Palliative care focuses on improving the quality of life for patients facing serious illnesses. Pain specialists and palliative care teams work together to develop personalized plans that may include medications, physical therapy, relaxation techniques, and psychological support. These approaches can effectively address pain and provide comfort to patients.

(4) In some cases, interventional procedures may be used to relieve pain caused by recurrent rectal cancer. These procedures include nerve blocks, where medication is injected near specific nerves to block pain signals, or neurolytic blocks, which involve using chemicals to destroy nerves responsible for transmitting pain signals. These interventions can provide localized pain relief and improve the overall well-being of the patient.

Rep2:

(1) One method to relieve pain in cases of recurrent rectal cancer after surgery is through medication. Pain medications such as opioids, nonsteroidal anti-inflammatory drugs (NSAIDs), and adjuvant drugs like antidepressants or anticonvulsants may be prescribed to manage pain. Opioids can help control severe pain, while NSAIDs can reduce inflammation and provide some pain relief. Adjuvant drugs may be used to target specific pain mechanisms, such as neuropathic pain. The choice of medication depends on the individual's pain severity, underlying conditions, and potential side effects.

(2) Another approach to alleviate pain in recurrent rectal cancer is palliative radiation therapy. Radiation therapy aims to shrink tumors and relieve symptoms by delivering high-energy beams to the affected area. It can help reduce pain, control bleeding, and improve quality of life. Palliative radiation therapy is often used in cases where surgery is not feasible or when the cancer has spread to other areas. The radiation dosage and schedule are tailored to the individual's needs, and it may be combined with other pain management techniques for optimal results.

(3) Interventional pain management techniques can also be employed to provide relief from pain associated with recurrent rectal cancer. These procedures target the nerves responsible for transmitting pain signals and can be performed by interventional radiologists or pain specialists. Common interventional techniques include nerve blocks, neurolytic procedures, and implantation of devices like spinal cord stimulators or intrathecal drug delivery systems. These interventions aim to disrupt pain signals, block nerve transmission, or release pain-relieving medications directly into the spinal fluid.

(4) Complementary and alternative therapies may also be considered as adjuncts to conventional pain management strategies. Techniques such as acupuncture, massage therapy, relaxation exercises, and mindfulness meditation have been found to help some individuals manage pain and improve their overall well-being. These therapies can be used alongside medical treatments to enhance pain relief and promote a sense of comfort.

**5, What are the interventional treatment methods for liver metastasis of colorectal cancer? Under what circumstances is interventional treatment chosen?**

(1) One interventional treatment method for liver metastasis of colorectal cancer is hepatic artery embolization or chemoembolization. This procedure involves the injection of embolic agents and chemotherapeutic drugs directly into the hepatic artery, which supplies blood to the liver tumors. The embolic agents block the blood supply to the tumors, while the chemotherapeutic drugs target the cancer cells, resulting in tumor shrinkage or stabilization. This approach is commonly used when the liver metastases are confined to a limited number of tumors and the patient is not a candidate for surgical resection.

(2) Another interventional treatment method for liver metastasis of colorectal cancer is radiofrequency ablation (RFA). RFA utilizes thermal energy to destroy the tumor cells. During the procedure, a needle-like probe is inserted into the liver tumors under image guidance, and high-frequency electrical currents are applied to heat and destroy the cancerous tissue. RFA is often considered for patients with small liver metastases who are not eligible for surgery or when surgery is not feasible due to the location or number of tumors.

(3) Transarterial chemoembolization (TACE) is another interventional treatment method for liver metastasis of colorectal cancer. TACE combines the delivery of chemotherapy drugs and the blocking of blood supply to the tumors. It involves the injection of chemotherapeutic agents directly into the hepatic artery, followed by the injection of embolic materials to block the blood vessels feeding the tumors. This technique allows for a higher concentration of chemotherapy drugs to be delivered directly to the tumor site, while the embolization cuts off the blood flow, further enhancing the treatment effect. TACE is typically considered for patients with liver metastases who are not surgical candidates or when systemic chemotherapy alone is not providing sufficient tumor control.

(4) Selective internal radiation therapy (SIRT) is an interventional treatment method used for liver metastasis of colorectal cancer. SIRT involves the injection of tiny radioactive microspheres directly into the blood vessels that supply the liver tumors. These microspheres deliver radiation directly to the tumor site, targeting the cancer cells while minimizing damage to healthy liver tissue. SIRT is often utilized when surgery and other treatment options are not feasible or have not been successful in controlling the liver metastases. It can be used as a standalone treatment or in combination with other therapies to improve tumor response.

Rep2:

(1) One interventional treatment method for liver metastasis of colorectal cancer is radiofrequency ablation (RFA). RFA involves the insertion of a needle-like electrode into the tumor, guided by imaging techniques such as ultrasound or CT scan. High-frequency electrical currents are then passed through the electrode, creating heat that destroys the tumor cells. RFA is often considered for small liver metastases that are confined to a limited area and can be easily accessed.

(2) Another interventional treatment method is transarterial chemoembolization (TACE). TACE involves the delivery of chemotherapy drugs directly into the blood vessels that supply the tumor. This is done through a catheter inserted into the artery that feeds the liver. Along with the chemotherapy drugs, embolic agents are also administered to block the blood supply to the tumor, thereby depriving it of nutrients and oxygen. TACE is commonly used when the liver metastases are larger or when surgical resection is not feasible.

(3) Selective internal radiation therapy (SIRT), also known as radioembolization, is another interventional treatment option. It involves the injection of tiny radioactive beads, known as microspheres, into the blood vessels that supply the liver tumors. These microspheres emit radiation locally, targeting the tumor and sparing the healthy liver tissue. SIRT is often considered for patients with liver metastases who are not suitable candidates for surgery or have failed other treatments.

(4) In certain circumstances, interventional treatment methods may be chosen when surgical resection is not feasible due to factors such as the size, location, or number of liver metastases. They can be used as a primary treatment option or as a bridge to surgery, aiming to downsize or stabilize the tumors before a curative surgical procedure. Interventional treatments may also be considered when the patient's overall health condition does not permit major surgery, or when there is a desire to minimize invasiveness and preserve liver function.

**6, What is "cold weapon" in the treatment of liver metastasis of colorectal cancer?**

(1) Cryoablation: Cryoablation is a minimally invasive treatment technique used for liver metastasis of colorectal cancer. It involves the use of extreme cold temperatures to destroy cancer cells. During the procedure, a probe is inserted into the liver and the tumor is frozen using liquid nitrogen or argon gas. The freezing temperatures cause the tumor cells to die, and the frozen tissue is eventually absorbed by the body. Cryoablation is typically used for small tumors and can be performed alongside other treatments like surgery or chemotherapy.

(2) Radiofrequency ablation (RFA): Radiofrequency ablation is another technique used in the treatment of liver metastasis of colorectal cancer. It utilizes high-frequency electrical currents to heat and destroy cancer cells. During the procedure, a special needle-like probe is inserted into the liver, and radiofrequency energy is transmitted through the probe to heat the tumor. The heat generated by RFA destroys the tumor cells, leading to their elimination. RFA is often used for small liver tumors and can be performed as a standalone treatment or in combination with other therapies.

(3) Microwave ablation: Microwave ablation is a similar approach to radiofrequency ablation, but it uses microwaves to heat and destroy cancer cells. Like RFA, it involves inserting a probe into the liver tumor and delivering microwave energy to heat the tissue. The heat generated by the microwaves causes cellular destruction, resulting in the eradication of the tumor. Microwave ablation is effective for treating liver metastasis of colorectal cancer, particularly for tumors that are difficult to access surgically or for patients who are not suitable candidates for surgery.

(4) Irreversible electroporation (IRE): Irreversible electroporation is a newer technique used in the treatment of liver metastasis of colorectal cancer. It involves applying short but powerful electrical pulses to the tumor, creating tiny pores in the cell membranes. These pores disrupt the cell's internal balance, leading to cell death. IRE is a precise and targeted treatment method that specifically destroys cancer cells while preserving surrounding healthy tissues. It can be used for liver tumors that are adjacent to critical structures or located in challenging anatomical locations. IRE may be performed as a standalone treatment or combined with other therapies for optimal results.

Rep2:

(1) Cold weapon in the treatment of liver metastasis of colorectal cancer generally refers to cryoablation. Cryoablation is a minimally invasive procedure that uses extreme cold temperatures to destroy cancer cells in the liver. During the procedure, a thin probe is inserted into the tumor, and liquid nitrogen or argon gas is circulated through the probe, creating an extremely cold environment. The freezing temperatures cause the cancer cells to undergo ice crystal formation and subsequent cell death. Cryoablation offers an alternative treatment option for patients who are not suitable candidates for surgery or those with small liver metastases that are difficult to remove surgically.

(2) In the context of liver metastasis of colorectal cancer, cold weapon can also refer to radiofrequency ablation (RFA). RFA uses high-frequency electrical currents to heat and destroy cancer cells. During the procedure, a needle-like electrode is inserted into the tumor, and radiofrequency energy is delivered to generate heat. The heat causes coagulative necrosis, leading to the destruction of the tumor. RFA is an effective treatment for small liver metastases and can be performed using minimally invasive techniques. It is often used as a complementary treatment alongside surgery or in cases where surgery is not feasible.

(3) Another interpretation of cold weapon in the treatment of liver metastasis of colorectal cancer is irreversible electroporation (IRE). IRE is a technique that uses electrical pulses to create nanopores in the cell membranes of cancer cells, leading to their death. The procedure involves inserting thin needles or electrodes into the tumor and applying short bursts of high-voltage electric pulses. These pulses disrupt the integrity of the cell membranes, causing the cancer cells to undergo apoptosis. IRE has shown promising results in treating liver metastases, particularly in cases where tumors are located near critical structures or in patients who cannot undergo surgery.

(4) It's worth mentioning that while the term "cold weapon" may not be commonly used in medical literature, the three techniques described (cryoablation, radiofrequency ablation, and irreversible electroporation) are considered ablative treatments for liver metastasis of colorectal cancer. These techniques aim to destroy cancer cells in the liver using different mechanisms, such as extreme cold temperatures, heat, or electrical pulses. The choice of technique depends on various factors, including tumor characteristics, location, and patient-specific factors. Treatment decisions are typically made in consultation with a multidisciplinary team of healthcare professionals experienced in managing liver metastasis.

**7, What should be done if there is a blockage after previous surgery for rectal cancer?**

(1) If there is a blockage after a previous surgery for rectal cancer, it is important to seek medical attention promptly. The appropriate course of action will depend on the specific circumstances, severity of the blockage, and individual patient factors. Generally, the following steps may be considered:

- Medical evaluation: The patient will undergo a thorough medical evaluation to determine the cause and extent of the blockage. This may involve physical examination, imaging tests such as CT scans or MRI, and possibly endoscopic procedures.

- Non-surgical interventions: In some cases, non-surgical interventions may be attempted to relieve the blockage. These can include bowel rest, dietary modifications, medications to soften the stool, and the use of enemas or suppositories. These interventions aim to relieve the obstruction and restore normal bowel function.

- Surgical options: If non-surgical interventions are not effective or if the blockage is severe, surgical intervention may be necessary. The specific surgical procedure will depend on the location and cause of the blockage. Surgical options can range from minimally invasive procedures to remove the blockage or bypass it, to more extensive surgeries such as colostomy or colostomy reversal.

(2) In cases where a blockage occurs after a previous surgery for rectal cancer, additional treatment options may be considered. These may include:

- Stenting: Placement of a stent can be considered as a temporary measure to relieve the blockage and allow the passage of stool. A stent is a small mesh tube that is inserted into the blocked area to open it up and restore bowel function.

- Radiation therapy: If the blockage is caused by recurrent cancer, radiation therapy may be used to shrink the tumor and relieve the obstruction. This can be done in combination with other treatments such as chemotherapy or surgery.

- Chemotherapy: If the blockage is due to recurrent cancer or metastasis, chemotherapy drugs may be prescribed to help shrink the tumor and alleviate the obstruction. Chemotherapy can be administered before or after surgery, depending on the individual case.

(3) In situations where a blockage occurs following a previous surgery for rectal cancer, careful management is crucial. Here are some general considerations:

- Pain management: Adequate pain management is essential to ensure the patient's comfort. Medications may be prescribed to alleviate pain and provide relief.

- Nutritional support: Depending on the severity and duration of the blockage, the patient may require nutritional support. This can involve a combination of oral nutritional supplements, enteral feeding (tube feeding), or parenteral nutrition (intravenous feeding).

- Close monitoring: Regular monitoring of the patient's condition is necessary to evaluate the effectiveness of the chosen treatment approach. This may involve follow-up appointments, imaging tests, and blood work to assess the progress and adjust the treatment plan as needed.

(4) In cases of blockage after a previous surgery for rectal cancer, the involvement of a multidisciplinary team is often essential. This team may consist of colorectal surgeons, medical oncologists, radiation oncologists, gastroenterologists, and specialized nurses. Collaborative decision-making and individualized treatment plans are key to effectively manage the blockage and optimize the patient's outcome.

Rep2:

(1) If there is a blockage after a previous surgery for rectal cancer, the first step is to seek immediate medical attention. A blockage can occur due to various reasons such as scar tissue formation, tumor recurrence, or narrowing of the intestines. The specific management approach will depend on the individual case, but generally, the following steps may be taken:

- Diagnostic imaging: The healthcare provider will order imaging tests such as CT scans or X-rays to assess the location and cause of the blockage. This helps determine the appropriate treatment approach.

- Non-surgical interventions: In some cases, non-surgical measures may be attempted to relieve the blockage. These can include the insertion of a stent to widen the narrowed area or the use of medications to reduce inflammation and promote bowel movements.

- Surgical intervention: If the blockage is severe or non-surgical methods are unsuccessful, surgery may be necessary. The specific surgical procedure will depend on the extent and cause of the blockage. It could involve removing the blockage, bypassing the affected area, or rerouting the intestine to restore normal bowel function.

(2) Another possible course of action when encountering a blockage after previous rectal cancer surgery is to consult with a specialist. Specialists in colorectal surgery or surgical oncology have expertise in managing post-surgical complications and can provide valuable insights and treatment options.

- Evaluation of previous surgery: The specialist will review the patient's medical history, including details of the previous rectal cancer surgery. This evaluation helps them understand the surgical techniques used, potential complications, and any specific factors that may contribute to the blockage.

- In-depth examination: The specialist will perform a thorough physical examination, including a rectal exam, to assess the blockage and determine its severity. They may also order additional tests such as colonoscopy or sigmoidoscopy to visualize the area directly.

- Collaborative treatment plan: Based on the evaluation and examination, the specialist will work with the patient to develop a personalized treatment plan. This may involve a combination of non-surgical interventions, medication, dietary changes, and possibly additional surgery if required.

(3) Seeking a second opinion from another qualified medical professional is another recommended step when dealing with a blockage after rectal cancer surgery. Second opinions can provide alternative perspectives, offer fresh insights, and ensure that all available treatment options have been considered.

- Gathering medical records: The patient should gather all relevant medical records, including surgical reports, imaging results, and pathology reports, to provide the second opinion specialist with a comprehensive understanding of the case.

- Consultation with another expert: The patient can schedule an appointment with a different specialist or surgical team to discuss the blockage and seek their expert opinion. The specialist will review the medical records, perform a physical examination, and provide their recommendations based on their expertise and experience.

- Weighing treatment options: After receiving the second opinion, the patient can compare the recommendations from both specialists and weigh the pros and cons of each option. This process can help the patient make an informed decision about the next steps in managing the blockage.

**8,** **Can colorectal cancer also cause jaundice?**

(1) Colorectal cancer typically does not cause jaundice. Jaundice is a condition characterized by the yellowing of the skin and eyes, usually caused by an excess of bilirubin in the bloodstream. Bilirubin is a yellow pigment produced during the breakdown of red blood cells, and it is normally processed by the liver and excreted through bile.

(2) Jaundice is more commonly associated with liver diseases, such as hepatitis or cirrhosis, where there is a disruption in the liver's ability to process bilirubin. However, in rare cases, colorectal cancer can cause jaundice if the tumor blocks the bile ducts. When the bile ducts become obstructed, bile cannot flow properly from the liver to the intestines, leading to an accumulation of bilirubin in the bloodstream and subsequent jaundice.

(3) It's important to note that jaundice is not a common symptom of colorectal cancer. The most common signs and symptoms of colorectal cancer include changes in bowel habits (such as diarrhea or constipation), rectal bleeding, blood in the stool, abdominal pain or cramping, unexplained weight loss, and fatigue. If you're experiencing jaundice or any concerning symptoms, it's best to consult a healthcare professional for a proper evaluation and diagnosis.

Rep2:

(1) Colorectal cancer typically does not cause jaundice directly. Jaundice is a condition characterized by yellowing of the skin and eyes, which occurs when there is a buildup of bilirubin in the body. Bilirubin is a yellow pigment produced during the breakdown of red blood cells.

(2) However, colorectal cancer can indirectly lead to jaundice if it obstructs the bile ducts. The bile ducts are responsible for carrying bile, a fluid produced by the liver that helps in the digestion of fats, from the liver to the small intestine. If a colorectal tumor grows near or within the bile ducts, it can block the flow of bile, leading to a condition called obstructive jaundice.

(3) Obstructive jaundice causes bile to accumulate in the liver, resulting in an increased level of bilirubin in the bloodstream. This buildup of bilirubin causes the characteristic yellowing of the skin and eyes. Other symptoms of obstructive jaundice may include dark urine, pale stools, itching, abdominal pain, and unexplained weight loss.

(4) If jaundice is present in a person with colorectal cancer, it may indicate that the cancer has advanced and spread to nearby structures, such as the liver or bile ducts. It is important to consult a healthcare professional if you experience symptoms such as jaundice or any other concerning signs. They can evaluate your condition, conduct appropriate tests, and provide an accurate diagnosis.

**The Stoma Cares**

**1, What is an intestinal stoma? What are the main functions and types of intestinal stomas?**

(1) An intestinal stoma is a surgical opening created in the abdominal wall that allows for the diversion of intestinal contents to the outside of the body. It is typically formed when a portion of the intestine is brought out onto the surface of the abdomen. The stoma appears as a small, pinkish, moist opening, which can be temporary or permanent, depending on the individual's condition.

(2) The main function of an intestinal stoma is to provide a new route for waste elimination when the normal pathway is either diseased, damaged, or removed. It allows for the passage of stool or fecal matter to be collected in an external pouching system, which is worn over the stoma and attached securely to the abdomen. By bypassing a segment of the intestine, an intestinal stoma helps to manage conditions such as bowel obstructions, inflammatory bowel disease, colorectal cancer, or other disorders affecting the intestines.

(3) There are different types of intestinal stomas, each created based on specific medical requirements. The two primary types are colostomy and ileostomy.

- Colostomy: In a colostomy, the stoma is made from a portion of the colon (large intestine). The location of the colostomy can vary, and it can be temporary or permanent, depending on the reason for the surgery. Temporary colostomies are usually created to allow a damaged or diseased portion of the colon to heal, while permanent colostomies are typically performed when a significant portion of the colon needs to be removed.

- Ileostomy: An ileostomy involves creating a stoma using the end of the small intestine (ileum). This type of stoma is generally located on the right side of the abdomen. Unlike a colostomy, an ileostomy bypasses the entire colon, including the rectum. Ileostomies can also be temporary or permanent, depending on the underlying condition.

Other less common types of intestinal stomas include urostomy (for urinary diversion) and jejunostomy (for direct access to the jejunum).

Rep2:

(1) An intestinal stoma is a surgically created opening in the abdominal wall that allows the passage of waste material from the intestines to exit the body. It is typically formed when a portion of the intestine is brought out through the abdominal wall and sutured in place. The stoma serves as an alternative route for the elimination of feces or intestinal contents when the normal route is blocked or not functional.

(2) The main function of an intestinal stoma is to divert the flow of feces or intestinal contents away from the usual route of elimination. This can be necessary in cases where the lower part of the digestive system is damaged, diseased, or removed. By creating an opening on the abdomen, the stoma allows waste material to be eliminated into a collection bag or pouch attached to the skin, thereby bypassing the damaged or non-functional section of the intestine.

(3) There are different types of intestinal stomas, depending on the section of the intestine involved and the purpose of the stoma. Some common types include:

- Colostomy: This stoma is created using the colon (large intestine). It may be formed in the ascending colon, transverse colon, or descending colon. The location of the colostomy determines the consistency of the stool.

- Ileostomy: This stoma is created using the ileum (the final part of the small intestine). It is typically formed when the colon and rectum are removed or bypassed. The stool from an ileostomy is liquid or semi-liquid.

- Loop stoma: In this type of stoma, a loop of the intestine is brought out onto the abdomen's surface. The loop is then opened and secured to the skin. A loop stoma can be temporary or permanent, depending on the medical condition and treatment plan.

- End stoma: This stoma is created by bringing the end of the intestine to the abdominal wall. It is typically a permanent stoma and can be either a colostomy or an ileostomy.

(4) There are additional variations and modifications of stomas, such as double-barrel colostomy, which involves dividing the colon and creating two separate stomas, and continent ileostomy, which involves creating a pouch from the small intestine that can be catheterized to remove waste. The type of stoma selected depends on the specific medical condition, the extent of surgery required, and the individual patient's needs. It is important for individuals with intestinal stomas to receive appropriate care and support to manage their stoma and maintain their quality of life.

**2,** **After having an intestinal stoma, can it be reversed?**

(1) Yes, in some cases, an intestinal stoma can be reversed. The reversal procedure is known as stoma closure or stoma reversal surgery. It involves reconnecting the previously diverted sections of the intestine to restore the normal flow of waste through the digestive system. The decision to reverse an intestinal stoma depends on various factors such as the underlying medical condition, the reason for the stoma creation, and the overall health of the individual. The reversal procedure is typically performed by a qualified surgeon and may require a hospital stay and a period of recovery.

(2) The possibility of reversing an intestinal stoma depends on several factors, including the type of stoma and the individual's specific circumstances. Temporary stomas, which are created for a specific period to allow the intestines to heal or bypass a problem area, are more likely to be reversible. Permanent stomas, on the other hand, are intended for long-term use and are less likely to be reversed. However, even some permanent stomas can be reversed in certain cases, especially if the individual's condition improves or if there are changes in their medical treatment options.

(3) Reversing an intestinal stoma is a surgical procedure that involves reconnecting the ends of the intestine that were previously separated during stoma creation. The surgery aims to restore the natural path of digestion and eliminate the need for an artificial opening on the abdomen. The decision to reverse a stoma is made after careful evaluation by medical professionals who consider the individual's overall health, the reason for stoma creation, and the potential benefits and risks of the reversal procedure. It's important to consult with a healthcare team, including a surgeon specializing in stoma reversal, to determine the feasibility and appropriateness of stoma reversal in each specific case.

(4) The reversal of an intestinal stoma is a surgical option that may be considered based on individual circumstances. However, it is essential to note that not all stomas can be reversed. Factors such as the type of stoma, the underlying medical condition, and the overall health of the individual play a significant role in determining the feasibility of stoma reversal. Consulting with a healthcare professional who specializes in stoma care and surgery is crucial for assessing the potential for reversal and making an informed decision. They can provide personalized guidance and recommendations based on the specific situation.

Rep2:

(1) Yes, it is generally possible to reverse an intestinal stoma. The reversal procedure is known as stoma closure or stoma reversal surgery. This surgery involves reconnecting the intestine, allowing waste to pass through the normal route and eliminating the need for the stoma.

(2) The decision to reverse an intestinal stoma depends on various factors, including the reason for the stoma creation, the individual's overall health, and the condition of the intestine. If the stoma was created due to a temporary condition or surgery, such as diverticulitis or bowel resection, and the underlying issue has been resolved, reversal may be a viable option.

(3) Stoma reversal surgery is typically performed by a skilled surgeon. The procedure involves making an incision near the stoma, disconnecting the intestine from the stoma, and reattaching the ends of the intestine to restore the continuity of the digestive tract. The specific technique used may vary depending on the individual's unique circumstances and the location of the stoma.

(4) After stoma closure surgery, it may take some time for the digestive system to fully recover and resume normal functioning. The individual may experience temporary changes in bowel habits or abdominal discomfort during the recovery period. Close monitoring by healthcare professionals is necessary to ensure proper healing and to address any complications that may arise.

(5) It is important to note that not all intestinal stomas can be reversed. In some cases, the stoma may be created as a permanent measure due to certain medical conditions or complications. The decision to reverse or keep a stoma is typically made after a thorough evaluation by healthcare professionals, considering the individual's specific circumstances and medical needs.

**3, How to change an intestinal stoma bag?**

(1) Generally, changing an intestinal stoma bag involves the following steps:

a. Gather the necessary supplies: You will need a new stoma bag, adhesive remover wipes or spray, clean towels or washcloths, a disposable bag for the old stoma bag, and any additional items recommended by your healthcare professional.

b. Prepare the area: Place a towel or washcloth underneath the stoma to catch any output during the bag change. Make sure the surrounding skin is clean and dry.

c. Remove the old stoma bag: Carefully peel off the adhesive barrier while supporting the skin around the stoma. Use an adhesive remover wipe or spray, if needed, to help loosen the adhesive. Gently lift the bag away from the skin, taking care not to apply too much force to the stoma.

d. Clean the stoma and surrounding skin: Use warm water and a mild, non-irritating cleanser or wipes to clean the stoma and the surrounding skin. Avoid using soap or products that contain oil or lotion, as they can interfere with the bag's adhesion.

e. Dry the area: Gently pat the stoma and surrounding skin dry with a clean towel or cloth. Ensure the skin is completely dry before applying a new bag.

f. Apply the new stoma bag: Position the adhesive barrier around the stoma, ensuring it fits securely and leaves no gaps. Smooth out any wrinkles or creases to create a proper seal. Attach the pouch to the adhesive barrier and ensure it is securely in place.

g. Dispose of the old bag: Seal the old stoma bag in a disposable bag and discard it in an appropriate manner.

(2) Generally, here's another way to change an intestinal stoma bag:

a. Begin by gathering the necessary supplies: You will need a new stoma bag, adhesive remover wipes or spray, clean water, mild soap or cleanser, a towel or washcloth, and a disposal bag.

b. Prepare the area: Place a towel or washcloth under the stoma to protect the surrounding surface. Ensure the area is clean and dry before starting the bag change.

c. Remove the old bag: Carefully peel off the adhesive barrier, taking care not to put excessive pressure on the stoma. If needed, use an adhesive remover wipe or spray to assist with the removal. Slowly lift the bag away from the skin, ensuring you don't cause any discomfort to the stoma.

d. Clean the stoma and surrounding skin: Use clean water and a mild soap or cleanser to gently cleanse the stoma and the skin around it. Avoid using products that contain alcohol, oil, or lotions, as they may affect the adhesion of the new bag.

e. Dry the area: Pat the stoma and surrounding skin dry with a towel or washcloth. Make sure the area is completely dry before applying the new bag.

f. Apply the new stoma bag: Position the adhesive barrier around the stoma, ensuring a proper fit and avoiding any wrinkles or creases. Press the adhesive firmly onto the skin to create a secure seal. Attach the new pouch to the adhesive barrier, making sure it is securely connected.

g. Dispose of the old bag: Place the old stoma bag into a disposable bag and dispose of it appropriately, following the guidelines provided by your healthcare professional.

(3) Generally, here's another method for changing an intestinal stoma bag:

a. Gather the necessary supplies: Prepare a new stoma bag, adhesive remover wipes or spray, warm water, mild soap, a towel or washcloth, and a disposal bag.

b. Prepare the area: Lay a towel or washcloth beneath the stoma to protect the surrounding surface. Ensure the skin is clean and dry before

Rep2:

(1) Start by gathering all the necessary supplies, such as a new stoma bag, adhesive remover, wipes, clean towels, and a disposal bag. Wash your hands thoroughly with soap and water before beginning the process.

(2) Carefully remove the existing stoma bag by peeling off the adhesive, starting from one corner and gently pulling it away from the skin. If the adhesive is stubborn, you can use an adhesive remover to help loosen it.

(3) Cleanse the skin around the stoma using mild soap and water or specialized stoma cleansers. Gently pat the area dry with a clean towel, being careful not to rub or irritate the skin.

(4) Take the new stoma bag and ensure it is the correct size and type for your stoma. Remove the backing from the adhesive portion of the bag, but be careful not to touch the adhesive with your fingers.

(5) Position the bag over the stoma, aligning it properly to ensure a secure fit. Gently press the adhesive around the stoma, starting from the center and moving outward, making sure there are no creases or gaps.

(6) Once the bag is securely attached, smooth out any wrinkles or bubbles to create a good seal. You can use your hand or the warmth of your palm to help the adhesive adhere properly.

(7) Dispose of the used stoma bag in a disposal bag and seal it properly. Wash your hands again with soap and water to ensure cleanliness.

**4,** **What are the products for stoma care?**

(1) Stoma care products are designed to support individuals who have undergone ostomy surgery. These products are specifically created to help manage and maintain a stoma, which is a surgically created opening on the abdomen that allows waste to exit the body. Stoma care products include items like stoma bags (also known as ostomy pouches), skin barriers, adhesive rings, and accessories like belts and adhesive removers. These products aim to provide comfort, protection, and convenience for individuals living with a stoma.

(2) One essential product in stoma care is the stoma bag or ostomy pouch. This bag is attached to the stoma and collects waste material, allowing individuals to lead a normal life without the need for constant bathroom visits. Stoma bags come in different types, such as one-piece and two-piece systems. One-piece systems have the bag and adhesive barrier combined, while two-piece systems have a separate adhesive wafer that attaches to the skin, and the bag can be easily attached and detached as needed.

(3) Skin barriers, also known as flanges or baseplates, are another crucial component of stoma care. These barriers are designed to protect the skin around the stoma from contact with waste material and to ensure a secure attachment of the stoma bag. They are typically made of skin-friendly materials such as hydrocolloid or silicone, which provide a gentle and protective barrier between the stoma and the skin.

(4) Adhesive rings or seals are additional products used in stoma care. These rings are placed around the stoma to create a seal between the stoma and the stoma bag, preventing leakage and ensuring a secure fit. They help maintain the integrity of the system, providing added protection and preventing skin irritation or damage.

(5) Stoma care accessories are also available to enhance convenience and comfort. These may include stoma belts that help keep the stoma bag in place and provide additional support, especially during physical activities. Adhesive removers are used to gently remove the adhesive from the skin when changing the stoma bag, ensuring a clean and irritation-free removal process.

Rep2:

(1) Stoma care products are specifically designed to assist individuals who have undergone stoma surgery. A stoma is an opening created in the body to divert the flow of bodily waste, such as urine or feces, into a bag or pouch on the outside of the body. Stoma care products play a crucial role in ensuring the comfort, hygiene, and overall well-being of individuals with stomas. They include various types of pouching systems, skin barriers, adhesive products, cleansers, and accessories.

(2) Pouching systems are an essential component of stoma care. They consist of a bag or pouch that securely collects waste material from the stoma. These pouches come in different sizes, shapes, and types, including one-piece or two-piece systems. One-piece systems have the pouch and adhesive barrier combined, while two-piece systems have a separate adhesive wafer and pouch. Pouches can also be drainable or closed-end, depending on the individual's needs.

(3) Skin barriers, also known as flanges or baseplates, are protective barriers that adhere to the skin surrounding the stoma. They create a seal that helps prevent leaks and irritation. Skin barriers are available in various sizes and materials to accommodate different stoma sizes and skin sensitivities. Some barriers have convexity options to help manage uneven or retracted stomas. They provide a secure foundation for attaching the pouching system and help maintain skin integrity.

(4) Adhesive products are used to ensure a secure and long-lasting attachment between the pouching system and the skin barrier. These can include adhesive tapes, strips, or rings. They help create a tight seal and prevent leakage, which is crucial for the comfort and confidence of individuals with stomas. Adhesive products should be gentle on the skin while providing a strong hold to keep the pouching system in place.

(5) Cleansers and skin-preparation products are used to clean and protect the skin around the stoma. These can include gentle cleansers, wipes, or sprays that help remove adhesive residue, oils, and other debris. Skin-preparation products, such as barrier films or powders, create a protective layer on the skin, preventing irritation and promoting better adhesion of the skin barrier.

(6) Accessories for stoma care are additional products that can enhance the convenience and effectiveness of managing a stoma. These can include stoma belts or bands that provide added support and security for the pouching system, deodorizers to minimize odor, irrigation systems for colostomies, or specialized products for specific stoma types or conditions. These accessories are designed to address individual needs and preferences, providing personalized stoma care solutions.

**5,** **What are the precautions for caring for an intestinal stoma?**

(1) One of the precautions for caring for an intestinal stoma is maintaining proper hygiene. It is essential to clean the stoma and the surrounding skin regularly to prevent infection and skin irritation. Use mild soap and water or specialized stoma cleansers recommended by healthcare professionals. Avoid using harsh chemicals, alcohol-based products, or adhesive removers that can damage the skin.

(2) Another precaution is ensuring a proper fit of the stoma appliance. The stoma pouching system should fit securely around the stoma without causing any discomfort or leaks. Regularly check the size and shape of the stoma to ensure the correct size of the appliance. Empty the pouch when it is about one-third full to prevent leakage and skin irritation.

(3) Proper diet and hydration are crucial for stoma care. It is important to maintain a well-balanced diet that includes an adequate amount of fiber, unless advised otherwise by a healthcare professional. Drinking plenty of fluids helps prevent dehydration, which can lead to thicker output and potential blockage of the stoma.

(4) Carefully monitor the stoma and surrounding skin for any signs of complications. Look for changes in color, size, or shape of the stoma, as well as skin redness, irritation, or signs of infection such as pus or foul odor. Report any unusual symptoms to a healthcare professional promptly.

(5) Protect the stoma during physical activities or when wearing tight clothing. Use a stoma guard or belt if necessary to prevent any pressure or impact on the stoma. Be cautious while participating in activities that involve contact sports or excessive abdominal pressure.

(6) Seek professional advice and support. Regularly consult with a healthcare professional who specializes in stoma care. They can provide guidance, address concerns, and teach proper stoma care techniques. Additionally, joining support groups or online communities can offer emotional support and a platform to share experiences with others who have stomas.

Rep2:

1. Proper Stoma Care: It is crucial to maintain good stoma care to prevent complications and maintain the health of the surrounding skin. This involves regular cleaning of the stoma and the skin around it using mild soap and water or a stoma cleanser recommended by a healthcare professional. Gently pat the area dry after cleaning to avoid irritation.

2. Skin Protection: Protecting the skin around the stoma is essential. Use a skin barrier or protective paste to create a barrier between the stoma output and the skin. This helps to prevent irritation, leakage, and skin breakdown. Ensure that the skin barrier fits well and is changed regularly or as advised by a healthcare professional.

3. Proper Bag Application: Ensure that the ostomy bag or pouch is applied correctly to the stoma. The bag should fit securely and create a proper seal to prevent leakage. Empty the bag when it is about one-third to one-half full to prevent it from becoming too heavy and pulling on the stoma.

4. Healthy Diet and Hydration: Maintain a well-balanced diet and stay hydrated to support overall intestinal health. Consult a healthcare professional for specific dietary recommendations. Avoid foods that may cause blockages or excessive gas production, as they can lead to complications with the stoma.

5. Regular Stoma Check-ups: Regularly monitor the stoma and the skin around it for any changes, such as redness, swelling, discharge, or unusual odors. Report any concerns or abnormalities to a healthcare professional promptly.

6. Physical Activity: Engage in regular physical activity as directed by a healthcare professional. Exercise helps promote healthy bowel function and can improve overall well-being. However, it's important to avoid activities that may put excessive strain on the stoma, such as heavy lifting or contact sports.

7. Emotional Support: Living with an intestinal stoma can have emotional and psychological effects. Seek support from healthcare professionals, support groups, or counselors specializing in ostomy care. Emotional well-being is essential for overall stoma care and adaptation to a new lifestyle.

**6,** **After getting a stoma, how should one eat?**

(1) Following the placement of a stoma, it is important to adjust your eating habits to accommodate the changes in your digestive system. Here are some general guidelines to consider:

- Chew your food thoroughly: Taking the time to chew your food thoroughly aids in digestion and can help prevent blockages or complications. It is recommended to chew your food until it reaches a semi-liquid consistency before swallowing.

- Stay hydrated: Drink plenty of fluids throughout the day to maintain hydration. Water is usually the best choice but consult your healthcare provider for any specific fluid requirements or restrictions.

- Monitor your diet: Depending on the type of stoma you have, there may be specific dietary recommendations. For example, if you have a colostomy, you may be advised to consume a high-fiber diet to help regulate bowel movements. On the other hand, if you have an ileostomy, you may need to avoid certain foods that can cause blockages, such as seeds, nuts, and tough fibrous foods. It's important to work closely with a registered dietitian or healthcare provider who specializes in ostomy care to create an individualized eating plan.

(2) Take note of portion sizes: It can be helpful to eat smaller, more frequent meals rather than large meals. This approach can minimize the stress on your digestive system and promote better digestion. Additionally, consider eating slower and allowing yourself ample time to enjoy your meals, as rushing can lead to swallowing air and potential discomfort.

(3) Choose a balanced diet: Aim for a well-rounded diet that includes a variety of nutrient-dense foods. This typically involves consuming a mix of fruits, vegetables, whole grains, lean proteins, and healthy fats. However, everyone's dietary needs and tolerances may vary, so it's crucial to work with a healthcare professional to tailor your diet to your specific situation.

(4) Experiment and observe: Keep track of how different foods affect your stoma and digestion. Certain foods may cause gas, odor, or output changes, while others may be well-tolerated. Maintaining a food diary can be helpful in identifying patterns and making adjustments to your diet accordingly. If you notice any significant changes or concerns, don't hesitate to consult your healthcare provider or an ostomy nurse for further guidance.

Rep2:

(1) After getting a stoma, it is important to adapt your eating habits to ensure proper nutrition and minimize potential complications. Generally, you should follow these guidelines:

- Chew your food thoroughly: Chewing your food well aids in digestion and helps prevent blockages in the stoma. Take your time and eat slowly.

- Stay hydrated: Drink plenty of fluids throughout the day to maintain hydration. This is particularly important as having a stoma may increase your risk of dehydration.

- Follow your healthcare professional's recommendations: Your healthcare provider will provide specific dietary instructions based on your individual needs and the type of stoma you have. It's essential to follow their guidance regarding diet and nutrition.

(2) Additionally, consider the following general recommendations for eating after getting a stoma:

- Incorporate a balanced diet: Aim to include a variety of foods from different food groups, such as fruits, vegetables, whole grains, lean proteins, and healthy fats. This helps ensure you receive essential nutrients.

- Monitor fiber intake: Depending on your stoma type and any associated conditions, your healthcare provider may advise adjusting your fiber intake. In some cases, a low-fiber diet might be recommended to reduce the risk of blockages or obstructions.

- Be mindful of gas-forming foods: Certain foods like beans, lentils, carbonated drinks, and cabbage can lead to excess gas production. Monitor how these foods affect your stoma and adjust your diet accordingly.

(3) Here are a few more general tips for eating with a stoma:

- Plan your meals and snacks: Maintaining a regular eating schedule can help regulate bowel movements and manage your stoma output. Plan your meals and snacks to include a mix of nutrients throughout the day.

- Manage potential odor: Some foods may contribute to stronger odors in the stool. Consider experimenting with different foods to find what works best for you, and discuss any concerns with your healthcare provider.

- Stay vigilant for food blockages: Pay attention to how your stoma reacts to different foods. If you notice any signs of blockages, such as abdominal pain, swelling, or decreased output, seek medical attention promptly.

**7, After getting a stoma, is it possible to participate in physical exercise?**

(1) Yes, it is generally possible to participate in physical exercise after getting a stoma. A stoma is an opening surgically created on the abdomen to divert waste from the digestive or urinary system. While the stoma itself does not restrict physical activity, it is important to consider individual circumstances, such as the type of stoma and any underlying medical conditions, before engaging in exercise. It is advisable to consult with a healthcare professional, such as a stoma nurse or a doctor, to get personalized advice on exercise guidelines and precautions specific to your situation.

(2) Engaging in physical exercise after getting a stoma can have numerous benefits. Exercise promotes overall health and well-being, including improved cardiovascular fitness, increased muscle strength and flexibility, enhanced mood, and stress reduction. It can also help manage weight, boost self-esteem, and improve body image. Regular exercise can contribute to a healthier lifestyle and improve the quality of life for individuals with a stoma, as it can help maintain or improve physical function and mobility.

(3) When participating in physical exercise with a stoma, it is important to take certain precautions. First, it is crucial to ensure that the stoma is well-protected during exercise to prevent any damage or injury. Using a stoma guard or protective belt can help provide support and safeguard the stoma. It may be necessary to choose appropriate clothing that allows for easy access to the stoma and provides comfort during movement.

(4) Additionally, it is important to be mindful of hydration while exercising, especially if you have an ostomy. Staying well-hydrated can help prevent dehydration, which may be more common for individuals with an ileostomy due to increased fluid loss. It is advisable to drink plenty of fluids before, during, and after exercise, and consult with a healthcare professional for specific hydration recommendations.

(5) Depending on the type of stoma and any accompanying medical conditions, certain activities or exercises may need to be modified or avoided altogether. For example, high-impact activities that involve significant abdominal movements or excessive pressure on the stoma area may need to be approached with caution. It is important to listen to your body, start slowly, and gradually increase the intensity and duration of exercise. Your healthcare professional can guide you on suitable exercises and any necessary modifications based on your specific circumstances.

(6) In conclusion, participating in physical exercise after getting a stoma is generally possible and can provide numerous benefits for overall health and well-being. However, it is crucial to consult with a healthcare professional to receive personalized advice, consider any individual limitations or precautions, and ensure that you engage in exercise safely and effectively.

Rep2:

(1) Yes, it is generally possible to participate in physical exercise after getting a stoma. A stoma is a surgical opening created on the abdomen to divert the flow of bodily waste into a bag attached to the skin's surface. While it may require some adjustments and precautions, individuals with a stoma can engage in various forms of physical activity to maintain their overall health and well-being.

(2) When participating in physical exercise with a stoma, it is important to consult with a healthcare professional or a stoma nurse. They can provide guidance specific to your condition and help you develop an exercise plan that suits your abilities and goals. They may recommend exercises that strengthen the core, improve cardiovascular fitness, and enhance overall strength and flexibility.

(3) It is advisable to start slowly and gradually increase the intensity and duration of your exercise routine. Listen to your body and pay attention to any discomfort or signs of irritation around the stoma site. It may be necessary to wear appropriate support garments or use specialized ostomy accessories to ensure comfort and prevent any potential complications during exercise.

(4) Engaging in physical activity with a stoma can offer numerous benefits. It can help improve overall fitness levels, boost energy, enhance mood, and reduce stress. Regular exercise can also help maintain a healthy weight, strengthen muscles, improve digestion, and promote better sleep. By working closely with healthcare professionals, individuals with a stoma can find suitable exercises and activities that accommodate their specific needs and allow them to stay active.

**8, After getting a stoma, is it possible to take a shower?**

(1) Yes, it is generally possible to take a shower after getting a stoma. A stoma is an opening created surgically on the abdomen to divert the flow of bodily waste. It can be a result of various medical conditions such as inflammatory bowel disease, colorectal cancer, or other gastrointestinal issues. While the stoma itself should not be submerged in water, precautions can be taken to ensure a safe and hygienic showering experience.

(2) When taking a shower with a stoma, it is important to protect the stoma from direct contact with water. You can cover the stoma with a waterproof adhesive stoma cover or a specialized shower shield designed for this purpose. These covers help to keep the stoma dry while allowing you to clean the surrounding area. It is essential to follow the instructions provided by your healthcare professional regarding the type and use of stoma covers.

(3) In addition to using stoma covers, it is advisable to avoid using soaps or cleansers directly on the stoma area during the shower. These products can irritate the stoma or cause damage to the surrounding skin. Instead, mild, non-perfumed soaps or cleansers can be used to clean the skin around the stoma. Gently patting the area dry with a soft towel is recommended after showering.

(4) It is also important to ensure that the water temperature is comfortable and not too hot. Extremely hot water can cause excessive sweating, which may lead to skin irritation or breakdown around the stoma. It is best to use lukewarm water during showers.

(5) If you have recently undergone stoma surgery, your healthcare team will provide you with specific instructions on how to care for your stoma, including showering guidelines. It is important to follow their advice and seek clarification if you have any concerns or questions.

Rep2:

(1) Yes, it is generally possible to take a shower after getting a stoma. However, it is important to take certain precautions to protect the stoma and the surrounding area.

(2) When taking a shower with a stoma, it is recommended to cover the stoma with a waterproof ostomy pouch or a stoma guard to prevent water from entering the stoma. This helps to keep the stoma clean and dry during the shower.

(3) Additionally, it is important to ensure that the water temperature is not too hot, as excessive heat can cause sweating and affect the adhesion of the ostomy pouch. Lukewarm water is usually recommended.

(4) It is advisable to avoid using harsh soaps or shower gels directly on the stoma area, as they can cause skin irritation. Instead, use mild, non-fragrant soaps and gently clean around the stoma with a soft cloth or sponge.

(5) After showering, it is essential to dry the stoma and the surrounding skin thoroughly. Excess moisture can lead to skin irritation or breakdown, so patting the area gently with a towel or using a hairdryer on a cool setting can be helpful.

(6) If you have any concerns or questions about showering with a stoma, it is always best to consult with your healthcare provider or a stoma care nurse. They can provide specific guidance and recommendations based on your individual needs and circumstances.

**9, After getting a stoma, is it possible to have sexual activity?**

(1) Yes, it is generally possible to engage in sexual activity after getting a stoma. A stoma is an opening created surgically on the abdomen to divert the flow of bodily waste from the digestive or urinary system. While it may present some physical and emotional challenges, it does not necessarily hinder sexual activity.

(2) Sexual activity after getting a stoma requires open communication and understanding between partners. It is important to discuss any concerns or limitations with your healthcare provider or a specialist nurse who can provide guidance specific to your situation. They can offer advice on positioning, support garments, and potential modifications to ensure comfort during sexual activity.

(3) Using accessories or support devices like ostomy belts or pouch covers can provide additional comfort and confidence during sexual activity with a stoma. Experimenting with different positions to find what works best for you and your partner can also help ensure a pleasurable experience. Some individuals may find it helpful to empty or change the stoma pouch before engaging in sexual activity to reduce any potential discomfort.

(4) It is essential to consider individual circumstances and any potential limitations that may arise due to the presence of a stoma. Factors such as the type of stoma, overall health, and personal preferences can vary between individuals. Therefore, it is crucial to consult with a healthcare professional who can provide personalized advice based on your specific needs and concerns. They can address any potential risks, provide guidance on maintaining hygiene, and offer support to ensure a healthy and enjoyable sexual experience after getting a stoma.

Rep2:

(1) Yes, it is possible to engage in sexual activity after getting a stoma. A stoma is an artificial opening created surgically on the abdomen to divert the flow of waste when a person's natural digestive or urinary system is impaired. While the presence of a stoma may require some adjustments and considerations, it does not necessarily prevent individuals from having a fulfilling and enjoyable sex life.

(2) Communication and understanding between partners are crucial. Open and honest communication is key when discussing sexual activity after getting a stoma. Both partners should share their concerns, desires, and any potential limitations or adjustments that may be necessary. This allows for mutual understanding and the opportunity to explore different approaches or positions that accommodate the individual's needs.

(3) Experimentation and finding what works best. Each person's experience with a stoma is unique, and what feels comfortable or pleasurable may vary. It may take some time and experimentation to find positions or techniques that work best for both partners. Exploring different approaches, trying new positions, and using additional support such as pillows or cushions can help find the most comfortable and enjoyable options.

(4) Seeking guidance from healthcare professionals. If individuals have concerns or specific questions about sexual activity after getting a stoma, it is advisable to consult healthcare professionals who specialize in stoma care, such as stoma nurses or doctors. They can provide personalized advice, address concerns, and offer guidance on managing the stoma during sexual activity.

(5) Emotional support and self-confidence. Adjusting to life with a stoma may impact a person's body image and self-esteem. It is essential to address any emotional concerns and seek support from loved ones, support groups, or mental health professionals if needed. Building self-confidence and feeling comfortable with one's body can positively influence sexual experiences and overall well-being.

**10, After getting a stoma, is it possible to work or study?**

(1) Yes, it is generally possible to work or study after getting a stoma. A stoma is an opening created surgically on the abdomen to divert waste from the digestive or urinary system. While living with a stoma requires adjustments and changes in daily routines, it does not necessarily prevent individuals from pursuing their professional or educational goals.

(2) With appropriate care and management, many people with stomas are able to continue their work or academic pursuits. Modern stoma appliances and accessories, such as ostomy bags, are designed to be discreet and secure, allowing individuals to maintain their personal hygiene and confidently engage in various activities, including work or study.

(3) The ability to work or study with a stoma depends on several factors, including the individual's overall health, the type of stoma they have, and the nature of their job or educational program. In most cases, individuals can resume their regular activities after a recovery period and with the necessary support from healthcare professionals, stoma care nurses, and support groups.

(4) It is important for individuals with stomas to communicate with their employers or educational institutions about their specific needs and any accommodations that may be required. Employers and educational institutions are generally required to make reasonable accommodations to ensure equal opportunities for individuals with medical conditions, including those with stomas.

(5) However, it's worth noting that some individuals may require additional time for recovery or may need to make adjustments in their work or study schedules due to medical appointments or the need for extra self-care. It is essential for individuals to prioritize their health and well-being and to seek guidance from healthcare professionals to manage their stoma effectively while pursuing their professional or educational goals.

Rep2:

(1) Yes, it is generally possible to work or study after getting a stoma. A stoma is an opening created surgically on the abdomen to divert the flow of waste from the body. While it may require some adjustments and accommodations, having a stoma does not necessarily prevent someone from pursuing their work or educational goals.

(2) With proper management and support, individuals with a stoma can continue their employment or studies. It is important to communicate with employers or educational institutions about the condition to ensure appropriate accommodations are in place. This may include flexible schedules for medical appointments, access to restroom facilities, and understanding colleagues or classmates who are educated about the stoma's requirements.

(3) Some individuals with a stoma may require temporary time off work or studies during the initial recovery period after surgery. This allows them to adapt to the stoma, receive any necessary training on stoma care, and regain their strength. However, most people can gradually return to their regular activities and may even find that their productivity improves as they adjust to the new normal.

(4) It is essential for individuals with a stoma to manage their condition effectively to minimize any impact on their work or studies. This involves proper stoma care, including changing the appliance regularly, maintaining good hygiene, and managing potential complications such as skin irritation or leaks. Access to support groups, healthcare professionals, and stoma care nurses can provide valuable guidance and assistance in navigating the challenges of living with a stoma while pursuing professional or educational goals.

(5) Remember that every person's experience with a stoma is unique, and the impact on work or studies can vary. It is crucial to consult with healthcare professionals, such as stoma care nurses or gastroenterologists, who can provide personalized advice based on individual circumstances. These specialists can offer guidance on managing the stoma, maintaining overall health, and finding practical solutions to ensure success in work or educational pursuits.

**11, After getting a stoma, is it possible to have children?**

(1) Yes, it is generally possible to have children after getting a stoma. Having a stoma, which is a surgically created opening in the abdomen to divert waste from the digestive or urinary system, does not necessarily impact a person's ability to conceive or carry a pregnancy.

(2) The ability to have children after getting a stoma depends on various factors, including the reason for the stoma, the specific medical condition or procedure involved, and individual circumstances. In some cases, the underlying condition that led to the stoma might affect fertility or pregnancy outcomes. It is important to consult with healthcare professionals, such as a gastroenterologist, urologist, or gynecologist, who can provide personalized advice based on the specific situation.

(3) In certain situations, having a stoma might require additional considerations during pregnancy. For example, if the stoma is located in the abdomen, changes in the body during pregnancy, such as weight gain and increased pressure on the abdomen, could potentially affect the function or placement of the stoma. Close monitoring by healthcare professionals is crucial to ensure the stoma remains functional and complications are addressed promptly.

(4) Depending on the type of stoma and the medical condition, certain adjustments might be necessary during pregnancy. For instance, if a person has an ileostomy or colostomy, the size and shape of the stoma opening might change as the body undergoes transformations during pregnancy. This could require adjustments to the stoma appliance or consultation with a stoma care nurse to ensure proper management.

(5) Overall, while having a stoma might introduce additional considerations, many individuals with stomas have successfully conceived, carried pregnancies to term, and become parents. It is important to work closely with healthcare professionals to address any concerns, optimize stoma care, and ensure a safe and healthy pregnancy journey.

Rep2:

(1) Yes, it is generally possible to have children after getting a stoma. Having a stoma, which is an opening created in the abdomen to divert the flow of waste out of the body, does not directly impact fertility or the ability to conceive. However, there may be some considerations and potential challenges that individuals with a stoma need to keep in mind when planning to have children.

(2) One important aspect to consider is the underlying condition that led to the need for a stoma. If the underlying condition affects fertility or reproductive organs, it may pose challenges in conceiving naturally. Certain medical conditions or surgeries that led to the stoma placement can affect the reproductive system, and it is essential to discuss these concerns with a healthcare professional or a specialist in fertility and reproductive health.

(3) Additionally, the physical and emotional demands of living with a stoma can also affect an individual's decision to have children. It is crucial to have open and honest conversations with healthcare providers, including stoma care nurses and surgeons, who can provide guidance and support in managing the stoma and the potential impact it may have on pregnancy and child-rearing.

(4) Depending on the type of stoma, such as a colostomy, ileostomy, or urostomy, individuals may need to consider how their stoma and associated appliances can be managed during pregnancy. The changing shape and size of the abdomen during pregnancy can require adjustments in stoma appliances and potentially pose challenges in maintaining a secure seal. Stoma care professionals can provide advice on choosing appropriate stoma supplies and techniques to ensure comfort and proper functioning during pregnancy.

(5) It is also important to discuss the potential impact of pregnancy and childbirth on the stoma itself. Pregnancy can lead to changes in the body, including weight gain and hormonal fluctuations, which may affect the stoma's placement or function. Consulting with healthcare professionals who are experienced in stoma care can help individuals plan and prepare for these changes.

(6) Lastly, pregnancy and parenting can be physically and emotionally demanding, and it is important for individuals with a stoma to have a support system in place. This may include family, friends, healthcare professionals, or support groups that can provide assistance and guidance throughout the journey of having children.

**12,** **After having an intestinal stoma, is it possible to travel?**

(1) Yes, it is generally possible to travel after having an intestinal stoma. People with intestinal stomas, such as colostomies or ileostomies, can travel and enjoy a variety of destinations and experiences. However, it is important to plan and prepare accordingly to ensure a smooth and comfortable trip.

(2) When considering travel with an intestinal stoma, it is essential to pack an adequate supply of stoma supplies, including pouches, adhesive products, and any other accessories specific to the individual's needs. It is advisable to carry more supplies than needed to account for unexpected delays or changes in plans.

(3) Additionally, it is crucial to research and identify healthcare facilities along the travel route or at the destination. This way, if any issues or concerns arise related to the stoma or its management, medical assistance can be sought promptly. It may also be helpful to carry a medical summary or letter from the healthcare provider explaining the individual's condition and the necessity of the stoma supplies.

(4) Planning travel itineraries with consideration for the availability of restrooms and suitable accommodations is important. Some individuals may prefer accommodations with private bathrooms or rooms closer to restroom facilities for convenience. Researching restaurants and attractions with accessible restroom facilities can also help ensure a more comfortable experience while traveling.

(5) It is advisable to inform the transportation provider about the presence of a stoma before the journey, especially for long-haul flights or train rides. They may be able to provide special accommodations or make arrangements to facilitate a smoother travel experience.

(6) Staying hydrated and maintaining a balanced diet during travel is essential for overall health and stoma function. It is important to drink plenty of water and avoid foods that may cause digestive issues or affect the stoma output. Carrying water and snacks while traveling can help in situations where suitable options may not be readily available.

(7) Lastly, it is essential to listen to the body and take breaks as needed during travel. Long periods of sitting or immobility can potentially affect circulation and overall comfort. Stretching and moving around periodically can help prevent any discomfort or complications.

Rep2:

(1) Yes, it is generally possible to travel after having an intestinal stoma. Traveling with an intestinal stoma may require some additional planning and preparation, but it should not prevent you from enjoying various types of travel experiences.

(2) One important aspect to consider when traveling with an intestinal stoma is managing your supplies. It is crucial to ensure you have an adequate supply of stoma pouches, adhesive products, and any other necessary accessories for the duration of your trip. It is recommended to carry more supplies than you anticipate needing, in case of unexpected delays or emergencies.

(3) Another consideration is transportation arrangements. Depending on the mode of transportation, you may need to inform the airline, train company, or cruise line about your medical condition in advance. This will allow them to provide any necessary accommodations, such as access to restroom facilities or storage for your medical supplies.

(4) When planning your itinerary, it can be helpful to research the availability of medical facilities along your travel route. Identifying hospitals or healthcare providers that are familiar with stoma care can give you peace of mind and ensure you have support if any issues arise during your trip.

(5) It is also advisable to carry a medical information card or bracelet that identifies you as a person with a stoma. This can be helpful in case of emergencies, as it provides essential information to medical professionals who may be unfamiliar with your condition.

(6) Depending on your destination, you may need to consider the climate and adjust your stoma care routine accordingly. Extreme heat or cold can affect the adhesion of your pouch, so it may be necessary to make adjustments to ensure a secure fit.

(7) Finally, remember to plan for relaxation and enjoyment during your trip. Having an intestinal stoma should not limit your ability to participate in activities or explore new places. However, it is important to listen to your body and take breaks as needed to maintain your overall well-being.

**13, After having an intestinal stoma, is it necessary to only use an ostomy bag?**

(1) After having an intestinal stoma, using an ostomy bag is generally recommended. An ostomy bag is a specialized medical device designed to collect waste from the stoma, which is the opening on the abdomen created during the surgery. The bag is attached securely around the stoma to collect feces or urine, depending on the type of stoma (colostomy, ileostomy, or urostomy). It provides a hygienic and convenient way to manage waste output, preventing it from coming into contact with the skin and clothing. Ostomy bags come in various sizes, shapes, and styles to suit individual needs and preferences.

(2) While using an ostomy bag is the most common and effective method of managing waste after an intestinal stoma, there are alternative options available. One such option is stoma caps or covers. These are small pouches designed for individuals with low or controlled fecal output. Stoma caps offer a lower-profile alternative to a full-size ostomy bag and can be worn discreetly under clothing. However, they may not provide the same level of security and confidence as a regular ostomy bag, particularly for individuals with higher output or unpredictable bowel movements.

(3) Another option for managing waste after an intestinal stoma is irrigation. This method is typically used in colostomies, where the large intestine is diverted to the abdominal wall. Irrigation involves introducing water into the stoma to stimulate bowel movement and empty the colon at regular intervals. By establishing a predictable bowel routine, individuals may be able to avoid wearing an ostomy bag permanently. However, irrigation is not suitable for all individuals, and it requires proper training, guidance, and coordination with healthcare professionals to ensure safety and effectiveness.

(4) In rare cases, some individuals may undergo continent procedures or surgical revisions to create an internal pouch or reservoir that can be self-managed without the need for an external ostomy bag. These procedures, such as continent ileostomy or continent urinary diversion, involve reconstructing the intestinal or urinary system to provide a mechanism for waste storage and controlled elimination. However, these procedures are complex and not commonly performed, and their suitability depends on individual factors and medical considerations.

Rep2:

(1) Yes, after having an intestinal stoma, it is generally necessary to use an ostomy bag. An ostomy bag, also known as an ostomy pouch or appliance, is designed to collect waste material from the stoma. The stoma is a surgically created opening on the abdomen that allows waste products to exit the body when a person's normal bowel or bladder function is compromised. The ostomy bag is securely attached around the stoma, providing a sealed environment to collect the waste. It helps maintain hygiene, prevent leakage, and allows for easy disposal of the waste, promoting comfort and convenience for the individual with a stoma.

(2) While there may be alternative options available, such as stoma caps or irrigation systems, the use of an ostomy bag is commonly recommended and widely used. Stoma caps are small covers that can be worn over the stoma, but they are typically used for individuals with a well-formed and predictable stoma output. Stoma irrigation involves flushing the bowel with water through the stoma to regulate bowel movements, but it is only suitable for specific individuals who have undergone specific surgical procedures and have the ability to perform irrigation. These alternatives are not suitable or applicable for everyone with a stoma, and the ostomy bag remains the most practical and reliable option for managing waste output.

(3) Ostomy bags come in various types and sizes to accommodate individual needs and preferences. They can be one-piece or two-piece systems, with the former having the bag and adhesive barrier attached together and the latter consisting of a separate bag and adhesive wafer that is attached to the skin around the stoma. Some ostomy bags are drainable, allowing the contents to be emptied without removing the bag entirely, while others are closed-end and need to be replaced when full. The bags are usually made of odor-resistant materials and have filters to release gas while containing any odors. The availability of different options ensures that individuals can find a bag that suits their lifestyle and promotes comfort and confidence.

(4) It is important to consult with a healthcare professional, such as an ostomy nurse, to determine the most suitable ostomy management method for an individual's specific situation. They can assess the stoma, provide guidance on proper ostomy care, and recommend the appropriate type of ostomy bag based on factors such as stoma size, output consistency, and individual preferences. Regular follow-up visits with an ostomy nurse or healthcare provider can also help address any concerns, provide ongoing support, and ensure the optimal management of the stoma.

**14, What are the possible complications that may occur in patients with intestinal stomas after discharge?**

1. Stoma blockage: One possible complication after discharge for patients with intestinal stomas is stoma blockage. This occurs when the opening of the stoma becomes obstructed, preventing the passage of stool or gas. Blockages can be caused by various factors such as inadequate chewing of food, dehydration, inadequate stoma size, or the presence of scar tissue. Symptoms may include abdominal pain, swelling around the stoma, and a decrease in output from the stoma.

2. Skin irritation or breakdown: Another potential complication is skin irritation or breakdown around the stoma site. The skin around the stoma is exposed to digestive enzymes and waste products, which can irritate and damage the skin. If proper care is not taken, the skin may become red, inflamed, or develop ulcers. Skin breakdown can be caused by factors like poor hygiene, improper fitting of the stoma appliance, or leakage of stool or digestive fluids onto the skin.

3. Stoma retraction or prolapse: Stoma retraction or prolapse can occur in some cases. Stoma retraction happens when the stoma sinks below the level of the abdominal wall, making it difficult to manage the stoma appliance properly. Stoma prolapse, on the other hand, is the protrusion of the stoma beyond the level of the abdominal wall. These conditions can cause challenges in achieving a secure seal with the stoma appliance, leading to leakage or difficulty in maintaining proper hygiene.

4. Stoma herniation or parastomal hernia: Patients with intestinal stomas are also at risk of developing hernias around the stoma site. A stoma herniation or parastomal hernia occurs when a portion of the intestine or other abdominal contents protrudes through a weakness in the abdominal wall around the stoma. This can cause discomfort, pain, and may require surgical intervention to repair.

5. Psychological and emotional challenges: Patients with intestinal stomas may also experience psychological and emotional challenges after discharge. Adjusting to life with a stoma can be emotionally challenging, leading to feelings of self-consciousness, anxiety, or depression. It is important to provide support, education, and counseling to help patients cope with these emotional aspects and promote their overall well-being.

Rep2:

1. Stoma prolapse: Stoma prolapse refers to the protrusion or telescoping of the stoma above the skin level. It can occur when the stoma becomes elongated or extends beyond its normal position. Prolapse can lead to difficulties in maintaining a proper seal with the stoma appliance, causing leakage, skin irritation, and discomfort for the patient

2. Stoma retraction: Stoma retraction happens when the stoma sinks below the skin level. This can occur due to changes in abdominal pressure, stoma size, or the presence of scar tissue. Retraction can make it challenging to secure the stoma appliance properly, leading to leakage and potential skin irritation.

3. Stoma stenosis: Stoma stenosis refers to the narrowing of the stoma opening, which can obstruct the passage of stool or intestinal contents. It may occur due to scarring, inflammation, or improper healing of the stoma. Stenosis can lead to blockages, difficulty in pouching, and potential abdominal discomfort for the patient.

4. Peristomal skin complications: The peristomal skin, the area surrounding the stoma, is prone to various complications. These may include skin irritation, redness, itching, and inflammation. Factors contributing to peristomal skin problems include leakage, allergies to stoma appliances or adhesives, inadequate hygiene, or underlying skin conditions.

5. Parastomal hernia: A parastomal hernia is the protrusion of an organ or tissue through the weakened abdominal wall adjacent to the stoma site. It occurs due to the weakened muscles around the stoma, often resulting from surgical incisions. Parastomal hernias can cause discomfort, difficulty in appliance fitting, and may require surgical intervention in severe cases.

6. Stoma necrosis: In rare cases, stoma necrosis can occur due to inadequate blood supply to the stoma. This can lead to tissue death and requires immediate medical attention. Causes of stoma necrosis may include surgical complications, infection, or underlying health issues that affect blood circulation.

7. Psychosocial impact: Patients with intestinal stomas may experience psychosocial challenges and emotional distress. Adjusting to the presence of a stoma, managing self-care routines, body image concerns, and potential limitations in daily activities can all impact a patient's well-being. Support from healthcare professionals, stoma support groups, and counseling services can be beneficial in addressing these psychological aspects.

**15, What are the symptoms of irritant (fecal) dermatitis? How should it be managed?**

(1) Irritant dermatitis, also known as fecal dermatitis or diaper dermatitis, is a skin condition that occurs when the skin comes into contact with irritants present in feces. The symptoms of irritant dermatitis can vary but typically include:

- Redness and inflammation of the skin in the diaper area

- Raised, bright red rash

- Scaling or peeling of the skin

- Swelling and tenderness in the affected area

- Discomfort or pain when the diaper area is touched or rubbed

- Development of small red bumps or pimples

(2) To manage irritant dermatitis effectively, it is essential to follow these general guidelines:

- Keep the diaper area clean and dry: Change diapers frequently and use gentle wipes or warm water to clean the area. Pat the skin dry rather than rubbing it.

- Use a barrier cream or ointment: Apply a thick layer of a protective barrier cream or ointment, such as zinc oxide or petroleum jelly, to create a barrier between the skin and irritants. This helps prevent further irritation and aids in the healing process.

- Avoid irritants: Avoid using harsh soaps, wipes, or scented products on the diaper area, as they can worsen the condition. Opt for mild, fragrance-free cleansers.

- Allow for diaper-free time: Give your baby some diaper-free time each day to allow the skin to breathe and reduce moisture buildup.

- Use breathable diapers: Choose diapers that are breathable and made of materials that allow air circulation. Avoid tight-fitting diapers that can trap moisture and heat.

- Be mindful of diet: If your baby is consuming solid foods, certain foods may contribute to increased stool acidity and worsen the condition. Consult a healthcare professional or pediatrician for guidance on adjusting the diet if necessary.

(3) If the symptoms persist or worsen despite home care, it is important to seek medical advice. A healthcare professional may recommend the following:

- Prescription ointments or creams: In some cases, a healthcare provider may prescribe a medicated cream or ointment containing hydrocortisone or other topical steroids to reduce inflammation and promote healing.

- Antifungal treatment: If a fungal infection is suspected or present, antifungal creams or ointments may be prescribed to treat the underlying infection.

- Evaluation for other conditions: If the symptoms are severe, recurrent, or not responding to treatment, further evaluation may be needed to rule out other skin conditions or infections.

- Counseling on proper diapering techniques: A healthcare professional can provide guidance on proper diapering techniques, including how to fit diapers correctly and prevent leakage or excessive moisture.

Rep2:

(1) Irritant dermatitis, also known as fecal dermatitis or diaper dermatitis, is a common condition that affects the skin in the diaper area. It is characterized by inflammation and irritation of the skin caused by contact with urine or feces, prolonged exposure to moisture, friction, and the occlusive environment created by diapers. The symptoms of irritant dermatitis include redness, swelling, and soreness in the diaper area, along with possible development of small red bumps, blisters, or peeling skin. The affected area may also feel warm to the touch and be accompanied by discomfort or pain.

(2) Managing irritant dermatitis involves several general measures to promote healing and prevent further irritation. First and foremost, maintaining good hygiene is essential. The diaper area should be cleaned gently with warm water and a mild, fragrance-free cleanser during diaper changes. Patting the area dry or allowing it to air-dry before putting on a fresh diaper can help reduce moisture. Applying a barrier cream or ointment containing zinc oxide can provide a protective layer and help soothe the skin.

(3) It is important to ensure frequent diaper changes to prevent prolonged contact with urine or feces. Changing diapers promptly after they become soiled can minimize exposure to irritants. Using diapers that are breathable and absorbent, and avoiding tight-fitting diapers or clothing that can cause friction, can help reduce irritation. In some cases, switching to cloth diapers or using disposable diapers without fragrances or other potential irritants may be beneficial.

(4) Additionally, allowing the diaper area to have some diaper-free time can promote healing. Exposing the skin to air can help dry out moisture and facilitate the healing process. It is advisable to avoid using wipes that contain alcohol or fragrances, as they can further irritate the skin. If the symptoms persist or worsen despite home care measures, it is recommended to consult a healthcare professional for further evaluation and appropriate treatment.

**16, For patients with an ileostomy, what are the symptoms of allergic dermatitis and how should it be treated?**

(1) Patients with an ileostomy who develop allergic dermatitis may experience symptoms such as redness, itching, and inflammation around the stoma site. The skin may also become dry, cracked, and may develop blisters or bumps. In severe cases, there may be oozing or bleeding from the affected area.

(2) To treat allergic dermatitis in patients with an ileostomy, it is important to identify and avoid the allergen that is causing the reaction. The skin should be kept clean and dry, and any irritants or potential allergens should be avoided. Topical treatments such as barrier creams, corticosteroids, and antihistamines may be used to relieve the symptoms and reduce inflammation. In some cases, systemic medications or immunosuppressants may be prescribed if the dermatitis is severe or persistent. It is important to consult with a healthcare provider for proper diagnosis and treatment of allergic dermatitis in patients with an ileostomy.

Rep2:

(1) Allergic dermatitis is a type of skin reaction that can occur in patients with an ileostomy due to contact with an allergen. Symptoms of allergic dermatitis can include:

1. Itching and burning sensation on the skin around the stoma

2. Redness and swelling of the skin around the stoma

3. Blisters or hives

4. Scaling or flaking of the skin

5. Pain or discomfort in the affected area

(2) Treatment for allergic dermatitis typically involves identifying and avoiding the allergen that is causing the reaction. Patients may need to change their ostomy appliance or the type of adhesive used to prevent further contact with the allergen. In addition, topical treatments such as corticosteroid creams or ointments may be prescribed to reduce inflammation and relieve symptoms. If the allergic dermatitis is severe, oral antihistamines or systemic corticosteroids may be necessary to control the reaction. It is important for patients with an ileostomy to work closely with their healthcare provider to manage allergic dermatitis and prevent further complications.

**17, What are the symptoms of peristomal hernia? How should it be managed?**

(1) Peristomal hernia refers to the protrusion of an organ or tissue through the weakened area around a stoma, which is a surgically created opening in the abdomen for waste elimination. The symptoms of peristomal hernia can vary but commonly include:

- Noticeable bulge or swelling around the stoma site

- Discomfort or pain in the abdominal area

- Changes in the size, shape, or appearance of the stoma

- Difficulty in maintaining a proper seal of the ostomy appliance

- Leakage or skin irritation around the stoma

- Digestive issues such as constipation or bowel obstruction

(2) The management of peristomal hernia depends on the severity of the symptoms and the impact on the individual's quality of life. In mild cases, non-surgical approaches may be attempted, which include:

- Wearing a supportive ostomy belt or binder to provide abdominal support and reduce hernia bulging

- Modifying daily activities to avoid heavy lifting or straining that can worsen the hernia

- Using a specialized hernia support garment or stoma hernia belt that provides targeted support around the stoma site

(3) If conservative measures are ineffective or the hernia is causing significant symptoms, surgical intervention may be necessary. Surgical management options for peristomal hernia include:

- Hernia repair with stoma relocation: The hernia is repaired, and the stoma is moved to a different location on the abdomen, typically away from the weakened area.

- Hernia repair with mesh placement: A synthetic mesh is used to reinforce the weakened abdominal wall and support the stoma.

- Stoma reinforcement with mesh: In cases where stoma relocation is not feasible, a mesh is placed around the stoma to provide support and reduce the risk of hernia recurrence.

(4) The choice of surgical approach depends on factors such as the size and complexity of the hernia, overall health condition, and the surgeon's expertise. It is important for individuals with peristomal hernia to consult with a healthcare professional, preferably a surgeon specializing in ostomy care, to determine the most appropriate management strategy based on their specific circumstances.

Rep2:

(1) Peristomal hernia refers to the protrusion of organs or tissues through a weakened area around a stoma, which is an opening created surgically on the abdomen to divert bodily waste. The symptoms of peristomal hernia can vary, but commonly include:

- A visible bulge or swelling around the stoma site.

- Discomfort or pain in the abdominal area, especially during physical activity or when lifting objects.

- Difficulty with the placement and adherence of the ostomy appliance.

- Changes in the appearance or function of the stoma, such as retraction or prolapse.

(2) Management of peristomal hernia typically involves a combination of non-surgical and surgical approaches, depending on the severity of the hernia and the patient's overall health.

Non-surgical management options may include:

- Wearing a supportive belt or ostomy hernia support garment to provide gentle compression and support around the stoma.

- Modifying daily activities to minimize strain on the abdominal muscles.

- Working with an ostomy nurse to ensure proper fitting and placement of the ostomy appliance.

- Implementing dietary modifications to maintain a healthy weight and reduce straining during bowel movements.

(3) In cases where non-surgical methods are not effective, or the hernia is large, surgical intervention may be recommended. Surgical management options for peristomal hernia include:

- Stoma relocation: The stoma is surgically moved to a different location on the abdomen, often away from the weakened area, to reduce the risk of recurrence.

- Hernia repair: The herniated tissues are surgically pushed back into place, and the weakened abdominal wall is reinforced with mesh or sutures to provide additional support.

- Hernia repair with mesh: A synthetic mesh is placed over the weakened area to reinforce the abdominal wall and prevent the hernia from recurring.

(4) The choice of management approach depends on various factors, such as the patient's overall health, the size and severity of the hernia, and the surgeon's expertise. It is crucial for individuals with peristomal hernia to consult with their healthcare provider or an ostomy specialist to determine the most appropriate management strategy for their specific case. Regular follow-up and ongoing care are essential to monitor the hernia and ensure optimal stoma function and quality of life.

**The Venous Cares**

**1, Can chemotherapy be administered through a central venous catheter?**

(1) Yes, chemotherapy can be administered through a central venous catheter. A central venous catheter is a flexible tube that is inserted into a large vein, usually in the chest or arm. It allows direct access to the central venous system, which includes the superior vena cava and right atrium of the heart. This type of catheter is commonly used for delivering chemotherapy because it provides a reliable and efficient route for medication administration.

(2) Administering chemotherapy through a central venous catheter offers several advantages. Firstly, the catheter can stay in place for an extended period, allowing repeated administrations of chemotherapy without the need for repeated needle sticks. This is particularly beneficial for patients who require frequent or long-term chemotherapy treatments. Secondly, the large size of the central veins allows for rapid dilution and distribution of the medication, improving the effectiveness of chemotherapy. Lastly, the central venous catheter can handle the administration of chemotherapy drugs that may be too irritating or damaging to smaller peripheral veins.

(3) The process of administering chemotherapy through a central venous catheter involves several steps. The catheter is inserted by a healthcare professional into a suitable vein, often guided by imaging techniques such as fluoroscopy or ultrasound. Once in place, the catheter is secured and connected to an infusion system, which may include an infusion pump. The chemotherapy medication is then prepared according to the prescribed dose and administered through the catheter. The healthcare team closely monitors the patient during the infusion to ensure proper medication delivery and to manage any potential side effects.

(4) It is important to note that the use of a central venous catheter for chemotherapy carries some risks and requires careful monitoring. Infections can occur at the catheter insertion site or along the catheter, leading to serious complications. Blood clots, known as deep vein thrombosis, can also develop around the catheter. To minimize these risks, proper catheter care

Rep2:

(1) Yes, chemotherapy can be administered through a central venous catheter. A central venous catheter is a thin, flexible tube that is inserted into a large vein in the chest, neck, or groin. It is used to deliver medications, fluids, and nutrition directly into the bloodstream. Chemotherapy drugs can be infused through the central venous catheter, allowing the medication to be distributed quickly throughout the body.

(2) Central venous catheters provide several advantages for administering chemotherapy. Firstly, they can be used for long-term treatment, as they can remain in place for weeks or even months. This eliminates the need for repeated needle sticks for each chemotherapy session. Secondly, central venous catheters can handle high volumes and concentrations of chemotherapy drugs, which may not be suitable for peripheral veins. This allows for more effective and efficient delivery of the medication. Additionally, the catheter can be accessed easily, making it convenient for both patients and healthcare providers during chemotherapy treatment.

(3) Another benefit of using a central venous catheter for chemotherapy administration is that it minimizes the risk of damage to peripheral veins. Chemotherapy drugs can be caustic and irritate the delicate veins in the arms or hands, leading to pain, inflammation, or even thrombosis. By utilizing a central venous catheter, the medication is delivered directly into a larger, more robust vein, reducing the likelihood of vein damage and associated complications.

(4) In addition, central venous catheters allow for multiple treatment options. They can be used for continuous infusion chemotherapy, where the medication is delivered over an extended period, or for intermittent infusions, where the drug is administered in cycles. The flexibility provided by central venous catheters enables healthcare providers to tailor the chemotherapy regimen to the specific needs of each patient.

**2, What are the commonly used central venous catheters in chemotherapy infusion, namely CVC, PICC, and PORT?**

(1) Central Venous Catheter (CVC): CVC is a commonly used central venous catheter in chemotherapy infusion. It is inserted into a large vein, usually the subclavian vein or jugular vein, and the tip of the catheter resides in the superior vena cava or right atrium. CVCs have multiple lumens that allow for the administration of chemotherapy drugs, fluids, and blood products. They are typically used for short-term or long-term chemotherapy treatment and provide a reliable access point for frequent medication administration or blood draws.

(2) Peripherally Inserted Central Catheter (PICC): PICC lines are another type of central venous catheter commonly used in chemotherapy infusion. They are inserted through a peripheral vein, typically in the arm, and advanced until the tip lies in the superior vena cava. PICC lines are longer than traditional CVCs, allowing them to reach the central veins while being inserted from a peripheral access site. They are more comfortable for patients compared to CVCs and offer a lower risk of complications such as infection or pneumothorax. PICC lines are suitable for long-term chemotherapy treatment and can remain in place for several weeks or months.

(3) Port-a-Cath (PORT): A Port-a-Cath, commonly referred to as a PORT, is another type of central venous catheter used in chemotherapy infusion. It consists of a reservoir or port that is surgically implanted beneath the skin, usually in the chest or upper arm, and connected to a catheter that is inserted into a central vein. The port remains completely under the skin when not in use and can be accessed with a special needle for chemotherapy infusion or blood sampling. PORTs are beneficial for patients who require frequent or long-term chemotherapy treatment. They offer a reduced risk of infection and increased patient comfort compared to other catheter types.

Rep2:

(1) Central Venous Catheter (CVC):

Central Venous Catheters (CVCs) are commonly used for chemotherapy infusion. These catheters are inserted into a large central vein, such as the superior vena cava or the internal jugular vein. CVCs have multiple lumens, allowing for the administration of chemotherapy drugs, fluids, and blood products. They are available in different lengths and sizes, and they can remain in place for an extended period, ranging from weeks to months. CVCs require surgical or radiologic placement and are typically used in patients who require frequent or long-term access for chemotherapy treatment.

(2) Peripherally Inserted Central Catheter (PICC):

Peripherally Inserted Central Catheters (PICCs) are another commonly used option for chemotherapy infusion. Unlike CVCs, PICCs are inserted peripherally, typically in the upper arm, and advanced until the catheter tip reaches the central veins. This procedure is usually performed by specially trained nurses or radiologists. PICCs are thinner and more flexible than CVCs, and they can be used for a variety of medical treatments, including chemotherapy. They are generally suitable for patients who require intermittent or short-to-medium-term access for chemotherapy administration.

(3) Port-A-Cath or Port:

A Port-A-Cath, commonly referred to as a Port, is a type of central venous access device used for chemotherapy infusion. It consists of a small chamber placed beneath the skin and connected to a catheter that is inserted into a central vein. Ports have a self-sealing septum on the skin surface that can be accessed using a special needle, allowing for repeated infusions and blood draws without the need for repeated needle insertions. Ports are often used in patients who require long-term chemotherapy treatment, as they offer a convenient and discreet option for accessing the bloodstream. They are typically placed under local anesthesia in an outpatient setting.

**3, What is extravasation of chemotherapy drugs?**

(1) Extravasation of chemotherapy drugs refers to the leakage of these powerful medications from the intended vein into the surrounding tissues during their administration. This can occur when a needle or catheter used to deliver the drugs accidentally punctures or dislodges a blood vessel, causing the medication to escape its intended pathway.

(2) When extravasation occurs, chemotherapy drugs, which are designed to kill cancer cells, can cause damage to healthy tissues. These medications are often toxic and can lead to severe local tissue injury, resulting in pain, swelling, inflammation, and potential long-term complications. The extent of damage depends on various factors, including the type and amount of drug involved, the duration of exposure, and the location of the extravasation.

(3) Prompt recognition and management of extravasation are crucial to minimize the harmful effects. Healthcare professionals closely monitor the infusion site during chemotherapy administration to detect signs of extravasation early. If extravasation is suspected, the infusion is typically stopped immediately, and the affected area is carefully assessed. Depending on the specific drug and severity of the extravasation, various interventions may be employed to mitigate the damage, such as applying cold or warm compresses, administering antidotes or neutralizing agents, or even performing surgical interventions in severe cases.

(4) In some cases, healthcare providers may use specific techniques to prevent extravasation, such as using central venous catheters or implantable ports for drug administration. Additionally, patient education plays a crucial role in helping individuals identify early signs of extravasation at home and promptly seek medical attention if it occurs. Regular communication between patients and healthcare providers is essential to ensure a proactive approach to managing and minimizing the risks associated with extravasation during chemotherapy treatment.

Rep2:

(1) Extravasation of chemotherapy drugs refers to the accidental leakage or escape of these powerful medications from the intended target area into the surrounding tissues. This can occur when a chemotherapy drug is administered intravenously and inadvertently infiltrates the surrounding tissues instead of flowing into the bloodstream. Extravasation can cause significant damage to the tissues and can lead to severe complications if not promptly managed.

(2) When extravasation of chemotherapy drugs occurs, the toxic substances meant to target cancer cells enter the surrounding healthy tissues. This can result in local tissue damage, inflammation, pain, and potential long-term complications such as scarring, impaired mobility, or nerve damage. The severity of the damage depends on factors like the type of drug, its concentration, the volume infiltrated, and the duration of exposure.

(3) Prompt recognition and appropriate management of chemotherapy extravasation are crucial to minimize the potential harm. Healthcare professionals who administer chemotherapy are trained to monitor the infusion site closely and promptly identify any signs of extravasation, such as swelling, pain, redness, or resistance to flow during administration. Once extravasation is suspected, the drug infusion is stopped, and specific steps are taken to mitigate the damage and promote healing.

(4) When extravasation is suspected, the healthcare provider may attempt to aspirate as much of the drug as possible from the affected area using a syringe. The site is then carefully evaluated to determine the extent of tissue damage. Depending on the severity, various interventions may be implemented, such as applying cold or warm compresses, elevating the affected limb, administering antidotes or neutralizing agents, and providing appropriate wound care. In some cases, surgical consultation may be necessary for more extensive tissue debridement or reconstructive procedures.

(5) After managing the acute phase, healthcare providers closely monitor the affected area for signs of complications or delayed tissue damage. Patients are educated on proper wound care and advised to report any persistent pain, swelling, or changes in sensation. Follow-up appointments are scheduled to assess the healing progress and ensure optimal recovery. Additionally, healthcare professionals continue to refine their techniques and guidelines to minimize the risk of chemotherapy extravasation and improve patient safety.

**4, Does the presence of a PICC line affect daily life?**

(1) Yes, the presence of a PICC line can affect daily life to some extent. A peripherally inserted central catheter (PICC) line is a thin, flexible tube that is inserted into a vein in the arm and threaded up into a larger vein near the heart. It is used to deliver medications, fluids, or nutrients directly into the bloodstream over an extended period. While it provides numerous benefits in terms of convenience and prolonged treatment access, there are several considerations that can impact daily life.

(2) One aspect that can be affected is physical comfort. The insertion site of the PICC line may cause discomfort, soreness, or tenderness, especially during the initial period after insertion. It may restrict some arm movements, making certain activities more challenging. However, with time and proper care, individuals often adapt and the discomfort lessens.

(3) Another factor is the need for maintaining strict hygiene and care around the PICC line. It is crucial to keep the insertion site clean, dry, and protected to prevent infections. This involves regular dressing changes and taking precautions while bathing or participating in activities that might expose the line to potential contaminants. Adhering to these precautions may require adjustments to daily routines and additional time for proper care.

(4) Additionally, the presence of a PICC line might have an impact on personal aesthetics and clothing choices. The line is typically external and may be visible, leading individuals to modify their clothing preferences to accommodate the presence of the line. This can include wearing loose-fitting or sleeveless clothing to provide easy access for dressing changes and maintenance.

(5) Finally, having a PICC line necessitates a level of vigilance and awareness in daily activities. It is important to be mindful of the line's position and ensure it remains secure to avoid accidental dislodgment. This might affect participation in certain physical activities or sports that carry a higher risk of line displacement. However, many individuals can still engage in a range of activities with precautions in place.

Rep2:

(1) Yes, the presence of a PICC line can affect daily life. A peripherally inserted central catheter (PICC) line is a long, flexible tube that is inserted into a vein in the arm and advanced until the tip reaches a large vein near the heart. It is typically used for long-term intravenous therapy, such as administering medications, fluids, or nutrients, or for blood draws. The presence of a PICC line requires certain adjustments and precautions in daily activities to ensure its functionality and reduce the risk of complications.

(2) One of the ways a PICC line can impact daily life is through restrictions on physical activities. Engaging in strenuous exercises or activities that involve intense arm movements may pose a risk of dislodging or damaging the PICC line. It is important to follow healthcare provider instructions and avoid activities that could put excessive stress on the line. This limitation may affect one's ability to participate in certain sports or physical tasks.

(3) Another aspect affected by a PICC line is personal hygiene. Care must be taken during bathing or showering to ensure that the PICC line remains dry and clean. Special precautions, such as covering the insertion site with a waterproof dressing or using a protective sleeve, may be necessary. Additionally, extra care should be taken when changing clothes or performing tasks that involve close contact with the line to prevent accidental tugging or pulling.

(4) Dressing maintenance is an essential aspect of daily life with a PICC line. The dressing covering the insertion site needs regular attention to ensure it stays clean and secure. Healthcare providers may provide specific instructions on when and how to change the dressing, as well as any additional precautions to prevent infection or irritation. Adhering to proper dressing care helps maintain the integrity of the PICC line and reduces the risk of complications.

(5) Emotional and psychological considerations can also arise from having a PICC line. Some individuals may experience anxiety or discomfort related to the presence of the line, especially if it is their first time or if they have had previous negative experiences with medical procedures. It is important to address any concerns with healthcare providers who can provide reassurance, support, or additional resources to help manage any emotional impact.

(6) Regular follow-up visits and maintenance of the PICC line may require adjustments in daily schedules. Healthcare providers often need to assess the PICC line's functionality, change dressings, and perform necessary flushes or other maintenance tasks. These visits may require travel to healthcare facilities and can potentially disrupt one's regular routine or work schedule.

**5, What are the things to pay attention to in the daily maintenance of PICC?**

(1) Proper dressing care: It is important to pay attention to the dressing covering the PICC line. The dressing should be clean, dry, and intact. Any signs of loosening, soiling, or moisture should be addressed promptly to prevent infection. Regularly changing the dressing according to healthcare provider instructions is essential for maintaining PICC line hygiene.

(2) Flushing the line: Flushing the PICC line regularly is crucial to maintain its patency and prevent blockages. Flushing with saline solution before and after medication administration or as per healthcare provider instructions helps to keep the line clear of any clot formation or debris. It is important to use appropriate flushing techniques and sterile equipment to minimize the risk of contamination.

(3) Monitoring for signs of infection: Monitoring the PICC line insertion site for any signs of infection is vital. Redness, swelling, warmth, or drainage around the insertion site should be reported to the healthcare provider immediately. It is important to follow the recommended guidelines for cleaning the site and keeping it dry to minimize the risk of infection.

(4) Securing the line: Properly securing the PICC line is essential to prevent accidental dislodgement or migration. Using securement devices or dressings recommended by the healthcare provider helps to keep the line in place. Regularly checking the stability of the line and addressing any issues promptly can prevent complications.

(5) Avoiding excessive force or tension: It is important to avoid any excessive force or tension on the PICC line. Care should be taken while moving or handling the line to prevent accidental pulling or tugging, which can lead to line dislodgement or damage. Educating the patient and caregivers about the importance of gentle handling and precautions can help ensure the line's integrity.

(6) Regular assessments and documentation: Regular assessments of the PICC line, including checking for signs of complications or blockages, should be conducted. Any changes in the line's condition or the patient's symptoms should be documented and reported to the healthcare provider. Proper documentation helps in tracking the line's progress and identifying any issues that may require intervention.

(7) Patient and caregiver education: Providing thorough education to the patient and caregivers about PICC line maintenance is crucial. They should understand the importance of proper hygiene, dressing care, flushing techniques, and signs of complications. Educating them about the do's and don'ts and providing written instructions or resources can empower them to actively participate in the daily maintenance of the PICC line.

(8) Regular follow-up visits: It is important to attend regular follow-up visits with the healthcare provider to assess the PICC line's condition and address any concerns. These visits allow for proper monitoring, line assessment, and potential adjustments in the care plan as needed. Following the recommended follow-up schedule helps ensure the ongoing maintenance and care of the PICC line.

Rep2:

(1) Flushing: Regular flushing of the PICC (Peripherally Inserted Central Catheter) is essential to maintain its patency. Flushing involves using a sterile solution to clear any blockages and prevent the formation of blood clots or the accumulation of medication residue within the catheter. This helps to ensure uninterrupted fluid and medication administration.

(2) Dressing Care: Proper care of the PICC dressing is crucial to prevent infection and maintain the integrity of the insertion site. The dressing should be clean, dry, and intact at all times. It should be changed according to the healthcare provider's instructions or if it becomes soiled or loosened. Adherence to sterile technique during dressing changes is essential to minimize the risk of contamination.

(3) Securement: Ensuring securement of the PICC helps prevent accidental dislodgement or migration of the catheter. The catheter should be adequately secured using a securement device or sterile tape to keep it in place. Regular assessment of the securement device or tape is important to ensure it remains intact and effective.

(4) Monitoring for Signs of Complications: It is crucial to monitor the PICC site and the patient for any signs of complications, such as infection, phlebitis (inflammation of the vein), or infiltration (leakage of fluids into the surrounding tissues). Signs of infection may include redness, swelling, warmth, or drainage at the insertion site. Any changes in the patient's condition, such as fever or pain, should also be promptly reported to the healthcare provider.

(5) Proper Hand Hygiene: Practicing good hand hygiene is essential before and after any PICC maintenance procedures. Proper handwashing with soap and water or using an alcohol-based hand sanitizer helps prevent the transfer of microorganisms and reduces the risk of infection.

(6) Education and Communication: Patients with a PICC and their caregivers should receive appropriate education and information regarding the care and maintenance of the catheter. They should understand the importance of following instructions, recognizing signs of complications, and seeking timely medical attention when necessary. Effective communication with healthcare providers ensures any concerns or questions regarding the PICC are addressed promptly.

(7) Regular Assessment: Regular assessment of the patient's overall condition, as well as the PICC insertion site, is vital. This includes monitoring vital signs, observing for signs of infection or complications, checking the patency of the catheter, and documenting any changes or abnormalities. Routine assessments help identify issues early and facilitate appropriate interventions.

(8) Collaboration with Healthcare Team: Collaboration and communication with the healthcare team, including nurses, doctors, and other specialists, are crucial for the optimal maintenance of the PICC. This ensures that the catheter is managed appropriately, any concerns are addressed, and necessary interventions are implemented in a timely manner.

**6, Can I take a shower with a PICC line?**

(1) Yes, it is generally possible to take a shower with a PICC line. However, precautions must be taken to ensure the line remains dry and clean to minimize the risk of infection.

(2) When taking a shower with a PICC line, it is important to cover the PICC line and the insertion site with a waterproof barrier. This can be achieved by using a specialized waterproof sleeve or a plastic bag secured with waterproof tape. These barriers help to keep the PICC line dry and protect it from water exposure.

(3) It is advisable to consult with your healthcare provider or nurse before showering with a PICC line to ensure you are taking the necessary precautions and following any specific guidelines related to your condition. They can provide you with detailed instructions on how to protect your PICC line during showering.

(4) Additionally, it is important to avoid submerging the PICC line in water, such as taking baths or swimming, as this can increase the risk of infection. Showers are generally preferred over baths when you have a PICC line.

Rep2:

(1) It is generally recommended to avoid showering with a PICC line in place. A peripherally inserted central catheter (PICC line) is a thin, long tube inserted into a vein in your arm and advanced until the tip reaches a large vein near your heart. It is used to administer medications or fluids, draw blood, or monitor certain medical conditions. PICC lines are typically covered with a dressing to keep them secure and sterile.

(2) PICC lines are susceptible to contamination and infection, and exposing them to water increases the risk. Moisture from the shower can seep through the dressing, potentially leading to bacterial or fungal growth around the insertion site. Additionally, the forceful water flow may dislodge the line or cause it to become contaminated. Therefore, it is generally advised to avoid showering or getting the PICC line wet.

(3) If you need to keep the PICC line dry while bathing, you can use a plastic wrap or specialized waterproof cover designed for this purpose. These covers are available in various sizes and can be securely taped or sealed to prevent water from reaching the PICC line. It's important to follow the instructions provided by your healthcare provider or the manufacturer of the cover to ensure proper usage.

(4) Taking precautions to keep the PICC line dry during bathing helps minimize the risk of complications. If you have any concerns or specific questions regarding showering with a PICC line, it is always best to consult your healthcare provider, as they can provide personalized advice based on your individual situation. They may have specific guidelines or recommendations based on the type of PICC line you have and your overall medical condition.

**7, What problems may occur during the placement period of a PICC catheter?**

(1) Complications related to the placement of a PICC catheter can include infection, thrombosis (blood clot formation), malpositioning, and mechanical issues.

Infection: One of the primary concerns during PICC catheter placement is the risk of infection. The insertion site and the catheter itself can serve as entry points for bacteria, leading to localized or systemic infections.

Thrombosis: Another potential problem is the formation of blood clots within the catheter or the surrounding blood vessels. Thrombosis can impede proper blood flow and increase the risk of complications such as deep vein thrombosis (DVT) or pulmonary embolism.

Malpositioning: Accurate placement of the PICC catheter tip is crucial to ensure it reaches the desired location, such as the superior vena cava. Malpositioning can occur if the catheter migrates from its intended position, leading to inadequate treatment or potential damage to surrounding structures.

Mechanical issues: PICC catheters can encounter mechanical problems such as kinking or dislodgement. These issues can disrupt the flow of fluids or medications, necessitating repositioning or replacement of the catheter.

(2) Bleeding, nerve injury, and allergic reactions are additional concerns that may arise during the placement period of a PICC catheter.

Bleeding: The insertion of a PICC catheter involves puncturing a blood vessel, which carries a risk of bleeding. While minor bleeding at the insertion site is common, significant bleeding or hematoma formation may occur if a vessel is punctured improperly or if the patient has a bleeding disorder.

Nerve injury: PICC catheter placement requires careful navigation near nerves, particularly in the arm or upper chest. If a nerve is accidentally damaged or irritated during the procedure, it can result in temporary or permanent nerve dysfunction, causing sensory or motor deficits.

Allergic reactions: Some individuals may develop allergic reactions to the materials used in PICC catheters, such as the catheter itself or the adhesives used to secure it. Allergic responses can range from mild skin irritation to more severe allergic reactions, requiring immediate medical attention.

(3) Phlebitis, air embolism, and catheter-related bloodstream infections are other potential problems that may occur during the placement period of a PICC catheter.

Phlebitis: Phlebitis refers to the inflammation of a vein, which can occur as a result of the presence of the catheter. Chemical irritation from medications or solutions infused through the catheter, mechanical irritation from the catheter rubbing against the vein wall, or infection can contribute to phlebitis.

Air embolism: If air enters the bloodstream during the placement or removal of a PICC catheter, it can lead to an air embolism. Air embolisms can obstruct blood vessels, preventing proper blood flow and potentially causing serious complications, including stroke or heart attack.

Catheter-related bloodstream infections (CRBSIs): Despite efforts to maintain strict aseptic techniques during PICC catheter insertion, the risk of CRBSIs remains. Bacteria can colonize the catheter or the insertion site, leading to bloodstream infections. CRBSIs can cause localized symptoms at the insertion site or systemic manifestations, such as fever, chills, and sepsis.

(4) Thrombophlebitis, catheter migration, and catheter fracture are further issues that may occur during the placement period of a PICC catheter.

Thrombophlebitis: Thrombophlebitis is the combination of thrombosis (blood clot formation) and phlebitis (vein inflammation). It can result from the presence of the catheter, leading to pain, swelling, and redness along the vein. If left untreated, thrombophlebitis can progress to more serious complications.

Catheter migration: PICC catheters may shift or migrate from their initial placement site due to movement or patient activity. Catheter migration can lead to inadequate therapy or accidental dislodgement, requiring repositioning or possible replacement of the catheter.

Catheter fracture: PICC catheters are typically long and flexible to reach the desired location. However, they can occasionally fracture, especially if subjected to excessive tension or bending. Catheter fractures can impede proper infusion, necessitating removal and potential replacement of the catheter.

Rep2:

(1) Incorrect placement: During the placement period of a peripherally inserted central catheter (PICC), one problem that may occur is incorrect placement. This means that the catheter may not be positioned in the desired location within the central venous system. Incorrect placement can lead to complications such as catheter malposition, thrombosis, or difficulty in administering medications or fluids.

(2) Catheter-related bloodstream infections: Another problem that can arise during the PICC catheter placement period is the risk of catheter-related bloodstream infections (CRBSIs). These infections occur when bacteria or other microorganisms enter the bloodstream through the catheter. CRBSIs can lead to serious complications, including sepsis, and require prompt medical intervention.

(3) Catheter dislodgement: During the placement period, catheter dislodgement is a potential problem. This refers to the unintentional movement or removal of the catheter from its intended position. Catheter dislodgement can disrupt the delivery of medications or fluids, require repositioning or replacement of the catheter, and increase the risk of complications such as bleeding or infection.

(4) Thrombosis: Thrombosis, or the formation of blood clots, is another complication that may occur during the placement period of a PICC catheter. The presence of the catheter can cause irritation and injury to the lining of the blood vessels, increasing the risk of thrombosis. If a blood clot forms within the catheter or around its tip, it can impede blood flow, lead to occlusion, and potentially result in serious complications such as pulmonary embolism.

(5) Catheter breakage or malfunction: Occasionally, a PICC catheter may break or malfunction during the placement period. This can happen due to various reasons, including mechanical stress or accidental trauma. Catheter breakage or malfunction can hinder the proper administration of medications or fluids, necessitating the removal or replacement of the catheter.

(6) Nerve or vessel injury: During the placement process, there is a slight risk of nerve or vessel injury. Although uncommon, accidental puncture or damage to nerves or blood vessels can occur during catheter insertion. This can lead to localized pain, bleeding, hematoma formation, or neurovascular complications that require immediate attention.

(7) Allergic reactions: In rare cases, a patient may experience an allergic reaction to the materials used in the PICC catheter, such as the catheter material itself, the adhesive, or the antiseptic solution used during insertion. Allergic reactions can range from mild skin irritation to more severe symptoms, including rash, swelling, or respiratory distress. Prompt identification and management of allergic reactions are essential.

(8) Patient discomfort: Lastly, some patients may experience discomfort or pain during the PICC catheter placement period. The procedure involves the insertion of a catheter into a vein, which can cause discomfort, soreness, or a sensation of pressure. Healthcare professionals aim to minimize patient discomfort by using appropriate techniques and providing adequate pain management.

**8, What should be done when there is swelling in the limb with a PICC line and ultrasound examination indicates venous thrombosis?**

(1). Administer Anticoagulant Medication: When there is swelling in a limb with a Peripherally Inserted Central Catheter (PICC) line and ultrasound examination confirms venous thrombosis, one of the primary steps is to initiate anticoagulant medication. Anticoagulants, such as heparin or warfarin, help prevent the formation of blood clots and reduce the risk of clot propagation or extension. These medications work by inhibiting the clotting factors in the blood, thereby preventing the existing clot from growing further.

(2). Consult with a Healthcare Provider: It is crucial to involve a healthcare provider experienced in managing venous thrombosis and PICC line-related complications. They can assess the severity of the thrombosis, evaluate the overall condition of the patient, and recommend an appropriate treatment plan. Depending on the patient's medical history and the extent of the clot, the healthcare provider may consider factors like the need for hospitalization, duration of anticoagulant therapy, or the possibility of using other interventions.

(3). Implement Limb Elevation and Compression: Limb elevation and compression techniques can be utilized to help alleviate swelling and improve blood flow in the affected limb. Elevating the limb above the heart level can assist in reducing edema and relieving discomfort. Additionally, using compression stockings or bandages can aid in applying gentle pressure on the limb, promoting venous blood flow, and preventing the accumulation of fluid.

(4). Monitor and Manage Pain: Venous thrombosis can cause pain and discomfort in the affected limb. It is essential to monitor and manage the pain effectively. This may involve the use of pain medication, such as nonsteroidal anti-inflammatory drugs (NSAIDs) or analgesics, as prescribed by a healthcare professional. Adequate pain management not only enhances the patient's comfort but also encourages mobility and reduces the risk of complications.

(5). Follow-Up with Regular Ultrasound Scans: Regular ultrasound scans should be conducted to monitor the progress of the thrombosis and evaluate the effectiveness of the treatment. These scans can help determine if the clot is resolving or if further intervention is required. It is important to maintain open communication with the healthcare provider and attend scheduled follow-up appointments to ensure appropriate management of the venous thrombosis.

Rep2:

(1). Administering Anticoagulant Medication: When there is swelling in the limb with a PICC line and ultrasound examination confirms venous thrombosis, one of the primary courses of action is to administer anticoagulant medication. Anticoagulants help prevent blood clots from forming or growing larger, reducing the risk of further complications. The specific choice of anticoagulant and dosage may vary depending on the patient’s medical history, the severity of the thrombosis, and other individual factors. It is crucial to consult with a healthcare professional to determine the appropriate anticoagulant therapy.

(2). Removing the PICC Line: Another step that may be necessary is the removal of the PICC line. The presence of the line itself can contribute to the development of blood clots, and removing it reduces the risk of complications. The decision to remove the PICC line will depend on several factors, including the patient’s overall condition, the severity of the thrombosis, and the healthcare provider’s assessment.

(3). Implementing Compression Therapy: Compression therapy is often recommended for managing venous thrombosis and associated swelling. This technique involves using compression stockings or bandages to apply gentle pressure on the affected limb. Compression helps improve blood flow, reducing the risk of clot formation and minimizing swelling. The use of compression therapy should be guided by a healthcare professional who can provide instructions on proper techniques and the duration of usage.

(4). Monitoring and Follow-up: After initiating treatment and taking necessary actions, it is essential to closely monitor the patient’s progress and schedule regular follow-up appointments. Monitoring may involve repeated ultrasound examinations to assess the status of the thrombosis and evaluate the effectiveness of the treatment. Additionally, healthcare providers may check for any signs of complications or adverse effects from the anticoagulant therapy. Regular follow-up appointments enable adjustments to the treatment plan as needed and ensure the patient’s well-being.

**9, Does it hurt when inserting an IV port for infusion?**

(1) The insertion of an IV port for infusion can cause discomfort or a mild sensation similar to a quick pinch or prick. This is because a small needle is used to puncture the skin and access a vein. However, the pain experienced can vary from person to person, and factors such as the individual's pain tolerance, the skill of the healthcare professional inserting the IV, and the location of the insertion site can also influence the level of discomfort.

(2) While the insertion of an IV port may cause some initial discomfort, the pain is typically brief and subsides quickly once the needle is in place. Healthcare professionals are trained to minimize any discomfort by using techniques to make the process as smooth as possible. They may apply a numbing cream or use a smaller gauge needle to reduce pain during insertion. Additionally, choosing an appropriate insertion site can also help minimize discomfort.

(3) Some individuals may experience more discomfort during the insertion of an IV port due to factors such as anxiety or fear of needles. If a person is particularly sensitive or anxious about the procedure, it is essential to communicate their concerns to the healthcare professional. They can offer additional support, reassurance, or even options for relaxation techniques to help alleviate any discomfort or anxiety.

(4) The experience of pain or discomfort during the insertion of an IV port can vary among individuals. Some people may report minimal discomfort or no pain at all, while others may experience a moderate level of discomfort. It is important to remember that the procedure is performed by trained professionals who strive to make it as comfortable as possible. If you have specific concerns about pain during the procedure, it is best to discuss them with the healthcare provider who will be performing the insertion. They can provide you with more personalized information and address any additional questions or concerns you may have.

Rep2:

(1) Inserting an IV port for infusion can cause discomfort, but it is typically a brief and tolerable sensation. The healthcare professional will first clean the area with an antiseptic and then use a needle to puncture the skin and access a vein. This initial needle insertion may feel like a quick pinch or sting. However, once the needle is removed, a flexible catheter is usually left in place to allow for the administration of fluids or medications. The catheter itself is thin and flexible, which minimizes discomfort during the infusion process.

(2) When an IV port is inserted, there may be some localized pain or soreness around the insertion site. This can occur due to the needle puncture and the presence of the catheter within the vein. However, the level of pain experienced varies among individuals. Some people may hardly feel any pain, while others may find it more uncomfortable. The healthcare professional will strive to make the procedure as comfortable as possible by using techniques to minimize discomfort and selecting an appropriate insertion site.

(3) The sensation of pain during IV port insertion can also be influenced by individual factors such as pain tolerance, anxiety levels, and previous experiences. Some people may experience minimal pain or discomfort, while others may find it more unpleasant. It is important to communicate any concerns or discomfort to the healthcare professional performing the procedure, as they can provide additional measures to alleviate pain, such as using numbing creams or adjusting the technique.

(4) In summary, inserting an IV port for infusion can cause some discomfort, typically in the form of a quick pinch or sting during needle insertion. After the needle is removed, there may be some localized pain or soreness around the insertion site. However, the level of pain experienced can vary among individuals, and healthcare professionals aim to minimize discomfort during the procedure. Communication with the healthcare provider is crucial to address any concerns and provide additional measures to alleviate pain if needed.

**10, How long can an infusion port be used for?**

(1) Infusion ports, also known as central venous access devices (CVADs), are typically designed to remain in place for an extended period. The duration for which an infusion port can be used depends on various factors, including the type of port, the patient's condition, and the purpose of the infusion. In general, infusion ports can be used for several weeks to several months or even longer.

(2) The lifespan of an infusion port is influenced by the material it is made of, such as silicone or polyurethane. Silicone ports are known for their durability and can often remain in place for several months without complications. Polyurethane ports, on the other hand, may require replacement more frequently, usually after a few weeks to a few months.

(3) The duration of use for an infusion port also depends on the patient's medical condition and treatment requirements. Some patients may require long-term intravenous therapy, such as chemotherapy or long-term antibiotic administration. In such cases, the infusion port can be used for an extended period, typically until the treatment is completed or no longer necessary.

(4) It is important to note that infusion ports require regular maintenance and monitoring to ensure their proper functioning and minimize the risk of complications, such as infection or blockage. Healthcare professionals closely monitor the condition of the port, including regular flushing and dressing changes. If any issues or complications arise, the port may need to be replaced sooner than expected.

(5) Ultimately, the duration for which an infusion port can be used varies depending on multiple factors. It is crucial to consult with healthcare professionals who can assess the specific circumstances and provide appropriate recommendations regarding the duration of use for an infusion port in individual cases.

Rep2:

(1) Infusion ports, also known as central venous catheters or ports, can generally be used for an extended period of time, ranging from several weeks to several months or even longer. The duration of use depends on various factors such as the patient's medical condition, the type of port used, and the specific treatment requirements.

(2) The lifespan of an infusion port is typically determined by the manufacturer's guidelines and medical recommendations. Some ports are designed for short-term use, such as those used during chemotherapy cycles, which may be accessed multiple times over a few weeks. Other ports are designed for long-term use, such as those utilized for prolonged intravenous therapies, and can remain in place for months or years.

(3) The duration of port use also depends on the patient's response to treatment. If a patient requires ongoing or frequent intravenous therapies, the port may be used for a longer period. However, if the treatment course is completed or the patient's condition improves, the port may be removed earlier.

(4) It is important to regularly assess the port site for any signs of infection, thrombosis (blood clot formation), or malfunction. Healthcare providers monitor the port and may recommend replacing it if complications arise. Ultimately, the decision to remove or replace an infusion port is based on the individual patient's needs and the healthcare team's assessment of the port's condition.

(5) In summary, the duration for which an infusion port can be used varies depending on factors such as the patient's medical condition, the type of port, and the treatment requirements. While some ports are intended for short-term use, others can remain in place for months or years. Regular monitoring and evaluation by healthcare providers help determine the appropriate duration of port usage for each patient.

**11, How to care for the incision after the implantation of an infusion port?**

(1) After the implantation of an infusion port, caring for the incision is crucial to prevent infection and promote healing. Here are some general guidelines to follow:

Keep the incision site clean and dry: Clean the area around the incision gently with mild soap and water, then pat it dry with a clean towel. Avoid scrubbing the area or using harsh cleansers.

Follow dressing instructions: Your healthcare provider may provide specific instructions on dressing changes. It's important to follow these instructions carefully. If a dressing is applied, make sure it stays clean and dry. If the dressing becomes wet or soiled, contact your healthcare provider for guidance.

Avoid submerging the incision: Refrain from swimming or bathing in a bathtub until your healthcare provider gives you the green light. Submerging the incision site in water can increase the risk of infection.

(2) Monitor for signs of infection: It's essential to watch for any signs of infection at the incision site. These can include increased redness, swelling, warmth, or drainage. If you notice any of these symptoms, contact your healthcare provider immediately. They may want to examine the incision and prescribe antibiotics if necessary.

Be cautious with physical activity: While it's important to stay active, be mindful of activities that may strain or irritate the incision area. Avoid heavy lifting or strenuous exercises until your healthcare provider advises it is safe.

Report any concerns or complications: If you experience excessive pain, bleeding, or have concerns about the healing process, don't hesitate to reach out to your healthcare provider. They are the best resource to address any complications or questions you may have.

(3) Practice good hygiene: Maintaining proper hygiene is crucial during the healing process. Here are some general tips:

Wash your hands thoroughly before touching the incision site or changing dressings.

Avoid scratching or picking at the incision.

Use a clean towel or paper towel to dry the area around the incision after cleaning.

Wear loose-fitting clothing to prevent friction and irritation on the incision site.

(4) Follow your healthcare provider's instructions: Your healthcare provider will provide specific guidelines based on your individual case. It's important to adhere to their instructions regarding caring for the incision, changing dressings, and attending follow-up appointments. They may have additional recommendations based on your specific situation, so always consult with them for the best care plan.

Rep2:

(1) After the implantation of an infusion port, it is important to care for the incision site to promote healing and prevent infection. Here are some general guidelines for caring for the incision:

Keep the incision clean and dry: Clean the area around the incision gently with mild soap and water, and pat it dry with a clean towel. Avoid scrubbing the incision or using harsh chemicals.

Follow dressing instructions: Your healthcare provider will provide specific instructions on how to dress the incision. It may involve keeping the incision covered with a sterile dressing or using an antibiotic ointment. Follow these instructions carefully.

Avoid getting the incision wet: Until your healthcare provider advises otherwise, avoid exposing the incision to water, such as during showers or baths. Use plastic wrap or a waterproof cover to protect the incision during these activities.

Avoid strenuous activities: Limit activities that could strain or stretch the incision, as this may delay the healing process. Follow your healthcare provider's recommendations on activity restrictions.

(2) Monitor for signs of infection: It is essential to watch for any signs of infection around the incision. These signs may include increased pain, redness, swelling, warmth, or drainage of pus. If you notice any of these symptoms, contact your healthcare provider immediately.

Report any concerns: If you have any concerns or questions about the incision site, don't hesitate to reach out to your healthcare provider. They can provide guidance and address any issues that arise.

(3) Practice good hygiene: Maintaining good hygiene can help prevent infection and promote healing. Wash your hands thoroughly before touching the incision area or changing dressings. Avoid touching the incision unnecessarily to minimize the risk of introducing bacteria.

Follow post-operative instructions: Your healthcare provider will provide specific post-operative instructions tailored to your situation. These may include guidelines for pain management, activity restrictions, and any necessary follow-up appointments. Adhere to these instructions carefully to ensure proper healing.

(4) Avoid smoking and alcohol: If you are a smoker or consume alcohol, it is advisable to avoid these habits during the healing process. Smoking and excessive alcohol consumption can impair the body's healing ability and increase the risk of complications.

**12, What should be noted during the use of an infusion port for needle insertion?**

(1) The position and stability of the infusion port should be carefully assessed before needle insertion. Ensure that the port is securely attached to the patient's body and that it is in the correct anatomical location for the intended infusion.

(2) Prior to needle insertion, the infusion port should be thoroughly cleaned using an appropriate antiseptic solution. This helps reduce the risk of introducing infections during the procedure.

(3) The needle used for infusion port access should be appropriately sized and compatible with the port system. It is important to select a needle length and gauge that allows for safe and effective access to the port without causing any damage.

(4) The healthcare professional performing the needle insertion should follow proper aseptic technique to minimize the risk of contamination. This includes using sterile gloves, maintaining a sterile field, and ensuring that the needle does not come into contact with any non-sterile surfaces.

(5) During needle insertion, it is crucial to apply gentle, steady pressure to advance the needle into the port. Excessive force or sudden movements should be avoided to prevent damage to the port or surrounding tissues.

(6) Once the needle is inserted into the port, the healthcare professional should verify proper placement by attempting to aspirate blood or check for blood return. This confirms that the needle is correctly positioned within the port's reservoir for infusion.

(7) After infusion is complete, the needle should be carefully removed from the port, taking care not to cause any trauma or dislodge the port. Proper disposal of the used needle should be followed according to healthcare facility guidelines and regulations.

(8) Regular monitoring of the infusion site and the port is essential to detect any signs of infection, leakage, or other complications. Prompt reporting of any abnormalities to the healthcare provider is important for timely intervention.

(9) Patient education and involvement are key to ensuring the safe use of an infusion port. Patients should receive instructions on how to care for the port site, recognize signs of complications, and report any concerns to their healthcare team.

Rep2:

(1) The position of the infusion port: When using an infusion port for needle insertion, it is important to note the exact location of the port on the patient's body. The port is typically implanted just beneath the skin, and its placement can vary depending on the individual's medical condition. Knowing the precise location of the port will ensure accurate needle insertion and minimize the risk of complications.

(2) Proper hygiene and aseptic technique: Maintaining strict hygiene and following aseptic technique is crucial when using an infusion port. Cleanse the area around the port with an appropriate antiseptic solution and allow it to dry completely before inserting the needle. It is essential to minimize the risk of introducing infections or contaminants during the procedure.

(3) Needle selection and insertion angle: Choosing the correct needle gauge and length is important when accessing an infusion port. The appropriate needle size will depend on the port's specifications and the patient's individual needs. The angle of needle insertion is usually around 30 degrees, but it may vary based on the specific port design. Following the manufacturer's guidelines and healthcare provider's instructions will help ensure proper needle selection and insertion technique.

(4) Flushing and blood return: Before infusing medication or fluids, it is crucial to ensure proper flushing and blood return from the infusion port. Use a sterile saline solution to flush the port, ensuring that it flows freely and without resistance. Aspiration of blood from the port after flushing helps confirm proper needle placement within the port and ensures that the medication or fluids are delivered to the intended location.

(5) Monitoring for complications: During and after needle insertion, it is important to closely monitor the patient for any signs of complications. This includes observing for bleeding, infection, or other adverse reactions. Promptly report any unusual symptoms or concerns to a healthcare professional for further evaluation and management.

**13, Can one take a shower with a portable infusion port?**

(1) Yes, it is generally possible to take a shower with a portable infusion port. Infusion ports are medical devices implanted under the skin to provide access to veins for medication administration or blood sampling. These ports are typically designed to be waterproof and allow patients to perform their daily activities, including showering and swimming, without significant restrictions.

(2) However, it is crucial to follow specific guidelines and precautions while showering with a portable infusion port. The specific instructions may vary depending on the type of infusion port and the recommendations provided by the healthcare provider. Generally, it is important to cover the port site securely to prevent water from directly contacting the area during the shower.

(3) One common approach to protect the infusion port during a shower is to use a specialized waterproof covering or a protective sleeve. These coverings are designed to create a barrier between the port and water, ensuring that it remains dry and unaffected during the showering process. It is essential to carefully follow the instructions provided with these coverings to ensure proper usage.

(4) Additionally, it is advisable to use lukewarm water instead of hot water while showering with a portable infusion port. Hot water can increase blood flow and potentially cause discomfort or irritation around the port site. It is important to avoid scrubbing or rubbing the port area vigorously to prevent dislodgment or damage to the device.

(5) Patients should consult with their healthcare provider or the medical team managing their infusion port for specific recommendations and instructions tailored to their individual situation. They can provide detailed guidelines on how to safely shower with a portable infusion port, taking into account any unique factors or considerations. It is crucial to prioritize proper care and protection of the port to prevent infections or complications.

Rep2:

(1) Yes, it is generally possible to take a shower with a portable infusion port. Portable infusion ports, also known as implanted ports or port-a-caths, are medical devices that are surgically implanted under the skin to provide access to veins for medication administration or blood withdrawal. These ports are designed to be durable and can withstand normal activities, including showering.

(2) However, it is essential to follow certain precautions while showering with a portable infusion port. One common recommendation is to cover the port site with a waterproof dressing or a specialized port cover to protect it from direct water exposure. This helps prevent water from entering the port and potentially causing infection or other complications.

(3) Additionally, it is important to avoid scrubbing or rubbing the port area vigorously during showering, as this may irritate the skin or dislodge the port. Gentle cleansing with mild soap and water is usually sufficient.

(4) It is advisable to consult with a healthcare professional or follow the specific guidelines provided by the medical team managing the portable infusion port. They can provide personalized instructions based on the individual's situation and ensure that proper care is taken while showering to maintain the integrity of the port and minimize the risk of complications.

**14, What should be noted when carrying a portable infusion port after discharge?**

(1) It is important to note the following when carrying a portable infusion port after discharge:

Ensure proper handling: Handle the portable infusion port with care to prevent any damage or dislodgment. Avoid excessive pressure or rough handling that may compromise its functionality.

Keep it clean: Maintain cleanliness by regularly cleaning the infusion port and the surrounding area according to the healthcare provider's instructions. This helps reduce the risk of infection and ensures proper functioning.

Protect from moisture: Prevent the portable infusion port from coming into contact with water or excessive moisture. Moisture can lead to bacterial growth or damage the port's components. Cover the port with a waterproof dressing or use protective covers if necessary.

(2) Carry necessary supplies: Carry the essential supplies needed for the care and maintenance of the portable infusion port. This may include sterile cleaning solutions, dressings, adhesive tapes, and any other items recommended by the healthcare provider. Having these supplies readily available ensures you can address any needs or emergencies that may arise.

Plan for emergencies: Be prepared for unexpected situations. Keep contact information for your healthcare provider or the infusion port manufacturer readily available. In case of complications or questions, you can reach out for assistance or guidance.

Follow instructions: Adhere to the instructions provided by your healthcare provider regarding the use and care of the portable infusion port. These instructions may include guidelines for flushing, changing dressings, monitoring for signs of infection, and any specific precautions you need to take.

(3) Avoid undue stress or strain: Minimize physical activities or movements that may put excessive stress on the infusion port. This can help prevent accidental dislodgment or damage. Follow any activity restrictions or recommendations given by your healthcare provider to ensure the longevity and proper functioning of the port.

Regular monitoring: Keep track of the port's condition and functioning. Check for any signs of infection, such as redness, swelling, or discharge, around the insertion site. Report any abnormalities or concerns to your healthcare provider promptly. Regular monitoring allows for early detection of any issues and timely intervention.

Educate caregivers: If you have caregivers or family members assisting you, make sure they are properly educated on the care and handling of the portable infusion port. This will enable them to provide the necessary support and assistance while ensuring the port's safety.

Rep2:

(1) It is important to ensure that the portable infusion port is securely fastened and protected during transportation. This can be done by using a proper carrying case or bag specifically designed for this purpose. The port should be kept in an upright position to prevent any leakage or damage.

(2) The patient should be aware of the specific instructions provided by their healthcare provider regarding the handling and care of the portable infusion port. This may include guidelines on cleaning, flushing, and changing the port, if necessary. It is crucial to follow these instructions diligently to maintain the functionality and sterility of the port.

(3) Regular monitoring of the portable infusion port is essential after discharge. This involves checking for any signs of infection, such as redness, swelling, or discharge at the insertion site. If any abnormalities are noticed, it is important to contact the healthcare provider immediately for further guidance.

(4) The patient should also be cautious while engaging in activities that may put excessive strain or pressure on the infusion port. It is advisable to avoid activities that involve heavy lifting, strenuous exercise, or direct impact to the port area. Protecting the port from accidental bumps or trauma can help prevent complications and maintain its integrity.

(5) Keeping the portable infusion port clean and dry is crucial. The patient should avoid exposing the port to excessive moisture or submerging it in water. If the port does come into contact with water or any other liquid, it should be thoroughly dried using a clean cloth. Moisture can increase the risk of infection or affect the functioning of the port.

(6) Regular communication with the healthcare provider is vital when carrying a portable infusion port. The patient should keep the healthcare team informed about any changes in their condition or any concerns related to the port. This allows for timely evaluation and necessary adjustments to the treatment plan, if required.

(7) Finally, it is important for the patient to educate their close contacts, such as family members or caregivers, about the presence of the portable infusion port and its importance. They should be aware of the potential risks and be trained on how to respond in case of an emergency or complications related to the port.

**The Pain Control**

**1, How did humans come to understand pain and cancer?**

(1) Humans came to understand pain through their sensory perception. Pain is a complex sensory and emotional experience that occurs when special nerve endings called nociceptors detect potential or actual tissue damage. These nociceptors are found throughout the body, including the skin, organs, and internal structures. When activated by harmful stimuli such as heat, pressure, or chemicals released from injured tissues, nociceptors send electrical signals to the brain, which then interprets these signals as pain. Over time, through observation and self-reporting, humans have developed a subjective understanding of pain and its various qualities, allowing them to describe and communicate their experiences.

(2) The understanding of cancer has evolved through centuries of scientific inquiry. Ancient civilizations recognized the existence of tumors, but it was not until the modern era that the nature of cancer became better understood. In the 19th century, advancements in microscopy and histology allowed researchers to observe cancer cells under a microscope, leading to the recognition that cancer originates from abnormal cell growth and division. The discovery of DNA and the understanding of genetics in the 20th century further illuminated the mechanisms underlying cancer development, including mutations in genes that regulate cell growth and division. Today, a multidisciplinary approach combining genetics, molecular biology, pathology, and clinical research continues to deepen our understanding of cancer, its causes, and potential treatments.

(3) Pain perception and cancer research have benefited from advancements in medical imaging techniques. X-rays, developed in the late 19th century, enabled the visualization of skeletal structures and the detection of certain tumors. In the mid-20th century, the introduction of computed tomography (CT) and magnetic resonance imaging (MRI) provided more detailed and cross-sectional views of the body, aiding in the diagnosis and staging of cancer. Positron emission tomography (PET) and single-photon emission computed tomography (SPECT) scans have further contributed to the understanding of cancer by detecting metabolic and molecular activities within tissues. These imaging technologies have revolutionized the field of oncology, allowing for earlier detection, accurate tumor characterization, and monitoring of treatment response.

(4) The development of sophisticated laboratory techniques has played a crucial role in unraveling the mechanisms of pain and cancer. Researchers have employed various molecular and cellular approaches to investigate the underlying processes involved. For pain, studies have focused on identifying the molecules involved in nociceptor activation and transmission of pain signals. Techniques like patch-clamping, which allows the measurement of electrical currents across individual cells, have provided insights into the function of ion channels involved in pain sensation. In the case of cancer, scientists have utilized techniques such as gene expression profiling, next-generation sequencing, and proteomics to analyze the genetic and molecular alterations that drive cancer initiation, progression, and metastasis. These techniques have revealed key signaling pathways, genetic mutations, and potential therapeutic targets, advancing our understanding of cancer biology.

(5) Clinical studies and patient data have also been instrumental in understanding pain and cancer. Observational studies, clinical trials, and patient registries have provided valuable information about the prevalence, risk factors, and outcomes associated with pain and cancer. Large-scale epidemiological studies have identified patterns and associations that help researchers understand the incidence and impact of these conditions. Moreover, patient-reported outcomes and experiences have played a significant role in shaping our understanding of pain and cancer, providing subjective insights that complement objective measurements and informing the development of patient-centered care approaches.

(6) Collaborative efforts among researchers, healthcare professionals, and patient advocacy groups have further contributed to the understanding of pain and cancer. Interdisciplinary collaborations bring together experts from various fields, such as neuroscience, oncology, pharmacology, psychology, and sociology, to tackle complex questions and share knowledge. Patient advocacy groups play a vital role in raising awareness, supporting research, and advocating

Rep2:

(1) Humans came to understand pain through their innate sensory system. Pain is a complex sensation that serves as a crucial warning mechanism to protect the body from potential harm. When a harmful stimulus, such as heat or injury, activates specialized pain receptors called nociceptors, electrical signals are generated and transmitted through the nervous system to the brain. The brain processes these signals and produces the subjective experience of pain. Over time, through empirical observation and experimentation, humans have developed a better understanding of the physiological and psychological aspects of pain, enabling them to develop effective strategies for managing and treating it.

(2) Understanding cancer has been a result of extensive scientific research and medical advancements. Cancer is a group of diseases characterized by the uncontrolled growth and spread of abnormal cells. The understanding of cancer began with observations and descriptions of tumors dating back centuries. However, it was not until the advent of modern scientific techniques, such as microscopy and genetics, that researchers gained deeper insights into the mechanisms underlying cancer development. Discoveries in genetics, molecular biology, and oncology have revealed that cancer arises from genetic mutations that disrupt the normal regulation of cell growth and division. Further studies have elucidated the role of environmental factors, lifestyle choices, and hereditary predispositions in cancer development. These findings have contributed to the development of diagnostic tools, treatment modalities, and preventive measures to combat cancer.

(3) Pain and cancer research have been extensively explored through clinical and experimental studies. Pain research involves investigations into the mechanisms of pain perception, the classification of different types of pain (such as acute, chronic, or neuropathic pain), and the development of effective pain management strategies. Scientists have studied pain at the cellular, molecular, and neurophysiological levels, uncovering the roles of various neurotransmitters, receptors, and pathways involved in pain signaling. This knowledge has led to the development of pain-relieving medications, such as analgesics and anesthetics, as well as non-pharmacological interventions like physical therapy, cognitive-behavioral therapy, and complementary approaches.

Cancer research, on the other hand, encompasses a wide range of disciplines, including cell biology, genetics, biochemistry, immunology, and epidemiology. Researchers have investigated the molecular mechanisms driving cancer initiation, progression, and metastasis. They have identified key oncogenes and tumor suppressor genes, elucidated the dysregulation of cellular processes like cell cycle control and apoptosis, and explored the interaction between cancer cells and the immune system. Additionally, studies have focused on understanding the risk factors associated with cancer, such as tobacco use, exposure to carcinogens, and genetic predispositions. This multidisciplinary research has paved the way for innovative cancer treatments, such as targeted therapies, immunotherapies, and precision medicine approaches.

(4) The understanding of pain and cancer is an ongoing endeavor fueled by continuous scientific inquiry and technological advancements. Researchers worldwide collaborate to unravel the complexities of these phenomena, seeking novel insights and approaches to improve patient care and outcomes. Through interdisciplinary efforts, including basic research, clinical trials, and translational studies, scientists aim to develop better diagnostic tools, more effective treatments, and preventive strategies to mitigate the impact of pain and cancer on individuals and society as a whole.

**2, Are there any guidelines for standardized diagnosis and treatment of cancer pain?**

(1) Standardized guidelines for the diagnosis and treatment of cancer pain are essential to ensure consistent and effective care for patients. These guidelines typically encompass a comprehensive approach that addresses the assessment, management, and follow-up of cancer-related pain. They aim to provide healthcare professionals with evidence-based recommendations and best practices to improve the quality of life for individuals experiencing cancer pain.

(2) Diagnosis guidelines for cancer pain often emphasize the importance of a thorough assessment, which includes evaluating the location, intensity, and characteristics of the pain. Additionally, healthcare providers assess the impact of pain on the patient's daily functioning, emotional well-being, and overall quality of life. The guidelines may recommend using standardized pain assessment tools to facilitate accurate and consistent evaluation.

(3) Treatment guidelines for cancer pain typically advocate for a multimodal approach, combining various strategies to effectively manage pain. Pharmacological interventions, such as opioids, nonsteroidal anti-inflammatory drugs (NSAIDs), and adjuvant medications, may be recommended based on the severity and type of pain. Non-pharmacological approaches like physical therapy, psychological interventions, and complementary therapies may also be incorporated into the treatment plan. The guidelines often emphasize the importance of individualizing treatment based on patient preferences, underlying disease factors, and potential adverse effects.

(4) Follow-up guidelines for cancer pain focus on regular assessment and monitoring to evaluate the effectiveness of the pain management plan. These guidelines may recommend scheduled visits to assess pain intensity, adjust medication dosages if necessary, and address any emerging concerns or side effects. Furthermore, the guidelines may stress the importance of interdisciplinary collaboration and communication among healthcare professionals to ensure coordinated care and continuity for patients with cancer pain.

Rep2:

(1) Yes, there are guidelines available for standardized diagnosis and treatment of cancer pain. These guidelines aim to provide healthcare professionals with evidence-based recommendations and best practices for managing pain in cancer patients. They help ensure consistent and effective care for individuals experiencing pain due to cancer or its treatments.

(2) The guidelines for standardized diagnosis of cancer pain generally involve a comprehensive assessment of the patient's pain. This assessment includes evaluating the intensity, location, quality, and impact of the pain on the patient's daily activities and overall well-being. It also considers the underlying cause of the pain, such as tumor growth, nerve compression, or treatment-related side effects.

(3) Treatment guidelines for cancer pain typically encompass a multimodal approach that combines pharmacological and non-pharmacological interventions. Pharmacological interventions may include the use of opioids, non-opioid analgesics, adjuvant medications (e.g., antidepressants, anticonvulsants), and other medications to manage specific pain symptoms. Non-pharmacological interventions may involve physical therapy, psychological support, complementary therapies, and interventional procedures like nerve blocks or radiotherapy.

(4) Additionally, the guidelines emphasize the importance of regular reassessment and adjustment of the pain management plan based on the patient's response and changing needs. They promote the concept of personalized care, taking into account individual variations in pain perception, comorbidities, and treatment goals. Collaborative decision-making between healthcare professionals and patients is also encouraged to ensure shared understanding and optimize pain control while minimizing potential adverse effects.

(5) These guidelines are developed and updated by expert panels or organizations specializing in pain management and oncology, such as the American Society of Clinical Oncology (ASCO) and the European Society for Medical Oncology (ESMO). They rely on a systematic review and synthesis of available research evidence, along with expert consensus, to provide reliable recommendations that healthcare providers can use to guide their clinical practice.

(6) It's important to note that guidelines may vary slightly across different regions or healthcare settings, and individual patient circumstances may require tailored approaches. Therefore, healthcare professionals should consider the specific context and preferences of their patients while applying the general principles outlined in the guidelines. Regular updates and ongoing education can help ensure that healthcare providers are aware of the latest evidence and best practices in cancer pain management.

**3, Why do colorectal cancer patients experience pain?**

(1) Colorectal cancer patients may experience pain due to the tumor itself. As the cancer grows, it can invade and irritate nearby tissues and organs, leading to discomfort and pain. The tumor can put pressure on nerves, causing nerve pain. Additionally, the tumor may obstruct the normal passage of stool, leading to bowel distension and cramping pain.

(2) Another reason for pain in colorectal cancer patients is the development of metastases. Cancer cells can spread from the primary tumor to other parts of the body, such as the liver, lungs, or bones. When these metastatic lesions grow and invade surrounding tissues, they can cause pain. The spread of cancer to bones, in particular, can be quite painful and may require specific interventions for pain management.

(3) Surgery is a common treatment for colorectal cancer, and postoperative pain is another potential source of discomfort. The incision made during surgery can cause pain during the healing process. Additionally, after surgery, patients may experience abdominal pain or cramping due to the manipulation of the intestines and other tissues during the procedure.

(4) In some cases, colorectal cancer patients may undergo chemotherapy or radiation therapy as part of their treatment. These treatments can cause side effects that contribute to pain. Chemotherapy drugs may cause peripheral neuropathy, a condition characterized by nerve damage and pain in the hands and feet. Radiation therapy, particularly when directed at the pelvis, can result in radiation-induced enteritis or proctitis, which can cause abdominal pain or rectal discomfort, respectively.

(5) Lastly, cancer-related pain in colorectal cancer patients can be influenced by individual factors such as the person's pain tolerance, psychological factors like anxiety or depression, and overall well-being. These factors can modulate the perception and experience of pain, making it a complex and multifaceted aspect of a patient's journey with colorectal cancer.

Rep2:

(1) Colorectal cancer patients may experience pain due to the tumor itself. As the tumor grows, it can invade and put pressure on nearby tissues, organs, or nerves, causing pain. The tumor can also obstruct the bowel, leading to cramping pain and discomfort. Additionally, if the cancer spreads (metastasizes) to other parts of the body, such as the liver or bones, it can cause pain in those areas as well.

(2) Another reason for pain in colorectal cancer patients is inflammation. Cancerous tumors can trigger an inflammatory response in the body, which can cause pain and discomfort. Inflammation can affect the surrounding tissues, leading to localized pain in the abdomen or rectal area. Inflammatory processes can also contribute to bowel dysfunction and cause abdominal cramping.

(3) Pain in colorectal cancer patients may be associated with nerve involvement. The tumor can infiltrate and compress nerves in the vicinity, resulting in nerve pain (neuropathic pain). This type of pain can be sharp, shooting, or tingling and may radiate to other areas. Nerve pain can be particularly challenging to manage and may require specialized treatments or medications to alleviate the symptoms.

(4) In some cases, colorectal cancer treatments can cause pain as a side effect. Surgery, radiation therapy, and chemotherapy are common treatment approaches for colorectal cancer, and they can all have pain-related consequences. Surgical procedures can lead to postoperative pain, while radiation therapy and chemotherapy can cause various types of discomfort, such as mouth sores, gastrointestinal irritation, or nerve damage, which can manifest as pain. Pain management strategies can be employed to help alleviate these treatment-related pains and improve the patient's quality of life.

**4, Is it considered serious if one wakes up in pain every night while sleeping?**

(1) Yes, it is generally considered serious if someone wakes up in pain every night while sleeping. Consistently experiencing pain during sleep can significantly impact an individual's quality of life and overall well-being. It may indicate an underlying medical condition or injury that requires attention and proper medical evaluation.

(2) Waking up in pain every night while sleeping should not be ignored, as it could be a sign of a chronic condition or an acute injury that needs medical attention. Ignoring persistent pain during sleep can lead to further complications and potentially worsen the underlying issue. Consulting with a healthcare professional is advisable to determine the cause of the pain and explore appropriate treatment options.

(3) Consistently waking up in pain during sleep is a matter that should be taken seriously. It could be indicative of various health conditions, such as arthritis, fibromyalgia, or even an injury. Seeking medical advice is crucial to identify the root cause of the pain, receive an accurate diagnosis, and develop an appropriate treatment plan. Addressing the issue promptly can help alleviate pain, improve sleep quality, and prevent potential long-term complications.

(4) Waking up in pain every night while sleeping should not be dismissed as a minor inconvenience. It is important to recognize the potential seriousness of this situation. Chronic pain can have a profound impact on physical and mental well-being, leading to sleep disturbances, fatigue, and decreased quality of life. Seeking medical attention and discussing the symptoms with a healthcare professional is essential for proper diagnosis and treatment.

Rep2:

(1) It is generally considered serious if someone consistently wakes up in pain every night while sleeping. Persistent pain during sleep can be indicative of an underlying medical condition or injury that requires attention. Ignoring or neglecting such pain can lead to further complications and may impact one's overall well-being and quality of life.

(2) Waking up in pain every night during sleep is a matter that should be taken seriously. Pain interrupts sleep and can prevent the body from getting the rest it needs to recover and heal. It is important to identify the source of the pain and seek appropriate medical advice to address the underlying cause.

(3) Consistently experiencing pain upon waking during sleep is a concerning symptom that should not be ignored. It could be a sign of an ongoing health issue, such as musculoskeletal problems, nerve compression, inflammation, or other conditions. Seeking medical evaluation and diagnosis is crucial to determine the cause of the pain and develop an appropriate treatment plan.

(4) Consistently waking up in pain every night while sleeping is a significant concern that should not be dismissed. Pain during sleep can disrupt the body's restorative processes and impact overall well-being. It is essential to consult with a healthcare professional who can evaluate the situation, conduct a thorough examination, and potentially order diagnostic tests to identify the underlying cause of the pain and provide appropriate treatment recommendations.

**5, Why do some colorectal cancer patients experience abdominal pain while others have bone pain?**

(1) Colorectal cancer patients may experience abdominal pain due to the primary tumor's location and its effects on the surrounding tissues. The tumor can grow and invade nearby structures, causing pressure and compression on the abdominal organs and nerves. This compression can lead to pain in the abdomen, as well as other symptoms like bloating, cramping, and a sense of fullness. The severity of the pain can vary depending on the size and extent of the tumor.

(2) On the other hand, some colorectal cancer patients may experience bone pain. This occurs when the cancer cells from the primary tumor spread (metastasize) to the bones, a process known as bone metastasis. The cancer cells invade the bone tissue, disrupting its normal structure and causing pain. Bone metastasis in colorectal cancer commonly affects the spine, hips, pelvis, and long bones of the legs. Bone pain associated with metastatic colorectal cancer can be persistent, worsen with movement, and may lead to fractures or other skeletal complications.

(3) The reason for the different types of pain experienced by colorectal cancer patients lies in the distinct biological mechanisms underlying tumor growth and spread. In the case of abdominal pain, the tumor primarily affects the local abdominal structures, such as the intestines, peritoneum, and nearby nerves. The pain arises from the direct compression or invasion of these tissues, triggering nerve signals that are interpreted as pain sensations.

(4) In contrast, bone pain in colorectal cancer occurs due to the spread of cancer cells to the bones. This process involves cancer cells detaching from the primary tumor, entering the bloodstream or lymphatic system, and eventually reaching the bones. Once there, the cancer cells disrupt normal bone tissue and cause inflammation, leading to bone pain. The bones have a rich supply of sensory nerves, and the presence of cancer cells triggers pain signals that are transmitted to the brain, resulting in bone pain for the patient.

Rep2:

(1) Colorectal cancer patients may experience abdominal pain due to the primary tumor's location within the colon or rectum. The tumor can obstruct or narrow the intestinal passage, causing discomfort, cramping, or sharp pains in the abdomen. Additionally, the tumor's growth may cause inflammation and irritation of the surrounding tissues, contributing to abdominal pain.

(2) On the other hand, bone pain in colorectal cancer patients typically occurs when the cancer metastasizes or spreads to the bones. Cancer cells can invade the bone tissue, leading to bone destruction and the release of substances that stimulate pain receptors. This bone involvement can result in persistent or intermittent pain, often localized to the affected bone or bones.

(3) The differing locations of pain in colorectal cancer patients are primarily determined by the disease progression and metastatic spread. Abdominal pain commonly arises when the tumor is primarily located in the colon or rectum, affecting the gastrointestinal tract. In contrast, bone pain is a result of metastasis, where cancer cells travel through the bloodstream or lymphatic system and establish secondary tumors in the bones.

(4) The development of abdominal pain or bone pain in colorectal cancer patients can also depend on individual factors such as the tumor's growth rate, the extent of metastasis, and the patient's overall health. Factors like the size of the primary tumor, its proximity to nerves, and the aggressiveness of the cancer cells can influence the likelihood and severity of pain experienced by the patient. Additionally, each person's pain perception and tolerance can vary, leading to differences in the manifestation and intensity of symptoms.

**6, What are some good methods for pain relief?**

(1) Over-the-counter pain relievers: Non-prescription medications such as acetaminophen (Tylenol) and nonsteroidal anti-inflammatory drugs (NSAIDs) like ibuprofen (Advil, Motrin) can be effective for mild to moderate pain relief. These medications work by reducing inflammation and blocking pain signals in the body.

(2) Topical analgesics: These are pain-relieving creams, gels, or patches that are applied directly to the skin over the painful area. They often contain ingredients like menthol, capsaicin, or lidocaine, which can help alleviate pain and provide localized relief.

(3) Heat and cold therapy: Applying heat or cold to the affected area can be helpful in relieving pain. Heat therapy, such as using heating pads or warm baths, helps increase blood flow and relax muscles, while cold therapy, such as ice packs or cold compresses, can reduce inflammation and numb the area.

(4) Physical therapy and exercise: In some cases, certain exercises and physical therapy techniques can help alleviate pain. These therapies aim to strengthen muscles, improve flexibility, and correct any imbalances or issues contributing to the pain. They can be particularly beneficial for chronic pain conditions.

(5) Relaxation techniques and stress management: Techniques like deep breathing, meditation, and guided imagery can help reduce pain by promoting relaxation and reducing stress. Stress and anxiety can intensify pain perception, so learning to manage them effectively can provide relief.

(6) Massage therapy: Professional massage or self-massage techniques can help relieve muscle tension and promote relaxation, which can reduce pain. Massage can also stimulate the release of endorphins, the body's natural painkillers.

(7) Transcutaneous electrical nerve stimulation (TENS): TENS therapy involves using a small battery-operated device to deliver low-voltage electrical currents to the skin. These electrical impulses can disrupt pain signals, providing temporary relief for some individuals.

(8) Mind-body therapies: Practices like acupuncture, acupressure, and chiropractic care may offer pain relief for certain individuals. These therapies focus on balancing the body's energy and addressing musculoskeletal imbalances that can contribute to pain.

(9) Prescription medications: For severe or chronic pain, prescription medications may be necessary. These can include opioids, muscle relaxants, anticonvulsants, or antidepressants. However, it's important to use these medications under the guidance of a healthcare professional due to their potential side effects and addictive nature.

(10) Complementary and alternative approaches: Various complementary therapies like herbal remedies, essential oils, and nutritional supplements are often used for pain relief. While their effectiveness may vary, it's essential to consult with a healthcare professional before using them to ensure safety and suitability.

Rep2:

(1) Nonsteroidal anti-inflammatory drugs (NSAIDs) such as ibuprofen or aspirin can be effective for relieving pain. These medications work by reducing inflammation in the body, which can help alleviate pain. They are commonly used for mild to moderate pain relief and are available over the counter in many countries. However, it's important to follow the recommended dosage and consult a healthcare professional if you have any underlying health conditions or are taking other medications.

(2) Acetaminophen, also known as paracetamol, is another commonly used pain reliever. It is effective in reducing pain and fever but does not have the same anti-inflammatory effects as NSAIDs. Acetaminophen is often used for mild to moderate pain relief and is available over the counter. However, it's important to follow the recommended dosage and avoid exceeding the maximum daily limit to prevent liver damage.

(3) Topical analgesics can provide localized pain relief. These are creams, gels, or patches that can be applied directly to the skin over the painful area. They work by numbing the nerves in the skin, which can help alleviate pain. Topical analgesics are commonly used for conditions such as muscle or joint pain, sprains, or strains. However, it's important to read and follow the instructions on the product label and consult a healthcare professional if the pain persists or worsens.

(4) Physical therapy and exercise can be beneficial for managing pain, especially for musculoskeletal conditions. A qualified physical therapist can develop a personalized treatment plan that includes exercises and techniques to improve flexibility, strength, and mobility, which can help reduce pain and improve overall function. Physical therapy may also involve other modalities such as heat or cold therapy, ultrasound, or electrical stimulation, depending on the specific condition. It's important to consult a healthcare professional and work with a qualified physical therapist to determine the appropriate exercises and techniques for your specific situation.

(5) Mind-body techniques, such as relaxation exercises, meditation, deep breathing, or guided imagery, can help manage pain by promoting relaxation and reducing stress. These techniques can help distract the mind from pain sensations and promote a sense of well-being. They can be used alongside other pain management strategies and may be particularly helpful for chronic pain conditions. It's important to learn these techniques from qualified instructors or through reputable resources to ensure proper guidance and effectiveness.

(6) Prescription medications may be necessary for severe or chronic pain that is not adequately managed by over-the-counter options. These medications can include opioids, muscle relaxants, or anticonvulsants, among others. It's crucial to work closely with a healthcare professional when using prescription medications for pain relief, as they can have potential side effects and require careful monitoring.

**7, Do you only need to take medication when experiencing cancer pain, and not when you are pain-free?**

(1) No, taking medication for cancer pain is not solely dependent on whether you are currently experiencing pain or not. Managing cancer pain involves a comprehensive approach that aims to provide relief and improve the overall quality of life for individuals with cancer. It is important to adhere to a prescribed medication regimen even when pain is not present to prevent pain from occurring or escalating to unmanageable levels.

(2) Cancer pain can be complex and multifaceted, influenced by various factors such as the type and stage of cancer, individual pain tolerance, and treatment interventions. Medications used to manage cancer pain, such as opioids, nonsteroidal anti-inflammatory drugs (NSAIDs), and adjuvant analgesics, are often prescribed on a regular basis to maintain a consistent level of pain control. Waiting until pain arises may lead to unnecessary suffering and difficulty in achieving adequate pain relief.

(3) Pain management in cancer care focuses on maintaining a baseline level of pain control and preventing pain breakthroughs. By adhering to a prescribed medication schedule, individuals can proactively manage their pain and prevent it from reaching severe levels. Medications may be adjusted based on the individual's pain levels, response to treatment, and side effects, but a proactive approach to pain management is generally more effective than reactive measures.

(4) It is crucial to communicate regularly with healthcare professionals to assess pain levels, adjust medication doses, and explore additional pain management strategies. Non-pharmacological approaches, such as physical therapy, relaxation techniques, and complementary therapies, can also be incorporated alongside medications to enhance pain management. The goal is to achieve optimal pain control and maintain a good quality of life throughout the cancer journey, irrespective of whether pain is currently experienced or not.

Rep2:

(1) No, medication for cancer pain is not only needed when experiencing pain. Cancer pain management typically involves a multimodal approach that includes both proactive and reactive strategies. Proactive pain management aims to prevent pain from occurring or escalating by administering medications regularly, even when pain is absent. This approach helps maintain pain control and improve the overall quality of life for individuals with cancer. By taking medication as prescribed, patients can achieve better pain management and reduce the likelihood of pain becoming severe or unmanageable.

(2) It is not advisable to rely solely on pain medication when pain is present and forego medication when pain-free. Cancer pain can vary in intensity and frequency, and waiting until pain occurs to start taking medication may lead to delays in achieving adequate pain relief. Regularly taking medication as prescribed helps maintain a consistent level of pain control, which can minimize the impact of pain on daily activities and enhance overall well-being. Additionally, certain pain medications may require time to reach optimal levels in the body, so adhering to a regular dosing schedule is crucial for their effectiveness.

(3) Taking medication only when experiencing cancer pain may not provide sufficient relief or adequately address the underlying causes of pain. Cancer pain can be complex and multifactorial, resulting from factors such as tumor growth, nerve involvement, inflammation, or treatment-related side effects. Some medications, such as opioids or adjuvant analgesics, require regular administration to achieve optimal pain control and manage the underlying mechanisms contributing to pain. By following a prescribed medication regimen, individuals can proactively manage pain, prevent its escalation, and potentially minimize the need for higher doses or more potent medications in the future.

(4) Neglecting medication when pain-free may result in a suboptimal pain management approach for individuals with cancer. Pain can have a significant impact on physical, emotional, and social well-being, even when it is not actively experienced. By adhering to a regular medication schedule, individuals can maintain a stable level of pain control, reduce the risk of breakthrough pain episodes, and potentially enhance their overall quality of life. Open communication with healthcare professionals is essential to ensure appropriate medication adjustments and identify any potential side effects or interactions, enabling personalized pain management strategies tailored to the individual's needs.

**8, How can I sleep when I'm in cancer pain?**

(1) It can be challenging to sleep when experiencing cancer pain, but there are several strategies you can try to improve your sleep. Firstly, ensure that you have a comfortable sleep environment by using supportive pillows and a mattress. Create a relaxing bedtime routine that includes activities like taking a warm bath, reading a book, or listening to calming music. Practice relaxation techniques such as deep breathing, progressive muscle relaxation, or guided imagery to help reduce pain and promote sleep. It's important to communicate with your healthcare team about your pain levels so that they can help adjust your pain management plan if necessary. They may suggest medications or other interventions to alleviate the pain, which can ultimately improve your sleep quality.

(2) Another approach to promoting sleep when experiencing cancer pain is to manage your pain with appropriate medications. Speak with your healthcare team about the medications you are currently taking and whether they can be adjusted to better control your pain during nighttime hours. They may recommend pain-relieving medications or adjustments to your current regimen to provide better pain management while you sleep. Remember to follow your healthcare team's instructions and guidelines for medication use to ensure safety and effectiveness.

(3) In addition to medication and relaxation techniques, consider incorporating lifestyle changes that can support better sleep. Establish a consistent sleep schedule by going to bed and waking up at the same time every day, even on weekends. Avoid consuming stimulants like caffeine and nicotine close to bedtime, as they can interfere with sleep. Create a peaceful sleep environment by keeping your bedroom cool, dark, and quiet. Use blackout curtains, earplugs, or a white noise machine to block out external distractions. It may also be helpful to try alternative therapies such as acupuncture, massage, or yoga, as they have shown potential in managing cancer pain and improving sleep quality.

(4) Engaging in regular physical activity, within the limits of your condition, can have a positive impact on sleep. Consult with your healthcare team to determine the types and amounts of exercise that are safe and appropriate for you. Engaging in gentle exercises like walking or stretching during the day can help reduce pain and promote relaxation, making it easier to fall asleep at night. However, avoid vigorous exercise close to bedtime, as it may have a stimulating effect and make it harder to sleep. Remember to listen to your body and pace yourself accordingly.

(5) Consider exploring complementary therapies that can provide pain relief and relaxation. Some cancer centers offer integrative medicine services, which may include techniques like acupuncture, massage, aromatherapy, or mindfulness meditation. These approaches can help alleviate pain, reduce stress, and improve sleep quality. Talk to your healthcare team or seek out practitioners experienced in working with cancer patients to explore these options.

(6) Finally, don't underestimate the power of emotional support and communication. Living with cancer can be emotionally challenging, and addressing any anxiety, stress, or depression can positively impact your sleep. Reach out to your loved ones, join support groups, or consider therapy or counseling to help you navigate the emotional aspects of your cancer journey. Sharing your concerns and fears can provide a sense of relief, leading to improved emotional well-being and potentially better sleep.

Rep2:

(1) Seek support from loved ones: It is important to reach out to your friends, family, and other loved ones for emotional support after being diagnosed with cancer. Share your feelings and concerns with them, and allow them to be there for you during this difficult time. Talking about your emotions can help alleviate the burden and provide a sense of comfort and understanding.

(2) Join a support group: Connecting with others who are going through or have been through similar experiences can be incredibly helpful. Consider joining a cancer support group where you can share your thoughts and feelings with people who understand what you're going through. These groups can provide a safe space to express your emotions, receive support, and gain insights from others who have faced similar challenges.

(3) Engage in self-care activities: Taking care of yourself physically, mentally, and emotionally is crucial when dealing with negative emotions. Engage in activities that bring you joy and help you relax, such as practicing meditation or mindfulness, engaging in hobbies, reading books, or listening to music. Prioritizing self-care can provide a sense of control and well-being, helping to alleviate negative emotions.

(4) Seek professional help: If you find that your negative emotions are persistent, overwhelming, or interfering with your daily life, it may be beneficial to seek professional help. A mental health professional, such as a therapist or counselor, can offer specialized support and guidance tailored to your emotional needs. They can help you develop coping strategies, provide a safe space to process your emotions, and offer valuable tools to navigate this challenging period.

(5) Educate yourself about cancer: Gaining knowledge and understanding about your diagnosis and treatment can help alleviate some of the anxiety and fear associated with cancer. Ask your healthcare team for reliable resources or reputable websites where you can learn more about your specific type of cancer, treatment options, and prognosis. Educating yourself can empower you to make informed decisions and reduce uncertainty, which may positively impact your emotional well-being.

(6) Practice stress-reducing techniques: Cancer diagnosis can lead to increased stress levels. Explore stress-reducing techniques such as deep breathing exercises, progressive muscle relaxation, or yoga. These practices can help calm your mind, reduce anxiety, and improve your overall emotional well-being.

(7) Express your emotions creatively: Artistic outlets such as writing in a journal, painting, or playing a musical instrument can provide a cathartic release for your emotions. Engaging in creative expression allows you to process and express your feelings in a nonverbal and meaningful way. It can serve as a form of therapy and help you gain clarity and emotional relief.

**9, What are the consequences if cancer pain is left untreated?**

(1) If cancer pain is left untreated, it can significantly impact a person's quality of life. Chronic and severe pain can lead to physical and emotional distress, affecting various aspects of daily functioning. Persistent pain can interfere with sleep, appetite, mobility, and overall well-being, making it difficult for individuals to engage in regular activities or enjoy their usual hobbies. Untreated pain may also contribute to increased fatigue, reduced ability to concentrate, and a diminished sense of self-worth.

(2) Neglecting to address cancer pain can result in a decline in the individual's mental and emotional well-being. The continuous experience of pain can lead to anxiety, depression, irritability, and mood swings. It can disrupt interpersonal relationships and social interactions, causing feelings of isolation and withdrawal. Unmanaged pain can also affect a person's ability to cope with the challenges of cancer treatment, leading to decreased compliance with medications and therapies.

(3) Leaving cancer pain untreated can impact the physical health and functioning of the affected individual. Severe and uncontrolled pain can weaken the immune system and hinder the body's ability to heal and recover. It may contribute to muscle wasting, decreased mobility, and reduced physical endurance. Inadequate pain management can also lead to changes in posture and movement patterns, potentially resulting in muscle imbalances and decreased overall physical fitness.

(4) Persistent untreated cancer pain can create a negative feedback loop, where pain amplification occurs over time. Uncontrolled pain can sensitize the nervous system, causing an increased perception of pain even with minor stimuli. This phenomenon, known as central sensitization, can lead to a condition called chronic pain syndrome, where the pain persists even after the cancer has been treated or eliminated. Managing cancer pain early and effectively is crucial in preventing the development of chronic pain conditions.

(5) In addition to the individual consequences, untreated cancer pain can also affect the overall management of the disease. Uncontrolled pain may lead to increased hospitalizations, emergency room visits, and healthcare costs. It can interfere with the effectiveness of cancer treatments, as severe pain can limit the ability to undergo necessary procedures or receive optimal doses of chemotherapy or radiation therapy. By addressing cancer pain promptly, healthcare professionals can improve the patient's overall treatment outcomes and enhance their ability to tolerate and respond to cancer therapies.

Rep2:

(1) Untreated cancer pain can significantly impact a person's quality of life. Pain can be both physically and emotionally debilitating, causing distress, anxiety, and depression. It can interfere with daily activities, sleep patterns, and overall functioning. The constant presence of pain can lead to social isolation and a loss of enjoyment in previously pleasurable activities. Inadequate pain management may also affect the individual's ability to comply with cancer treatments, leading to potential delays or interruptions in their care.

(2) Chronic untreated cancer pain can have physiological consequences. Persistent pain can trigger a stress response in the body, leading to increased heart rate, elevated blood pressure, and hormonal imbalances. These changes can weaken the immune system and make it harder for the body to fight off infections or recover from illness. Uncontrolled pain may also interfere with appetite and nutrition, leading to weight loss, malnutrition, and muscle wasting.

(3) Untreated cancer pain can have long-term psychological effects. The constant experience of pain can contribute to the development of psychological conditions such as anxiety and depression. The emotional distress caused by unrelieved pain can lead to a decreased desire to participate in life, social withdrawal, and a reduced sense of well-being. Additionally, chronic pain can affect cognitive function, including memory and concentration, impairing a person's ability to perform daily tasks or engage in meaningful activities.

(4) Lack of appropriate pain management can negatively impact the overall prognosis and treatment outcomes for individuals with cancer. Uncontrolled pain may result in suboptimal response to cancer treatments, such as chemotherapy or radiation therapy. Inadequate pain control can also lead to reduced adherence to treatment regimens, as patients may be less willing to undergo potentially uncomfortable or painful procedures. Effective pain management, on the other hand, can enhance a person's overall well-being, improve treatment compliance, and potentially support better outcomes in terms of disease control and survival.

(5) It is important to recognize that each person's experience with cancer pain is unique, and the consequences of untreated pain can vary. However, in general, untreated cancer pain can have a profound impact on multiple aspects of a person's life, including physical, emotional, social, and functional well-being. Addressing and managing cancer pain through a multidisciplinary approach that includes medication, physical therapy, psychological support, and palliative care can significantly improve quality of life and overall treatment outcomes.

**10, Is it good to use pain patches when experiencing cancer pain?**

(1) Pain patches can be beneficial for managing cancer pain. These patches typically contain medications such as opioids or nonsteroidal anti-inflammatory drugs (NSAIDs) that are absorbed through the skin and provide localized pain relief. They are designed to deliver a continuous dose of medication over a specific period, which can help maintain a consistent level of pain control. Pain patches can be particularly useful for individuals who have difficulty swallowing or tolerating oral medications, or for those who require constant pain relief throughout the day.

(2) Pain patches offer convenience and ease of use compared to other forms of pain medication. Once applied to the skin, the patches can provide long-lasting relief, usually ranging from a few hours to several days, depending on the specific product. They eliminate the need for frequent dosing or injections, allowing individuals to focus on their daily activities without constant interruption for pain management. This can improve the overall quality of life for cancer patients by minimizing the impact of pain on their daily routines.

(3) Pain patches can be advantageous in terms of targeted pain relief. By directly applying the patch to the area experiencing pain, the medication is delivered precisely to the affected site. This localized treatment can be particularly useful for individuals with cancer pain that is limited to a specific area, such as bone metastases or surgical sites. By bypassing the digestive system, pain patches can also reduce the risk of gastrointestinal side effects that may be associated with oral pain medications.

(4) It's important to note that pain patches should be used under the guidance of a healthcare professional. They are typically prescribed based on the individual's specific needs and pain severity. The healthcare provider will consider various factors, such as the type and location of the pain, the patient's overall health, and any potential drug interactions or allergies. It is crucial to follow the prescribed instructions for patch application and duration of use to avoid complications or misuse. Regular communication with the healthcare team is essential to ensure optimal pain management and address any concerns or adjustments needed.

Rep2:

(1) Pain patches can be a beneficial option for managing cancer pain. These patches contain medications, such as opioids or nonsteroidal anti-inflammatory drugs (NSAIDs), that are absorbed through the skin and provide localized pain relief. They can be particularly useful for individuals who have difficulty swallowing pills or require consistent, long-lasting pain control. Pain patches can be applied directly to the affected area, allowing the medication to be absorbed directly into the tissues, providing targeted relief.

(2) Pain patches offer a convenient and steady release of medication, maintaining a consistent level of pain relief over an extended period. This controlled release mechanism can be advantageous for cancer patients who experience chronic or persistent pain. Additionally, pain patches can help minimize the side effects associated with oral pain medications, such as gastrointestinal upset or sedation, as the medication is delivered directly to the site of pain.

(3) Pain patches can be a suitable option for cancer patients who require a multimodal approach to pain management. They can be used alongside other pain medications, such as oral opioids, to provide complementary relief. Pain patches are available in different strengths, allowing healthcare professionals to tailor the dosage to the individual's needs. They can be part of a comprehensive pain management plan that includes various strategies to address the different aspects of cancer-related pain.

(4) It is important to consult with a healthcare professional, such as an oncologist or pain specialist, before using pain patches or making any changes to pain management strategies. They can evaluate the specific needs and circumstances of the individual and provide personalized recommendations. Healthcare professionals can assess the appropriateness of pain patches based on factors such as the type and severity of pain, the individual's overall health, and any existing medication regimens. They can also monitor for potential side effects or complications and adjust the treatment plan accordingly.

**11, Do I need to increase the dosage of painkillers when experiencing cancer pain, or should I switch to a different medication?**

(1) When experiencing cancer pain, it is essential to consult with a healthcare professional who specializes in pain management, such as an oncologist or palliative care specialist. They will assess your specific situation and provide personalized recommendations. In some cases, increasing the dosage of painkillers may be appropriate to effectively manage the pain. However, it is crucial to follow the prescribed dosage and not exceed it without medical guidance.

(2) Switching to a different medication may be an option if the current painkillers are not adequately managing the cancer pain or if you are experiencing intolerable side effects. Different medications work in various ways to target pain, and a healthcare professional can evaluate which alternative medication may be more suitable for your specific pain type and severity.

(3) Another aspect to consider is combining different pain management strategies. This can include using a combination of medications with different mechanisms of action, such as opioids, nonsteroidal anti-inflammatory drugs (NSAIDs), or adjuvant medications like antidepressants or anticonvulsants. By combining approaches, healthcare professionals aim to provide better pain relief and minimize potential side effects.

(4) Additionally, non-pharmacological interventions can complement medication-based pain management. Techniques such as physical therapy, acupuncture, massage, or relaxation exercises may help reduce cancer pain and improve overall well-being. Integrating these complementary approaches into your pain management plan can be discussed with your healthcare team to find the most effective combination for you.

Rep2:

(1) In general, it is advisable to consult with your healthcare provider when experiencing cancer pain. They are the best resource to guide you in managing your pain effectively. Depending on the severity of your pain and your individual circumstances, your healthcare provider may recommend increasing the dosage of your current painkiller. This can be done by adjusting the dose or frequency of administration, as prescribed. Increasing the dosage of painkillers should only be done under the supervision of a healthcare professional to ensure safety and effectiveness.

(2) Another possibility your healthcare provider might consider is switching to a different medication for cancer pain management. There are various types of painkillers available, including opioids, nonsteroidal anti-inflammatory drugs (NSAIDs), and adjuvant medications. The choice of medication depends on the nature and intensity of your pain, as well as any potential side effects and your overall health condition. It's crucial to have an open discussion with your healthcare provider to explore alternative medications that may provide better pain relief or fewer side effects.

(3) Your healthcare provider may also suggest a combination of approaches to address your cancer pain. This can include using a combination of different painkillers or utilizing additional therapies such as physical therapy, acupuncture, or nerve blocks. Complementary approaches like relaxation techniques and counseling may also be recommended to help manage pain and improve your overall well-being. Every individual's experience with cancer pain is unique, so a tailored approach based on your specific needs is essential.

(4) Remember, communication with your healthcare provider is key in managing cancer pain effectively. They can assess your pain, monitor your response to current medications, and adjust the treatment plan accordingly. It's important to report any changes in your pain level, side effects, or concerns to your healthcare provider promptly so that they can provide appropriate guidance and support throughout your cancer journey.

**12, Can different types of painkillers be taken simultaneously when experiencing cancer pain?**

(1) Yes, different types of painkillers can be taken simultaneously when experiencing cancer pain. This approach is known as multimodal analgesia or combination therapy. Cancer pain can be complex and may require a combination of pain medications to achieve adequate pain relief. Using multiple painkillers with different mechanisms of action can target pain from various angles and provide better pain control. It is important to consult with a healthcare professional who specializes in pain management to determine the appropriate combination of painkillers based on the individual's specific needs and medical condition.

(2) When managing cancer pain, healthcare professionals may prescribe different types of painkillers simultaneously to address different aspects of pain. For example, opioids such as morphine or oxycodone can be used to manage moderate to severe pain, while nonsteroidal anti-inflammatory drugs (NSAIDs) like ibuprofen or naproxen can help reduce inflammation and provide additional pain relief. Additionally, adjuvant medications such as antidepressants or anticonvulsants may be prescribed to target neuropathic pain that often accompanies cancer. By combining these painkillers, healthcare providers aim to optimize pain control and improve the patient's overall quality of life.

(3) The simultaneous use of different painkillers for cancer pain allows for a more comprehensive and tailored approach to pain management. Each painkiller has its own unique mechanism of action, targeting specific pain pathways or addressing different pain characteristics. By combining painkillers with different modes of action, synergistic effects can be achieved, resulting in better pain relief. Moreover, using multiple painkillers may allow for lower doses of individual medications, reducing the risk of side effects associated with higher doses of a single drug. However, it is crucial to follow the healthcare professional's guidance and dosage instructions to ensure safety and effectiveness.

(4) The combination of painkillers in cancer pain management can involve various classes of medications, including opioids, NSAIDs, adjuvant analgesics, and other supportive drugs. The specific combination and dosages depend on the individual's pain intensity, underlying condition, and response to treatment. It is essential to have open communication with the healthcare provider regarding pain levels, treatment efficacy, and any side effects experienced. Regular monitoring and adjustments may be necessary to optimize pain control while minimizing adverse effects. Always consult with a healthcare professional to determine the most appropriate combination of painkillers for managing cancer pain.

Rep2:

(1) Yes, different types of painkillers can be taken simultaneously when experiencing cancer pain. Cancer pain can vary in intensity and nature, and it often requires a multimodal approach to manage effectively. This approach may involve combining different classes of painkillers, also known as analgesics, to achieve better pain control.

(2) Non-opioid analgesics, such as nonsteroidal anti-inflammatory drugs (NSAIDs) and acetaminophen, can be used in conjunction with other painkillers. These medications work by reducing inflammation and inhibiting pain signals. They are commonly used for mild to moderate cancer pain and can be safely combined with other pain management options.

(3) Opioid analgesics, such as morphine, oxycodone, or fentanyl, are often prescribed for moderate to severe cancer pain. They work by binding to opioid receptors in the body, blocking pain signals, and providing relief. These medications can be used alongside non-opioid analgesics to enhance pain control.

(4) Adjuvant analgesics, such as antidepressants or anticonvulsants, may also be utilized to manage cancer pain. These medications are not primarily designed as painkillers but can effectively alleviate certain types of pain, such as neuropathic pain commonly experienced by cancer patients. They can be used in combination with other pain medications to address specific pain mechanisms.

**13, Are opioid drugs considered narcotics, and can taking them daily lead to addiction?**

(1) Yes, opioid drugs are considered narcotics. Narcotics refer to a class of drugs that are derived from or chemically similar to opium. Opioids, including both prescription painkillers and illegal substances like heroin, fall under this category. These drugs work by binding to opioid receptors in the brain, spinal cord, and other parts of the body, effectively reducing pain sensations.

(2) Taking opioids daily can indeed lead to addiction. Opioids are known to have a high potential for dependence and abuse. When used over an extended period, the brain can adapt to their presence, leading to tolerance. This means that higher doses are required to achieve the same effect, which can increase the risk of addiction. Additionally, opioids can cause euphoria and a sense of well-being, making them psychologically addictive.

(3) Addiction to opioids can have severe consequences. People who become addicted to opioids may experience withdrawal symptoms when they try to stop using the drug. These symptoms can be both physically and psychologically distressing and may include anxiety, agitation, nausea, vomiting, muscle aches, insomnia, and intense drug cravings. Addiction can also lead to social, occupational, and financial problems, as individuals prioritize obtaining and using opioids over other aspects of their lives.

(4) It is crucial to use opioids strictly as prescribed by a healthcare professional to minimize the risk of addiction. If you are concerned about the potential for addiction or experiencing difficulties with opioid use, it is essential to seek help and guidance from a healthcare provider. They can provide support, develop a tapering plan if necessary, and recommend alternative pain management strategies to minimize the risk of addiction.

Rep2:

(1) Opioid drugs are indeed considered narcotics. The term "narcotic" refers to a class of drugs that primarily act on the central nervous system to relieve pain and induce sedation. Opioids, including prescription painkillers like oxycodone and hydrocodone, as well as illicit drugs like heroin, fall under this category. They work by binding to opioid receptors in the brain, spinal cord, and other parts of the body, which can produce pain relief and euphoria.

(2) Taking opioid drugs daily can potentially lead to addiction. Opioids are highly addictive substances, and their regular use, even when taken as prescribed by a healthcare professional, can increase the risk of developing a substance use disorder. Prolonged use of opioids can lead to physical dependence, where the body adapts to the presence of the drug and requires higher doses to achieve the same effects. This physiological dependence, coupled with the rewarding properties of opioids, can drive individuals to continue using them despite negative consequences.

(3) Addiction to opioids can have severe consequences for an individual's health and well-being. Opioid addiction is characterized by compulsive drug-seeking behavior, loss of control over drug use, and continued use despite negative consequences. It can lead to various physical and mental health issues, including respiratory depression, constipation, sedation, confusion, depression, and increased risk of overdose. Addiction to opioids can also disrupt relationships, employment, and overall quality of life.

(4) It is important to note that not everyone who takes opioids will develop an addiction. However, the risk of addiction increases with long-term use and higher doses. Factors such as genetic predisposition, personal or family history of substance abuse, and concurrent mental health conditions can further contribute to the likelihood of addiction. It is crucial to use opioid medications strictly as directed by a healthcare professional and to be aware of the signs of addiction, seeking help promptly if addiction is suspected. Additionally, non-opioid alternatives for pain management should be explored whenever possible to minimize the risks associated with opioid use.

**14, Does long-term use of opioid drugs result in diminishing pain relief?**

(1) Prolonged use of opioid drugs can lead to diminishing pain relief. Initially, opioids provide effective pain relief by binding to opioid receptors in the brain and spinal cord, reducing the transmission of pain signals. However, over time, the body can develop a tolerance to opioids, requiring higher doses to achieve the same level of pain relief. This tolerance occurs due to adaptations in the body's opioid receptors and neurochemical systems, leading to reduced sensitivity and response to the drug.

(2) With continued use of opioids, the phenomenon of opioid-induced hyperalgesia (OIH) can occur, which exacerbates pain rather than relieving it. OIH is a paradoxical response where long-term opioid use actually increases sensitivity to pain. It is believed to result from neuroadaptive changes in the central nervous system, including heightened excitability of pain pathways and enhanced pain perception. Consequently, individuals may experience a decreased efficacy of opioids for pain relief and even heightened pain sensitivity.

(3) Another factor contributing to diminishing pain relief is the development of physical dependence. Prolonged opioid use can lead to physiological changes in the body, resulting in dependence. When individuals become dependent on opioids, abruptly reducing or discontinuing the medication can trigger withdrawal symptoms, including increased pain perception. This can create a cycle where individuals require opioids not only for pain relief but also to prevent withdrawal symptoms, making it challenging to achieve optimal pain management.

(4) In addition, the long-term use of opioids can have neuroplastic effects on the brain. Chronic opioid exposure can cause changes in the brain's reward and pain pathways, leading to altered pain processing and reduced efficacy of the drugs over time. These neuroplastic changes can contribute to the diminishing pain relief observed with prolonged opioid use.

Rep2:

(1) Yes, long-term use of opioid drugs can result in diminishing pain relief. Opioids work by binding to specific receptors in the brain and spinal cord to reduce pain signals. However, over time, the body can develop tolerance to the effects of opioids, requiring higher doses to achieve the same level of pain relief. This diminishing response to opioids is known as tolerance.

(2) Tolerance occurs because the brain's receptors adapt to the continuous presence of opioids. With prolonged use, the receptors become less responsive to the drug, leading to reduced pain relief. This diminishing effect necessitates higher opioid doses, which can increase the risk of side effects and opioid-related complications.

(3) Additionally, long-term opioid use can result in a phenomenon called opioid-induced hyperalgesia. This condition causes increased sensitivity to pain, meaning that individuals may experience heightened pain sensations even with the presence of opioids. Opioid-induced hyperalgesia can further complicate pain management and reduce the effectiveness of opioid therapy.

(4) It is important to note that long-term opioid use carries significant risks, including the potential for addiction, dependence, and overdose. Therefore, healthcare professionals carefully monitor patients on chronic opioid therapy and explore alternative pain management strategies, such as non-opioid medications, physical therapy, psychological interventions, and interventional procedures, to minimize the need for long-term opioid use.

**15, Why is there no bowel movement for many days after taking opioid medications?**

(1) Opioid medications can cause constipation by affecting the normal functioning of the digestive system. Opioids bind to specific receptors in the gastrointestinal tract, including the intestines, which reduces the muscular contractions that move waste through the digestive system. This reduction in muscular activity slows down the passage of stool, resulting in constipation. Additionally, opioids can also cause the muscles in the anal sphincter to tighten, making it harder to pass stool.

(2) Another reason for the lack of bowel movement after taking opioid medications is their effect on water absorption in the intestines. Opioids can increase the absorption of water from the intestines, which leads to drier and harder stools. This dehydration of the stool makes it more difficult to pass through the digestive system and contributes to constipation.

(3) Opioids can also affect the production of certain substances in the body that regulate bowel movements. For example, they can decrease the production of substances like serotonin in the intestines. Serotonin plays a role in promoting bowel movements by stimulating the muscles in the intestines. The decreased levels of serotonin caused by opioids can result in slower movement of stool through the digestive system, leading to constipation.

(4) Additionally, opioids can affect the nervous system and disrupt the coordination between the muscles in the intestines, making them contract less frequently and less efficiently. This disruption in the normal muscle activity necessary for propelling stool through the digestive tract can contribute to delayed bowel movements and constipation.

Rep2:

(1) Opioid medications, such as morphine, codeine, or oxycodone, can cause a decrease in bowel movements due to their effects on the gastrointestinal system. These medications bind to opioid receptors in the central nervous system, which can lead to reduced activity in the gastrointestinal tract. The decreased motility of the intestines can slow down the passage of stool through the digestive system, resulting in delayed or infrequent bowel movements.

(2) Opioids can also affect the muscles in the intestines, causing them to contract less frequently and with less force. This reduction in intestinal muscle activity, known as decreased peristalsis, further contributes to the delay in bowel movements. The decreased muscle contractions make it more difficult for the stool to move through the intestines, leading to constipation.

(3) Additionally, opioids can increase the reabsorption of water from the intestines, making the stool harder and drier. This increased water absorption, combined with reduced intestinal motility, creates an environment conducive to the formation of hard and compacted stools. The harder stool is more challenging to pass, leading to straining and further exacerbating constipation.

(4) Furthermore, opioid medications can affect the secretion of fluids into the intestines. Normally, the digestive system secretes fluids to aid in the digestion and movement of food through the intestines. Opioids can interfere with this fluid secretion, reducing the lubrication within the intestines and making it harder for the stool to pass through.

**16, Is a Demerol safe and effective painkiller?**

(1) Demerol, also known by its generic name meperidine, can be safe and effective for pain relief when used as prescribed by a healthcare professional. However, like all opioid painkillers, it carries a risk of dependence, addiction, and overdose, especially if it is misused or taken in larger doses than prescribed.

(2) It is important to follow the dosage instructions and precautions provided by your doctor or pharmacist, to never share Demerol with others, and to promptly report any side effects or concerns to your healthcare provider. Additionally, alternative pain management options, such as non-opioid analgesics or physical therapy, should be considered and discussed with your doctor.

Rep2:

(1) Demerol, also known as meperidine, is a prescription opioid pain medication that is used to treat moderate to severe pain. It can be effective at relieving pain, but it also comes with risks and potential side effects.

(2) While Demerol can be safe and effective when used as directed by a healthcare professional, it is important to understand that it can also be habit-forming and lead to dependence or addiction. It can also cause side effects such as nausea, dizziness, drowsiness, constipation, and difficulty breathing.

(3) Due to these risks, Demerol is typically not the first choice for pain relief and is usually only used for short-term pain management in specific situations, such as in postoperative or labor pain. It is important to follow your healthcare professional's instructions carefully and to use Demerol only as prescribed. If you experience any concerning side effects or have any questions or concerns about Demerol or other pain medications, you should speak with your healthcare provider.

**17, Does taking painkillers affect the effectiveness of radiation therapy and chemotherapy?**

(1) Painkillers, such as nonsteroidal anti-inflammatory drugs (NSAIDs) or opioids, typically do not affect the effectiveness of radiation therapy and chemotherapy. These medications primarily target pain relief and do not directly interfere with the mechanisms of these cancer treatments. Therefore, patients can usually take painkillers as prescribed to manage their pain without compromising the effectiveness of radiation therapy or chemotherapy.

(2) Radiation therapy works by damaging the DNA of cancer cells, which leads to their destruction. Chemotherapy, on the other hand, uses drugs to kill or slow down the growth of cancer cells. Painkillers do not interfere with these mechanisms. Instead, they act on the nervous system to alleviate pain sensations. Therefore, taking painkillers alongside radiation therapy or chemotherapy is generally considered safe and does not impact the effectiveness of these treatments.

(3) It's important to note that managing pain during cancer treatment is crucial for a patient's well-being and quality of life. Pain can arise due to various factors, including the cancer itself or the side effects of treatment. By effectively controlling pain, patients can experience improved comfort and a better ability to carry out daily activities. Painkillers play a vital role in achieving this pain management goal without negatively affecting the outcomes of radiation therapy or chemotherapy.

(4) However, it's essential for patients to consult with their healthcare team before taking any medications, including painkillers, during cancer treatment. Healthcare professionals can provide personalized advice based on the specific type and stage of cancer, as well as the individual's overall health condition. They can recommend suitable pain management strategies and medications while considering any potential interactions with the ongoing radiation therapy or chemotherapy. Open communication with the healthcare team is crucial to ensure comprehensive and safe cancer care.

Rep2:

(1) Painkillers, such as nonsteroidal anti-inflammatory drugs (NSAIDs) and opioids, generally do not affect the effectiveness of radiation therapy and chemotherapy. The primary purpose of painkillers is to alleviate pain and provide comfort to patients undergoing cancer treatment. These medications target pain receptors and do not directly interfere with the mechanisms of radiation or chemotherapy.

(2) Radiation therapy and chemotherapy work by targeting and damaging cancer cells, inhibiting their growth, and ultimately causing their death. Painkillers, on the other hand, function by blocking pain signals or reducing inflammation. As pain management is an important aspect of cancer treatment, painkillers are often prescribed to patients to improve their quality of life. While painkillers can have side effects, they generally do not impact the efficacy of radiation therapy or chemotherapy.

(3) It is important for patients undergoing radiation therapy or chemotherapy to communicate with their healthcare team regarding their pain levels and the use of painkillers. The healthcare professionals can provide guidance on the appropriate pain management strategies that align with the specific cancer treatment plan. They may suggest certain pain medications or dosage adjustments based on the individual's needs and potential interactions with other medications.

(4) Overall, taking painkillers as prescribed should not significantly affect the effectiveness of radiation therapy or chemotherapy. Pain management is crucial for patients to cope with the side effects and discomfort associated with cancer treatment. It is essential to maintain open communication with healthcare providers to ensure optimal pain control while undergoing radiation therapy or chemotherapy.

**18, Can cancer pain be completely relieved, and when can painkillers be discontinued?**

(1) Cancer pain can often be effectively managed and reduced to a tolerable level, but complete relief may not always be possible. The goal of pain management in cancer patients is to enhance their quality of life by minimizing pain and discomfort. A multimodal approach is typically employed, combining various interventions such as medication, physical therapy, psychological support, and alternative therapies. The selection of painkillers and treatment strategies depends on the type and severity of pain, as well as the individual's overall health and preferences.

(2) Painkillers are typically used in cancer pain management to alleviate symptoms and improve the patient's well-being. Medications such as opioids (e.g., morphine, oxycodone) are commonly prescribed for moderate to severe pain. Adjuvant medications, including nonsteroidal anti-inflammatory drugs (NSAIDs), anticonvulsants, and antidepressants, may also be used to address specific aspects of pain or to enhance the effectiveness of opioids. The dosage and frequency of painkillers are adjusted based on the individual's response and the level of pain control achieved.

(3) Discontinuing painkillers in cancer patients should be done under the guidance of healthcare professionals and in consideration of the patient's pain status and overall treatment plan. Painkillers are typically tapered gradually to prevent withdrawal symptoms or rebound pain. The decision to discontinue painkillers depends on several factors, including the underlying cause of pain, the effectiveness of other pain management strategies implemented, and the patient's overall condition and preferences. It is important to note that painkillers may still be required intermittently or on an as-needed basis, even when pain is well-controlled.

(4) Pain management in cancer patients is an ongoing process that requires regular assessment and adjustments. The goal is to achieve an optimal balance between pain control and minimizing medication side effects. In some cases, pain may become more manageable over time, allowing for the reduction or discontinuation of painkillers. However, some patients may require long-term pain management or palliative care to maintain a satisfactory quality of life. The decision to discontinue painkillers should always be made in consultation with the healthcare team to ensure the patient's comfort and well-being.

Rep2:

(1) Cancer pain can often be effectively managed and significantly relieved, but complete relief may not always be possible. The goal of pain management in cancer patients is to minimize pain and maximize their quality of life. Various treatment options are available, including medications, such as opioids, nonsteroidal anti-inflammatory drugs (NSAIDs), and adjuvant analgesics. These medications can help alleviate pain by targeting different mechanisms within the body.

(2) The decision to discontinue painkillers in cancer patients is not based on a fixed timeline but rather on an individualized approach. Discontinuation of painkillers depends on several factors, such as the underlying cause of the pain, the effectiveness of the treatment, and the patient's overall condition. In some cases, if the cancer is successfully treated or goes into remission, the need for painkillers may decrease or even cease. However, it is essential to consult with healthcare professionals who can assess the situation and determine the appropriate time to discontinue pain medications.

(3) It is important to note that pain management in cancer patients is a dynamic process. As the disease progresses or if new symptoms arise, pain management strategies may need to be adjusted. Healthcare providers will continuously evaluate the effectiveness of pain medications and consider alternative approaches, such as interventional pain procedures or palliative care, to ensure optimal pain control and comfort. The primary focus is on maintaining the patient's comfort and overall well-being throughout their cancer journey.

(4) The goal of cancer pain management is to provide relief and improve the patient's quality of life. While complete relief of pain may not always be attainable, advancements in pain management techniques and medications have significantly improved the ability to control cancer-related pain. Regular communication between the patient and their healthcare team is crucial to adjust pain management strategies, and the decision to discontinue painkillers should be made in consultation with medical professionals based on individual circumstances.

**19, If severe cancer pain occurs, does it mean that one won't live for long?**

(1) Severe cancer pain does not necessarily mean that one won't live for long. The presence of severe pain in cancer patients can be attributed to various factors, such as the location and extent of the tumor, nerve involvement, and the individual's pain threshold. While the presence of severe pain can be distressing and indicate advanced disease progression in some cases, it is not a definitive predictor of life expectancy. Many factors, including the type and stage of cancer, overall health status, treatment options, and individual response to treatment, contribute to determining the prognosis.

(2) It is important to note that the experience of severe cancer pain does not directly correlate with a shortened lifespan. Cancer pain management has improved significantly over the years, with various medications and interventions available to alleviate pain and improve quality of life. The treatment of cancer pain is a critical aspect of palliative care, aimed at providing relief and improving overall well-being. Therefore, even if someone experiences severe pain, it is possible to address it effectively, enabling them to live longer with an improved quality of life.

(3) The occurrence of severe cancer pain does not necessarily indicate a limited life expectancy. Pain is a complex symptom that can arise from different causes, including cancer itself or its treatment. Multidisciplinary approaches to pain management, including medications, physical therapy, psychological support, and complementary therapies, have proven effective in relieving severe pain and enhancing the patient's ability to function and enjoy life. It is crucial to consult with healthcare professionals who can assess the individual's condition, develop a tailored pain management plan, and provide support throughout the cancer journey.

(4) While severe cancer pain can be distressing, it is important not to solely equate it with a poor prognosis. Each cancer case is unique, and survival rates vary depending on factors such as cancer type, stage, overall health, and treatment response. Advances in cancer treatments, including surgery, chemotherapy, radiation therapy, targeted therapies, and immunotherapy, have significantly improved outcomes for many patients. Palliative care specialists and pain management teams can work together to develop an individualized approach to pain control, aiming to enhance comfort, maintain functionality, and optimize the patient's quality of life.

Rep2:

(1) Severe cancer pain does not necessarily indicate that one won't live for long. Pain can be a common symptom experienced by individuals with cancer, but its severity does not provide a definitive prognosis. The prognosis depends on various factors such as the stage of cancer, treatment options available, response to treatment, and overall health of the individual. It's important to consult with healthcare professionals to understand the specific situation and explore appropriate pain management strategies.

(2) The presence of severe cancer pain should not be solely interpreted as an indication of impending death. Cancer pain can result from various factors such as tumor growth, nerve compression, inflammation, or treatment side effects. Effective pain management approaches, including medications, radiation therapy, surgery, and complementary therapies, can often provide relief and improve the quality of life for individuals with cancer. Prognosis is determined by comprehensive evaluation and assessment of multiple factors, not solely based on pain intensity.

(3) Severe cancer pain does not serve as a direct predictor of life expectancy. While cancer pain can be distressing, advances in pain management techniques have significantly improved the ability to control and alleviate pain in cancer patients. The prognosis for individuals with cancer depends on numerous factors, including cancer type, stage, treatment options, and individual response to treatment. It is essential to consult with healthcare professionals to develop a personalized treatment plan and address pain management concerns.

(4) The presence of severe cancer pain does not definitively determine how long an individual will live. Pain is a subjective experience and can vary greatly among individuals. Additionally, pain perception and tolerance can differ widely. Cancer pain management focuses on improving quality of life and ensuring optimal comfort for patients. Prognosis is determined by comprehensive assessment of the cancer stage, treatment options, overall health, and response to therapy. Effective pain management strategies are available to address and alleviate severe cancer pain, promoting better quality of life for individuals living with cancer.

**20, What is the solution if there is a needle-like or electric shock-like pain accompanied by numbness, and painkillers don't work?**

(1) One possible solution for needle-like or electric shock-like pain accompanied by numbness, which does not respond to painkillers, is to consult a medical professional. These symptoms could be indicative of a nerve-related condition or injury, such as a pinched nerve, nerve compression, or neuropathy. A healthcare provider can perform a thorough examination, potentially order diagnostic tests, and provide a proper diagnosis and treatment plan based on the underlying cause of the symptoms.

(2) Another potential solution is to explore non-pharmacological pain management techniques. These may include physical therapy, chiropractic care, acupuncture, or transcutaneous electrical nerve stimulation (TENS). Physical therapy can help improve muscle strength, flexibility, and posture, which can alleviate pressure on nerves and reduce pain. Chiropractic care focuses on aligning the spine to relieve nerve impingement. Acupuncture involves the insertion of thin needles into specific points on the body to promote pain relief and balance energy flow. TENS therapy utilizes low-voltage electrical currents to stimulate nerves and reduce pain.

(3) In some cases, the underlying cause of the pain and numbness may require specific medical interventions. For example, if a herniated disc is compressing a nerve, surgery may be necessary to alleviate the pressure and provide relief. Alternatively, if the symptoms are due to a chronic condition such as diabetic neuropathy or multiple sclerosis, a healthcare professional can prescribe medications or treatments targeted at managing the underlying condition and its associated symptoms.

(4) It is crucial to communicate openly and honestly with healthcare professionals regarding the ineffectiveness of painkillers. They can explore alternative medication options, adjust dosages, or consider other pain management strategies. It may also be helpful to keep a pain diary, noting the frequency, intensity, and duration of the symptoms, as well as any triggers or alleviating factors. This information can assist the healthcare provider in making an accurate diagnosis and developing an individualized treatment plan.

Rep2:

(1) If you experience needle-like or electric shock-like pain accompanied by numbness, and painkillers don't provide relief, it is important to consult a healthcare professional for a proper diagnosis and appropriate treatment. The symptoms you described could be indicative of various conditions, and a medical evaluation is necessary to determine the underlying cause.

(2) One possible cause of needle-like or electric shock-like pain accompanied by numbness is a nerve-related condition such as neuropathy. Neuropathy occurs when there is damage or dysfunction to the nerves, leading to abnormal sensations and pain. In such cases, treatment may involve medications specifically targeting nerve pain, physical therapy, or other interventions recommended by a healthcare professional.

(3) Another potential cause of your symptoms could be a spinal disc issue, such as a herniated disc. When a disc in the spine becomes herniated or ruptured, it can compress nearby nerves, leading to pain and numbness that radiates into the affected area. Treatment options for herniated discs may include physical therapy, pain management techniques, or in some cases, surgery.

(4) It is also possible that your symptoms could be related to a condition called radiculopathy, which occurs when a nerve root is compressed or irritated as it exits the spinal column. Radiculopathy can cause sharp, shooting pain along the affected nerve pathway, along with numbness or weakness. Treatment for radiculopathy may involve a combination of medications, physical therapy, and possibly injections or surgical intervention.

**21, Are there any other pain relief methods besides oral analgesics for cancer pain?**

(1) Transdermal patches: Transdermal patches are one alternative method for pain relief in cancer patients. These patches contain medications that are absorbed through the skin and into the bloodstream, providing a continuous release of pain-relieving medication. They can be convenient for patients who have difficulty swallowing oral medications or who require long-term pain management.

(2) Epidural analgesia: Epidural analgesia involves the administration of pain medication directly into the space around the spinal cord. This method is commonly used for managing severe cancer pain. An epidural catheter is inserted into the epidural space, and medication, such as opioids or local anesthetics, is delivered through the catheter to provide targeted pain relief. This approach can be particularly effective for pain that affects specific areas of the body.

(3) Nerve blocks: Nerve blocks are a technique used to interrupt the transmission of pain signals along specific nerves. By injecting local anesthetics or other medications near the nerves responsible for transmitting pain, nerve blocks can provide targeted pain relief for cancer patients. This method is especially useful for localized pain or pain originating from specific nerve pathways.

(4) Radiotherapy: Radiotherapy, also known as radiation therapy, can be employed to alleviate cancer-related pain. High-energy beams are directed at the tumor or metastatic sites, helping to shrink or destroy cancer cells and reduce pain. Radiotherapy is commonly used in palliative care to improve quality of life by reducing pain symptoms in patients with advanced cancer.

(5) Intrathecal drug delivery: Intrathecal drug delivery involves the direct administration of pain-relieving medications into the spinal fluid. A small pump is surgically implanted under the skin, and a catheter is placed into the intrathecal space, allowing medications like opioids to be delivered directly to the spinal cord. This method can provide effective pain relief while using lower doses of medication compared to oral administration, thereby reducing systemic side effects.

(6) Complementary therapies: Complementary therapies, such as acupuncture, massage, and relaxation techniques, can be used alongside traditional pain management approaches to provide additional pain relief for cancer patients. While these methods may not directly target the cancer pain itself, they can help alleviate associated symptoms and improve overall well-being, leading to a reduction in pain perception. It's important to discuss these options with healthcare professionals to ensure they are safe and appropriate for individual patients.

Rep2:

(1) Yes, there are several other pain relief methods besides oral analgesics for cancer pain. One such method is transdermal patches or topical creams that contain analgesic medications. These patches or creams are applied directly to the skin, allowing the medication to be absorbed and provide localized pain relief. They can be particularly useful for managing pain in specific areas, such as joint or muscle pain.

(2) Another pain relief method for cancer pain is the use of intravenous (IV) medications. IV analgesics can be administered directly into a vein, allowing for rapid pain relief. This method is often used in situations where immediate pain relief is required or when oral medications are not effective. IV opioids, such as morphine, are commonly used for severe cancer pain.

(3) Interventional pain management techniques can also be employed for cancer pain relief. These techniques involve procedures that target specific nerves or regions to block pain signals. Examples include nerve blocks, where local anesthetics or steroids are injected near specific nerves to numb the area and relieve pain, and neurolytic blocks, which involve the use of chemicals or radiofrequency heat to destroy the nerves responsible for transmitting pain signals.

(4) Palliative radiation therapy is another pain relief method used for cancer pain management. It involves the use of high-energy radiation to target and shrink tumors or reduce their effects on surrounding tissues. By reducing tumor size and alleviating pressure on nerves or organs, radiation therapy can effectively relieve pain in cancer patients.

(5) Complementary therapies, such as acupuncture, massage therapy, and relaxation techniques, can also play a role in managing cancer pain. These therapies aim to enhance overall well-being, reduce stress, and promote relaxation, which can help alleviate pain and improve quality of life.

(6) In some cases, invasive procedures like nerve ablation or neurostimulation may be considered. Nerve ablation involves using heat, cold, or chemicals to destroy or disable specific nerves that are causing pain. Neurostimulation, on the other hand, uses electrical impulses to block pain signals from reaching the brain. These procedures are typically reserved for severe, chronic cancer pain that has not responded to other treatments.
